# Novel nicotinoid structures for covalent modification of wood: an environmentally friendly way for its protection against insects†‡

**DOI:** 10.1039/d0ra02071k

**Published:** 2020-04-21

**Authors:** Martin Söftje, Sophie Acker, Rudy Plarre, Jan C. Namyslo, Dieter E. Kaufmann

**Affiliations:** Institute of Organic Chemistry, Clausthal University of Technology Leibnizstraße 6 38678 Clausthal-Zellerfeld Germany dieter.kaufmann@tu-clausthal.de; Bundesanstalt für Materialforschung und -prüfung Unter den Eichen 87 12205 Berlin Germany

## Abstract

Timber is constantly exposed to environmental influences under outdoor conditions which limits its lifetime and usability. In order to counteract the damaging processes caused by insects, we have developed a novel and more environmentally friendly method to protect wood materials *via* covalent modification by organic insecticides. Starting with an important class of synthetic insecticides which are derived from the natural insecticide nicotine, various new carboxylic acid derivatives of imidacloprid were made accessible. These activated neonicotinoids were utilized for the chemical modification of wood hydroxy groups. In contrast to conventional wood preservation methods in which biocides are only physically bound to the surface for a limited time, the covalent fixation of the preservative guarantees a permanent effect against wood pests, demonstrated in standardized biological tests. Additionally, the environmental interaction caused by non-bound neonicotinoids is significantly reduced, since both, a smaller application rate is required and leaching of the active ingredient is prevented. By minimizing the pest infestation, the lifetime of the material increases while preserving the natural appearance of the material.

## Abstract 2

A novel and eco-friendly procedure for durable wood protection applying covalently bound organic insecticides is presented. Biological tests confirmed the effectiveness of the method, which also reduces the environmental impact.

## Graphical abstract

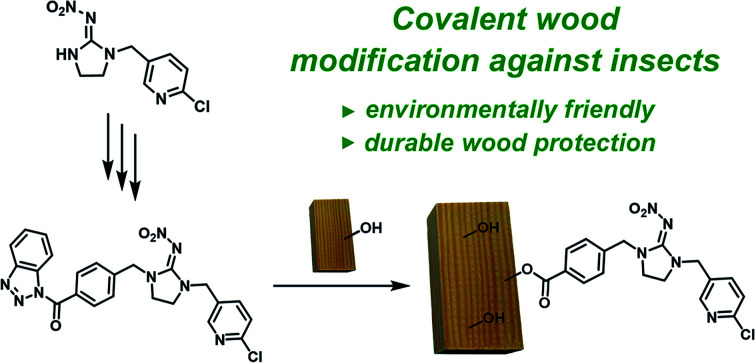

## Introduction

The renewable natural resource wood is one of the oldest construction materials in the world and remains of great interest due to its recoverability and its good mechanical, acoustical, and thermal properties.^1^ However, the CO_2_ neutral, unprotected biomaterial suffers from significant environmental impacts which limit its period of use. Wood products and constructions are prone to attack or even complete destruction by insects such as termites or beetle larvae. In the subtropical and tropical regions termites are widespread and the most important pests on wood.^2,3^ Whereas in moderate regions like Central to Northern Europe the house longhorn beetle and the common wood worm are economically most relevant. The primary damage to the wood is caused by the feeding larvae, whereas the grown insects cause no further damage to the wood and only pursue their reproductive instinct.

The damage caused to wood by insect attack can be significantly reduced through applying suitable insecticides, such as neonicotinoids.^4–6^ In addition to its widespread disposition as a plant protection product, imidacloprid and its derivatives are also used as active ingredients in wood preservatives.^6,7^ The effect of the neonicotinoids is based on an interaction of the insecticide with the nicotinic acetylcholine receptor (nAChR) of the nerve cells of the insects. Neonicotinoids bind to the nAChR, which causes a continuous transmission of synaptic impulses and thereby an over-excitation of the central nervous system of the affected insects.^8^ The insecticides can be ingested orally or *via* the cuticle.^5^ This mode of action occurs selectively with insects. Therefore, at the current stage of knowledge, neonicotinoids with the amounts applied against insects are of no harm to mammals as well as to humans.^8^ However, this class of compounds has recently been criticized to cause damage on beneficial insects such as wild and honey bees.^9–12^ Neonicotinoids are suspected to be partly responsible for the massive decline of insects in recent years. This has led to restriction for the field use of the neonicotinoids imidacloprid, thiamethoxam, and clothianidin in the European Union in 2018.^7^

To protect wood-based materials against insects, neonicotinoids are still allowed to be used both for industrial wood preservation and also for private use. The insecticides can be applied as surface application or by impregnation.^7,13^ However, physically bound protective layers have proved to be disadvantageous in terms of durability, since they can be washed out easily. This leads to both, reapplication at regular intervals consuming large amounts of the insecticide and its continuous release into the environment through a leaching process.

Our group has developed a new and, in contrast to conventional impregnation, more environmentally friendly method for a covalent modification of wood through applying activated benzoic acids.^14–18^ By esterification of wood with benzotriazolyl-activated carboxylic acids, the material properties can be specifically influenced, while simultaneously retaining its outer appearance. The modification of wood pursues the goal to increase the lifetime of the natural material in order to improve the attractiveness and usability of the material. The modification of soft and fast-growing wood species is especially important since the optimization of their properties would allow to use such treated wood materials as a substitute for harder and slower growing tropical wood. The covalent modification of wood using neonicotinoids with an activated acid function opens up completely new opportunities to protect wood against insects. Our developed process is particularly environmentally friendly because organic insecticides, such as the controversial imidacloprid remains fully effective and yet will not be leached into the environment due to the hydrolytic stability especially of the benzoates, proven in previous work.^7^ Therefore, lower amounts of the insecticide will be required in the long term, since reapplication is avoided.

## Results and discussion

### Synthesis of the neonicotinoids

**Scheme 1:**
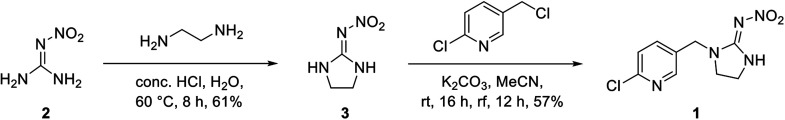
Synthesis of imidacloprid (1).

The neonicotinoid imidacloprid (1) occupies a synthetic key position on the route to protect wood against insects. As shown in Scheme 1 imidacloprid was synthesized from nitroguanidine (2) in a two-step synthetic pathway *via* imidazolidine 3 according to literature.^19–22^

**Scheme 2:**
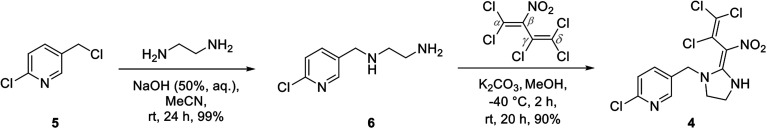
Synthesis of neonicotinoid 4.

As additional neonicotinoid we used butadiene derivative 4 which had previously been synthesized by Zapol'skii *et al.* starting from 2-chloro-5-(chloromethyl)pyridine 5 (Scheme 2).^23–26^

**Scheme 3:**
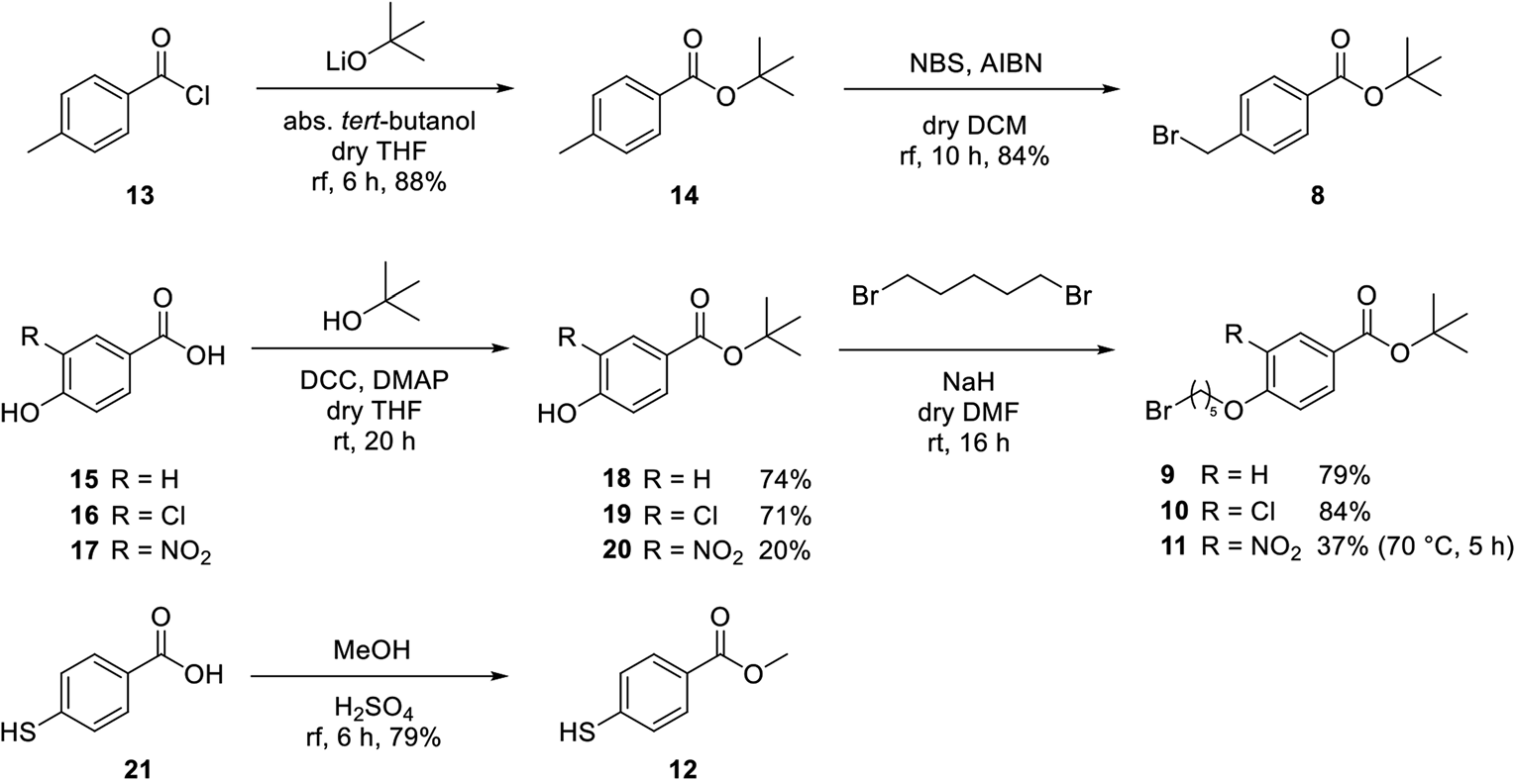
Synthesis of different linking units 8–12.

For covalent attachment of neonicotinoids 1 and 4 to wood, implementation of a carboxylic acid function into the respective insecticides was to achieve. This was done by substitution reactions using suitable linking units 7–12 carrying an ester-protected carboxylic acid group. In addition to commercially available *tert*-butyl bromoacetate (7), Scheme 3 shows the specific synthesis pathways of the linker molecules 8–12 following different literature procedures.^27–31^

**Scheme 4:**
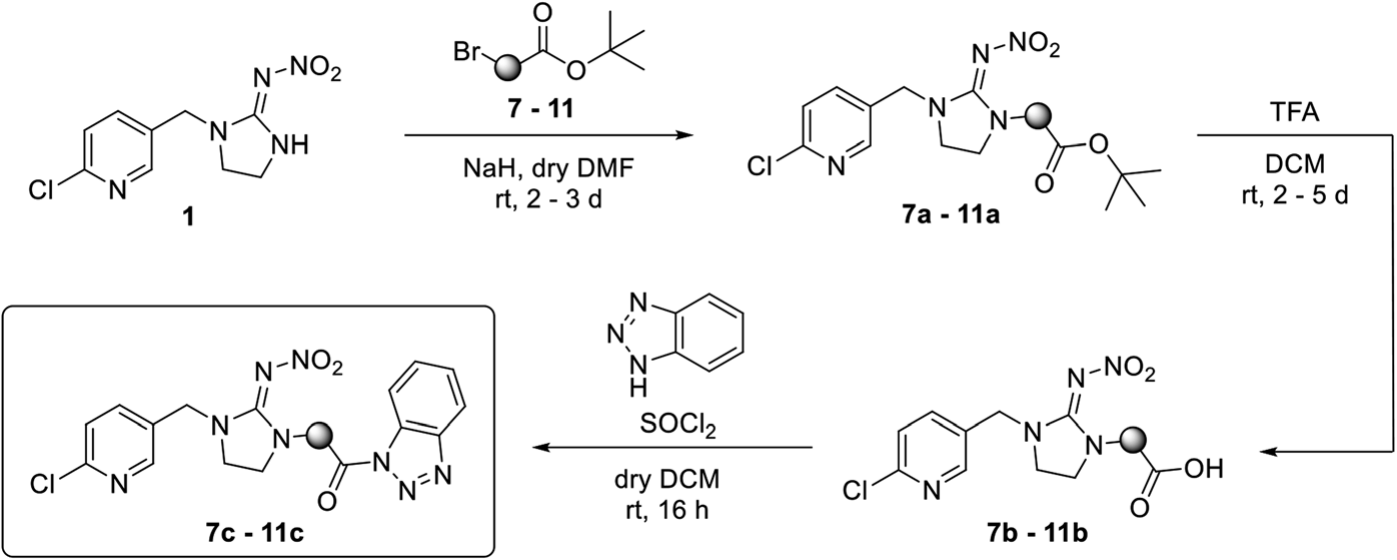
Synthesis of the activated neonicotinoids 7c–11c.

The linking units 7–11 are characterized by different distances between the carbonyl group and the insecticidal substructure. In order to attach the linker moieties 7–11 to imidacloprid (1), nucleophilic substitution reactions were carried out applying sodium hydride in dry DMF (Scheme 4), following a modified procedure from Kagabu *et al.*^32^ The substitution products 7a–11a have been isolated in good yields (Table 1). The ester groups were subsequently hydrolysed using trifluoroacetic acid in DCM according to Manicardi *et al.*^33,34^ The almost quantitatively obtained carboxylic acids 7b–11b were then activated with thionyl chloride and 1*H*-benzotriazole (Scheme 4). This reaction process, in which the carboxylic acid chloride first forms and then reacts *in situ* to the activated benzotriazolate, was developed by Katritzky.^35^ The resulting carboxylic acid amides 7c–11c are characterized by a high stability in combination with a high reactivity towards nucleophiles, such as lignocellulosic materials like wood. The structures of the products and the corresponding yields are given in Table 1.

**Table 1:** Structures and yields of the synthesized neonicotinoid derivatives 7a–11c

Linker	Substituted imidacloprid^a^	Hydrolysed imidacloprid	Activated imidacloprid^b^
7	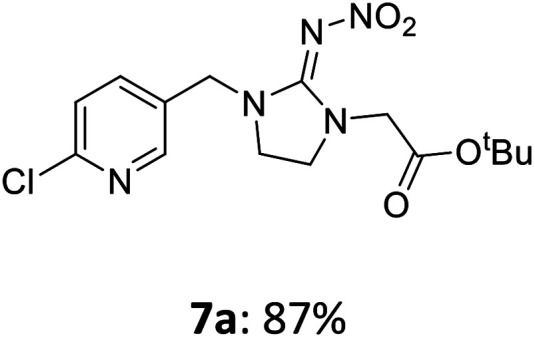	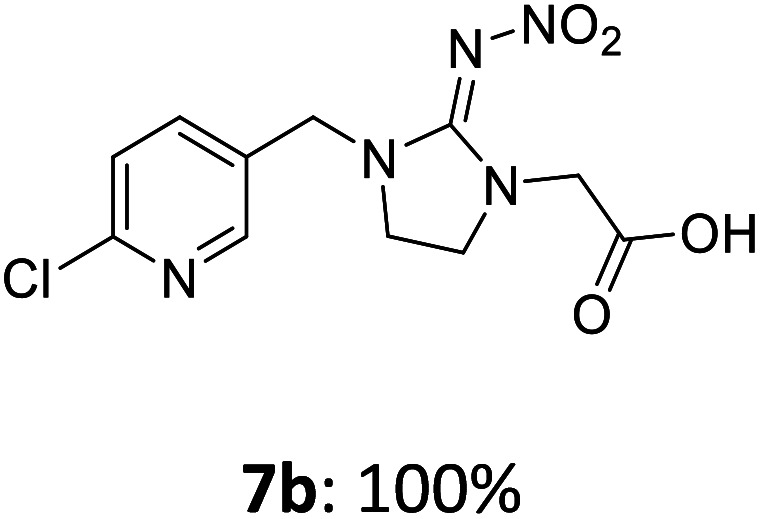	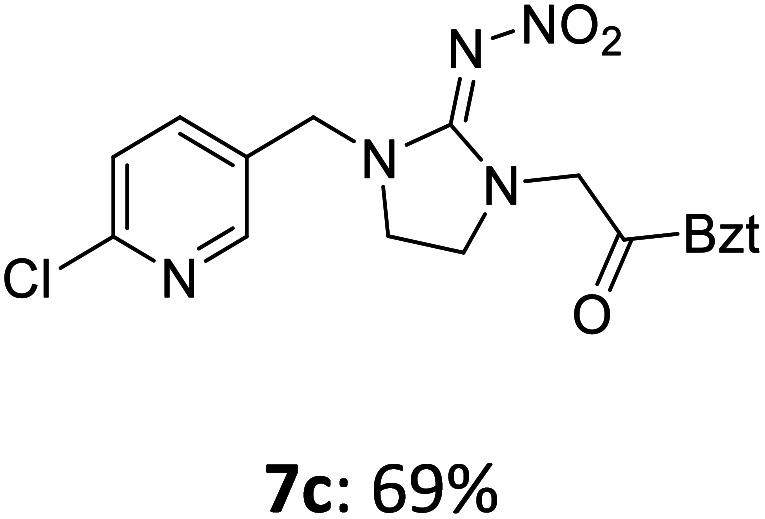
8	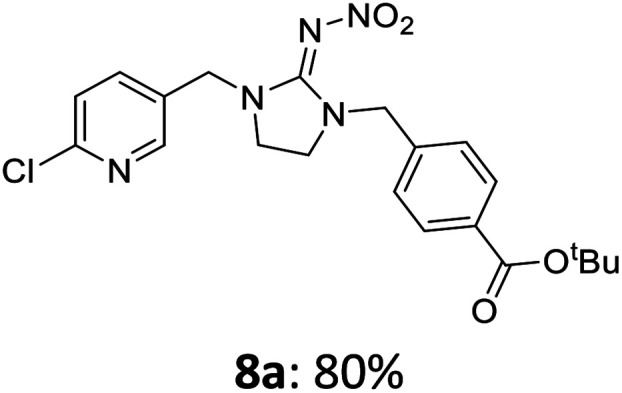	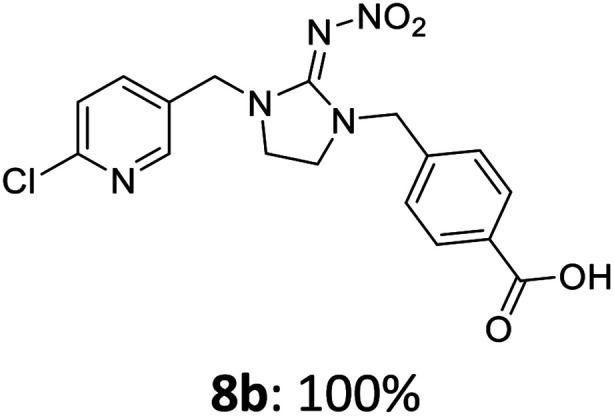	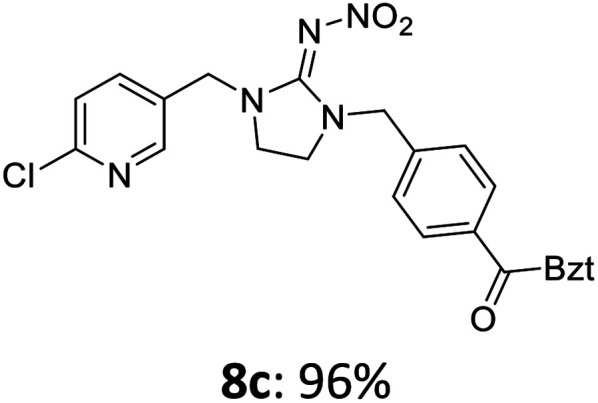
9	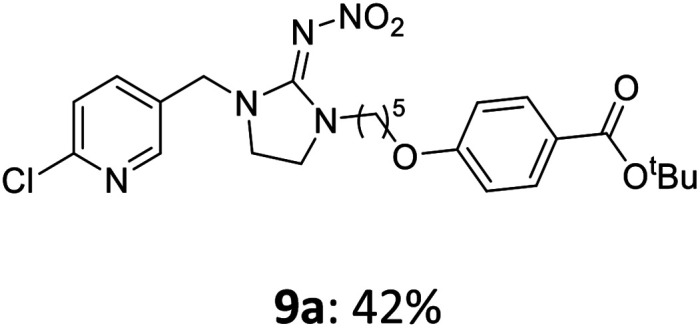	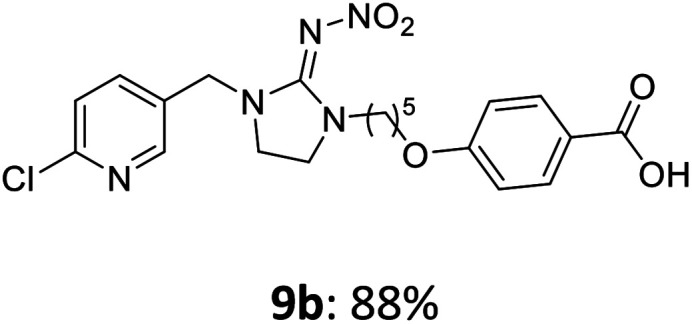	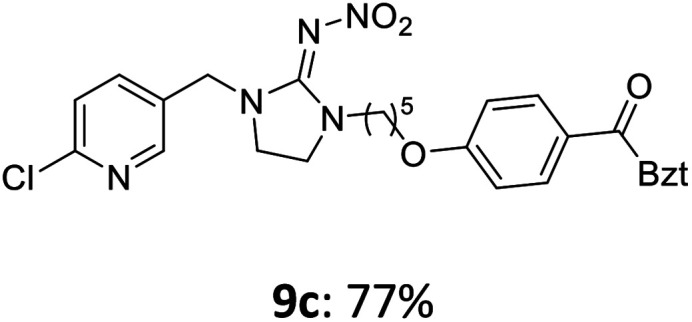
10	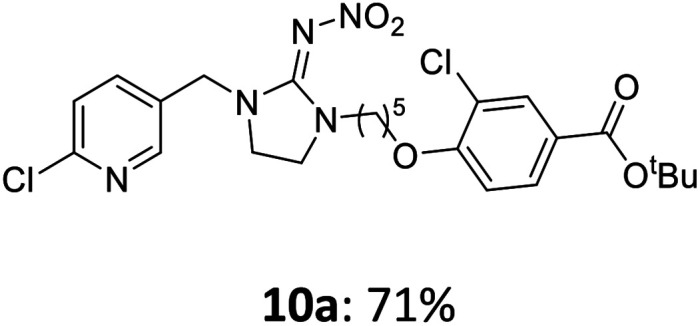	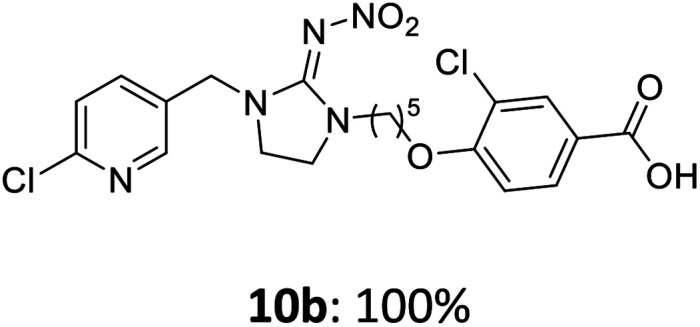	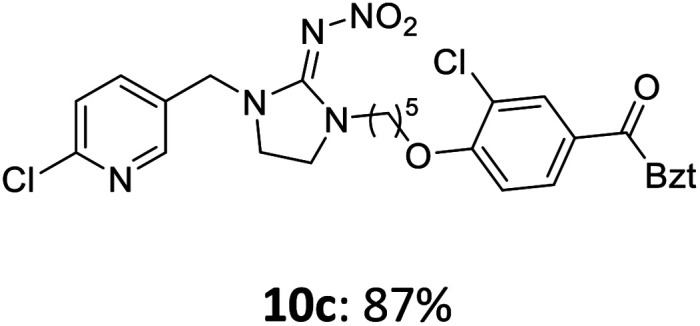
11	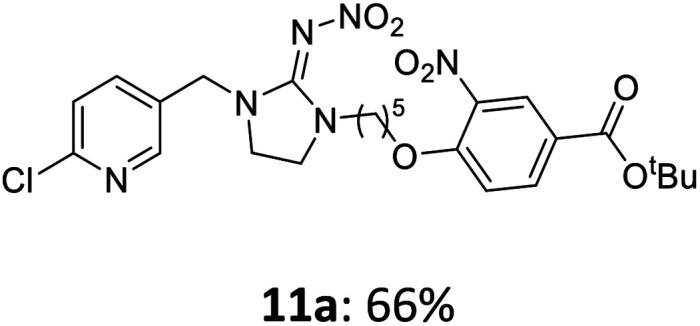	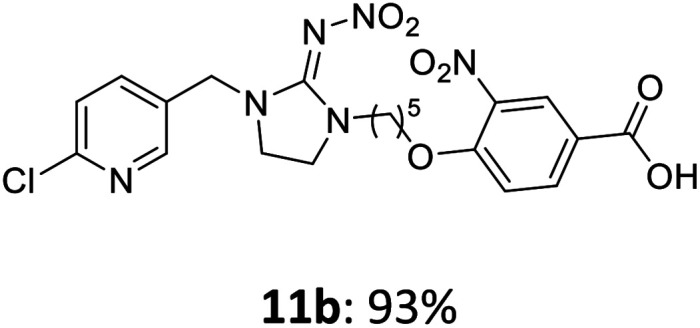	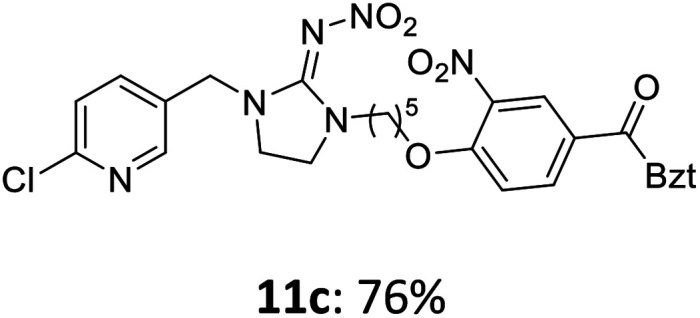

a ^*t*^Bu = *tert* butyl.

b Bzt = benzotriazol-1-yl.

**Scheme 5:**
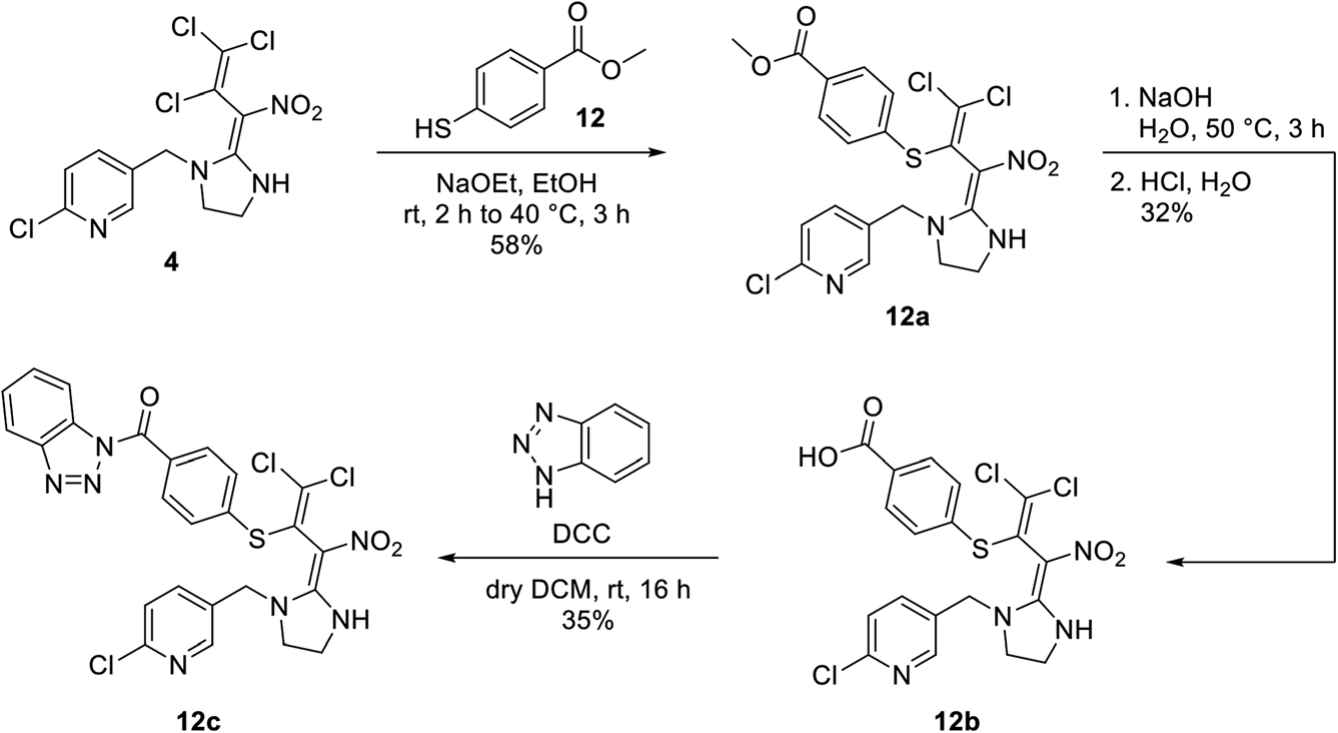
Synthesis of the activated butadiene neonicotinoid 12c.

Attempts have also been made to substitute the butadiene neonicotinoid 4 on the secondary amine followed by activation analogously to Scheme 4. Unfortunately, the substitution reaction failed as neonicotinoid 4 decomposed during the deprotonation with sodium hydride. The use of different bases such as sodium hydroxide also remained unsuccessful. However, the butadiene skeleton of neonicotinoid 4 provides additional potential positions for attaching a nucleophilic substrate. Therefore, a nucleophilic substitution in *γ*-position of the butadiene was carried out successfully using thiol linker 12. In the next step, the methyl ester 12a was saponified using an aqueous solution of sodium hydroxide. Acidification with conc. hydrochloric acid finally gave the carboxylic acid 12b which was then activated under the previously described conditions leading to 12c (Scheme 5).

### Covalent wood modification

**Scheme 6:**
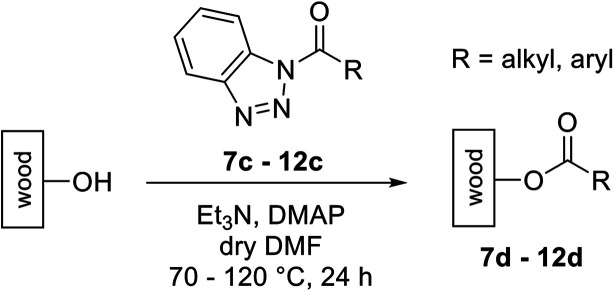
Modification reaction of wood.

The activated neonicotinoids 7c–12c were then applied in the wood modification process, developed previously in our group in order to improve the biological resistance against insects.^14–18^ At first, veneer chips of Scots pine sapwood were used for the modification process. To ensure a high sample throughput, the modifications were performed in the “Synthesis 1” liquid system parallel synthesizer. In principle, however, the functionalization can also be carried out in standard Schlenk tubes. During the chemical modification, a reaction occurs between the substrate and the hydroxy groups of the wood biopolymers. However, the alcohol groups present in the crystalline regions of the cellulose form strong hydrogen bonds between the polymer strands and therefore do not react.^1^ Consequently, the number of reactive units is significantly lower compared to the total number of hydroxy groups being present. Replacement experiments with tritiated water reported by Sumi revealed that an average of 6.9–8.0 mmol hydroxy groups per gram wood are accessible to chemical reactions.^36^ In addition, the number of accessible alcohol groups depends on the sample preparation, especially the drying process and the moisture content of the sample.^1,37,38^ Therefore, a mean value of 7.0 mmol of accessible hydroxy groups per gram wood is assumed. Prior to the modification, the wood sample was swollen under nitrogen atmosphere in a mixture of triethylamine and 4-dimethylaminopyridine (DMAP) in dry DMF for 2 h at 50 °C. During this initial process, the pore structures of the wood broaden so that the reactants can diffuse more easily into the material and react with the enlarged cell surface. Furthermore, a deprotonation of the wooden hydroxy groups already takes place during the swelling process, so that the following esterification is facilitated. After the addition of the activated neonicotinoid 7c–12c (7.0 mmol per gram wood), the sample reacted for 24 h at 70 °C and 120 °C, respectively. The reaction conditions are summarized in Scheme 6.

After the modification reaction, the wood samples were extracted and dried before determining the mass gain. The WPG (weight percentage gain) and QCO (quantity of covalently bonded organo-material) values summarized in Table 2 show, that all insecticides 7c–12c were successfully bound to pine wood leading to a significant weight gain. At 70 °C, the acetamide 7c and the benzamide 8c were bound with high amounts to the wood polymers (0.24–0.26 mmol g^−1^), whereas the pentoxy derivative 9c only led to a lower QCO value of 0.10 mmol g^−1^. However, the introduction of electron-withdrawing groups such as a chlorine substituent in 10c or a nitro group in 11c allowed to significantly increase the covalently bound amount of substance up to 0.29 mmol g^−1^ which is comparable to the results of 7c and 8c. At 120 °C, larger amounts of the substances were usually tethered than at 70 °C. Only acetamide 7c led to a significantly lower QCO value at 120 °C (0.06 mmol g^−1^) than at 70 °C (0.24 mmol g^−1^), which can be explained by the fact that 7c decomposes at higher temperatures.

**Table 2:** Results of the wood modifications using activated neonicotinoids 7c–12c (WPG and QCO values are given as well as the associated standard deviations *σ* for experiments that were carried out several times)

Sample (reagent)	70 °C		120 °C	
	WPG_Ø_ ± *σ* [%]	QCO_Ø_ ± *σ* [mmol g^−1^]	WPG_Ø_ ± *σ* [%]	QCO_Ø_ ± *σ* [mmol g^−1^]
7d (**7c**)	7.2 ± 0.7	0.24 ± 0.02	1.9 ± 0.8	0.06 ± 0.03
8d (**8c**)	9.8 ± 1.7	0.26 ± 0.05	19.3 ± 1.3	0.52 ± 0.03
9d (**9c**)	4.3	0.10	9.4	0.21
10d (**10c**)	9.9 ± 0.1	0.21 ± 0.00	22.3 ± 2.9	0.47 ± 0.06
11d (**11c**)	14.2	0.29	19.3	0.40
12d (**12c**)	7.2	0.15	7.5	0.16

In addition to the imidacloprid substrates, the butadiene derivative 12c was also applied for wood modification and led to very similar QCO values of 0.15–0.16 mmol g^−1^ at both temperatures. Finally, imidacloprid (1) and insecticide 4 were bound successfully to the wood material.

### Analytic characterization of the modified wood

The actual formation of a covalent bond between the wood biopolymers and the applied reagent has already been confirmed by Drafz *via* 2-dimensional NMR spectroscopy, or by Söftje and Ehrhardt *via* thermal analyses (Pyrolysis GC MS).^7,39,40^ In addition, the penetration depth of the modifying reagent into the wood tissue has also been examined using μ-CT techniques.^39,41^ Furthermore, ATR-IR spectroscopy has proven as a very reliable and meaningful method in order to characterize modified wood, which is the reason why this non-destructive method has been chosen to examine all treated samples.^7,14–18,39,41^

**Fig. 1:**
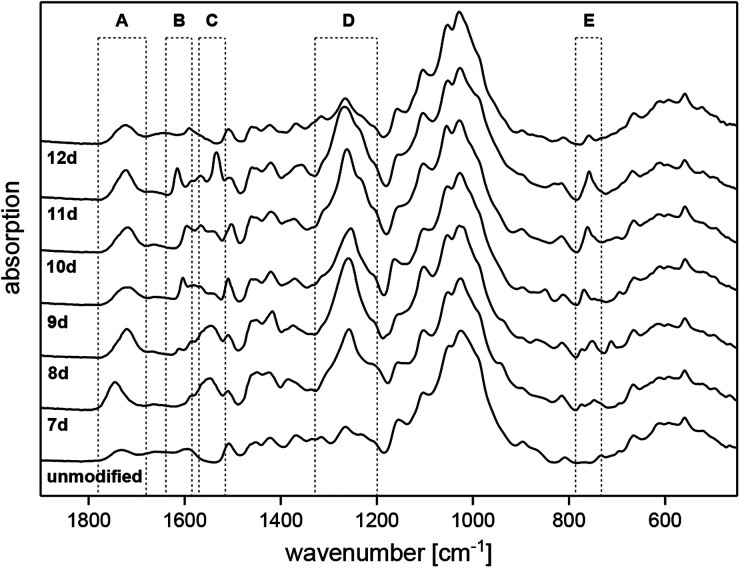
ATR-IR spectra of unmodified and modified wood in comparison (A: *ν*_C=O_, B: *ν*_C=C,arom._, C: *ν*_NO_2_,asym._, D: *ν*_C–O,asym._, E: *δ*_C–H,arom._).

Fig. 1 displays the ATR-IR spectra of untreated Scots pine sapwood in comparison to the chemically modified samples 7d–12d. The biopolymers of wood itself lead to characteristic vibration bands, which can be assigned to the structural components of the lignocellulose material according to literature.^42–47^ The spectral comparison of unmodified and modified wood shows significant changes caused by the formation of covalent bonds during the modification process. The formed ester functions are leading to an increase of the carbonyl stretching vibrations at approximately 1700 cm^−1^ (area A) as well as the corresponding C–O stretching bands at 1250 cm^−1^ (area D). Both bands unequivocally confirm the formation of a covalent bond between the wood polymers and the activated substrates 7c–12c. Furthermore, slightly enlarged aromatic C=C deformation vibrations at about 1600 cm^−1^ (area B) are visible for the modifications 8d–12d due to the introduced aromatic groups. The corresponding aromatic deformation vibrations were also detected at about 760 cm^−1^ (area E). The final proof of a successful modification is given by a specific band of the chemically introduced neonicotinoid. In detail, the asymmetric stretching vibration of the nitro moiety at 1550 cm^−1^ (area C) clearly confirms the attachment of the insecticidal group. In addition to the discussed changes in the ATR-IR spectra caused by the formed ester bonds of the bound insecticides, further intensity changes can be recognized in the spectra (see Fig. S1 in the ESI‡). In fact, a slightly visible decrease of the hydroxyl stretching vibrations at 3340 cm^−1^ for the samples 7d–12d in contrast to unmodified wood confirms the covalent esterification of the wood biopolymers.

### Biological tests of the modified wood

After the successful modification of the veneer chips with the previously synthesized neonicotinoids 7c–12c, we aimed to investigate the biological effectiveness of the functionalization. Therefore, the modification process had to be upscaled since European standardized efficacy tests require wood specimens measuring 15 × 25 × 50 mm^3^. Five of these blocks were modified simultaneously in a glass reactor whereby 150 mL of anhydrous DMF, triethylamine and DMAP were used. For the biological tests, activated benzamide 8c (1.0 mmol per gram wood) was applied as modification reagent. This derivative was chosen due to the stability of its aromatic ester group and the consistently good yields of the synthetic steps from 8a to 8c in order to obtain the activated reagent 8c in sufficient quantities for the following wood modification.

The small veneer chips were already completely penetrated by the modifying solution during the procedure described above. While the pinewood chips were functionalized in their entirety, it was necessary to achieve a sufficient penetration depth of the reagents for the larger pine wood blocks. Therefore, the reaction solution was prepared in a separate flask under nitrogen atmosphere before being transferred by means of a negative pressure of 0.5 bar into the glass reactor. The wood samples were swollen at this pressure for 2 h at 50 °C before increasing the pressure by nitrogen addition to atmospheric pressure. The samples reacted for 24 h at 70 °C like previously shown in Scheme 6. The modified samples 8d′ showed a bound amount of insecticide 8c (QCO) of 0.18 ± 0.01 mmol per gram wood on average (corresponds to a WPG value of 6.7 ± 0.3%).

The modified wood samples 8d′ were tested according to DIN EN 46-1 for their biological effectiveness against the larvae of the domestic house longhorn beetle (*Hylotrupes bajulus*).^48^ To compare the mortalities 5 extracted but unmodified wood samples and 3 completely untreated control samples were tested analogously to the 5 modified wood blocks.

**Fig. 2:**
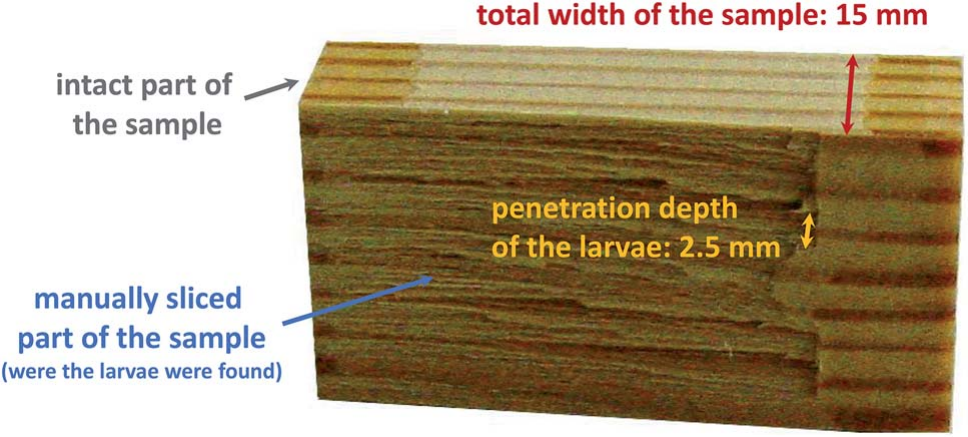
Final inspection of the modified test specimens 8d′ after 12 weeks: it is shown a wood block with its manually split surface were the dead larvae were found. The larvae have only penetrated the material to just below the surface up to 2.5 mm before they died. The rest of the sample remains intact and was not damaged by the larvae.

After 4 weeks of the test procedure, it was observed that 66.0% of the larvae had already died on the surface of the modified wood samples, while the remaining larvae had gnawed into the biomaterial. After a total of 12 weeks, the final examination of the wood samples was carried out by manually cutting up all test specimens. This procedure was used to find the larvae, which were then subjected to a vitality check. As shown in Fig. 2, the larvae did not penetrate very deep into the modified wood blocks (up to 2.5 mm) and were already located just below the surface. On the other hand, the unmodified samples had to be completely taken apart in order to find the deeply infiltrated larvae. At the end of the test, the mortality rate for the functionalized samples was 100.0% (Table 3), the extracted but unmodified samples averaged 10.9%, and the untreated control samples had a mortality of 4.8%.

**Table 3:** Results of the biological tests of the modified wood samples 8d′ (modified with 8c: WPG = 6.7 ± 0.2%, QCO = 0.18 ± 0.01 mmol) against the larvae of the domestic house longhorn beetle following DIN EN 46-1.^48^ The average mortality rates of the 5 and 3 wood blocks, respectively, as well as the associated standard deviations *σ* are given

Sample	Surface mortality rate (*Ø*) ± *σ* after 4 weeks [%]	Final mortality rate (*Ø*) ± *σ* [%]
Modified sample 8d′ (extracted, modified with 8c)	66.0 ± 28.8	100.0 ± 0.0
Reference sample I (extracted, unmodified)	2.0 ± 4.5	10.9 ± 10.6
Reference sample II (untreated reference)	0.0 ± 0.0	4.8 ± 8.2

According to the test standard DIN EN 46-1, untreated controls are allowed to have a natural mortality rate of up to 30%.^48^ Therefore, the test procedure was valid and gave a very good efficacy of the covalent modification of pine wood using benzamide 8c against the larvae of the domestic longhorn beetle.

## Experimental

### Preparation of the organic precursors

General experimental information and synthetic procedures are described in the ESI.‡^1^H NMR, ^13^C NMR and ^15^C NMR spectra are available as well.

### General procedure for chemical modification of wood chips following Kaldun *et al.*^17,18^

In general, the common amount of 7 mmol of the appropriate wood modifying reagent was applied per 1.00 g of veneer chips of Scots pine sapwood (approx. 10 × 10 × 0.7 mm^3^, approx. 0.04–0.07 g). Each reaction was carried out in the Heidolph “Synthesis 1” parallel synthesizer in 6 mL of anhydrous DMF with 2 equivalents of distilled triethylamine and 10 mol% of DMAP under nitrogen atmosphere. Prior to this wood modification, the wood was extracted for 24 h in a Soxhlet apparatus with a solvent mixture comprising toluene/acetone/methanol in a 4 : 1 : 1 ratio. Subsequently, the pretreated wood was oven-dried at 105 °C for 24 h and then stored under vacuum in a laboratory desiccator. The wood modification reaction started with heating the wood sample with triethylamine and DMAP in dry DMF at 50 °C for 2 h. Subsequently, the modification reagent was added and afterwards the temperature was increased to 70 °C or 120 °C for 24 h. After this reaction time, the mixture was allowed to cool down to rt. The thus modified wood was washed successively with THF (50 mL), chloroform (50 mL), and diethyl ether (50 mL). Afterwards, the wood sample was extracted again for 24 h applying the Soxhlet apparatus with the same solvent mixture as stated above. Finally, the modified wood was dried at 105 °C for 24 h and finally weighed.

### General procedure for chemical modification of DIN EN 46-1 wood blocks^48^

An amount of 1 mmol of the wood modifying reagent was applied per 1.00 g of blocks of Scots pine sapwood (approx. 15 × 25 × 50 mm^3^, approx. 9.00–10.00 g). The reaction was carried out in a special glas reactor which was closed with a flat flange. 5 blocks were modified simultaneously in the same vessel whereby 150 mL of anhydrous DMF, 2 equivalents of distilled triethylamine and 10 mol% of DMAP were used under nitrogen atmosphere. Prior to this wood modification, the wood blocks were extracted for 3 d in a Soxhlet apparatus with a solvent mixture comprising toluene/acetone/methanol in a 4 : 1 : 1 ratio. Subsequently, the pretreated wood was oven-dried at 105 °C for 3 d and then stored under vacuum in a laboratory desiccator. The modification started with the evacuation of the wood samples in the glass reactor using a rotary vane pump. Afterwards, a vacuum of 0.5 bar was applied using a diaphragm pump. The reaction solution consisting of *N*-(1-{[4-(1*H*-benzotriazole-1-carbonyl)phenyl]methyl}-3-[(6-chloropyridin-3-yl)methyl]imidazolidin-2-ylidene)nitramide (8c, 1 mmol g^−1^ wood), triethylamine and DMAP in dry DMF was prepared during this time in a separate flask under nitrogen atmosphere. The solution was then transferred *via* a metal cannula through a septum into the glass reactor with the aid of the above mentioned negative pressure. The wood samples were heated at 50 °C for 2 h at 0.5 bar. Subsequently, the pressure was increased by means of nitrogen supply to atmospheric pressure. 30 mL dry DMF were added and the reaction mixture was stirred for 24 h at 70 °C. After this reaction time, the mixture was allowed to cool down to rt. The thus modified wood was washed successively with THF (250 mL), chloroform (250 mL), and diethyl ether (250 mL). Afterwards, the wood samples were extracted again for 3 d applying the Soxhlet apparatus with the same solvent mixture as stated above. Finally, the modified wood was dried at 105 °C for 3 d and finally weighed.

### Procedure of the biological test of the wood samples according to the DIN EN 46-1 standard^48^

In order to compare the mortality rates five modified wood blocks 8d′, five extracted but unmodified wood samples (reference sample I) and three completely untreated control samples (reference sample II) were tested analogously. First, a glass pane was glued to one side of each wood block followed by the positioning of ten egg larvae of the domestic house longhorn beetle into the gap between the wood surface and the glass pane. After a rest period of four weeks, the test specimens were subjected to an optical check in order to count the larvae which died on the surface and which had gnawed into each sample. Following another rest time of eight weeks (12 weeks since the start of the test), the wood samples were finally examined by manually splitting the test specimens. This procedure was used to find and count all the larvae, which had gnawed into the samples followed by subjecting the found larvae to a vitality test. Based on the balance of the living and dead larvae, mortality rates were calculated both for the surface mortality (after four weeks) and for the whole sample (after 12 weeks).

## Conclusion

In the course of this work, a number of novel activated neonicotinoids 7c–12c, based on an imidacloprid moiety, were synthesized and consequently used for covalent wood modification. Particularly noteworthy is the benzamide 8c, which was attached to pinewood with considerable amounts of up to 0.52 mmol per gram wood. All modified wood samples were spectroscopically analyzed. The attached fragments were detected by ATR-IR spectroscopy, which proved the formation of a covalent bond between the insecticide and the wood cell surface. According to the standard DIN EN 46-1, the specimen functionalized with imidacloprid benzamide 8c showed an extraordinary biological activity against the larvae of the domestic house longhorn beetle (*Hylotrupes bajulus*). Finally, the successfully passed DIN-EN 46-1 test proved a mortality of the larvae of 100% and thereby underlines the effectiveness of this covalent modification against insects. In addition, this newly developed wood protection process is not only characterized by its biological effectiveness but also by its high persistency. The covalent fixation prevents leaching of the insecticide, which also leads to a good environmental compatibility of this unique wood protection method. The covalent fixation ought to be applicable to other types of insecticides or fungicides, too.

## Conflicts of interest

There are no conflicts to declare.

## Supplementary Material

RA-010-D0RA02071K-s001
