# Supplementary material for: Novel nicotinoid structures for covalent modification of wood: an environmentally friendly way for its protection against insects
Source: RSC Adv. 2020 Apr 21;10(27):15726–33. doi: 10.1039/d0ra02071k (PMC9052505; doi:10.1039/d0ra02071k)
Supplement: RA-010-D0RA02071K-s001 [file RA-010-D0RA02071K-s001.pdf]

Electronic Supplementary Material (ESI) for RSC Advances.
This journal is © The Royal Society of Chemistry 2020

Supporting Information

Novel nicotinoid structures for covalent modification of wood: an environmentally friendly way for its protection against insects†

Martin Söftje,[a] Sophie Acker,[a] Rudy Plarre,[b] Jan C. Namyslo[a] and Dieter E. Kaufmann*[a]

[a] Dr. M. Söftje, S. Acker, Dr. J. C. Namyslo, Prof. Dr. D. E. Kaufmann
Institute of Organic Chemistry
Clausthal University of Technology
Leibnizstraße 6, 38678 Clausthal-Zellerfeld, Germany,
E-Mail: dieter.kaufmann@tu-clausthal.de

[b] Dr. R. Plarre
Bundesanstalt für Materialforschung und -prüfung
Unter den Eichen 87, 12205 Berlin, Germany

† Chemical improvement of surfaces, Part 7.

Table of Contents

1. General experimental information 3

2. Synthesis of the neonicotinoids 4

3. Wood modifications 26

4. Biological tests 28

5. NMR spectra 29

6. Literature 74

1. General experimental information

Thin layer chromatography (TLC) was performed on Merck TLC plates (aluminum based) silica gel 60 F 254. Purifications were carried out by using column chromatography on silica gel 60 (Merck). Petroleum ether as eluent had the boiling range 60 – 70 °C. Melting points were determined with a Differential Scanning Calorimeter Perkin-Elmer DSC6 or with a Stuart SMP3 apparatus. ^{1}H NMR (600 MHz), ^{13}C NMR (150 MHz), ^{15}N NMR (61 MHz): Avance III 600 MHz FT-NMR spectrometer (Bruker, Rheinstetten, Germany). ^{1}H NMR (400 MHz), ^{13}C NMR (100 MHz): Avance 400 FT-NMR spectrometer (Bruker). ^{1}H and ^{13}C NMR spectra were referenced to the residual solvent peak [$CDCl_3$: δ = 7.26 ppm (^{1}H), δ = 77.0 ppm (^{13}C) or DMSO-d_6: δ = 2.50 ppm (^{1}H), δ = 39.5 ppm (^{13}C)]. For the ^{15}N NMR spectra nitromethane (δ = 0.0 ppm) was used as an internal or external standard. In all cases, peak assignments were accomplished by DEPT135-, HSQC- and HMBC-NMR experiments. The coupling constants *J* are given in Hertz (Hz). For the determination of the multiplicities the following abbreviations apply: s = singlet, bs = broad singlet, d = doublet, dd = doublet of a doublet, ddd = doublet of a doublet of a doublet, t = triplet, m = multiplet. Primary and tertiary carbon atoms have been marked with a "+", secondary with a "-" and quaternary with an "o", according to the peak orientation in the DEPT135 spectra. EI mass spectra were obtained on a Varian 320 MS Triple Quad GC/MS/MS instrument with a Varian 450-GC usually in direct mode (DEP method) using electron impact ionization (70 eV). In the case of chlorinated and brominated compounds, all peak values of molecular ions as well as fragments refer to the isotope ^{35}Cl and ^{79}Br, respectively. ESI mass spectra were measured on a LC-MSD Series 1100 (Agilent/Hewlett Packard). The elemental composition was confirmed by high resolution ESI mass spectrometry, performed on a Waters Acquity UPLC, coupled to a Waters Q TOF Premier, while high resolution EI mass spectrometry was performed on a Waters Micromass GCT in direct mode. All HRMS results were satisfactory relative to the calculated accurate mass of the molecular ion (±2.3 ppm, $R \approx 10000$). FTIR spectra were obtained with a Bruker Alpha-T FT-IR in the range of 400 to 4000 cm^{-1}. Liquids and solids were measured via a platinum ATR unit. IR spectra of the modified wood samples were obtained with a Bruker Tensor II also equipped with a platinum ATR unit, measured under maximum contact pressure. Wood modification reactor: Heidolph “Synthesis 1” Liquid System Parallel Synthesizer (Heidolph, Schwabach, Germany). Tetrahydrofurane (THF) and dichloromethane (DCM) were dried using an MP5 Solvent Purification System from Inert Technology (Amesbury, MA, USA). Dry *N,N*-dimethylformamide (DMF) and all other chemicals were used as purchased from Acros GmbH & Co. KG (Karlsruhe, Germany), Sigma-Aldrich Chemie GmbH (Taufkirchen, Germany) or Merck KGaA (Darmstadt, Germany). Wood samples were obtained from the Section of Wood Biology and Wood Products, Georg-August-University Göttingen (Göttingen, Germany) and from the Bundesanstalt für Materialforschung und -prüfung (BAM, Berlin, Germany). The biological investigations were carried out at the Bundesanstalt für Materialforschung und -prüfung (BAM, Berlin, Germany) according to DIN EN 46-1.[1]

2. Synthesis of the neonicotinoids

General procedure A for nucleophilic substitutions following KAGABU:[2] 1.00 – 2.00 eq. of sodium hydride (60% dispersion in paraffin oil) were added to a solution of the alcohol or the secondary amine in dry DMF under nitrogen atmosphere and ice cooling. The suspension was stirred for 15 min – 1 h at 0 °C and for a further 1 – 2 h at room temperature (rt). Subsequently, 1.10 – 4.00 eq. of the electrophilic component were added quickly under ice cooling. The mixture was stirred for 3 h – 3 d at the indicated temperature before adding water or a saturated aqueous (aq.) solution of ammonium chloride. **Variant 1:** The mixture was extracted with DCM and the combined organic phases were washed with water and dried over magnesium sulfate. After evaporation of the solvent and adsorption on silica gel, the product was purified by column chromatography and dried *in vacuo*. **Variant 2:** The precipitate was collected by filtration, washed with water, possibly petroleum ether and dried *in vacuo*. **Variant 3:** The mixture was extracted with DCM and the combined organic phases were washed with water and dried over magnesium sulfate. The solution was concentrated, the residue was dissolved in a small amount of cold methanol and cooled in an ice bath. Subsequently, the precipitated solid was suspended in petroleum ether, collected by filtration, washed with cold ethanol and dried *in vacuo*.

General procedure B for acidic ester hydrolyses following MANICARDI:[3,4] 7.50 – 30.00 eq. of trifluoroacetic acid were added to a solution of the ester compound in DCM or chloroform under ice cooling. The mixture was stirred for 1 – 4 d at rt. If necessary, another 7.50 – 15.00 eq. of trifluoroacetic acid were added and the mixture was stirred for a further 7 h – 3 d at the indicated temperature. **Variant 1:** Methanol and chloroform were added and the solvent was removed *in vacuo*. The process was repeated until a solid was obtained. The resulting product was dried *in vacuo*. **Variant 2:** Methanol and chloroform were added and the solution was concentrated *in vacuo*. The precipitate was collected by filtration, washed with water and dried *in vacuo*. **Variant 3:** Water was added and the resulting mixture was washed with chloroform. The aq. phase was concentrated to dryness and the product was dried *in vacuo*.

General procedure C for the activation of the carbonyl group with 1*H*-benzotriazole following NAMYSLO:[5–8] 1.10 – 1.68 eq. of thionyl chloride were added to a suspension of the carboxylic acid (or the sodium carboxylate) and 2.20 – 3.52 eq. 1*H*-benzotriazole in dry DCM. The mixture was stirred for 16 – 30 h at rt. Subsequently, water respectively a saturated solution of ammonium chloride (aq.) was added. **Variant 1:** The mixture was extracted with DCM and the combined organic phases were washed with water and dried over magnesium sulfate. After evaporation of the solvent and adsorption on silica gel, the product was purified by column chromatography and dried *in vacuo*. **Variant 2:** The mixture was extracted with DCM and the combined organic phases were washed with water and dried over magnesium sulfate. After evaporation of the solvent the product was dried *in vacuo*. **Variant 3:** The precipitate was collected by filtration, washed with water and DCM. The product was dried *in vacuo*. **Variant 4:** The mixture was extracted with DCM and the combined organic phases were concentrated. The precipitated solid was suspended in petroleum ether, collected by filtration, washed with a 2 M solution of hydrochloric acid (aq.) and water. The product was dried *in vacuo*.

N-Imidazolidin-2-ylidenenitramide (3)[9,10]

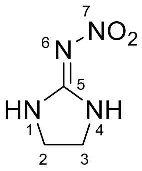

Compound **3** was prepared according to literature.[9,10] Ethane-1,2-diamine (19.050 g, 21.17 mL, 317.00 mmol, 1.00 eq.) was added to a solution of nitroguanidine (**2**) (33.000 g, 317.00 mmol, 1.00 eq.) in water (190 mL) and conc. hydrochlorid acid (25 mL) over a period of 15 min. The mixture was stirred for 8 h at 60 °C and afterwards cooled to 0 °C. The precipitate was collected by filtration, washed with water and dried *in vacuo*. The product was isolated as a beige solid, yield: 25.216 g (194.00 mmol, 61%). m.p. (DSC): 179 °C. ^{1}H NMR (400 MHz, DMSO-d_6): δ = 8.40 (bs, 2 H, NH-1, NH-4), 3.57 (s, 4 H, H-2, H-3) ppm. ^{13}C NMR (100 MHz, DMSO-d_6): δ = 163.1 (o, 1 C, C-5), 41.8 (-, 2 C, C-2, C-3) ppm. IR (ATR): $\tilde{\nu}$ = 3419, 3195, 3120, 2987, 2932, 2893, 1608, 1560, 1486, 1466, 1449, 1377, 1309, 1281, 1206, 1188, 1113, 1036, 1017, 973, 930, 834, 782, 748, 695, 642, 574, 504, 464, 433, 411 cm^{-1}. MS (EI, DEP, 70 eV): *m/z* (%) = 129.9 (100) [M$^+$], 99.8 (25) [M-C_2H_4-H_2]$^+$.

***N*^{1}-[(6-Chloropyridin-3-yl)methyl]ethane-1,2-diamine (6)[11]**

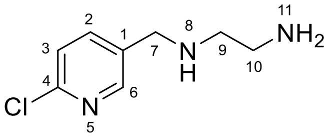

Compound **6** was prepared according to literature.[11] A sodium hydroxide solution (50% aq., 1.190 g, 0.78 mL, 14.87 mmol, 1.20 eq.) was added to a solution of ethane-1,2-diamine (3.720 g, 4.14 mL, 62.00 mmol, 5.00 eq.) in acetonitrile (20 mL). A solution of 2-chloro-5-(chloromethyl)pyridine (2.008 g, 12.39 mmol, 1.00 eq.) in acetonitrile (20 mL) was added under ice cooling. The mixture was stirred for 24 h at rt. Subsequently, the solvent was removed *under vacuo*, the residue was dissolved in an aq. sodium hydroxide solution (10%, 20 mL) and extracted with DCM (3 × 30 mL). The combined organic phases were dried over magnesium sulfate and concentrated. The product was dried *in vacuo* and isolated as a yellow oil, yield: 2.267 g (12.21 mmol, 99%). ^{1}H NMR (400 MHz, $CDCl_3$): δ = 8.31 (d, $J_{H,H}$ = 2.3 Hz, 1 H, H-6), 7.65 (dd, $J_{H,H}$ = 8.1, 2.3 Hz, 1 H, H-2), 7.26 (d, $J_{H,H}$ = 8.1 Hz, 1 H, H-3), 3.78 (s, 2 H, H-7), 2.80 (t, $J_{H,H}$ = 5.7 Hz, 2 H, H-10), 2.66 (t, $J_{H,H}$ = 5.7 Hz, 2 H, H-9), 1.61 (bs, 3 H, NH-8, NH-11) ppm. ^{13}C NMR (100 MHz, $CDCl_3$): δ = 150.0 (o, 1 C, C-4), 149.3 (+, 1 C, C-6), 138.7 (+, 1 C, C-2), 134.8 (o, 1 C, C-1), 124.0 (+, 1 C, C-3), 51.7 (-, 1 C, C-9), 50.3 (-, 1 C, C-7), 41.5 (-, 1 C, C-10) ppm. IR (ATR): $\tilde{\nu}$ = 3361, 3288, 2928, 2825, 1664, 1585, 1566, 1539, 1455, 1385, 1359, 1330, 1286, 1134, 1099, 1023, 922, 814, 730, 678, 657, 633, 570, 537, 498, 446, 413 cm^{-1}. MS (ESI, +): *m/z* (%) = 186.1 (100) [M+H]$^+$.

N-{(2E)-1-[(6-Chloropyridin-3-yl)methyl]imidazolidin-2-ylidene}nitramide (Imidacloprid, 1)[12]

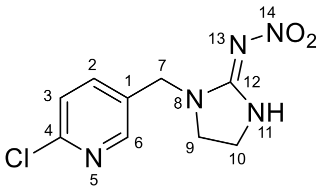

Compound **1** was prepared according to literature.[12] A solution of 2-chloro-5-(chloromethyl)pyridine (2.000 g, 11.73 mmol, 1.00 eq.) in acetonitrile (20 mL) was added to a suspension of *N*-imidazolidin-2-ylidenenitramid (**3**) (1.755 g, 13.49 mmol, 1.15 eq.) and potassium carbonate (2.760 g, 19.94 mmol, 1.70 eq.) in acetonitrile (20 mL). The mixture was refluxed for 6 h, stirred overnight at rt and refluxed for another 6 h before being filtered and washed with DCM (10 mL). The residue was adsorbed on silica gel and purified by column chromatography (ethyl acetate). The product was dried *in vacuo* and isolated as a colorless solid, yield: 1.701 g (6.65 mmol, 57%). m.p. (DSC): 143 °C. ^{1}H NMR (600 MHz, DMSO-d_6): δ = 8.97 (s, 1 H, NH-11), 8.36 (dd, $J_{H,H}$ = 2.5, 0.6 Hz, 1 H, H-6), 7.78 (dd, $J_{H,H}$ = 8.2, 2.5 Hz, 1 H, H-2), 7.50 (dd, $J_{H,H}$ = 8.2, 0.6 Hz, 1 H, H-3), 4.48 (s, 2 H, H-7), 3.66 – 3.63 (m, 2 H, H-10), 3.51 – 3.48 (m, 2 H, H-9) ppm. ^{13}C NMR (151 MHz, DMSO-d_6): δ = 160.3 (o, 1 C, C-12), 149.5 (o, 1 C, C-4), 149.3 (+, 1 C, C-6), 139.3 (+, 1 C, C-2), 131.5 (o, 1 C, C-1), 124.3 (+, 1 C, C-3), 45.1 (-, 1 C, C-9), 44.5 (-, 1 C, C-7), 41.6 (-, 1 C, C-10) ppm. ^{15}N NMR (61 MHz, DMSO-d_6): δ = -12.8 (1 N, N-14), -72.8 (1 N, N-5), -145.5 (1 N, N-13), -288.9 (1 N, N-11), -292.0 (1 N, N-8) ppm. IR (ATR): $\tilde{\nu}$ = 3342, 1558, 1478, 1456, 1435, 1389, 1359, 1296, 1274, 1222, 1201, 1139, 1128, 1100, 1049, 1024, 993, 975, 960, 938, 918, 844, 825, 811, 784, 750, 741, 712, 675, 631, 614, 511, 477, 453, 411, 406 cm^{-1}. MS (ESI, +): *m/z* (%) = 278.0 (100) [M+Na]$^{+}$.

2-Chloro-5-{[(2E)-2-(2,3,3-trichloro-1-nitroprop-2-en-1-ylidene)imidazolidin-1-yl]methyl}pyridine (4)[13–16]

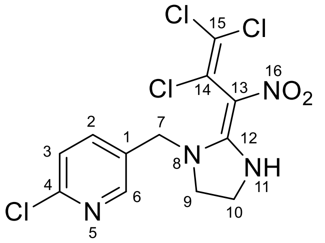

Compound **4** was prepared according to literature.[13–16] A solution of 1,1,2,4,4-pentachloro-3-nitrobuta-1,3-diene (3.364 g, 12.40 mmol, 1.00 eq.) in methanol (20 mL) was added dropwise to a solution of N^1-[(6-chloropyridin-3-yl)methyl]ethane-1,2-diamine (**6**) (2.302 g, 12.40 mmol, 1.00 eq.) and potassium carbonate (2.056 g, 14.88 mmol, 1.20 eq.) in methanol (40 mL) at -40 °C. The mixture was stirred at -40 °C for 2 h and another 20 h at rt before adding water (50 mL). The precipitate was collected by filtration, washed with water and petroleum ether and dried *in vacuo*. The product was isolated as a yellow solid, yield: 4.301 g (11.20 mmol, 90%). m.p. (DSC): 178 °C (decomp.). ^{1}H NMR (400 MHz, DMSO-d_6): δ = 9.40 (s, 1 H, NH-11), 8.35 (dd, $J_{H,H}$ = 2.6, 0.6 Hz, 1 H, H-6), 7.77 (dd, $J_{H,H}$ = 8.3, 2.6 Hz, 1 H, H-2), 7.55 (dd, $J_{H,H}$ = 8.3, 0.6 Hz, 1 H, H-3), 4.55 (s, 2 H, H-7), 3.81 – 3.72 (m, 4 H, H-9, H-10) ppm. ^{13}C NMR (100 MHz, DMSO-d_6): δ = 159.6 (o, 1 C, C-12), 149.4 (o, 1 C, C-4), 147.7 (+, 1 C, C-6), 137.7 (+, 1 C, C-2), 131.3 (o, 1 C, C-1), 125.4 (o, 1 C, C-15), 125.0 (o, 1 C, C-14), 124.2 (+, 1 C,

C-3), 103.1 (o, 1 C, C-13), 50.7 (-, 1 C, C-9), 49.2 (-, 1 C, C-7), 42.5 (-, 1 C, C-10) ppm. IR (ATR): $\tilde{\nu}$ = 3296, 3058, 1593, 1564, 1520, 1483, 1460, 1439, 1394, 1297, 1247, 1191, 1130, 1101, 1042, 1021, 998, 952, 931, 904, 832, 794, 763, 730, 707, 667, 631, 496, 454, 431, 414 cm^{-1}. MS (EI, DEP, 70 eV): *m/z* (%) = 384.4 (1) $[M^+]$, 346.9 (3) $[M\text{-}Cl]^+$, 282.1 (8), 237.2 (9), 207.3 (8) $[M\text{-}C_2Cl_3\text{-}NO_2]^+$, 126.1 (100) $[C_6H_5ClN]^+$, 99.2 (18).

***tert*-Butyl 4-methylbenzoate (14)[17]**

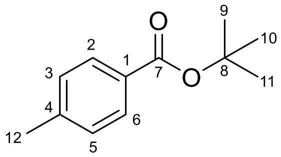

Compound **14** was prepared according to literature.[17] Dry *tert*-butanol (23.720 g, 30.00 mL, 320.00 mmol, 16.00 eq.) was added to a solution of lithium *tert*-butoxide (1.761 g, 22.00 mmol, 1.10 eq.) in dry THF (30 mL) under nitrogen atmosphere. The mixture was stirred for 30 min, followed by addition of 4-methylbenzoyl chloride (**13**) (3.090 g, 2.64 mL, 20.00 mmol). The solution was refluxed for 6 h and stirred overnight at rt. Subsequently, the solvent was removed *in vacuo* and the residue dissolved in ethyl acetate. The organic phase was washed with water (2 x 20 mL), dried over magnesium sulfate and concentrated. The product was dried *in vacuo* and isolated as a yellow oil, yield: 3.393 g (17.65 mmol, 88%). ^1H NMR (400 MHz, $CDCl_3$): δ = 7.88 (d, $J_{H,H}$ = 8.2 Hz, 2 H, H-2, H-6), 7.21 (d, $J_{H,H}$ = 8.2 Hz, 2 H, H-3, H-5), 2.40 (s, 3 H, H-12), 1.59 (s, 9 H, H-9, H-10, H-11) ppm. ^{13}C NMR (100 MHz, $CDCl_3$): δ = 165.9 (o, 1 C, C-7), 142.9 (o, 1 C, C-4), 129.4 (+, 2 C, C-2, C-6), 129.3 (o, 1 C, C-1), 128.9 (+, 2 C, C-3, C-5), 80.7 (o, 1 C, C-8), 28.2 (+, 3 C, C-9, C-10, C-11), 21.6 (+, 1 C, C-12) ppm. IR (ATR): $\tilde{\nu}$ = 2977, 2930, 1708, 1611, 1578, 1508, 1475, 1457, 1408, 1392, 1367, 1310, 1286, 1255, 1185, 1163, 1106, 1037, 1020, 958, 867, 850, 791, 753, 691, 639, 612, 503, 480, 457 cm^{-1}. MS (EI, DEP, 70 eV): *m/z* (%) = 192.4 (100) $[M^+]$, 118.9 (35) $[M\text{-}C_4H_9O]^+$.

***tert*-Butyl 4-(bromomethyl)benzoate (8)[17]**

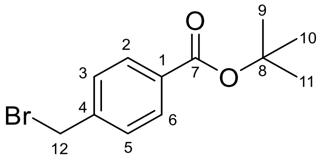

Compound **8** was prepared according to literature.[24] Azobis(isobutyronitrile) (0.030 g, 0.19 mmol, 0.01 eq.) was added to a solution of *tert*-butyl 4-methylbenzoate (**14**) (3.390 g, 17.65 mmol) and *N*-bromosuccinimide (3.460 g, 19.42 mmol, 1.10 eq.) in dry DCM (50 mL). The solution was refluxed for 6 h. Water (15 mL) was added and the mixture extracted with DCM (2 x 30 mL). The combined organic phases were dried over magnesium sulfate and concentrated. The residue was adsorbed on silica gel and purified by column chromatography (petroleum ether:ethyl acetate = 10:1). The product was dried *in vacuo* and isolated as a colorless solid, yield: 4.010 g (14.79 mmol, 84%), m.p. (DSC): 53 °C. ^1H NMR (400 MHz, $CDCl_3$): δ = 7.96 (d, $J_{H,H}$ = 8.3 Hz, 2 H, H-2, H-6), 7.43 (d, $J_{H,H}$ = 8.3 Hz, 2 H, H-3, H-5), 4.49 (s, 2 H, H-12), 1.59 (s, 9 H, H-9, H-10, H-11) ppm. ^{13}C NMR (100 MHz, $CDCl_3$): δ = 165.2 (o, 1 C, C-7), 142.1 (o, 1 C, C-4), 132.0 (o, 1 C, C-1), 129.9 (+, 2 C, C-2, C-6), 128.9 (+, 2 C, C-3, C-5), 81.2 (o, 1 C, C-8), 32.4 (-, 1 C, C-12), 28.2 (+, 3 C, C-9, C-10, C-11) ppm. IR (ATR): $\tilde{\nu}$ = 3056, 3041, 3005, 2980, 2931, 1694, 1610, 1576, 1509, 1475, 1456, 1416, 1391, 1369, 1363, 1315, 1292, 1254, 1203, 1187, 1158, 1116, 1018, 971, 866, 846, 822, 804, 773, 750,

704, 637, 625, 603, 500, 488, 464, 448, 435 cm^{-1}. MS (EI, DEP, 70 eV): *m/z* (%) = 269.9 (2) $[M^+]$, 214.8 (66) $[M+H-C_4H_9]^+$, 196.7 (29) $[M-C_4H_9O]^+$, 190.5 (14) $[M-Br]^+$, 168.1 (40) $[M-C_5H_9O_2]^+$, 159.8 (30), 135.1 (100) $[M+H-Br-C_4H_9]^+$, 118.2 (34) $[M-Br-C_4H_9O]^+$, 98.0 (55), 90.4 (29) $[M-Br-C_5H_9O_2]^+$.

tert-Butyl 4-hydroxybenzoate (18)[18]

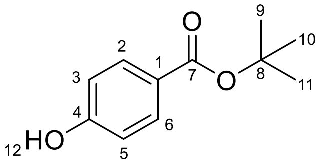

Compound **18** was prepared according to literature.[18] Dry *tert*-butanol (79.000 g, 100.00 mL, 1066.00 mmol, 36.80 eq.) was added to a solution of 4-hydroxybenzoic acid (**15**) (4.000 g, 29.00 mmol) and 4-dimethylaminopyridine (DMAP) (0.177 g, 1.45 mmol, 0.05 eq.) in dry THF (100 mL) under nitrogen atmosphere. A solution of *N*,*N'*-dicyclohexylcarbodiimine (DCC) (5.980 g, 29.00 mmol, 1.00 eq.) in dry THF (50 mL) was added dropwise. The mixture was stirred for 20 h at rt, filtered, and the solvent removed *under vacuo*. The residue was dissolved in DCM (50 mL) and washed with an aq. 0.3 molar solution of sodium carbonate (50 mL). The aq. phase was extracted with DCM (2 x 30 mL), the combined organic phases were dried over magnesium sulfate and concentrated. The crude product was adsorbed on silica gel and purified by column chromatography (petroleum ether:ethyl acetate = 5:1). The product was dried *in vacuo* and isolated as a colorless solid, yield: 4.178 g (21.51 mmol, 74%). m.p. (DSC): 137 °C. ^1H NMR (400 MHz, $CDCl_3$): δ = 7.89 (d, $J_{H,H}$ = 8.8 Hz, 2 H, H-2, H-6), 6.86 (d, $J_{H,H}$ = 8.8 Hz, 2 H, H-3, H-5), 6.43 (bs, 1 H, OH-12), 1.59 (s, 9 H, H-9, H-10, H-11) ppm. ^{13}C NMR (100 MHz, $CDCl_3$): δ = 166.2 (o, 1 C, C-7), 159.9 (o, 1 C, C-4), 131.7 (+, 2 C, C-2, C-6), 124.1 (o, 1 C, C-1), 115.1 (+, 2 C, C-3, C-5), 81.0 (o, 1 C, C-8), 28.2 (+, 3 C, C-9, C-10, C-11) ppm. IR (ATR): $\tilde{\nu}$ = 3284, 3000, 2980, 2971, 2934, 1674, 1606, 1589, 1515, 1443, 1393, 1368, 1318, 1298, 1281, 1253, 1227, 1178, 1154, 1130, 1102, 1039, 1008, 956, 849, 775, 754, 700, 639, 618, 520, 499, 467, 455, 439 cm^{-1}. MS (EI, DEP, 70 eV): *m/z* (%) = 194.2 (6) $[M^+]$, 173.1 (19), 138.1 (100) $[M+H-C_4H_9]^+$, 121.1 (82) $[M-C_4H_9O]^+$, 109.1 (12), 100.1 (24).

tert-Butyl 3-chloro-4-hydroxybenzoate (19)[19]

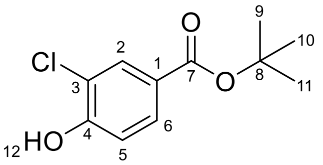

Compound **19** was prepared according to literature.[19] Dry *tert*-butanol (22.520 g, 28.50 mL, 304.00 mmol, 36.90 eq.) was added to a solution of 3-chloro-4-hydroxybenzoic acid hemihydrate (**16**) (1.500 g, equates to pure 3-chloro-4-hydoxybenzoic acid: 1.422 g, 8.24 mmol, 1.00 eq.) and 4-dimethylaminopyridine (0.064 g, 0.52 mmol, 0.06 eq.) in dry THF (30 mL) under nitrogen atmosphere. Dry magnesium sulfate was added to bind the crystal water of the hemihydrate. A solution of DCC (2.870 g, 13.91 mmol, 1.69 eq.) in dry THF (20 mL) was added dropwise. The mixture was stirred for 20 h at rt, filtered and dried over magnesium sulfate. The crude product was adsorbed on silica gel and purified by column chromatography (petroleum ether:ethyl acetate = 8:1). The product was dried *in vacuo* and isolated as a colorless solid, yield: 1.328 g (5.81 mmol, 71%). m.p. (DSC): 109 °C. ^1H NMR

(400 MHz, $CDCl_3$): δ = 7.97 (d, $J_{H,H}$ = 2.0 Hz, 1 H, H-2), 7.83 (dd, $J_{H,H}$ = 8.5, 2.0 Hz, 1 H, H-6), 7.03 (d, $J_{H,H}$ = 8.5 Hz, 1 H, H-5), 5.92 (bs, 1 H, OH-12), 1.58 (s, 9 H, H-9, H-10, H-11) ppm. ^{13}C NMR (100 MHz, $CDCl_3$): δ = 164.4 (o, 1 C, C-7), 154.8 (o, 1 C, C-4), 130.6 (+, 1 C, C-2), 130.1 (+, 1 C, C-6), 125.6 (o, 1 C, C-1), 119.7 (o, 1 C, C-3), 115.7 (+, 1 C, C-5), 81.3 (o, 1 C, C-8), 28.2 (+, 3 C, C-9, C-10, C-11) ppm. IR (ATR): $\tilde{\nu}$ = 3201, 3174, 3006, 2979, 2971, 2932, 1687, 1669, 1599, 1578, 1519, 1477, 1455, 1409, 1393, 1366, 1322, 1299, 1275, 1252, 1217, 1170, 1152, 1120, 1054, 895, 850, 826, 761, 716, 697, 635, 546, 496, 463, 450, 440, 409 cm^{-1}. MS (EI, DEP, 70 eV): *m/z* (%) = 227.7 (16) [M^+], 171.9 (100), [$M+H-C_4H_9$]$^+$, 154.3 (37) [$M-C_4H_9O$]$^+$, 110.4 (39), 96.8 (32).

***tert*-Butyl 4-hydroxy-3-nitrobenzoate (20)[20,21]**

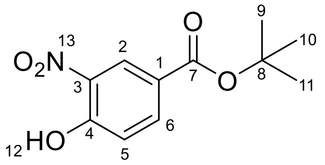

Compound **20** was prepared according to literature.[20,21] Dry *tert*-butanol (15.58 g, 19.73 mL, 210.00 mmol, 35.00 eq.) was added to a solution of 4-hydroxy-3-nitrobenzoic acid (**17**) (1.100 g, 6.01 mmol, 1.00 eq.) and DMAP (0.044 g, 0.36 mmol, 0.06 eq.) in dry THF (30 mL) under nitrogen atmosphere. A solution of DCC (1.301 g, 6.31 mmol, 1.05 eq.) in dry THF (20 mL) was added dropwise. The mixture was stirred for 20 h at rt, filtered and dried over magnesium sulfate. The crude product was adsorbed on silica gel and purified by column chromatography (petroleum ether:ethyl acetate = 8:1). The product was dried *in vacuo* and isolated as a yellow solid, yield: 0.284 g (1.19 mmol, 20%). m.p. (DSC): 130 °C. ^{1}H NMR (400 MHz, $CDCl_3$): δ = 10.8 (s, 1 H, OH-12), 8.73 (d, $J_{H,H}$ = 2.1 Hz, 1 H, H-2), 8.17 (dd, $J_{H,H}$ = 8.8, 2.1 Hz, 1 H, H-6), 7.18 (d, $J_{H,H}$ = 8.8 Hz, 1 H, H-5), 1.60 (s, 9 H, H-9, H-10, H-11) ppm. ^{13}C NMR (100 MHz, $CDCl_3$): δ = 163.3 (o, 1 C, C-7), 157.7 (o, 1 C, C-4), 137.9 (+, 1 C, C-6), 133.1 (o, 1 C, C-3), 127.0 (+, 1 C, C-2), 124.6 (o, 1 C, C-1), 119.9 (+, 1 C, C-5), 82.2 (o, 1 C, C-8), 28.1 (+, 3 C, C-9, C-10, C-11) ppm. IR (ATR): $\tilde{\nu}$ = 3274, 3099, 2981, 2941, 1714, 1622, 1576, 1538, 1489, 1475, 1459, 1418, 1393, 1365, 1329, 1295, 1262, 1151, 1140, 1100, 1070, 925, 918, 849, 819, 762, 754, 709, 666, 640, 567, 537, 494, 460, 450, 424 cm^{-1}. MS (EI, DEP, 70 eV): *m/z* (%) = 238.9 (5) [M^+], 182.9 (61), [$M+H-C_4H_9$]$^+$, 165.9 (100) [$M-C_4H_9O$]$^+$, 152.9 (9), 119.8 (53) [$M-C_4H_9O-NO_2$]$^+$, 91.9 (27). ESI-MS (-): *m/z* (%) = 499.1 (70) [2M-2H+Na]$^-$, 238.0 (100) [M-H]$^-$. HR-MS (ESI, -): calcd. for [$C_{11}H_{13}NO_5$-H]$^-$: 238.0715, found: 238.0716 (Δ = 0.4 ppm).

***tert*-Butyl 4-[(5-bromopentyl)oxy]benzoate (9)**

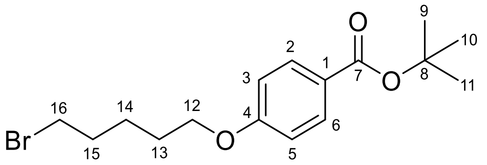

The product was synthesized following general procedure A using *tert*-butyl 4-hydroxybenzoate (**18**) (0.500 g, 2.57 mmol, 1.00 eq.) and sodium hydride (60%, 0.154 g, 3.86 mmol, 1.50 eq.) in dry DMF (12 mL); 1 h at 0 °C, 1 h at rt; 1,5-dibromopentane (1.776 g, 1.05 mL, 7.72 mmol, 3.00 eq.); 16 h at rt; water (10 mL); variant 1: DCM (3 x 20 mL), water (2 x 20 mL), column chromatography (petroleum ether:ethyl acetate = 20:1 to 2:1). The product

was isolated as a colorless solid, yield: 0.702 g (2.05 mmol, 79%). m.p. (DSC): 78 °C. ^{1}H NMR (400 MHz, $CDCl_3$): δ = 7.92 (d, $J_{H,H}$ = 9.0 Hz, 2 H, H-2, H-6), 6.87 (d, $J_{H,H}$ = 9.0 Hz, 2 H, H-3, H-5), 4.01 (t, $J_{H,H}$ = 6.3 Hz, 2 H, H-12), 3.44 (t, $J_{H,H}$ = 6.7 Hz, 2 H, H-16), 1.98 – 1.91 (m, 2 H, H-15), 1.87 – 1.80 (m, 2 H, H-13), 1.67 – 1.61 (m, 2 H, H-14), 1.58 (s, 9 H, H-9, H-10, H-11) ppm. ^{13}C NMR (100 MHz, $CDCl_3$): δ = 165.6 (o, 1 C, C-7), 162.3 (o, 1 C, C-4), 131.3 (+, 2 C, C-2, C-6), 124.4 (o, 1 C, C-1), 113.8 (+, 2 C, C-3, C-5), 80.5 (o, 1 C, C-8), 67.7 (-, 1 C, C-12), 33.5 (-, 1 C, C-16), 32.4 (-, 1 C, C-15), 28.3 (-, 1 C, C-13), 28.2 (+, 3 C, C-9, C-10, C-11), 24.8 (-, 1 C, C-14) ppm. IR (ATR): $\tilde{\nu}$ = 3007, 2963, 2931, 2887, 2864, 2835, 1706, 1602, 1579, 1510, 1469, 1443, 1390, 1364, 1315, 1292, 1247, 1152, 1114, 1105, 1045, 1012, 973, 922, 869, 848, 827, 813, 789, 770, 755, 740, 697, 646, 631, 562, 531, 511, 478, 465 cm^{-1}. MS (EI, DEP, 20 eV): *m/z* (%) = 342.2 (22) [M^{+}], 286.2 (19) [M+H-C_4H_9]$^{+}$, 271.2 (6), 159.1 (36), 151.1 (100) [M+H-C_4H_9-C_4H_8Br]$^{+}$, 138.1 (75) [M+2H-C_4H_9-C_5H_{10}Br]$^{+}$, 128.2 (18), 95.2 (12). HR-MS (EI, +): calcd. for [$C_{16}H_{23}BrO_3$]$^{+}$: 342.0831, found: 342.0831 (Δ = 0.0 ppm).

tert-Butyl 4-[(5-bromopentyl)oxy]-3-chlorobenzoate (10)

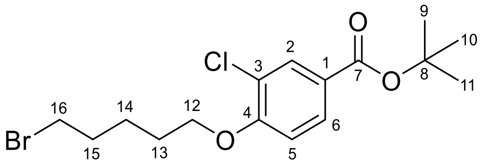

The product was synthesized following general procedure A using *tert*-butyl 3-chloro-4-hydroxybenzoate (**19**) (1.200 g, 5.25 mmol, 1.00 eq.) and sodium hydride (60%, 0.315 g, 7.87 mmol, 1.50 eq.) in dry DMF (12 mL); 1 h at 0 °C, 2 h at rt; 1,5-dibromopentane (4.830 g, 2.84 mL, 20.99 mmol, 4.00 eq.); 16 h at rt; water (10 mL); variant 1: DCM (3 x 40 mL), water (4 x 40 mL), column chromatography (petroleum ether:ethyl acetate = 15:1 to 0:1). The product was isolated as a colorless solid, yield: 1.662 g (4.40 mmol, 84%). m.p. (DSC): 54 °C. ^{1}H NMR (600 MHz, $CDCl_3$): δ = 7.97 (d, $J_{H,H}$ = 2.1 Hz, 1 H, H-2), 7.85 (dd, $J_{H,H}$ = 8.6, 2.1 Hz, 1 H, H-6), 6.89 (d, $J_{H,H}$ = 8.6 Hz, 1 H, H-5), 4.09 (t, $J_{H,H}$ = 6.3 Hz, 2 H, H-12), 3.45 (t, $J_{H,H}$ = 6.8 Hz, 2 H, H-16), 1.99 – 1.94 (m, 2 H, H-15), 1.91 – 1.87 (m, 2 H, H-13), 1.70 – 1.65 (m, 2 H, H-14), 1.58 (s, 9 H, H-9, H-10, H-11) ppm. ^{13}C NMR (150 MHz, $CDCl_3$): δ = 164.5 (o, 1 C, C-7), 157.7 (o, 1 C, C-4), 131.5 (+, 1 C, C-2), 129.5 (+, 1 C, C-6), 125.1 (o, 1 C, C-1), 122.5 (o, 1 C, C-3), 111.9 (+, 1 C, C-5), 81.1 (o, 1 C, C-8), 68.8 (-, 1 C, C-12), 33.5 (-, 1 C, C-16), 32.3 (-, 1 C, C-15), 28.2 (+, 3 C, C-9, C-10, C-11), 28.1 (-, 1 C, C-13), 24.7 (-, 1 C, C-14) ppm. IR (ATR): $\tilde{\nu}$ = 3003, 2974, 2952, 2926, 2909, 2875, 2855, 1696, 1596, 1573, 1499, 1474, 1457, 1440, 1430, 1405, 1388, 1366, 1296, 1277, 1231, 1167, 1152, 1127, 1052, 1041, 1012, 964, 921, 911, 903, 855, 825, 790, 766, 738, 711, 649, 566, 554, 507, 497, 465, 453, 446, 416 cm^{-1}. MS (EI, DEP, 70 eV): *m/z* (%) = 376.2 (2) [M^{+}], 319.8 (1) [M+H-C_4H_9]$^{+}$, 302.8 (3) [M-C_4H_9O]$^{+}$, 171.7 (100) [M+2H-C_4H_9-C_5H_{10}Br]$^{+}$, 148.7 (78) [C_5H_{10}Br]$^{+}$, 99.0 (5). HR-MS (ESI, +): calcd. for [$C_{16}H_{22}BrClO_3$+Na]$^{+}$: 399.0339, found: 399.0330 (Δ = 2.3 ppm).

tert-Butyl 4-[(5-bromopentyl)oxy]-3-nitrobenzoate (11)

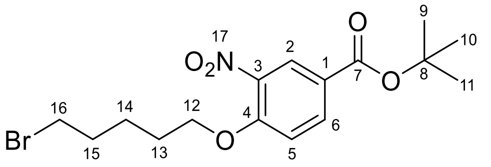

The product was synthesized following general procedure A using *tert*-butyl 4-hydroxy-3-nitrobenzoate (**20**) (1.000 g, 4.18 mmol, 1.00 eq.) and sodium hydride (60%, 0.251 g, 6.27 mmol, 1.50 eq.) in dry DMF (10 mL); 1 h at 0 °C, 2 h at rt; 1,5-dibromopentane (3.840 g, 2.26 mL, 16.72 mmol, 4.00 eq.); 5 h at 70 °C, 16 h at rt; water (10 mL); variant 1: DCM (3 x 50 mL), water (3 x 100 mL), column chromatography (petroleum ether:ethyl acetate = 8:1). The product was isolated as a pale yellow oil, yield: 0.601 g (1.55 mmol, 37%). ^{1}H NMR (400 MHz, $CDCl_3$): δ = 8.39 (d, $J_{H,H}$ = 2.2 Hz, 1 H, H-2), 8.13 (dd, $J_{H,H}$ = 8.8, 2.2 Hz, 1 H, H-6), 7.07 (d, $J_{H,H}$ = 8.8 Hz, 1 H, H-5), 4.16 (t, $J_{H,H}$ = 6.2 Hz, 2 H, H-12), 3.44 (t, $J_{H,H}$ = 6.7 Hz, 2 H, H-16), 1.97 – 1.84 (m, 4 H, H-13, H-15), 1.69 – 1.64 (m, 2 H, H-14), 1.58 (s, 9 H, H-9, H-10, H-11) ppm. ^{13}C NMR (100 MHz, $CDCl_3$): δ = 163.5 (o, 1 C, C-7), 155.0 (o, 1 C, C-4), 139.3 (o, 1 C, C-3), 135.0 (+, 1 C, C-6), 126.9 (+, 1 C, C-2), 124.3 (o, 1 C, C-1), 113.5 (+, 1 C, C-5), 81.9 (o, 1 C, C-8), 69.5 (-, 1 C, C-12), 33.4 (-, 1 C, C-16), 32.2 (-, 1 C, C-15), 28.1 (+, 3 C, C-9, C-10, C-11), 27.9 (-, 1 C, C-13), 24.5 (-, 1 C, C-14) ppm. IR (ATR): $\tilde{\nu}$ = 2976, 2938, 2879, 1711, 1615, 1572, 1533, 1499, 1473, 1458, 1393, 1367, 1355, 1276, 1247, 1165, 1121, 1079, 1033, 1004, 980, 919, 848, 837, 761, 737, 711, 687, 641, 561, 532, 496, 479, 463, 452 cm^{-1}. MS (EI, DEP, 70 eV): *m/z* (%) = 387.1 (2) $[M^+]$, 313.9 (3) $[M-C_4H_9O]^+$, 184.0 (12), 165.9 (29) $[C_5H_{10}BrO]^+$, 148.9 (100) $[C_5H_{10}Br]^+$, 120.1 (14). HR-MS (ESI, +): calcd. for $[C_{16}H_{22}BrNO_5+Na]^+$: 410.0579, found: 410.0580 (Δ = 0.2 ppm).

Methyl 4-sulfanylbenzoate (12)[22,23]

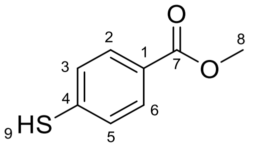

Compound **12** was prepared according to literature.[22,23] Conc. sulfuric acid (0.442 g, 0.24 m, 4.50 mmol, 0.20 eq.) was added to a solution of 4-sulfanylbenzoic acid (**21**) (3.524 g, 22.86 mmol, 1.00 eq.) in methanol (10 mL). The mixture was refluxed for 6 h before beeing quenched with ethyl acetate (30 mL). The solution was concentrated, washed with water (2 x 25 mL) and an aq. saturated solution of $NaHCO_3$ (20 mL). The combined organic phases were dried over magnesium sulfate, concentrated and dried *in vacuo*. The product was isolated as a pale yellow solid, yield: 3.034 g (18.04 mmol, 79%). m.p. (DSC): 50 °C. ^{1}H NMR (400 MHz, $CDCl_3$): δ = 7.88 (d, $J_{H,H}$ = 8.5 Hz, 2 H, H-2, H-6), 7.28 (d, $J_{H,H}$ = 8.5 Hz, 2 H, H-3, H-5), 3.89 (s, 3 H, H-8), 3.60 (s, 1 H, SH-9) ppm. ^{13}C NMR (100 MHz, $CDCl_3$): δ = 166.6 (o, 1 C, C-7), 138.3 (o, 1 C, C-4), 130.2 (+, 2 C, C-2, C-6), 128.1 (+, 2 C, C-3, C-5), 127.1 (o, 1 C, C-1), 52.0 (+, 1 C, C-8) ppm. IR (ATR): $\tilde{\nu}$ = 3462, 3031, 2997, 2948, 2845, 2578, 1704, 1592, 1561, 1489, 1453, 1434, 1401, 1370, 1308, 1275, 1194, 1179, 1110, 1013, 957, 901, 855, 839, 829, 756, 691, 631, 521, 488, 466 cm^{-1}. MS (EI, DEP, 70 eV): *m/z* (%) = 168.0 (49) $[M^+]$, 137.0 (100) $[M-CH_3O]^+$, 109.0 (39) $[M-C_2H_3O_2]^+$.

tert-Butyl [3-[(6-chloropyridin-3-yl)methyl]-2-(nitroimino)imidazolidin-1-yl]acetate (7a)

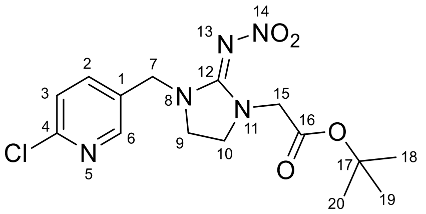

The product was synthesized following general procedure A using Imidacloprid (**1**) (3.000 g, 11.73 mmol, 1.00 eq.) and sodium hydride (60%, 0.704 g, 17.60 mmol, 1.50 eq.) in dry DMF (8 mL); 1 h at 0 °C, 2 h at rt; *tert*-butyl 2-bromoacetate (**7**) (3.320 g, 2.50 mL, 17.01 mmol, 1.45 eq.); 3 d at rt; water (50 mL); variant 2: water (2 x 40 mL), petroleum ether (1 x 20 mL). The product was isolated as a beige solid, yield: 3.768 g (10.19 mmol, 87%). m.p. (DSC): 121 °C. ^{1}H NMR (600 MHz, $CDCl_3$): δ = 8.26 (d, $J_{H,H}$ = 2.4 Hz, 1 H, H-6), 7.66 (dd, $J_{H,H}$ = 8.2, 2.4 Hz, 1 H, H-2), 7.29 (d, $J_{H,H}$ = 8.2 Hz, 1 H, H-3), 4.44 (s, 2 H, H-7), 4.00 (s, 2 H, H-15), 3.82 – 3.79 (m, 2 H, H-10), 3.60 – 3.56 (m, 2 H, H-9), 1.40 (s, 9 H, H-18, H-19, H-20) ppm. ^{13}C NMR (151 MHz, $CDCl_3$): δ = 166.3 (o, 1 C, C-16), 161.1 (o, 1 C, C-12), 151.4 (o, 1 C, C-4), 149.1 (+, 1 C, C-6), 138.8 (+, 1 C, C-2), 129.0 (o, 1 C, C-1), 124.6 (+, 1 C, C-3), 83.0 (o, 1 C, C-17), 48.5 (-, 1 C, C-15), 47.2 (-, 1 C, C-7), 46.9 (-, 1 C, C-10), 45.3 (-, 1 C, C-9), 27.8 (+, 3 C, C-18, C-19, C-20) ppm. ^{15}N NMR (61 MHz, $CDCl_3$): δ = -11.7 (1 N, N-14), -69.8 (1 N, N-5), -144.3 (1 N, N-13), -279.3 (1 N, N-8), -285.4 (1 N, N-11) ppm. IR (ATR): $\tilde{\nu}$ = 1740, 1578, 1549, 1463, 1442, 1419, 1389, 1363, 1356, 1309, 1287, 1251, 1228, 1212, 1162, 1132, 1101, 1034, 1023, 990, 967, 951, 944, 935, 916, 861, 839, 822, 812, 783, 767, 744, 679, 659, 643, 632, 581, 525, 493, 459, 424, 410 cm^{-1}. MS (ESI, +): *m/z* (%) = 370.1 (70) $[M+H]^+$. HR-MS (ESI, +): calcd. for $[C_{15}H_{20}ClN_5O_4+Na]^+$: 392.1102, found: 392.1100 (Δ = 0.5 ppm).

tert-Butyl 4-{[3-[(6-chloropyridin-3-yl)methyl]-2-(nitroimino)imidazolidin-1-yl]methyl}benzoate (8a)

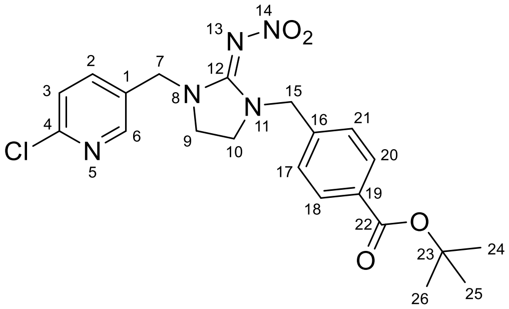

The product was synthesized following general procedure A using Imidacloprid (**1**) (1.400 g, 5.48 mmol, 1.00 eq.) and sodium hydride (60%, 0.329 g, 8.21 mmol, 1.50 eq.) in dry DMF (4.5 mL); 15 min at 0 °C, 2 h at rt; *tert*-butyl 4-(bromomethyl)benzoate (**8**) (1.782 g, 6.57 mmol, 1.20 eq.); 3 d at rt; water (20 mL); variant 3: DCM (3 x 40 mL), water (3 x 40 mL); methanol (2 mL), petroleum ether (12 mL); ethanol (5 mL). The product was isolated as a pale beige solid, yield: 1.948 g (4.37 mmol, 80%). m.p. (DSC): 124 °C. ^{1}H NMR (600 MHz, $CDCl_3$): δ = 8.34 (d, $J_{H,H}$ = 2.1 Hz, 1 H, H-6), 7.97 (d, $J_{H,H}$ = 8.1 Hz, 2 H, H-18, H-20), 7.74 (dd, $J_{H,H}$ = 8.1, 2.1 Hz, 1 H, H-2), 7.36 (d, $J_{H,H}$ = 8.1 Hz, 2 H, H-17, H-21), 7.35 (d, $J_{H,H}$ = 8.1 Hz, 1 H, H-3), 4.55 (s, 2 H, H-15), 4.53 (s, 2 H, H-7), 3.66 – 3.63 (m, 2 H, H-9), 3.62 – 3.59 (m, 2 H, H-10), 1.59 (s, 9 H, H-24, H-25, H-26) ppm. ^{13}C NMR (151 MHz, $CDCl_3$): δ = 165.0 (o, 1 C, C-22), 161.1 (o, 1 C, C-12), 151.6 (o, 1 C, C-4), 149.3 (+, 1 C, C-6), 139.0 (+, 1 C, C-2),

138.1 (o, 1 C, C-16), 132.2 (o, 1 C, C-19), 130.0 (+. 2 C, C-18, C-20), 128.8 (o, 1 C, C-1), 128.0 (+, 2 C, C-17, C-21), 124.7 (+, 1 C, C-3), 81.2 (o, 1 C, C-23), 50.2 (-, 1 C, C-15), 47.4 (-, 1 C, C-7), 45.1 (-, 1 C, C-9), 44.9 (-, 1 C, C-10), 28.0 (+, 3 C, C-24, C-25, C-26) ppm. ^{15}N NMR (61 MHz, CDCl$_3$): δ = -11.1 (1 N, N-14), -69.8 (1 N, N-5), -145.9 (1 N, N-13), -275.2 (1 N, N-11), -277.8 (1 N, N-8) ppm. IR (ATR): $\tilde{\nu}$ = 2972, 2929, 1699, 1613, 1582, 1564, 1543, 1476, 1449, 1411, 1391, 1370, 1265, 1203, 1187, 1162, 1109, 1020, 997, 969, 937, 849, 832, 811, 774, 758, 739, 709, 691, 670, 635, 629, 495, 453, 429, 409 cm^{-1}. MS (ESI, +): *m/z* (%) = 468.1 (100) [M+Na]$^+$. HR-MS (ESI, +): calcd. for [$C_{21}H_{24}ClN_5O_4$+Na]$^+$: 468.1415, found: 468.1413 (Δ = 0.4 ppm).

***tert*-Butyl 4-({5-[3-[(6-chloropyridin-3-yl)methyl]-2-(nitroimino)imidazolidin-1-yl]pentyl}oxy)benzoate (9a)**

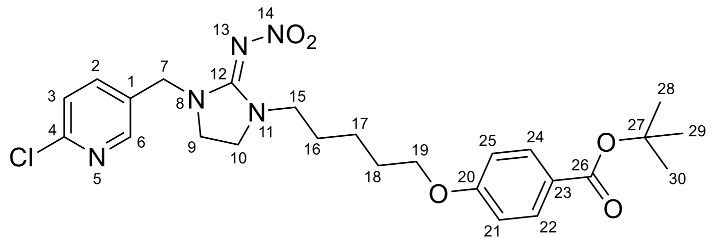

Procedure I starting from Imidacloprid (9a): The product was synthesized following general procedure A using Imidacloprid (**1**) (0.300 g, 1.17 mmol, 1.00 eq.) and sodium hydride (60%, 0.070 g, 1.76 mmol, 1.50 eq.) in dry DMF (4.5 mL); 15 min at 0 °C, 2 h at rt; *tert*-butyl 4-[(5-bromopentyl)oxy]benzoate (**9**) (0.443 g, 1.29 mmol, 1.10 eq.); 3 d at rt; water (20 mL); variant 1: DCM (3 x 40 mL), water (3 x 40 mL), column chromatography (ethyl acetate). The product was isolated as a colorless solid, yield: 0.254 g (0.49 mmol, 42%). m.p. (DSC): 93 °C. ^{1}H NMR (600 MHz, CDCl$_3$): δ = 8.32 (d, $J_{H,H}$ = 2.0 Hz, 1 H, H-6), 7.91 (d, $J_{H,H}$ = 8.8 Hz, 2 H, H-22, H-24), 7.70 (dd, $J_{H,H}$ = 8.1, 2.0 Hz, 1 H, H-2), 7.33 (d, $J_{H,H}$ = 8.1 Hz, 1 H, H-3), 6.87 (d, $J_{H,H}$ = 8.8 Hz, 2 H, H-21, H-25), 4.47 (s, 2 H, H-7), 4.00 (t, $J_{H,H}$ = 6.2 Hz, 2 H, H-19), 3.77 – 3.74 (m, 2 H, H-10), 3.63 – 3.60 (m, 2 H, H-9), 3.34 (t, $J_{H,H}$ = 7.5 Hz, 2 H, H-15), 1.84 – 1.79 (m, 2 H, H-18), 1.72 – 1.67 (m, 2 H, H-16), 1.58 (s, 9 H, H-28, H-29, H-30), 1.52 – 1.47 (m, 2 H, H-17) ppm. ^{13}C NMR (151 MHz, CDCl$_3$): δ = 165.4 (o, 1 C, C-26), 162.1 (o, 1 C, C-20), 161.0 (o, 1 C, C-12), 151.4 (o, 1 C, C-4), 149.2 (+, 1 C, C-6), 138.9 (+, 1 C, C-2), 131.1 (+, 2 C, C-22, C-24), 129.0 (o, 1 C, C-1), 124.6 (+, 1 C, C-3), 124.2 (o, 1 C, C-23), 113.6 (+, 2 C, C-21, C-25), 80.3 (o, 1 C, C-27), 67.4 (-, 1 C, C-19), 47.1 (-, 1 C, C-7), 46.5 (-, 1 C, C-15), 45.4 (-, 1 C, C-10), 44.9 (-, 1 C, C-9), 28.4 (-, 1 C, C-18), 28.0 (+, 3 C, C-28, C-29, C-30), 26.2 (-, 1 C, C-16), 22.9 (-, 1 C, C-17) ppm. ^{15}N NMR (61 MHz, CDCl$_3$): δ = -13.2 (1 N, N-14), -72.1 (1 N, N-5), -148.6 (1 N, N-13), -276.9 (1 N, N-11), -281.4 (1 N, N-8) ppm. IR (ATR): $\tilde{\nu}$ = 2965, 2939, 2866, 1699, 1606, 1553, 1538, 1509, 1466, 1451, 1429, 1394, 1369, 1291, 1263, 1249, 1188, 1159, 1112, 1102, 1070, 1028, 1007, 974, 938, 909, 868, 849, 818, 772, 752, 696, 667, 657, 643, 543, 518, 491, 428 cm^{-1}. MS (ESI, +): *m/z* (%) = 540.1 (100) [M+Na]$^+$, 518.2 (24) [M+H]$^+$. HR-MS (ESI, +): calcd. for [$C_{25}H_{32}ClN_5O_5$+Na]$^+$: 540.1990, found: 540.1988 (Δ = 0.4 ppm).

***tert*-Butyl 3-chloro-4-({5-[3-[(6-chloropyridin-3-yl)methyl]-2-(nitroimino)imidazolidin-1-yl]pentyl}oxy)benzoate (10a)**

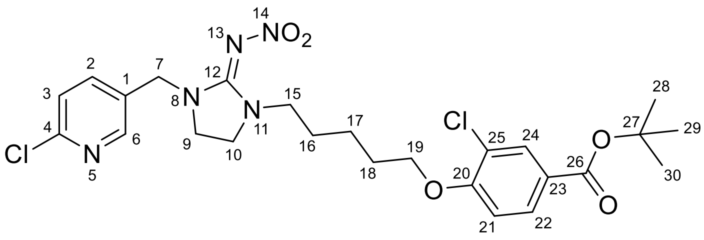

Procedure I: The product was synthesized following general procedure A using Imidacloprid (**1**) (0.750 g, 2.93 mmol, 1.00 eq.) and sodium hydride (60%, 0.176 g, 4.40 mmol, 1.50 eq.) in dry DMF (6 mL); 15 min at 0 °C, 2 h at rt; *tert*-butyl 4-[(5-bromopentyl)oxy]-3-chlorobenzoate (**10**) (1.219 g, 3.23 mmol, 1.10 eq.); 3 d at rt; water (40 mL); variant 1: DCM (3 x 50 mL), water (4 x 50 mL), column chromatography (ethyl acetate). The product was isolated as a colorless solid, yield: 0.947 g (1.71 mmol, 58%). **Procedure II:** The product was synthesized following general procedure A using Imidacloprid (**1**) (0.950 g, 3.72 mmol, 1.00 eq.) and sodium hydride (60%, 0.223 g, 5.57 mmol, 1.50 eq.) in dry DMF (6 mL); 15 min at 0 °C, 2 h at rt; *tert*-butyl 4-[(5-bromopentyl)oxy]-3-chlorobenzoate (**10**) (1.544 g, 4.09 mmol, 1.10 eq.); 3 d at rt; saturated aq. solution of NH_4Cl (40 mL); variant 1: DCM (3 x 50 mL), water (4 x 50 mL), column chromatography (ethyl acetate). The product was isolated as a colorless solid, yield: 1.463 g (2.65 mmol, 71%). m.p. (DSC): 78 °C. ^{1}H NMR (600 MHz, $CDCl_3$): δ = 8.27 (dd, $J_{H,H}$ = 2.5, 0.6 Hz, 1 H, H-6), 7.90 (d, $J_{H,H}$ = 2.1 Hz, 1 H, H-24), 7.80 (dd, $J_{H,H}$ = 8.7, 2.1 Hz, 1 H, H-22), 7.65 (dd, $J_{H,H}$ = 8.2, 2.5 Hz, 1 H, H-2), 7.28 (dd, $J_{H,H}$ = 8.2, 0.6 Hz, 1 H, H-3), 6.86 (d, $J_{H,H}$ = 8.7 Hz, 1 H, H-21), 4.42 (s, 2 H, H-7), 4.03 (t, $J_{H,H}$ = 6.1 Hz, 2 H, H-19), 3.73 – 3.70 (m, 2 H, H-10), 3.57 – 3.54 (m, 2 H, H-9), 3.31 (t, $J_{H,H}$ = 7.5 Hz, 2 H, H-15), 1.85 – 1.81 (m, 2 H, H-18), 1.69 – 1.65 (m, 2 H, H-16), 1.52 (s, 9 H, H-28, H-29, H-30), 1.51 – 1.47 (m, 2 H, H-17) ppm. ^{13}C NMR (151 MHz, $CDCl_3$): δ = 164.3 (o, 1 C, C-26), 161.1 (o, 1 C, C-12), 157.4 (o, 1 C, C-20), 151.5 (o, 1 C, C-4), 149.2 (+, 1 C, C-6), 139.0 (+, 1 C, C-2), 131.2 (+, 1 C, C-24), 129.5 (+, 1 C, C-22), 129.0 (o, 1 C, C-1), 125.0 (o, 1 C, C-23), 124.6 (+, 1 C, C-3), 122.1 (o, 1 C, C-25), 111.9 (+, 1 C, C-21), 81.0 (o, 1 C, C-27), 68.6 (-, 1 C, C-19), 47.2 (-, 1 C, C-7), 46.5 (-, 1 C, C-15), 45.4 (-, 1 C, C-10), 45.0 (-, 1 C, C-9), 28.2 (-, 1 C, C-18), 28.0 (+, 3 C, C-28, C-29, C-30), 26.2 (-, 1 C, C-16), 22.9 (-, 1 C, C-17) ppm. ^{15}N NMR (61 MHz, $CDCl_3$): δ = -10.9 (1 N, N-14), -69.9 (1 N, N-5), -146.1 (1 N, N-13), -274.7 (1 N, N-11), -279.1 (1 N, N-8) ppm. IR (ATR): $\tilde{\nu}$ = 2976, 2935, 2873, 1704, 1597, 1560, 1538, 1498, 1459, 1420, 1390, 1368, 1250, 1165, 1112, 1056, 1024, 1002, 973, 950, 902, 848, 816, 765, 733, 707, 641, 631, 495, 466, 445, 428, 412 cm^{-1}. MS (ESI, +): *m/z* (%) = 574.2 (100) [M+Na]$^+$. HR-MS (ESI, +): calcd. for [$C_{25}H_{31}Cl_2N_5O_5$+Na]$^+$: 574.1600, found: 574.1609 (Δ = 1.6 ppm).

tert-Butyl 4-({5-[3-[(6-chloropyridin-3-yl)methyl]-2-(nitroimino)imidazolidin-1-yl]pentyl}oxy)-3-nitrobenzoate (11a)

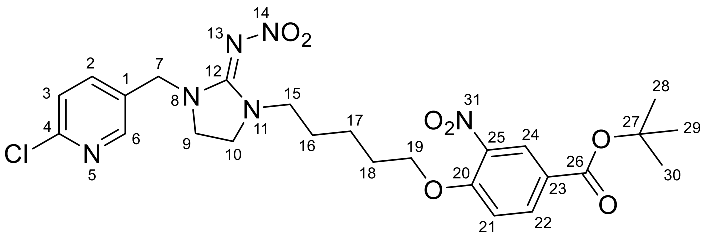

The product was synthesized following general procedure A using Imidacloprid (**1**) (0.539 g, 2.11 mmol, 1.00 eq.) and sodium hydride (60%, 0.127 g, 3.16 mmol, 1.50 eq.) in dry DMF (3 mL); 1 h at 0 °C, 2 h at rt; _tert_-butyl 4-[(5-bromopentyl)oxy]-3-nitrobenzoate (**11**) (0.901 g, 2.32 mmol, 1.10 eq.) in dry DMF (3 mL); 3 d at rt, 3 h at 60 °C; saturated aq. solution of NH_4Cl (40 mL); variant 1: DCM (3 x 50 mL), water (4 x 50 mL), column chromatography (ethyl acetate). The product was isolated as a beige solid, yield: 0.778 g (1.38 mmol, 66%). m.p. (DSC): 80 °C. ^1H NMR (600 MHz, $CDCl_3$): δ = 8.29 (d, $J_{H,H}$ = 2.2 Hz, 1 H, H-24), 8.24 (dd, $J_{H,H}$ = 2.5, 0.5 Hz, 1 H, H-6), 8.06 (dd, $J_{H,H}$ = 8.8, 2.2 Hz, 1 H, H-22), 7.64 (dd, $J_{H,H}$ = 8.2, 2.5 Hz, 1 H, H-2), 7.24 (dd, $J_{H,H}$ = 8.2, 0.5 Hz, 1 H, H-3), 7.04 (d, $J_{H,H}$ = 8.8 Hz, 1 H, H-21), 4.40 (s, 2 H, H-7), 4.10 (t, $J_{H,H}$ = 5.9 Hz, 2 H, H-19), 3.74 – 3.70 (m, 2 H, H-10), 3.60 – 3.57 (m, 2 H, H-9), 3.28 (t, $J_{H,H}$ = 7.2 Hz, 2 H, H-15), 1.81 – 1.77 (m, 2 H, H-18), 1.66 – 1.61 (m, 2 H, H-16), 1.51 (s, 9 H, H-28, H-29, H-30), 1.49 – 1.44 (m, 2 H, H-17) ppm. ^{13}C NMR (151 MHz, $CDCl_3$): δ = 163.3 (o, 1 C, C-26), 161.0 (o, 1 C, C-12), 154.8 (o, 1 C, C-20), 151.2 (o, 1 C, C-4), 149.2 (+, 1 C, C-6), 138.9 (o, 1 C, C-25), 138.9 (+, 1 C, C-2), 134.9 (+, 1 C, C-22), 129.0 (o, 1 C, C-1), 126.5 (+, 1 C, C-24), 124.5 (+, 1 C, C-3), 124.0 (o, 1 C, C-23), 113.6 (+, 1 C, C-21), 81.7 (o, 1 C, C-27), 69.2 (-, 1 C, C-19), 47.0 (-, 1 C, C-7), 46.1 (-, 1 C, C-15), 45.3 (-, 1 C, C-10), 45.0 (-, 1 C, C-9), 27.9 (-, 1 C, C-18), 27.9 (+, 3 C, C-28, C-29, C-30), 26.0 (-, 1 C, C-16), 22.6 (-, 1 C, C-17) ppm. ^{15}N NMR (61 MHz, $CDCl_3$): δ = -7.9 (1 N, N-31), -10.7 (1 N, N-14), -69.8 (1 N, N-5), -146.5 (1 N, N-13), -274.5 (1 N, N-11), -278.6 (1 N, N-8) ppm. IR (ATR): $\tilde{\nu}$ = 2975, 2937, 2872, 1709, 1615, 1560, 1530, 1458, 1418, 1390, 1367, 1248, 1164, 1122, 1078, 1024, 992, 971, 952, 919, 846, 837, 816, 759, 687, 650, 640, 631, 535, 495, 466, 425, 411 cm^{-1}. MS (ESI, +): _m/z_ (%) = 585.2 (100) $[M+Na]^+$. HR-MS (ESI, +): calcd. for $[C_{25}H_{31}ClN_6O_7+Na]^+$: 585.1840, found: 585.1836 (Δ = 0.7 ppm).

Methyl 4-{[1,1-dichloro-3-{1-[(6-chloropyridin-3-yl)methyl]imidazolidin-2-ylidene}-3-nitroprop-1-en-2-yl]sulfanyl}benzoate (12a)

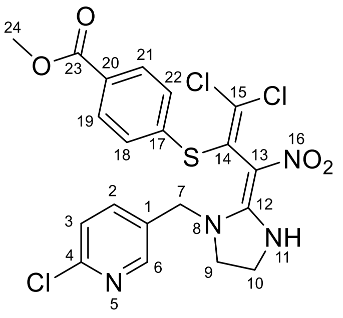

Methyl 4-sulfanylbenzoate (**12**) (0.964 g, 5.73 mmol, 1.10 eq.) was added to a solution of sodium ethanolate (0.602 g, 8.85 mmol, 1.70 eq.) in ethanol (100 mL) at 0 °C. The mixture was stirred for 2 h at rt before adding a solution of 2-chloro-5-{[(2_E_)-2-(2,3,3-trichloro-1-

nitroprop-2-en-1-ylidene)imidazolidin-1-yl]methyl}pyridine (**4**) (2.000 g, 5.21 mmol, 1.00 eq.) in ethanol (50 mL) at 0 °C. The mixture was stirred for 1h at rt and for 3 h at 40 °C. Afterwards, water (40 mL) was added, the solution was neutralized with conc. hydrochlorid acid and extracted with DCM (3 x 50 mL). The combined organic phases were washed with water (3 x 100 mL) and dried over magnesium sulfate. The residue was adsorbed on silica gel, purified by column chromatography (ethyl acetate) and dried *in vacuo*. The product was isolated as a yellow solid, yield: 1.566 g (3.04 mmol, 58%). m.p. (DSC): 158 °C (decomp.). ^{1}H NMR (600 MHz, DMSO-d_6): δ = 8.99 (s, 1 H, NH-11), 8.32 (dd, $J_{H,H}$ = 2.6, 0.6 Hz, 1 H, H-6), 7.90 (d, $J_{H,H}$ = 8.7 Hz, 2 H, H-19, H-21), 7.73 (dd, $J_{H,H}$ = 8.3, 2.6 Hz, 1 H, H-2), 7.52 (d, $J_{H,H}$ = 8.7 Hz, 2 H, H-18, H-22), 7.52 (dd, $J_{H,H}$ = 8.3, 0.6 Hz, 1 H, H-3), 4.52 (bs, 2 H, H-7), 3.85 (s, 3 H, H-24), 3.59 – 3.56 (m, 2 H, H-9), 3.45 (bs, 2 H, H-10) ppm. ^{13}C NMR (151 MHz, DMSO-d_6): δ = 165.6 (o, 1 C, C-23), 159.2 (o, 1 C, C-12), 149.5 (o, 1 C, C-4), 148.3 (+, 1 C, C-6), 138.3 (+, 1 C, C-2), 136.3 (o, 1 C, C-17), 133.1 (+, 2 C, C-18, C-22), 131.2 (o, 1 C, C-1), 129.7 (o, 1 C, C-20), 129.6 (o, 1 C, C-14), 129.5 (+, 2 C, C-19, C-21), 124.2 (+, 1 C, C-3), 120.0 (o, 1 C, C-15), 103.3 (o, 1 C, C-13), 52.3 (+, 1 C, C-24), 49.8 (-, 1 C, C-9), 48.5 (-, 1 C, C-7), 41.9 (-, 1 C, C-10) ppm. ^{15}N NMR (61 MHz, DMSO-d_6): δ = -17.3 (1 N, N-16), -72.9 (1 N, N-5), -277.0 (1 N, N-11), -287.0 (1 N, N-8) ppm. IR (ATR): $\tilde{\nu}$ = 3313, 2949, 2892, 1717, 1553, 1516, 1460, 1434, 1391, 1367, 1272, 1175, 1103, 1039, 1015, 951, 932, 856, 821, 759, 728, 713, 691, 649, 629, 580, 495, 474, 468, 438, 422, 410, 402 cm^{-1}. MS (ESI, +): *m/z* (%) = 537.0 (100) [M+Na]$^{+}$. HR-MS (ESI, +): calcd. for [$C_{20}H_{17}Cl_3N_4O_4S$+Na]$^{+}$: 536.9934, found: 536.9933 (Δ = 0.2 ppm).

[3-[(6-Chloropyridin-3-yl)methyl]-2-(nitroimino)imidazolidin-1-yl]acetic acid (7b)[24]

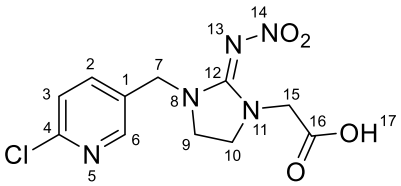

The product was synthesized following general procedure B using *tert*-butyl [3-[(6-chloropyridin-3-yl)methyl]-2-(nitroimino)imidazolidin-1-yl]acetate (**7a**) (1.318 g, 3.56 mmol, 1.00 eq.) in DCM (8 mL); trifluoroacetic acid (3.050 g, 2.06 mL, 26.70 mmol, 7.50 eq.); 1 d at rt; again trifluoroacetic acid (3.050 g, 2.06 mL, 26.70 mmol, 7.50 eq.); 1 d at rt; variant 1: methanol (4 mL), chloroform (4 mL). The product was isolated as a pale beige solid, yield: 1.115 g (3.56 mmol, 100%). m.p. (DSC): 126 °C (decomp.). ^{1}H NMR (600 MHz, DMSO-d_6): δ = 8.57 (bs, 1 H, OH-17), 8.34 (dd, $J_{H,H}$ = 2.5, 0.5 Hz, 1 H, H-6), 7.77 (dd, $J_{H,H}$ = 8.2, 2.5 Hz, 1 H, H-2), 7.52 (dd, $J_{H,H}$ = 8.2, 0.5 Hz, 1 H, H-3), 4.48 (s, 2 H, H-7), 3.98 (s, 2 H, H-15), 3.82 – 3.78 (m, 2 H, H-10), 3.71 – 3.67 (m, 2 H, H-9) ppm. ^{13}C NMR (151 MHz, DMSO-d_6): δ = 169.3 (o, 1 C, C-16), 161.0 (o, 1 C, C-12), 149.8 (o, 1 C, C-4), 149.4 (+, 1 C, C-6), 139.4 (+, 1 C, C-2), 130.6 (o, 1 C, C-1), 124.4 (+, 1 C, C-3), 47.5 (-, 1 C, C-15), 47.0 (-, 1 C, C-10), 46.5 (-, 1 C, C-7), 45.8 (-, 1 C, C-9) ppm. ^{15}N NMR (61 MHz, DMSO-d_6): δ = -15.9 (1 N, N-14), -73.4 (1 N, N-5), -148.5 (1 N, N-13), -282.3 (1 N, N-8), -287.6 (1 N, N-11) ppm. IR (ATR): $\tilde{\nu}$ = 3101, 2946, 2902, 1738, 1543, 1431, 1383, 1254, 1196, 1151, 1134, 1103, 1037, 1024, 1009, 992, 938, 816, 797, 774, 760, 748, 721, 694, 656, 628, 561, 520, 495, 432 cm^{-1}. MS (ESI, +): *m/z* (%) = 314.0 (53) [M+H]$^{+}$. HR-MS (ESI, +): calcd. for [$C_{11}H_{12}ClN_5O_4$+Na]$^{+}$: 336.0476, found: 336.0476 (Δ = 0.0 ppm).

4-{[3-[(6-Chloropyridin-3-yl)methyl]-2-(nitroimino)imidazolidin-1-yl]methyl}benzoic acid (8b)

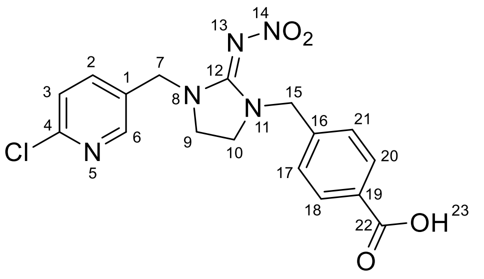

The product was synthesized following general procedure B using *tert*-butyl 4-{[3-[(6-chloropyridin-3-yl)methyl]-2-(nitroimino)imidazolidin-1-yl]methyl}benzoate (**8a**) (1.382 g, 3.10 mmol, 1.00 eq.) in DCM (8 mL); trifluoroacetic acid (5.300 g, 3.58 mL, 46.5 mmol, 15.00 eq.); 2 d at rt; again trifluoroacetic acid (5.300 g, 3.58 mL, 46.5 mmol, 15.00 eq.); 3 d at rt; variant 1: methanol (4 mL), chloroform (4 mL); The product was isolated as a pale beige solid, yield: 1.203 g (3.09 mmol, 100%). m.p. (SMP3): 205 °C (decomp.). ^{1}H NMR (600 MHz, DMSO-d_6): δ = 12.64 (bs, 1 H, OH-23), 8.38 (d, $J_{H,H}$ = 2.4 Hz, 1 H, H-6), 7.94 (d, $J_{H,H}$ = 8.3 Hz, 2 H, H-18, H-20), 7.82 (dd, $J_{H,H}$ = 8.2, 2.4 Hz, 1 H, H-2), 7.56 (d, $J_{H,H}$ = 8.2 Hz, 1 H, H-3), 7.43 (d, $J_{H,H}$ = 8.3 Hz, 2 H, H-17, H-21), 4.51 (s, 2 H, H-15), 4.48 (s, 2 H, H-7) 3.70 – 3.67 (m, 2 H, H-9), 3.65 – 3.62 (m, 2 H, H-10) ppm. ^{13}C NMR (151 MHz, DMSO-d_6): δ = 167.0 (o, 1 C, C-22), 160.9 (o, 1 C, C-12), 149.7 (o, 1 C, C-4), 149.5 (+, 1 C, C-6), 140.0 (o, 1 C, C-16), 139.5 (+, 1 C, C-2), 130.4 (o, 1 C, C-1), 130.3 (o, 1 C, C-19), 129.7 (+, 2 C, C-18, C-20), 127.9 (+, 2 C, C-17, C-21), 124.3 (+, 1 C, C-3), 49.2 (-, 1 C, C-15), 46.6 (-, 1 C, C-7), 45.6 (-, 1 C, C-9), 45.5 (-, 1 C, C-10) ppm. IR (ATR): $\tilde{\nu}$ = 2964, 2897, 2665, 2555, 1683, 1612, 1563, 1539, 1456, 1443, 1411, 1381, 1352, 1319, 1251, 1178, 1155, 1138, 1125, 1103, 1018, 938, 857, 835, 814, 789, 775, 758, 739, 711, 685, 662, 644, 629, 544, 497, 478, 464, 422, 412 cm^{-1}. MS (ESI, +): *m/z* (%) = 412.0 (100) [M+Na]$^{+}$. HR-MS (ESI, +): calcd. for $[C_{17}H_{16}ClN_5O_4+Na]^{+}$: 412.0789, found: 412.0789 (Δ = 0.0 ppm).

4-({5-[3-[(6-Chloropyridin-3-yl)methyl]-2-(nitroimino)imidazolidin-1-yl]pentyl}oxy)benzoic acid (9b)

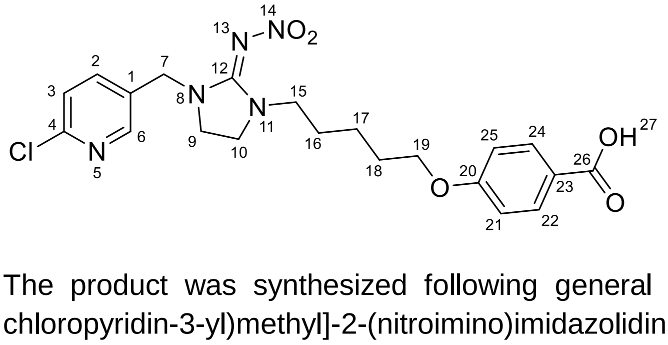

The product was synthesized following general procedure B using *tert*-butyl 4-({5-[3-[(6-chloropyridin-3-yl)methyl]-2-(nitroimino)imidazolidin-1-yl]pentyl}oxy)benzoate (**9a**) (0.583 g, 1.13 mmol, 1.00 eq.) in DCM (6 mL); trifluoroacetic acid (1.925 g, 1.30 mL, 16.88 mmol, 15.00 eq.); 3 d at rt; variant 2: methanol (4 mL), chloroform (4 mL); water (2 x 4 mL). The product was isolated as a beige solid, yield: 0.457 g (0.99 mmol, 88%). m.p. (DSC): 170 °C. ^{1}H NMR (600 MHz, DMSO-d_6): δ = 12.59 (s, 1 H, OH-27), 8.35 (dd, $J_{H,H}$ = 2.5, 0.5 Hz, 1 H, H-6), 7.87 (d, $J_{H,H}$ = 9.0 Hz, 2 H, H-22, H-24), 7.78 (dd, $J_{H,H}$ = 8.2, 2.5 Hz, 1 H, H-2), 7.54 (dd, $J_{H,H}$ = 8.2, 0.5 Hz, 1 H, H-3), 7.00 (d, $J_{H,H}$ = 9.0 Hz, 2 H, H-21, H-25), 4.43 (s, 2 H, H-7), 4.03 (t, $J_{H,H}$ = 6.5 Hz, 2 H, H-19), 3.76 – 3.73 (m, 2 H, H-10), 3.65 – 3.62 (m, 2 H, H-9), 3.22 (t,

$J_{H,H}$ = 7.3 Hz, 2 H, H-15), 1.76 – 1.72 (m, 2 H, H-18), 1.63 – 1.58 (m, 2 H, H-16), 1.41 – 1.36 (m, 2 H, H-17) ppm. ^{13}C NMR (151 MHz, DMSO-d_6): δ = 167.0 (o, 1 C, C-26), 162.2 (o, 1 C, C-20), 160.7 (o, 1 C, C-12), 149.7 (o, 1 C, C-4), 149.4 (+, 1 C, C-6), 139.4 (+, 1 C, C-2), 131.3 (+, 2 C, C-22, C-24), 130.6 (o, 1 C, C-1), 124.3 (+, 1 C, C-3), 122.8 (o, 1 C, C-23), 114.2 (+, 2 C, C-21, C-25), 67.6 (-, 1 C, C-19), 46.2 (-, 1 C, C-7), 45.6 (-, 1 C, C-15), 45.3 (-, 1 C, C-9), 45.2 (-, 1 C, C-10), 28.0 (-, 1 C, C-18), 25.7 (-, 1 C, C-16), 22.4 (-, 1 C, C-17) ppm. IR (ATR): $\tilde{\nu}$ = 2943, 2875, 1703, 1607, 1567, 1538, 1513, 1464, 1418, 1381, 1300, 1261, 1166, 1140, 1103, 1043, 1020, 1010, 994, 952, 850, 835, 813, 774, 734, 696, 630, 528, 510, 494, 478, 431, 418, 409 cm^{-1}. MS (ESI, +): *m/z* (%) = 484.2 (27) [M+Na]$^+$, 462.3 (100) [M+H]$^+$. HR-MS (ESI, +): calcd. for [$C_{21}H_{24}ClN_5O_5$+Na]$^+$: 484.1364, found: 484.1363 (Δ = 0.2 ppm).

3-Chloro-4-({5-[3-[(6-chloropyridin-3-yl)methyl]-2-(nitroimino)imidazolidin-1-yl]pentyl}oxy)benzoic acid (10b)

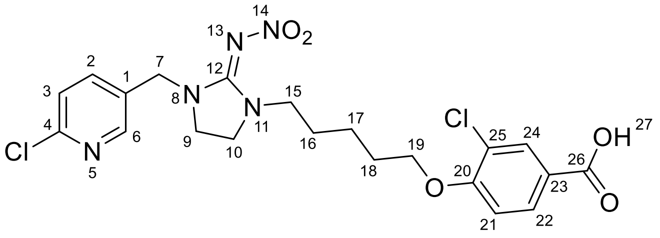

The product was synthesized following general procedure B using *tert*-butyl 3-chloro-4-({5-[3-[(6-chloropyridin-3-yl)methyl]-2-(nitroimino)imidazolidin-1-yl]pentyl}oxy)benzoate (**10a**) (0.941 g, 1.70 mmol, 1.00 eq.) in DCM (10 mL); trifluoroacetic acid (2.910 g, 1.97 mL, 25.50 mmol, 15.00 eq.); 3 d at rt; variant 1: methanol (4 mL), chloroform (4 mL). The product was isolated as a pale yellow solid, yield: 0.845 g (1.70 mmol, 100%). m.p. (SMP3): 105 °C. ^{1}H NMR (600 MHz, DMSO-d_6): δ = 12.81 (s, 1 H, OH-27), 8.35 (dd, $J_{H,H}$ = 2.5, 0.6 Hz, 1 H, H-6), 7.90 (d, $J_{H,H}$ = 2.1 Hz, 1 H, H-24), 7.87 (dd, $J_{H,H}$ = 8.7, 2.1 Hz, 1 H, H-22), 7.78 (dd, $J_{H,H}$ = 8.2, 2.5 Hz, 1 H, H-2), 7.54 (dd, $J_{H,H}$ = 8.2, 0.6 Hz, 1 H, H-3), 7.24 (d, $J_{H,H}$ = 8.7 Hz, 1 H, H-21), 4.43 (s, 2 H, H-7), 4.14 (t, $J_{H,H}$ = 6.4 Hz, 2 H, H-19), 3.76 – 3.73 (m, 2 H, H-10), 3.65 – 3.62 (m, 2 H, H-9), 3.22 (t, $J_{H,H}$ = 7.3 Hz, 2 H, H-15), 1.80 – 1.76 (m, 2 H, H-18), 1.65 – 1.60 (m, 2 H, H-16), 1.44 – 1.39 (m, 2 H, H-17) ppm. ^{13}C NMR (151 MHz, DMSO-d_6): δ = 166.1 (o, 1 C, C-26), 160.7 (o, 1 C, C-12), 157.4 (o, 1 C, C-20), 149.8 (o, 1 C, C-4), 149.4 (+, 1 C, C-6), 139.4 (+, 1 C, C-2), 130.8 (+, 1 C, C-24), 130.6 (o, 1 C, C-1), 130.0 (+, 1 C, C-22), 124.3 (+, 1 C, C-3), 123.8 (o, 1 C, C-23), 121.3 (o, 1 C, C-25), 113.2 (+, 1 C, C-21), 68.8 (-, 1 C, C-19), 46.3 (-, 1 C, C-7), 45.6 (-, 1 C, C-15), 45.3 (-, 1 C, C-9), 45.2 (-, 1 C, C-10), 27.9 (-, 1 C, C-18), 25.7 (-, 1 C, C-16), 22.4 (-, 1 C, C-17) ppm. IR (ATR): $\tilde{\nu}$ = 2935, 2873, 1674, 1593, 1565, 1542, 1503, 1461, 1413, 1390, 1370, 1319, 1277, 1252, 1216, 1149, 1126, 1109, 1055, 1037, 1023, 986, 967, 946, 914, 899, 869, 822, 764, 743, 708, 693, 670, 642, 571, 547, 493, 476, 441, 426, 411 cm^{-1}. MS (ESI, +): *m/z* (%) = 518.1 (100) [M+Na]$^+$. HR-MS (ESI, +): calcd. for [$C_{21}H_{23}Cl_2N_5O_5$+Na]$^+$: 518.0974, found: 518.0974 (Δ = 0.0 ppm).

4-({5-[3-[(6-Chloropyridin-3-yl)methyl]-2-(nitroimino)imidazolidin-1-yl]pentyl}-oxy)-3-nitrobenzoic acid (11b)

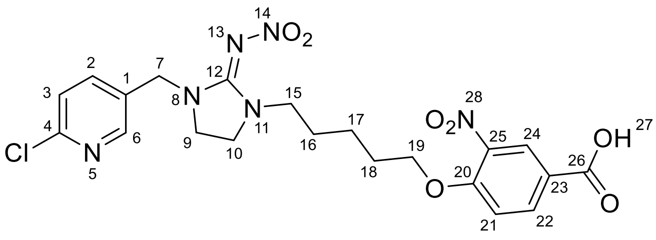

The product was synthesized following general procedure B using *tert*-butyl 4-({5-[3-[(6-chloropyridin-3-yl)methyl]-2-(nitroimino)imidazolidin-1-yl]pentyl}oxy)-3-nitrobenzoate (**11a**) (0.770 g, 1.37 mmol, 1.00 eq.) in chloroform (10 mL); trifluoroacetic acid (4.680 g, 3.16 mL, 41.00 mmol, 30.00 eq.); 3 d at rt, 7 h under reflux; variant 3: water (20 mL), chloroform (3 x 15 mL). The product was isolated as a yellow viscous liquid, yield: 0.642 g (1.27 mmol, 93%). ^{1}H NMR (600 MHz, DMSO-d_6): δ = 10.71 (s, 1 H, OH-27), 8.34 (dd, $J_{H,H}$ = 2.5, 0.5 Hz, 1 H, H-6), 8.33 (d, $J_{H,H}$ = 2.2 Hz, 1 H, H-24), 8.15 (dd, $J_{H,H}$ = 8.9, 2.2 Hz, 1 H, H-22), 7.77 (dd, $J_{H,H}$ = 8.3, 2.5 Hz, 1 H, H-2), 7.52 (dd, $J_{H,H}$ = 8.3, 0.5 Hz, 1 H, H-3), 7.45 (d, $J_{H,H}$ = 8.9 Hz, 1 H, H-21), 4.43 (s, 2 H, H-7), 4.24 (t, $J_{H,H}$ = 6.3 Hz, 2 H, H-19), 3.77 – 3.73 (m, 2 H, H-10), 3.66 – 3.62 (m, 2 H, H-9), 3.21 (t, $J_{H,H}$ = 7.3 Hz, 2 H, H-15), 1.78 – 1.74 (m, 2 H, H-18), 1.63 – 1.58 (m, 2 H, H-16), 1.42 – 1.37 (m, 2 H, H-17) ppm. ^{13}C NMR (151 MHz, DMSO-d_6): δ = 165.5 (o, 1 C, C-26), 160.7 (o, 1 C, C-12), 154.6 (o, 1 C, C-20), 149.7 (o, 1 C, C-4), 149.4 (+, 1 C, C-6), 139.4 (+, 1 C, C-2), 138.9 (o, 1 C, C-25), 135.2 (+, 1 C, C-22), 130.6 (o, 1 C, C-1), 126.1 (+, 1 C, C-24), 124.3 (+, 1 C, C-3), 122.8 (o, 1 C, C-23), 115.1 (+, 1 C, C-21), 69.5 (-, 1 C, C-19), 46.3 (-, 1 C, C-7), 45.6 (-, 1 C, C-15), 45.3 (-, 1 C, C-9), 45.2 (-, 1 C, C-10), 27.7 (-, 1 C, C-18), 25.6 (-, 1 C, C-16), 22.2 (-, 1 C, C-17) ppm. IR (ATR): $\tilde{\nu}$ = 3079, 2943, 2873, 1779, 1713, 1615, 1569, 1532, 1462, 1420, 1390, 1352, 1260, 1205, 1157, 1136, 1077, 1026, 993, 969, 951, 914, 819, 760, 699, 679, 634, 497, 474, 430, 408, 404 cm^{-1}. MS (ESI, +): *m/z* (%) = 1035.3 (10) [2M+Na]$^+$, 529.1 (100) [M+Na]$^+$, 507.1 (14) [M+H]$^+$. HR-MS (ESI, +): calcd. for $[C_{21}H_{23}ClN_6O_7+Na]^+$: 529.1214, found: 529.1206 (Δ = 1.5 ppm).

4-{[1,1-Dichloro-3-{1-[(6-chloropyridin-3-yl)methyl]imidazolidin-2-ylidene}-3-nitroprop-1-en-2-yl]sulfanyl}benzoic acid (12b)

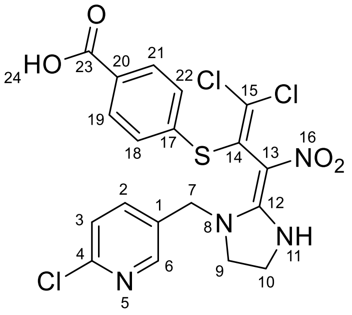

Sodium hydroxide (0.146 g, 3.64 mmol, 1.20 eq.) was added to a suspension of methyl 4-{[1,1-dichloro-3-{1-[(6-chloropyridin-3-yl)methyl]imidazolidin-2-ylidene}-3-nitroprop-1-en-2-yl]sulfanyl}benzoate (**12a**) (1.566 g, 3.04 mmol, 1.00 eq.) in water (250 mL). The mixture was stirred for 3 h at 50 °C, subsequently filtered and acidified with conc. hydrochloric acid to pH 1. The aq. phase was concentrated to dryness and the product dried *in vacuo*. The product was isolated as a pale orange solid, yield: 0.482 g (0.96 mmol, 32%). m.p. (DSC): 208 °C

(decomp.). ^{1}H NMR (600 MHz, DMSO-d_6): δ = 8.99 (s, 1 H, NH-11), 8.31 (dd, $J_{H,H}$ = 2.6, 0.6 Hz, 1 H, H-6), 7.88 (d, $J_{H,H}$ = 8.6 Hz, 2 H, H-19, H-21), 7.73 (dd, $J_{H,H}$ = 8.3, 2.6 Hz, 1 H, H-2), 7.53 (dd, $J_{H,H}$ = 8.3, 0.6 Hz, 1 H, H-3), 7.50 (d, $J_{H,H}$ = 8.6 Hz, 2 H, H-18, H-22), 4.52 (bs, 2 H, H-7), 3.58 – 3.56 (m, 2 H, H-9), 3.44 – 3.39 (m, 2 H, H-10) ppm. The carboxylic acid proton (1 H, OH 24) could not be detected due to signal broadening. ^{13}C NMR (151 MHz, DMSO-d_6): δ = 166.6 (o, 1 C, C-23), 159.2 (o, 1 C, C-12), 149.4 (o, 1 C, C-4), 148.3 (+, 1 C, C-6), 138.4 (+, 1 C, C-2), 135.5 (o, 1 C, C-17), 133.2 (+, 2 C, C-18, C-22), 131.3 (o, 1 C, C-1), 131.2 (o, 1 C, C-20), 129.9 (o, 1 C, C-14), 129.7 (+, 2 C, C-19, C-21), 124.3 (+, 1 C, C-3), 119.5 (o, 1 C, C-15), 103.3 (o, 1 C, C-13), 49.9 (-, 1 C, C-9), 48.4 (-, 1 C, C-7), 42.0 (-, 1 C, C-10) ppm. IR (ATR): $\tilde{\nu}$ = 3358, 3260, 1720, 1594, 1584, 1559, 1526, 1470, 1459, 1439, 1392, 1368, 1327, 1286, 1260, 1221, 1183, 1172, 1127, 1115, 1102, 1051, 1017, 990, 974, 964, 954, 934, 900, 858, 840, 831, 814, 792, 764, 753, 742, 727, 703, 687, 658, 651, 626, 609, 544, 529, 514, 492, 456, 436, 427, 412 cm^{-1}. MS (ESI, -): *m/z* (%) = 499.0 (100) [M-H]$^-$. HR-MS (ESI, -): calcd. for $[C_{19}H_{15}Cl_3N_4O_4S-H]^-$: 498.9801, found: 498.9806 (Δ = 1.0 ppm).

***N*-{1-[2-(1*H*-Benzotriazol-1-yl)-2-oxoethyl]-3-[(6-chloropyridin-3-yl)methyl]imidazolidin-2-ylidene}nitramide (7c)**

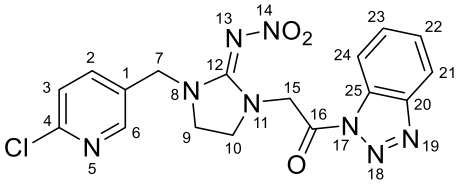

The product was synthesized following general procedure C using [3-[(6-chloropyridin-3-yl)methyl]-2-(nitroimino)imidazolidin-1-yl]acetic acid (**7b**) (1.490 g, 4.75 mmol, 1.00 eq.) and 1*H*-benzotriazole (1.992 g, 16.72 mmol, 3.52 eq.) in dry DCM (40 mL); thionyl chloride (0.706 g, 0.43 mL, 5.94 mmol, 1.25 eq.); 30 h at rt; water (20 mL); variant 3: water (2 x 40 mL), DCM (2 x 20 mL). The product was isolated as a colorless solid, yield: 1.349 g (3.25 mmol, 69%). m.p. (DSC): 194 °C (decomp.). ^{1}H NMR (600 MHz, DMSO-d_6): δ = 8.41 (dd, $J_{H,H}$ = 2.4, 0.7 Hz, 1 H, H-6), 8.30 (ddd, $J_{H,H}$ = 8.3, 1.0, 0.9 Hz, 1 H, H-21), 8.23 (ddd, $J_{H,H}$ = 8.2, 1.0, 0.9 Hz, 1 H, H-24), 7.84 (dd, $J_{H,H}$ = 8.3, 2.4 Hz, 1 H, H-2), 7.83 (ddd, $J_{H,H}$ = 8.2, 7.1, 1.0 Hz, 1 H, H-23), 7.65 (ddd, $J_{H,H}$ = 8.3, 7.1, 1.0 Hz, 1 H, H-22), 7.59 (dd, $J_{H,H}$ = 8.3, 0.7 Hz, 1 H, H-3), 5.17 (s, 2 H, H-15), 4.57 (s, 2 H, H-7), 4.01 – 3.97 (m, 2 H, H-10), 3.81 – 3.78 (m, 2 H, H-9) ppm. ^{13}C NMR (151 MHz, DMSO-d_6): δ = 166.9 (o, 1 C, C-16), 160.8 (o, 1 C, C-12), 149.8 (o, 1 C, C-4), 149.4 (+, 1 C, C-6), 145.3 (o, 1 C, C-20), 139.4 (+, 1 C, C-2), 131.2 (+, 1 C, C-23), 130.6 (o, 1 C, C-1), 130.4 (o, 1 C, C-25), 126.8 (+, 1 C, C-22), 124.4 (+, 1 C, C-3), 120.3 (+, 1 C, C-21), 113.7 (+, 1 C, C-24), 49.8 (-, 1 C, C-15), 47.5 (-, 1 C, C-10), 46.3 (-, 1 C, C-7), 45.8 (-, 1 C, C-9) ppm. ^{15}N NMR (61 MHz, DMSO-d_6): δ = -5.0 (1 N, N-18), -21.8 (1 N, N-19), -73.8 (1 N, N-5), -129.0 (1 N, N-17), -141.3 (1 N, N-13), -282.4 (1 N, N-8), -292.1 (1 N, N-11) ppm. The nitrogen atom of the nitro group (N-14) could not be detected due to insufficient solubility of the substance. IR (ATR): $\tilde{\nu}$ = 2997, 2969, 2909, 1760, 1607, 1594, 1573, 1558, 1538, 1482, 1467, 1458, 1443, 1421, 1413, 1380, 1342, 1307, 1292, 1277, 1266, 1230, 1211, 1166, 1137, 1121, 1100, 1071, 1044, 1021, 1009, 1000, 971, 938, 907, 832, 818, 805, 774, 763, 749, 736, 705, 688, 678, 660, 634, 629, 554, 538, 511, 500, 490, 468, 433, 413 cm^{-1}. MS (ESI, +): *m/z* (%) = 437.1 (100) [M+Na]$^+$. HR-MS (ESI, +): calcd. for $[C_{17}H_{15}ClN_8O_3+Na]^+$: 437.0853, found: 437.0858 (Δ = 1.1 ppm).

***N*-(1-{[4-(1*H*-Benzotriazole-1-carbonyl)phenyl]methyl}-3-[(6-chloropyridin-3-yl)methyl]imidazolidin-2-ylidene)nitramide (8c)**

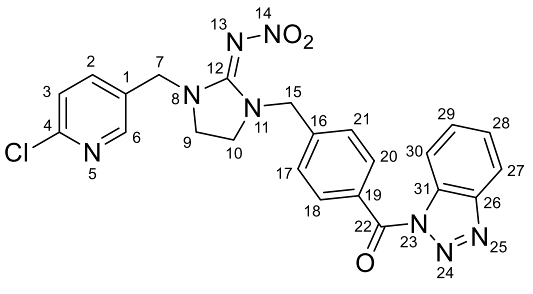

Procedure I: The product was synthesized following general procedure C using 4-{[3-[(6-chloropyridin-3-yl)methyl]-2-(nitroimino)imidazolidin-1-yl]methyl}benzoic acid (**8b**) (1.490 g, 3.82 mmol, 1.00 eq.) and 1*H*-benzotriazole (1.603 g, 13.46 mmol, 3.52 eq.) in dry DCM (30 mL); thionyl chloride (0.568 g, 0.35 mL, 4.78 mmol, 1.25 eq.); 24 h at rt; water (20 mL); variant 3: water (2 x 40 mL), DCM (2 x 20 mL). The product was isolated as a beige solid, yield: 1.407 g (2.87 mmol, 75%). **Procedure II:** The product was synthesized following general procedure C using 4-{[3-[(6-chloropyridin-3-yl)methyl]-2-(nitroimino)imidazolidin-1-yl]methyl}benzoic acid (**8b**) (1.136 g, 2.91 mmol, 1.00 eq.) and 1*H*-benzotriazole (1.222 g, 10.26 mmol, 3.52 eq.) in dry DCM (30 mL); thionyl chloride (0.555 g, 0.34 mL, 4.66 mmol, 1.60 eq.); 24 h at rt; water (20 mL); variant 4: DCM (3 x 50 mL); petroleum ether (20 mL); 2.0 molar solution of hydrochloric acid (aq., 2 x 20 mL), water (2 x 20 mL). The product was isolated as a beige solid, yield: 1.379 g (2.81 mmol, 96%). m.p. (DSC): 192 °C. ^{1}H NMR (600 MHz, DMSO-d_6): δ = 8.40 (dd, $J_{H,H}$ = 2.5, 0.6 Hz, 1 H, H-6), 8.33 (ddd, $J_{H,H}$ = 8.2, 1.0, 0.9 Hz, 1 H, H-30), 8.30 (ddd, $J_{H,H}$ = 8.3, 1.0, 0.9 Hz, 1 H, H-27), 8.13 (d, $J_{H,H}$ = 8.5 Hz, 2 H, H-18, H-20), 7.84 (dd, $J_{H,H}$ = 8.2, 2.5 Hz, 1 H, H-2), 7.84 (ddd, $J_{H,H}$ = 8.2, 7.1, 1.0 Hz, 1 H, H-29), 7.66 (ddd, $J_{H,H}$ = 8.3, 7.1, 1.0 Hz, 1 H, H-28), 7.58 (d, $J_{H,H}$ = 8.5 Hz, 2 H, H-17, H-21), 7.57 (dd, $J_{H,H}$ = 8.2, 0.6 Hz, 1 H, H-3), 4.60 (s, 2 H, H-15), 4.51 (s, 2 H, H-7), 3.72 (s, 4 H, H-9, H-10) ppm. ^{13}C NMR (151 MHz, DMSO-d_6): δ = 166.3 (o, 1 C, C-22), 160.9 (o, 1 C, C-12), 149.7 (o, 1 C, C-4), 149.5 (+, 1 C, C-6), 145.2 (o, 1 C, C-26), 140.9 (o, 1 C, C-16), 139.5 (+, 1 C, C-2), 131.8 (+, 2 C, C-18, C-20), 131.7 (o, 1 C, C-31), 130.9 (o, 1 C, C-19), 130.8 (+, 1 C, C-29), 130.4 (o, 1 C, C-1), 127.6 (+, 2 C, C-17, C-21), 126.7 (+, 1 C, C-28), 124.3 (+, 1 C, C-3), 120.1 (+, 1 C, C-27), 114.4 (+, 1 C, C-30), 49.2 (-, 1 C, C-15), 46.6 (-, 1 C, C-7), 45.7 (-, 1 C, C-9), 45.7 (-, 1 C, C-10) ppm. ^{15}N NMR (61 MHz, DMSO-d_6): δ = 1.6 (1 N, N-24), -16.2 (1 N, N-14), -23.2 (1 N, N-25), -73.9 (1 N, N-5), -127.4 (1 N, N-23), -149.9 (1 N, N-13), -280.7 (1 N, N-11), -281.4 (1 N, N-8) ppm. IR (ATR): $\tilde{\nu}$ = 3081, 2982, 2921, 1700, 1610, 1574, 1524, 1458, 1441, 1405, 1378, 1368, 1326, 1301, 1269, 1201, 1182, 1152, 1134, 1103, 1044, 1003, 968, 935, 906, 887, 848, 816, 807, 780, 772, 757, 736, 688, 657, 625, 583, 567, 538, 518, 504, 490, 477, 448, 432 cm^{-1}. MS (ESI, +): *m/z* (%) = 513.2 (100) [M+Na]$^+$. HR-MS (ESI, +): calcd. for [$C_{23}H_{19}ClN_8O_3$+Na]$^+$: 513.1166, found: 513.1165 (Δ = 0.2 ppm).

N-(1-{5-[4-(1H-Benzotriazole-1-carbonyl)phenoxy]pentyl}-3-[(6-chloropyridin-3-yl)methyl]imidazolidin-2-ylidene)nitramide (9c)

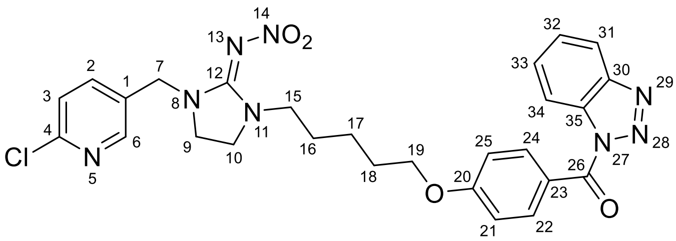

The product was synthesized following general procedure C using 4-({5-[3-[(6-chloropyridin-3-yl)methyl]-2-(nitroimino)imidazolidin-1-yl]pentyl}oxy)benzoic acid (**9b**) (0.400 g, 0.87 mmol, 1.00 eq.) and 1*H*-benzotriazole (0.363 g, 3.05 mmol, 3.52 eq.) in dry DCM (10 mL); thionyl chloride (0.129 g, 0.08 mL, 1.08 mmol, 1.25 eq.); 16 h at rt; water (20 mL); variant 1: DCM (3 x 50 mL), water (3 x 100 mL), column chromatography (ethyl acetate). The product was isolated as a colorless solid, yield: 0.376 g (0.67 mmol, 77%). m.p. (DSC): 137 °C. ^{1}H NMR (600 MHz, $CDCl_3$): δ = 8.34 (ddd, $J_{H,H}$ = 8.2, 1.0, 0.9 Hz, 1 H, H-34), 8.30 (d, $J_{H,H}$ = 2.4 Hz, 1 H, H-6), 8.25 (d, $J_{H,H}$ = 9.0 Hz, 2 H, H-22, H-24), 8.14 (ddd, $J_{H,H}$ = 8.2, 1.0, 0.9 Hz, 1 H, H-31), 7.69 (dd, $J_{H,H}$ = 8.3, 2.4 Hz, 1 H, H-2), 7.67 (ddd, $J_{H,H}$ = 8.2, 7.1, 1.0 Hz, 1 H, H-33), 7.52 (ddd, $J_{H,H}$ = 8.2, 7.1, 1.0 Hz, 1 H, H-32), 7.32 (d, $J_{H,H}$ = 8.3 Hz, 1 H, H-3), 7.02 (d, $J_{H,H}$ = 9.0 Hz, 2 H, H-21, H-25), 4.45 (s, 2 H, H-7), 4.07 (t, $J_{H,H}$ = 6.2 Hz, 2 H, H-19), 3.75 – 3.71 (m, 2 H, H-10), 3.59 – 3.56 (m, 2 H, H-9), 3.34 (t, $J_{H,H}$ = 7.6 Hz, 2 H, H-15), 1.87 – 1.83 (m, 2 H, H-18), 1.73 – 1.67 (m, 2 H, H-16), 1.54 – 1.49 (m, 2 H, H-17) ppm. ^{13}C NMR (151 MHz, $CDCl_3$): δ = 165.6 (o, 1 C, C-26), 163.5 (o, 1 C, C-20), 161.1 (o, 1 C, C-12), 151.8 (o, 1 C, C-4), 149.3 (+, 1 C, C-6), 145.6 (o, 1 C, C-30), 139.1 (+, 1 C, C-2), 134.3 (+, 2 C, C-22, C-24), 132.5 (o, 1 C, C-35), 130.1 (+, 1 C, C-33), 129.0 (o, 1 C, C-1), 126.1 (+, 1 C, C-32), 124.8 (+, 1 C, C-3), 123.3 (o, 1 C, C-23), 120.0 (+, 1 C, C-31), 114.7 (+, 1 C, C-34), 114.2 (+, 2 C, C-21, C-25), 67.8 (-, 1 C, C-19), 47.3 (-, 1 C, C-7), 46.7 (-, 1 C, C-15), 45.6 (-, 1 C, C-10), 45.0 (-, 1 C, C-9), 28.5 (-, 1 C, C-18), 26.4 (-, 1 C, C-16), 23.0 (-, 1 C, C-17) ppm. ^{15}N NMR (61 MHz, $CDCl_3$): δ = 2.2 (1 N, N-28), -13.2 (1 N, N-14), -23.1 (1 N, N-29), -72.1 (1 N, N-5), -125.3 (1 N, N-27), -148.6 (1 N, N-13), -277.0 (1 N, N-11), -281.3 (1 N, N-8) ppm. IR (ATR): $\tilde{\nu}$ = 3089, 3051, 2946, 2923, 2868, 1688, 1602, 1576, 1565, 1535, 1510, 1485, 1462, 1406, 1367, 1354, 1290, 1263, 1249, 1235, 1179, 1147, 1131, 1104, 1038, 1003, 933, 887, 841, 810, 775, 764, 750, 734, 689, 670, 636, 626, 581, 568, 500, 474, 433, 410 cm^{-1}. MS (ESI, +): *m/z* (%) = 585.3 (100) $[M+Na]^+$. HR-MS (ESI, +): calcd. for $[C_{27}H_{27}ClN_8O_4+Na]^+$: 585.1741, found: 585.1744 (Δ = 0.5 ppm).

_N_-(1-{5-[4-(1_H_-Benzotriazole-1-carbonyl)-2-chlorophenoxy]pentyl}-3-[(6-chloropyridin-3-yl)methyl]imidazolidin-2-ylidene)nitramide (10c)

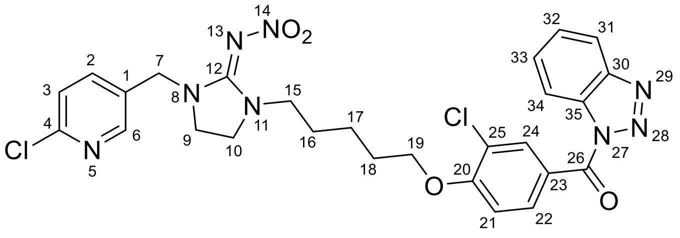

The product was synthesized following general procedure C using 3-chloro-4-({5-[3-[(6-chloropyridin-3-yl)methyl]-2-(nitroimino)imidazolidin-1-yl]pentyl}oxy)benzoic acid (**10b**) (0.865 g, 1.74 mmol, 1.00 eq.) and 1*H*-benzotriazole (0.731 g, 6.13 mmol, 3.52 eq.) in dry DCM (10 mL); thionyl chloride (0.301 g, 0.18 mL, 2.53 mmol, 1.45 eq.); 16 h at rt; water (20 mL); variant 1: DCM (3 x 50 mL), water (3 x 100 mL), column chromatography (ethyl acetate). The product was isolated as a colorless solid, yield: 0.908 g (1.52 mmol, 87%). m.p. (DSC): 167 °C, 195 °C (decomp.). ^{1}H NMR (600 MHz, DMSO-d_6): δ = 8.35 (dd, $J_{H,H}$ = 2.5, 0.6 Hz, 1 H, H-6), 8.30 (ddd, $J_{H,H}$ = 8.3, 0.9, 0.9 Hz, 1 H, H-34), 8.29 (ddd, $J_{H,H}$ = 8.2, 1.0, 0.9 Hz, 1 H, H-31), 8.24 (d, $J_{H,H}$ = 2.2 Hz, 1 H, H-24), 8.15 (dd, $J_{H,H}$ = 8.8, 2.2 Hz, 1 H, H-22), 7.83 (ddd, $J_{H,H}$ = 8.3, 7.1, 1.0 Hz, 1 H, H-33), 7.78 (dd, $J_{H,H}$ = 8.3, 2.5 Hz, 1 H, H-2), 7.65 (ddd, $J_{H,H}$ = 8.2, 7.1, 0.9 Hz, 1 H, H-32), 7.54 (dd, $J_{H,H}$ = 8.3, 0.6 Hz, 1 H, H-3), 7.41 (d, $J_{H,H}$ = 8.8 Hz, 1 H, H-21), 4.44 (s, 2 H, H-7), 4.24 (t, $J_{H,H}$ = 6.4 Hz, 2 H, H-19), 3.78 – 3.73 (m, 2 H, H-10), 3.66 – 3.63 (m, 2 H, H-9), 3.24 (t, $J_{H,H}$ = 7.3 Hz, 2 H, H-15), 1.85 – 1.80 (m, 2 H, H-18), 1.67 – 1.61 (m, 2 H, H-16), 1.48 – 1.41 (m, 2 H, H-17) ppm. ^{13}C NMR (151 MHz, DMSO-d_6): δ = 164.5 (o, 1 C, C-26), 160.7 (o, 1 C, C-12), 158.2 (o, 1 C, C-20), 149.7 (o, 1 C, C-4), 149.4 (+, 1 C, C-6), 145.1 (o, 1 C, C-30), 139.5 (+, 1 C, C-2), 133.2 (+, 1 C, C-24), 132.8 (+, 1 C, C-22), 131.9 (o, 1 C, C-35), 130.7 (+, 1 C, C-33), 130.6 (o, 1 C, C-1), 126.6 (+, 1 C, C-32), 124.3 (+, 1 C, C-3), 123.9 (o, 1 C, C-23), 121.2 (o, 1 C, C-25), 120.0 (+, 1 C, C-31), 114.5 (+, 1 C, C-34), 113.2 (+, 1 C, C-21), 69.0 (-, 1 C, C-19), 46.2 (-, 1 C, C-7), 45.6 (-, 1 C, C-15), 45.3 (-, 1 C, C-9), 45.2 (-, 1 C, C-10), 27.8 (-, 1 C, C-18), 25.7 (-, 1 C, C-16), 22.3 (-, 1 C, C-17) ppm. IR (ATR): $\tilde{\nu}$ = 3121, 3086, 3057, 2941, 2885, 2871, 2858, 1697, 1594, 1562, 1538, 1503, 1484, 1461, 1446, 1411, 1393, 1377, 1360, 1266, 1239, 1228, 1155, 1127, 1108, 1051, 1021, 999, 948, 894, 859, 832, 821, 782, 772, 761, 739, 707, 687, 657, 630, 601, 569, 553, 513, 491, 467, 428, 412 cm^{-1}. MS (ESI, +): *m/z* (%) = 619.1 (100) $[M+Na]^+$. HR-MS (ESI, +): calcd. for $[C_{27}H_{26}Cl_2N_8O_4+Na]^+$: 619.1352, found: 619.1354 (Δ = 0.3 ppm).

_N_-(1-{5-[4-(1_H_-Benzotriazole-1-carbonyl)-2-nitrophenoxy]pentyl}-3-[(6-chloropyridin-3-yl)methyl]imidazolidin-2-ylidene)nitramide (11c)

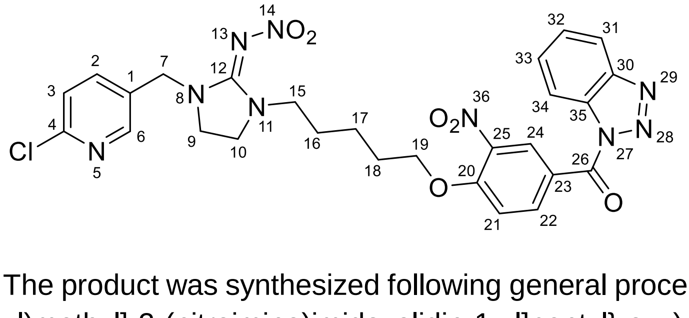

The product was synthesized following general procedure C using 4-({5-[3-[(6-chloropyridin-3-yl)methyl]-2-(nitroimino)imidazolidin-1-yl]pentyl}-oxy)-3-nitrobenzoic acid (**11b**) (0.597 g, 1.18 mmol, 1.00 eq.) and 1*H*-benzotriazole (0.494 g, 4.15 mmol, 3.52 eq.) in dry DCM (10 mL);

thionyl chloride (0.175 g, 0.11 mL, 1.47 mmol, 1.25 eq.); 16 h at rt; saturated aq. solution of NH_4Cl (20 mL); variant 2: DCM (3 x 50 mL), water (7 x 100 mL). The product was isolated as a beige solid, yield: 0.544 g (0.90 mmol, 76%). m.p. (DSC): 207 °C (decomp.). ^{1}H NMR (600 MHz, $CDCl_3$): δ = 8.84 (d, $J_{H,H}$ = 2.3 Hz, 1 H, H-24), 8.55 (dd, $J_{H,H}$ = 8.9, 2.3 Hz, 1 H, H-22), 8.37 (ddd, $J_{H,H}$ = 8.3, 1.0, 0.9 Hz, 1 H, H-34), 8.31 (d, $J_{H,H}$ = 2.4 Hz, 1 H, H-6), 8.17 (ddd, $J_{H,H}$ = 8.2, 1.0, 0.9 Hz, 1 H, H-31), 7.73 (ddd, $J_{H,H}$ = 8.3, 7.2, 1.0 Hz, 1 H, H-33), 7.71 (dd, $J_{H,H}$ = 8.2, 2.4 Hz, 1 H, H-2), 7.57 (ddd, $J_{H,H}$ = 8.2, 7.2, 1.0 Hz, 1 H, H-32), 7.34 (d, $J_{H,H}$ = 8.2 Hz, 1 H, H-3), 7.26 (d, $J_{H,H}$ = 8.9 Hz, 1 H, H-21), 4.47 (s, 2 H, H-7), 4.27 (t, $J_{H,H}$ = 5.8 Hz, 2 H, H-19), 3.79 – 3.76 (m, 2 H, H-10), 3.64 – 3.61 (m, 2 H, H-9), 3.39 (t, $J_{H,H}$ = 7.2 Hz, 2 H, H-15), 1.94 – 1.90 (m, 2 H, H-18), 1.76 – 1.71 (m, 2 H, H-16), 1.62 – 1.57 (m, 2 H, H-17) ppm. ^{13}C NMR (151 MHz, $CDCl_3$): δ = 163.3 (o, 1 C, C-26), 161.0 (o, 1 C, C-12), 155.8 (o, 1 C, C-20), 151.2 (o, 1 C, C-4), 149.1 (+, 1 C, C-6), 145.3 (o, 1 C, C-30), 138.9 (+, 1 C, C-2), 138.9 (o, 1 C, C-25), 137.7 (+, 1 C, C-22), 131.9 (o, 1 C, C-35), 130.5 (+, 1 C, C-33), 129.5 (+, 1 C, C-24), 129.0 (o, 1 C, C-1), 126.4 (+, 1 C, C-32), 124.5 (+, 1 C, C-3), 122.8 (o, 1 C, C-23), 119.9 (+, 1 C, C-31), 114.5 (+, 1 C, C-34), 113.9 (+, 1 C, C-21), 69.6 (-, 1 C, C-19), 47.0 (-, 1 C, C-7), 46.1 (-, 1 C, C-15), 45.3 (-, 1 C, C-10), 45.0 (-, 1 C, C-9), 27.8 (-, 1 C, C-18), 25.9 (-, 1 C, C-16), 22.6 (-, 1 C, C-17) ppm. ^{15}N NMR (61 MHz, $CDCl_3$): δ = 2.9 (1 N, N-28), -9.4 (1 N, N-36), -10.7 (1 N, N-14), -17.6 (1 N, N-29), -70.0 (1 N, N-5), -123.8 (1 N, N-27), -146.7 (1 N, N-13), -274.1 (1 N, N-11), -278.1 (1 N, N-8) ppm. IR (ATR): $\tilde{\nu}$ = 3079, 2932, 2864, 1699, 1612, 1562, 1530, 1484, 1449, 1419, 1358, 1254, 1224, 1167, 1149, 1134, 1106, 1083, 1045, 1026, 1003, 972, 953, 914, 859, 846, 825, 780, 771, 752, 693, 666, 655, 630, 565, 494, 428, 420, 411 cm^{-1}. MS (ESI, +): *m/z* (%) = 630.2 (100) [M+Na]$^+$. HR-MS (ESI, +): calcd. for $[C_{27}H_{26}ClN_9O_6+Na]^+$: 630.1592, found: 630.1583 (Δ = 1.4 ppm).

(1*H*-Benzotriazol-1-yl)(4-{[1,1-dichloro-3-{1-[(6-chloropyridin-3-yl)methyl]imidazolidin-2-ylidene}-3-nitroprop-1-en-2-yl]sulfanyl}phenyl)methanone (12c)

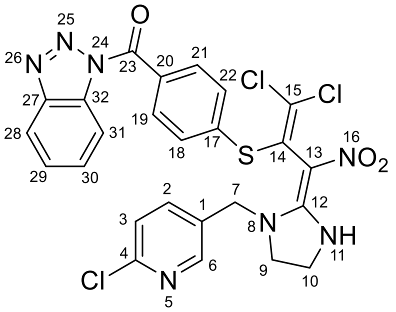

The procedure is based on a method mentioned by SHEIKH.[25] *N*,*N*'-dicyclohexylmethanediimine (0.238 g, 1.15 mmol, 1.20 eq.) was added to a solution of 4-{[1,1-dichloro-3-{1-[(6-chloropyridin-3-yl)methyl]imidazolidin-2-ylidene}-3-nitro-prop-1-en-2-yl]sulfanyl}benzoic acid (**12b**) (0.482 g, 0.96 mmol, 1.00 eq.) and 1*H*-benzotriazole (0.126 g, 1.06 mmol, 1.10 eq.) in dry DCM (30 mL) under nitrogen atmosphere. The mixture was stirred for 16 h at rt. Subsequently, water (30 mL) was added, the suspension was filtered and the filtrate extracted with DCM (3 x 50 mL). The combined organic phases were washed with water (10 x 100 mL) and dried over magnesium sulfate. The solution was concentrated to one third of its original volume and stored overnight in a freezer. The solution was filtered to remove by-products and the filtrate was concentrated to dryness. The product was dried *in vacuo* and isolated as a pale orange solid, yield: 0.205 g (0.34 mmol, 35%). m.p. (DSC): 175 °C (decomp.). ^{1}H NMR (600 MHz, DMSO-d_6): δ = 9.00 (s, 1 H, NH-11), 8.34 (ddd, $J_{H,H}$ = 8.2, 1.0, 0.9 Hz, 1 H, H-31), 8.32 (dd, $J_{H,H}$ = 2.6, 0.5 Hz, 1 H, H-6), 8.31 (ddd, $J_{H,H}$ = 8.2, 1.0, 0.9 Hz,

1 H, H-28), 8.10 (d, $J_{H,H}$ = 8.6 Hz, 2 H, H-19, H-21), 7.85 (ddd, $J_{H,H}$ = 8.2, 7.2, 1.0 Hz, 1 H, H-30), 7.74 (dd, $J_{H,H}$ = 8.3, 2.6 Hz, 1 H, H-2), 7.67 (ddd, $J_{H,H}$ = 8.2, 7.2, 1.0 Hz, 1 H, H-29), 7.64 (d, $J_{H,H}$ = 8.6 Hz, 2 H, H-18, H-22), 7.53 (dd, $J_{H,H}$ = 8.3, 0.5 Hz, 1 H, H-3), 4.43 (bs, 2 H, H-7), 3.61 – 3.58 (m, 2 H, H-9), 3.56 – 3.53 (m, 2 H, H-10) ppm. ^{13}C NMR (151 MHz, DMSO-d_6): δ = 165.9 (o, 1 C, C-23), 159.4 (o, 1 C, C-12), 149.4 (o, 1 C, C-4), 148.4 (+, 1 C, C-6), 145.2 (o, 1 C, C-27), 138.4 (+, 1 C, C-2), 137.3 (o, 1 C, C-17), 132.0 (+, 2 C, C-18, C-22), 131.8 (+, 2 C, C-19, C-21), 131.7 (o, 1 C, C-32), 131.4 (o, 1 C, C-20), 131.1 (o, 1 C, C-1), 130.8 (+, 1 C, C-30), 129.2 (o, 1 C, C-14), 126.7 (+, 1 C, C-29), 124.2 (+, 1 C, C-3), 120.7 (o, 1 C, C-15), 120.1 (+, 1 C, C-28), 114.5 (+, 1 C, C-31), 103.2 (o, 1 C, C-13), 49.8 (-, 1 C, C-9), 48.6 (-, 1 C, C-7), 41.8 (-, 1 C, C-10) ppm. IR (ATR): $\tilde{\nu}$ = 3311, 3071, 2926, 2902, 2851, 1704, 1626, 1557, 1520, 1483, 1460, 1450, 1435, 1419, 1394, 1362, 1320, 1287, 1262, 1244, 1228, 1206, 1185, 1148, 1124, 1103, 1039, 1005, 984, 952, 935, 887, 843, 820, 779, 770, 746, 689, 646, 628, 591, 568, 550, 503, 493, 429, 410 cm^{-1}. MS (ESI, +): *m/z* (%) = 624.0 (100) [M+Na]$^+$. HR-MS (ESI, +): calcd. for [$C_{25}H_{18}Cl_3N_7O_3S$+Na]$^+$: 624.0155, found: 624.0158 (Δ= 0.5 ppm).

3. Wood modifications

The following table S1 presents the detailed results of the modification of the wood chips **7d – 12d**. In addition, table S2 shows the corresponding results of the modification of the standardized wood blocks **8d'** for the biological tests.

Table S1. Results of the modification of the wood chips **7d – 12d**.

modification	temperature [°C]	sample	sample mass [g] (unmodified)	sample mass [g] (modified)	mass difference [g]	WPG [%]	QCO [mmol g^{-1}]
7d	70	a	0.0597	0.0643	0.0046	7.7	0.26
7d	70	b	0.0580	0.0617	0.0037	6.4	0.22
7d	70	c	0.0507	0.0545	0.0038	7.5	0.25
**Ø 7d**	**70**					**7.2**	**0.24**
**σ 7d**	**70**					**0.7**	**0.02**
7d	120	a	0.0543	0.0550	0.0007	1.3	0.04
7d	120	b	0.0533	0.0546	0.0013	2.4	0.08
**Ø 7d**	**120**					**1.9**	**0.06**
**σ 7d**	**120**					**0.8**	**0.03**
8d	70	a	0.0697	0.0777	0.0080	11.5	0.31
8d	70	b	0.0584	0.0631	0.0047	8.0	0.22
8d	70	c	0.056	0.0615	0.0055	9.8	0.26
**Ø 8d**	**70**					**9.8**	**0.26**
**σ 8d**	**70**					**1.7**	**0.05**
8d	120	a	0.0544	0.0644	0.0100	18.4	0.49
8d	120	b	0.0580	0.0697	0.0117	20.2	0.54
**Ø 8d**	**120**					**19.3**	**0.52**
**σ 8d**	**120**					**1.3**	**0.03**
9d	70	a	0.0506	0.0528	0.0022	4.3	0.10
**Ø 9d**	**70**					**4.3**	**0.10**
**σ 9d**	**70**					**n/a**	**n/a**
9d	120	a	0.0510	0.0558	0.0048	9.4	0.21
**Ø 9d**	**120**					**9.4**	**0.21**
**σ 9d**	**120**					**n/a**	**n/a**
10d	70	a	0.0613	0.0673	0.0060	9.8	0.20
10d	70	b	0.0622	0.0684	0.0062	10.0	0.21
**Ø 10d**	**70**					**9.9**	**0.21**
**σ 10d**	**70**					**0.1**	**0.00**
10d	120	a	0.0702	0.0873	0.0171	24.4	0.51
10d	120	b	0.0655	0.0788	0.0133	20.3	0.42
**Ø 10d**	**120**					**22.3**	**2.9**
**σ 10d**	**120**					**0.47**	**0.06**
11d	70	a	0.0536	0.0612	0.0076	14.2	0.29
**Ø 11d**	**70**					**14.2**	**0.29**
**σ 11d**	**70**					**n/a**	**n/a**
11d	120	a	0.0512	0.0611	0.0099	19.3	0.40
**Ø 11d**	**120**					**19.3**	**0.40**
**σ 11d**	**120**					**n/a**	**n/a**
12d	70	a	0.0443	0.0475	0.0032	7.2	0.15
**Ø 12d**	**70**					**7.2**	**0.15**
**σ 12d**	**70**					**n/a**	**n/a**
12d	120	a	0.0386	0.0415	0.0029	7.5	0.16
**Ø 12d**	**120**					**n/a**	**n/a**
**σ 12d**	**120**					**0.0**	**0.00**

Table S2. Results of the modification of the standardized wood blocks **8d'** for the biological tests.

modification	temperature [°C]	sample	sample mass [g] (unmodified)	sample mass [g] (modified)	mass difference [g]	WPG [%]	QCO [mmol g^{-1}]
8d'	70	a	8.0656	8.6356	0.5700	7.1	0.19
8d'	70	b	8.1325	8.6591	0.5266	6.5	0.17
8d'	70	c	8.1090	8.6308	0.5218	6.4	0.17
8d'	70	d	7.9173	8.4537	0.5364	6.8	0.18
8d'	70	e	7.9676	8.4910	0.5234	6.6	0.18
**Ø 8d'**	**70**					**6.7**	**0.18**
**σ 8d'**	**70**					**0.3**	**0.01**

In addition to Fig.1 in the main document, Fig. S1 shows the complete range of the ATR-IR spectra of the samples **7d** – **12d**. Adjacent to the characteristic bands resulting from the covalent modification, changes in the intensity of the OH bands are visible at 3340 cm^{-1}.

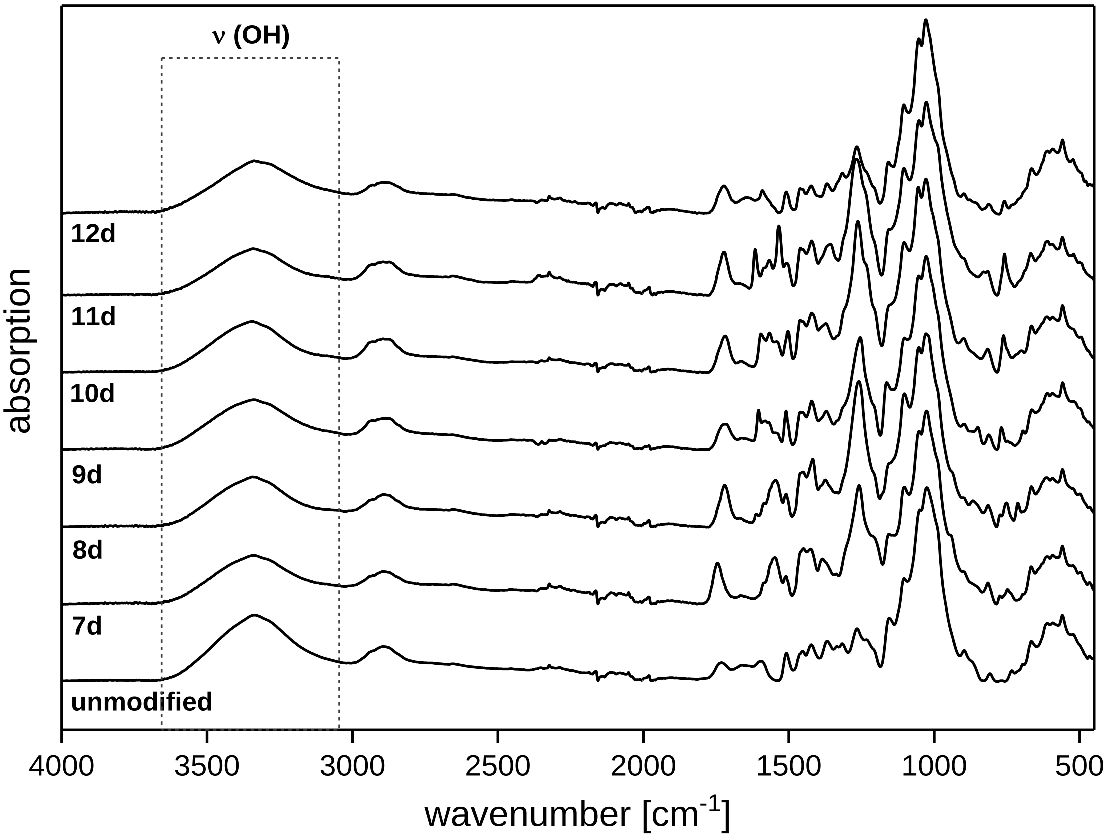

Fig. S1. Entire ATR-IR spectra of unmodified and modified wood in comparison.

4. Biological tests

The following tables S3 – S5 show the detailed results of the biological tests following DIN EN 46-1 of the modified wood samples **8d'** as well as the reference samples I and II against the larvae of the domestic house longhorn beetle.[1]

Table S3. Results of the biological tests of the modified samples **8d'**.

		after 4 weeks			after 12 weeks at the end of the test					
		larvae	dead larvae (surface)	mortality rate [%] (surface)	larvae found	larvae not found	living larvae (gnawed in)	dead larvae (gnawed in)	dead larvae (gnawed in + surface)	mortality rate [%] (final)
**sample 8d'**	block I	10	6	60.0	9	1	0	3	9	100.0
	block II	10	2	20.0	10	0	0	8	10	100.0
	block III	10	9	90.0	9	1	0	0	9	100.0
	block IV	10	7	70.0	10	0	0	3	10	100.0
	block V	10	9	90.0	10	0	0	1	10	100.0
	**Ø mortality [%]**			**66.0**						**100.0**
	**σ mortality [%]**			**28.8**						**0.0**

Table S4. Results of the biological tests of the reference samples I.

		after 4 weeks			after 12 weeks at the end of the test					
		larvae	dead larvae (surface)	mortality rate [%] (surface)	larvae found	larvae not found	living larvae (gnawed in)	dead larvae (gnawed in)	dead larvae (gnawed in + surface)	mortality rate [%] (final)
**reference sample I**	block I	10	0	0.0	9	1	7	2	2	22.2
	block II	10	0	0.0	8	2	7	1	1	12.5
	block III	10	0	0.0	8	2	8	0	0	0.0
	block IV	10	1	10.0	10	0	8	1	2	20.0
	block V	10	0	0.0	9	1	9	0	0	0.0
	**Ø mortality [%]**			**2.0**						**10.9**
	**σ mortality [%]**			**4.5**						**10.6**

Table S5. Results of the biological tests of the reference samples II.

		after 4 weeks			after 12 weeks at the end of the test					
		larvae	dead larvae (surface)	mortality rate [%] (surface)	larvae found	larvae not found	living larvae (gnawed in)	dead larvae (gnawed in)	dead larvae (gnawed in + surface)	mortality rate [%] (final)
**reference sample II**	block I	10	0	0.0	7	3	6	1	1	14.3
	block II	10	0	0.0	9	1	9	0	0	0.0
	block III	10	0	0.0	8	2	8	0	0	0.0
	**Ø mortality [%]**			**0.0**						**4.8**
	**σ mortality [%]**			**0.0**						**8.2**

5. <u>NMR spectra</u>

All NMR spectra are shown in the following.

_N_-Imidazolidin-2-ylidenenitramide (3):

^{1}H NMR – 400 MHz

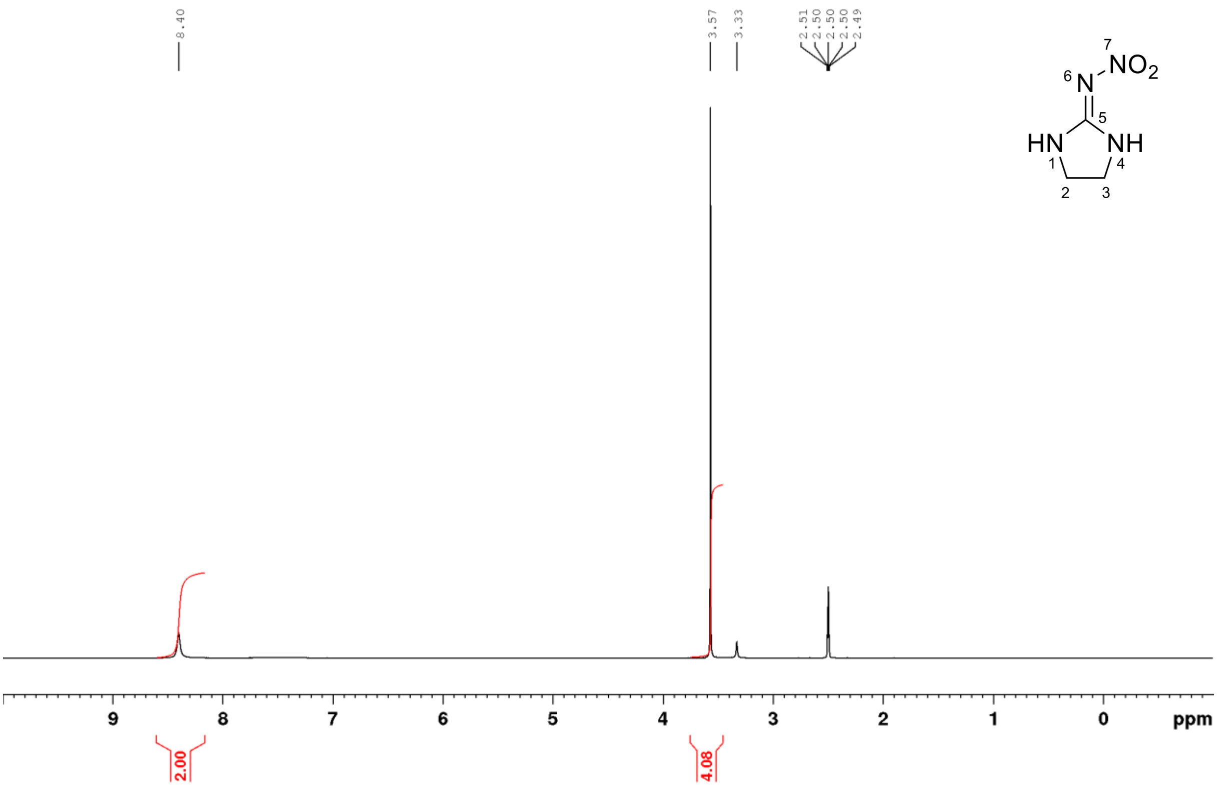

^{13}C NMR – 100 MHz

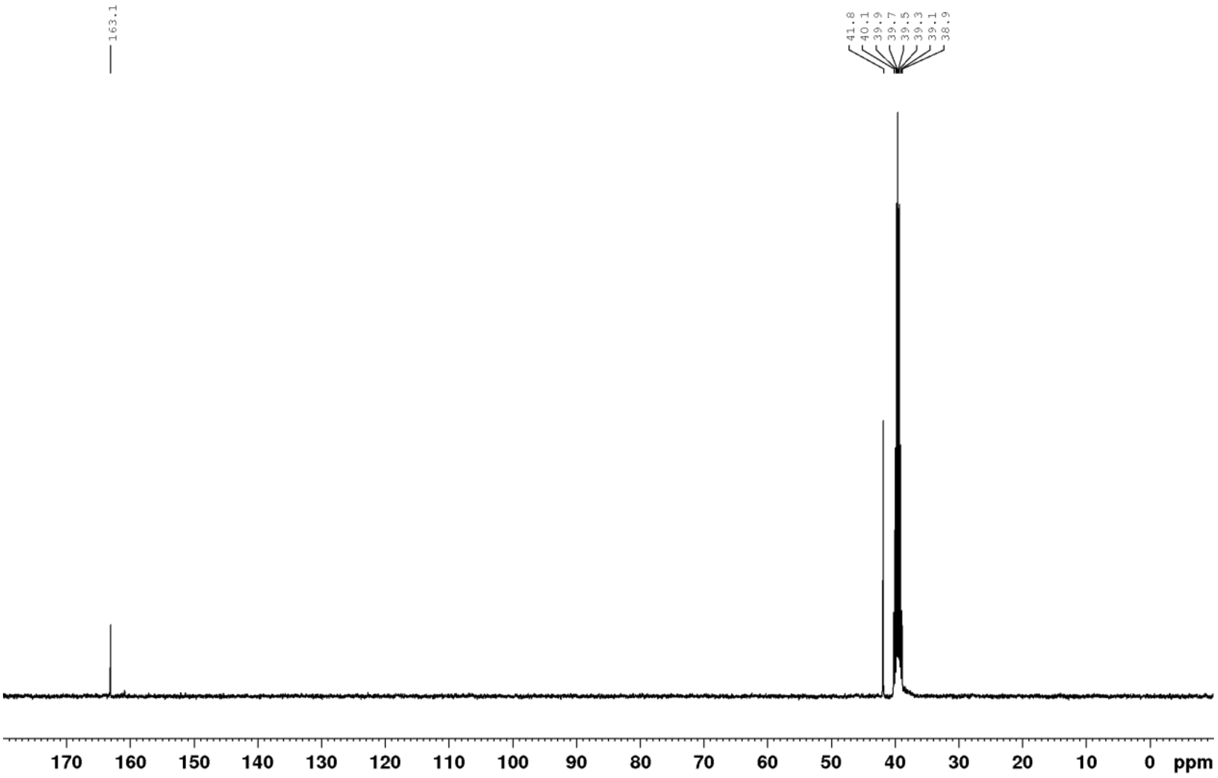

***N*¹-[(6-Chloropyridin-3-yl)methyl]ethane-1,2-diamine (6)**

^{1}H NMR – 400 MHz

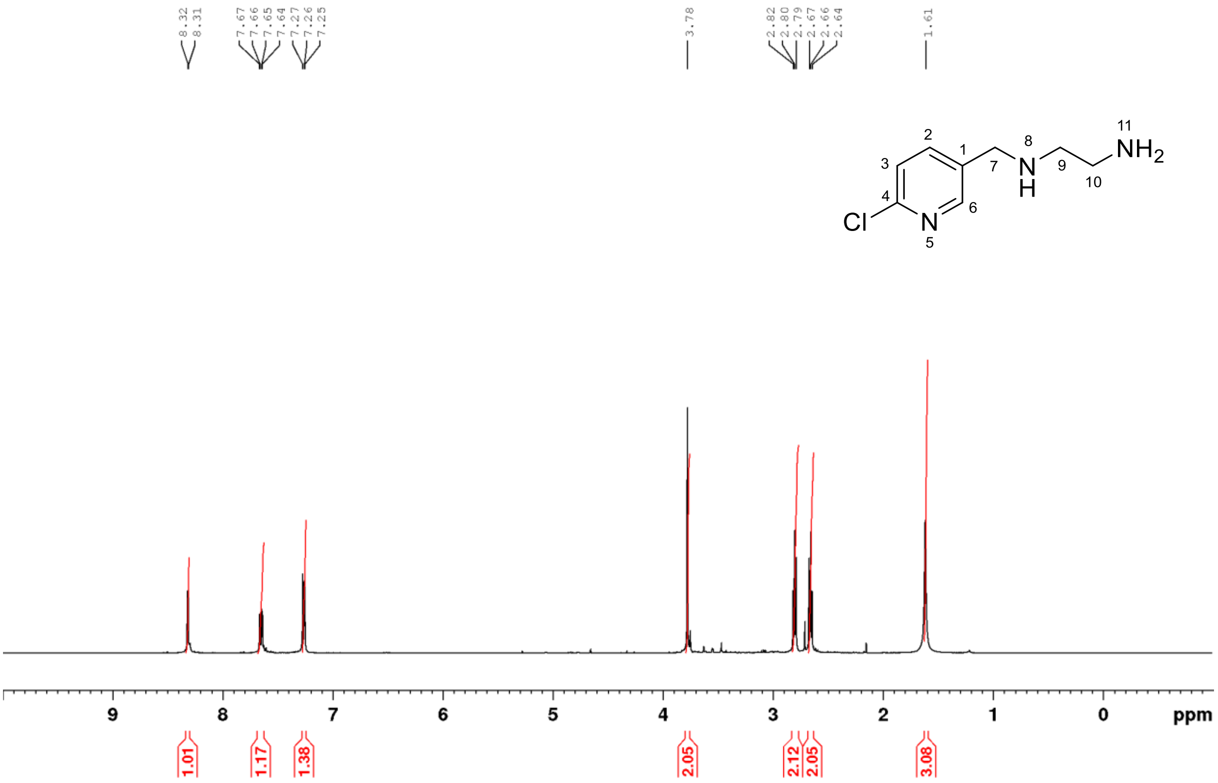

^{13}C NMR – 100 MHz

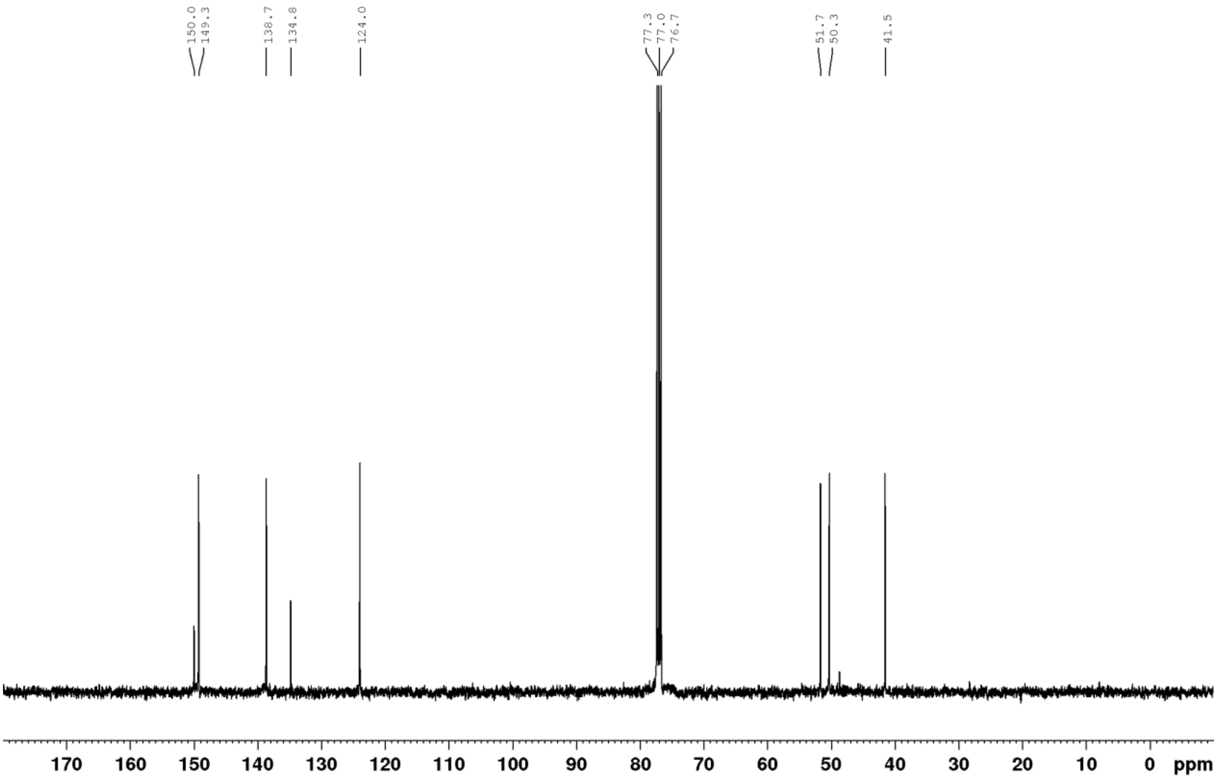

_N_-{(2_E_)-1-[(6-Chloropyridin-3-yl)methyl]imidazolidin-2-ylidene}nitramide (1)

^{1}H NMR – 600 MHz

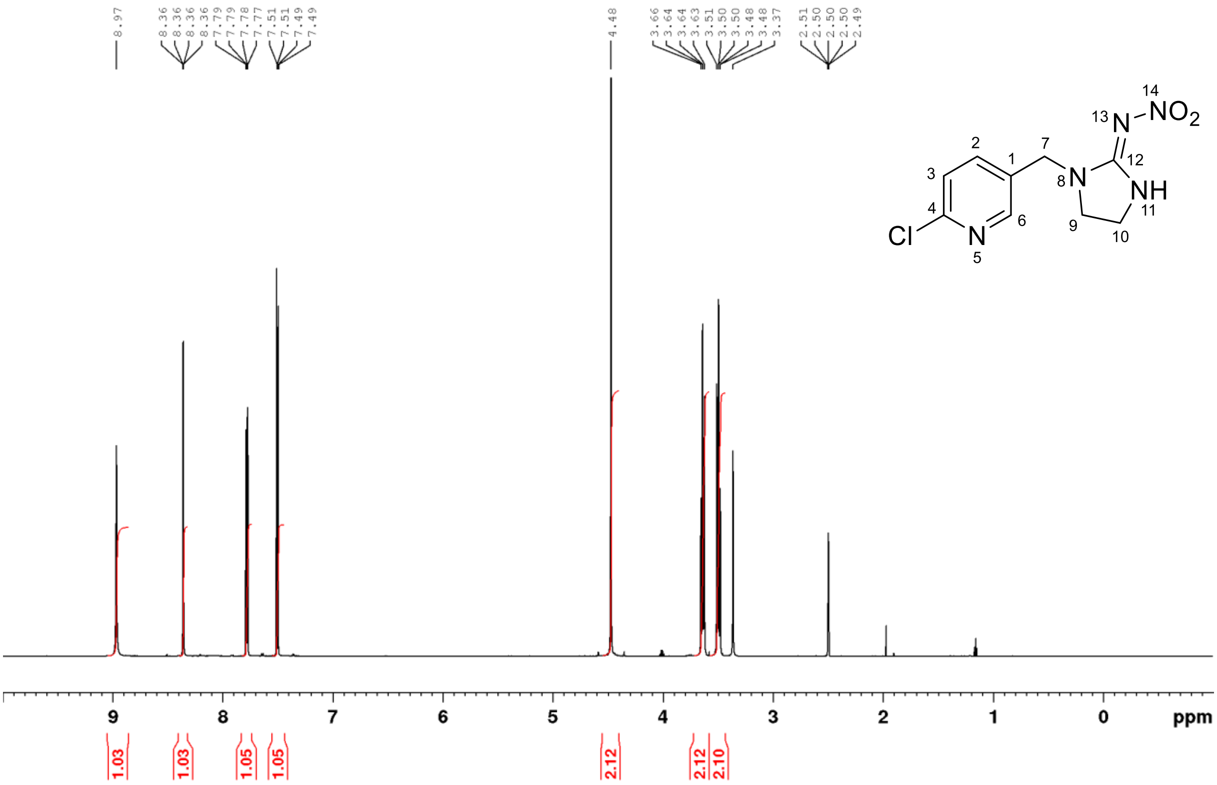

^{13}C NMR – 150 MHz

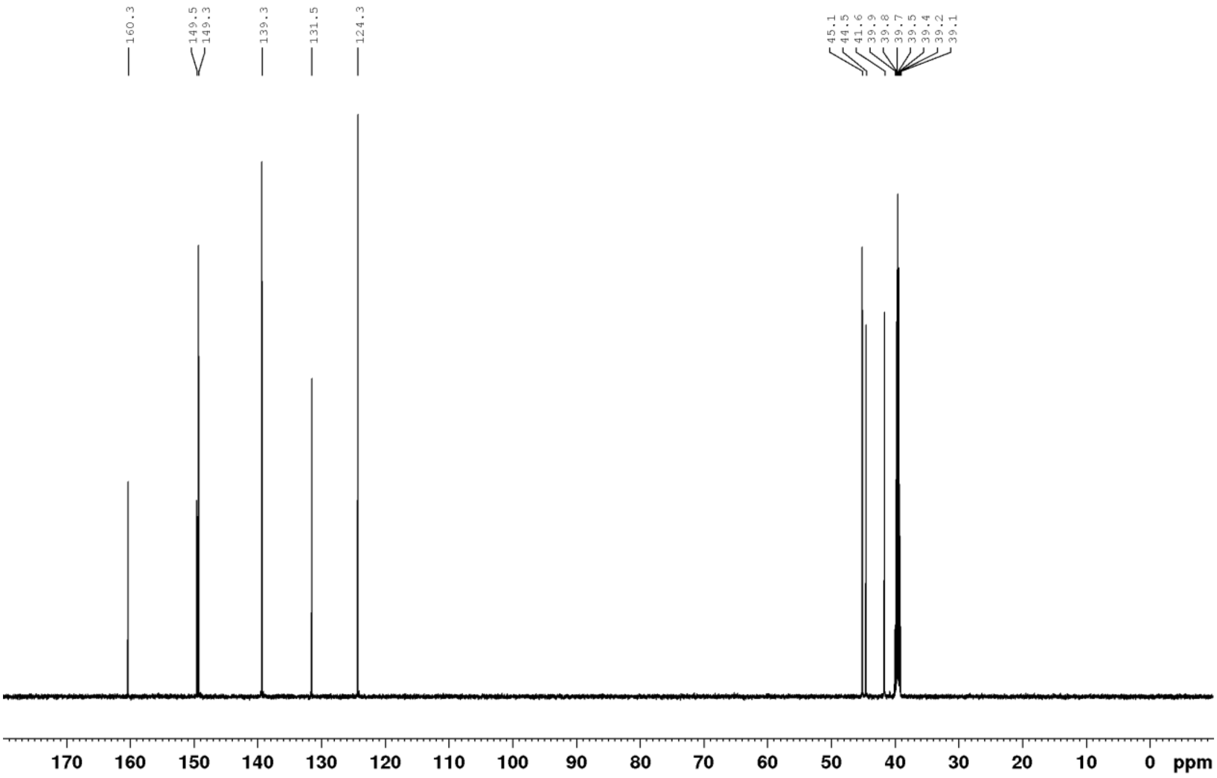

^{15}N NMR – 61 MHz

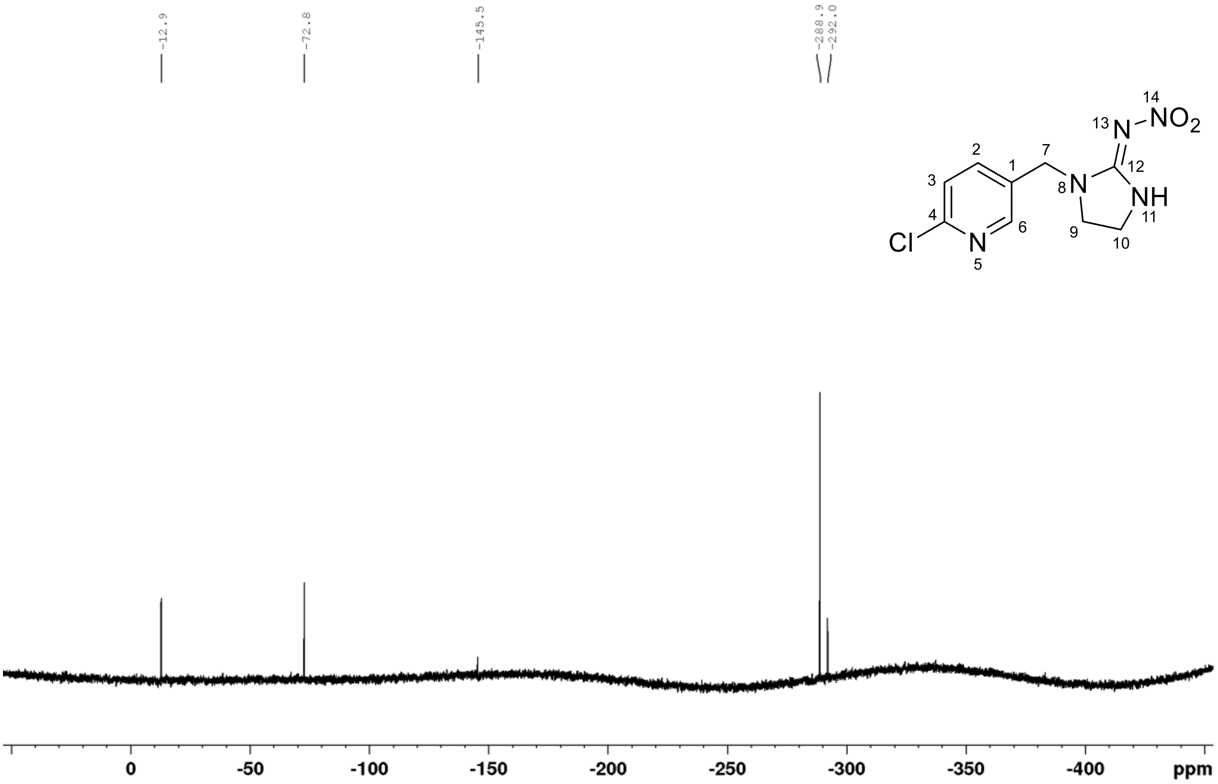

2-Chloro-5-{[(2*E*)-2-(2,3,3-trichloro-1-nitroprop-2-en-1-ylidene)imidazolidin-1-yl]methyl}pyridine (4)

^{1}H NMR – 400 MHz

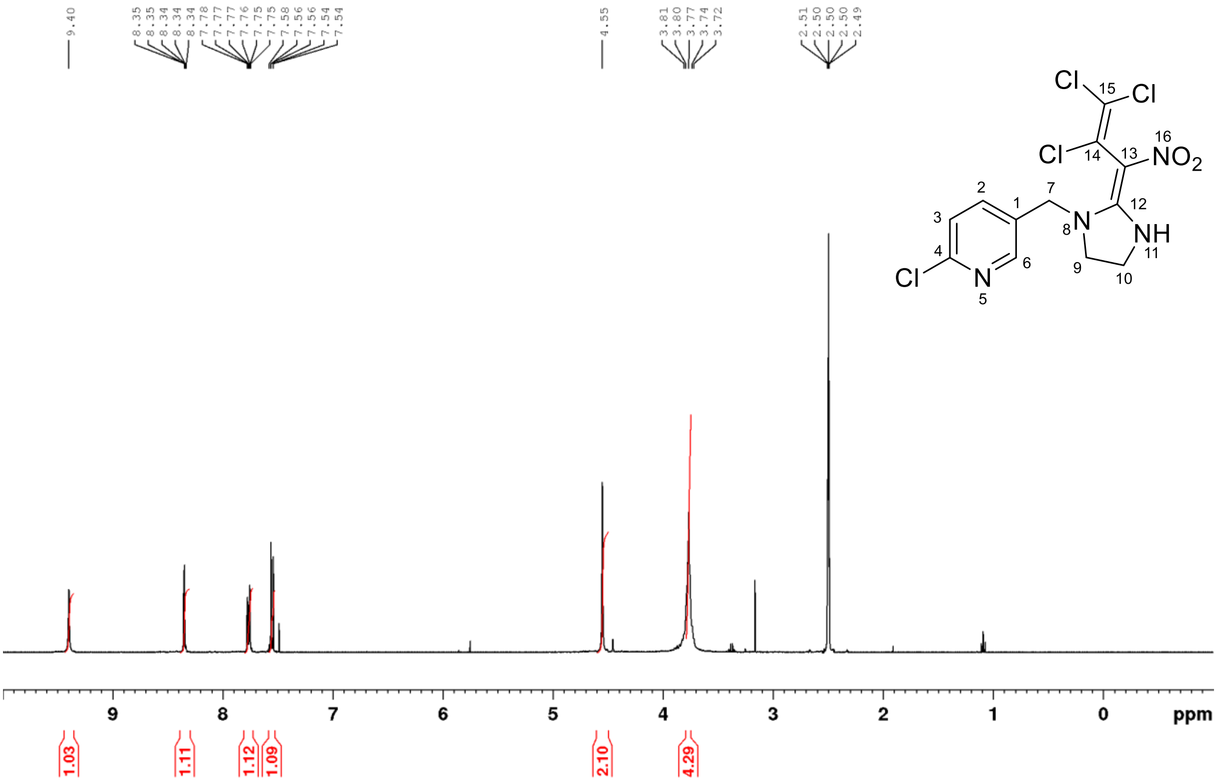

^{13}C NMR – 100 MHz

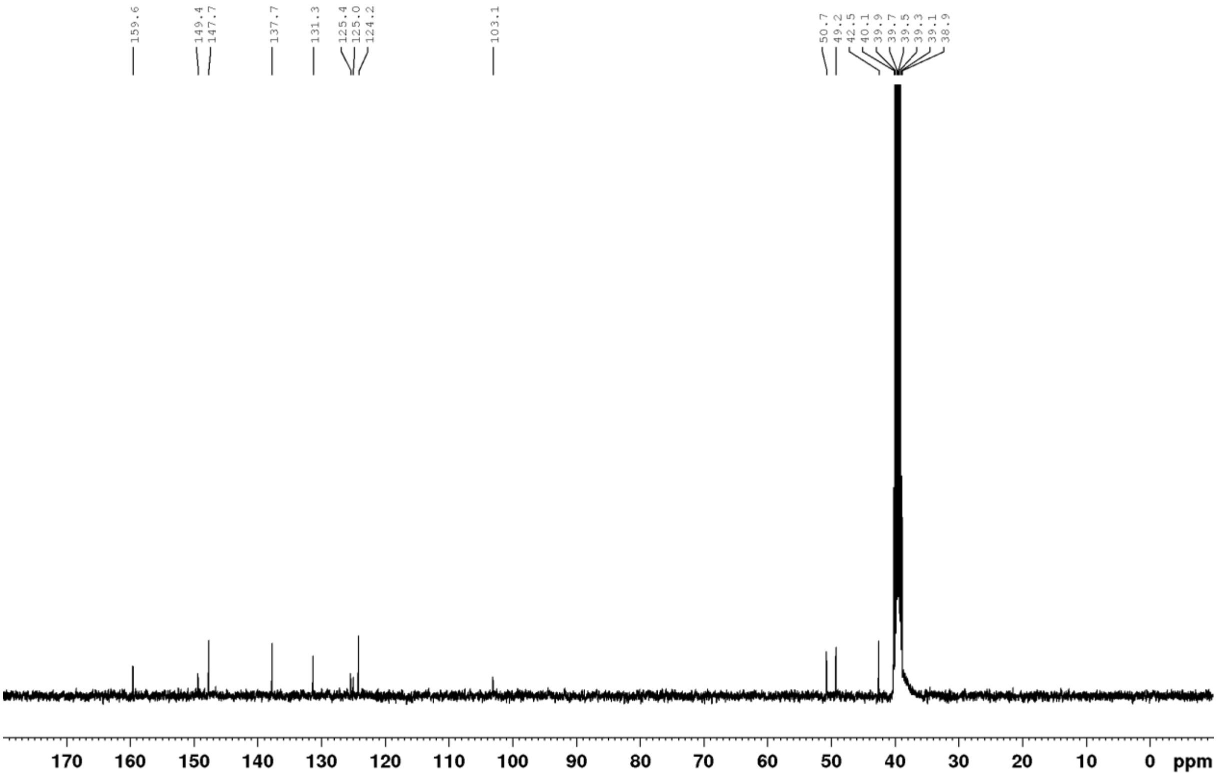

***tert*-Butyl 4-methylbenzoate (14)**

^{1}H NMR – 400 MHz

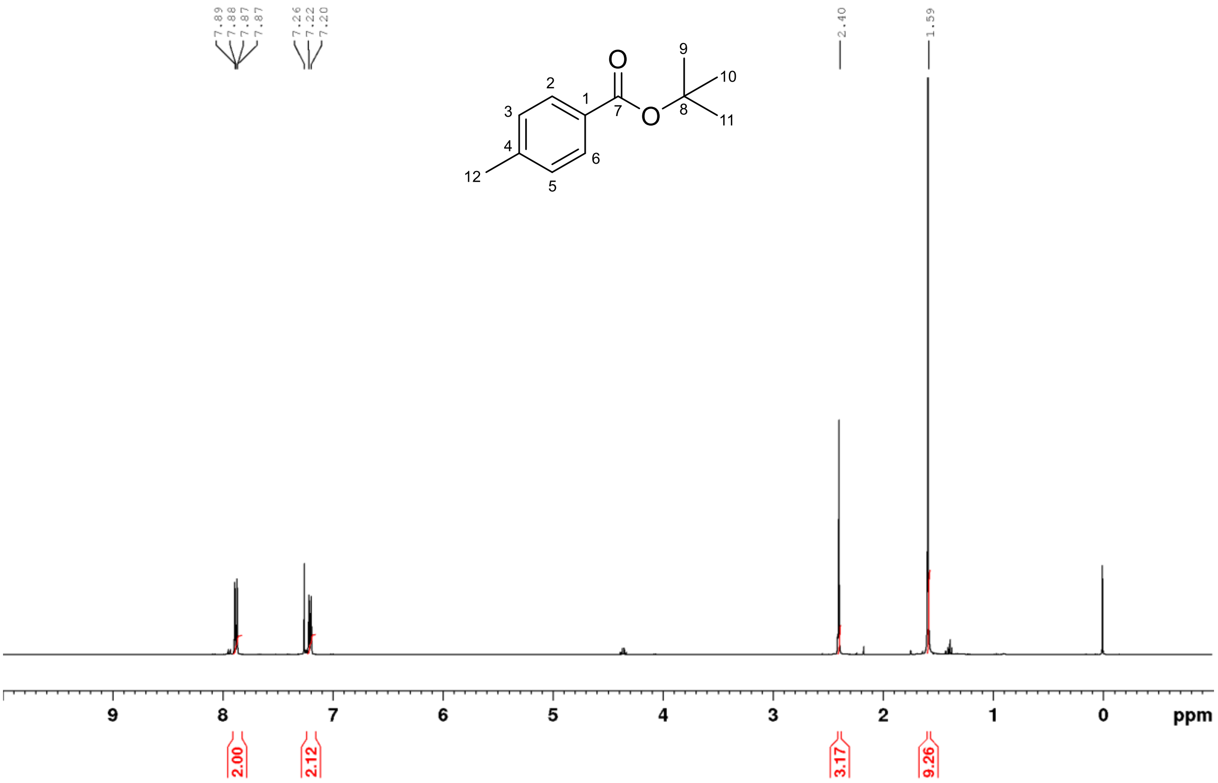

^{13}C NMR – 100 MHz

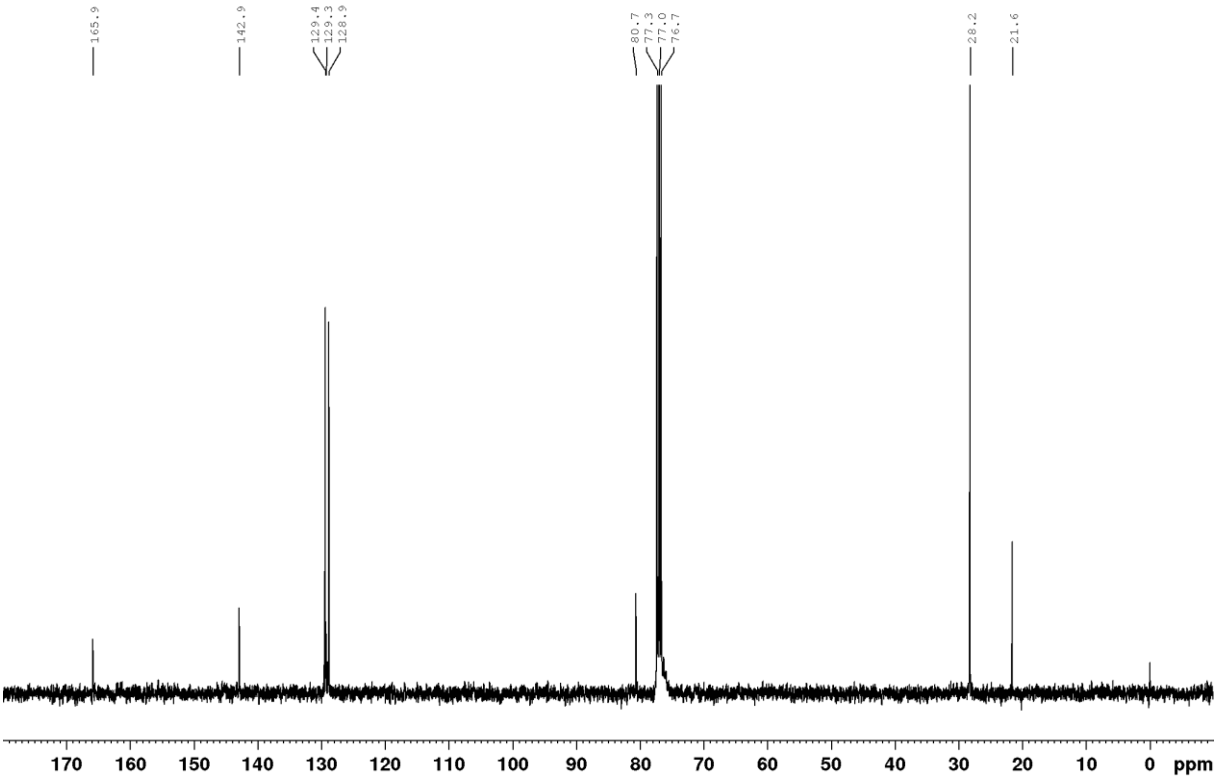

tert-Butyl 4-(bromomethyl)benzoate (8)

^{1}H NMR – 400 MHz

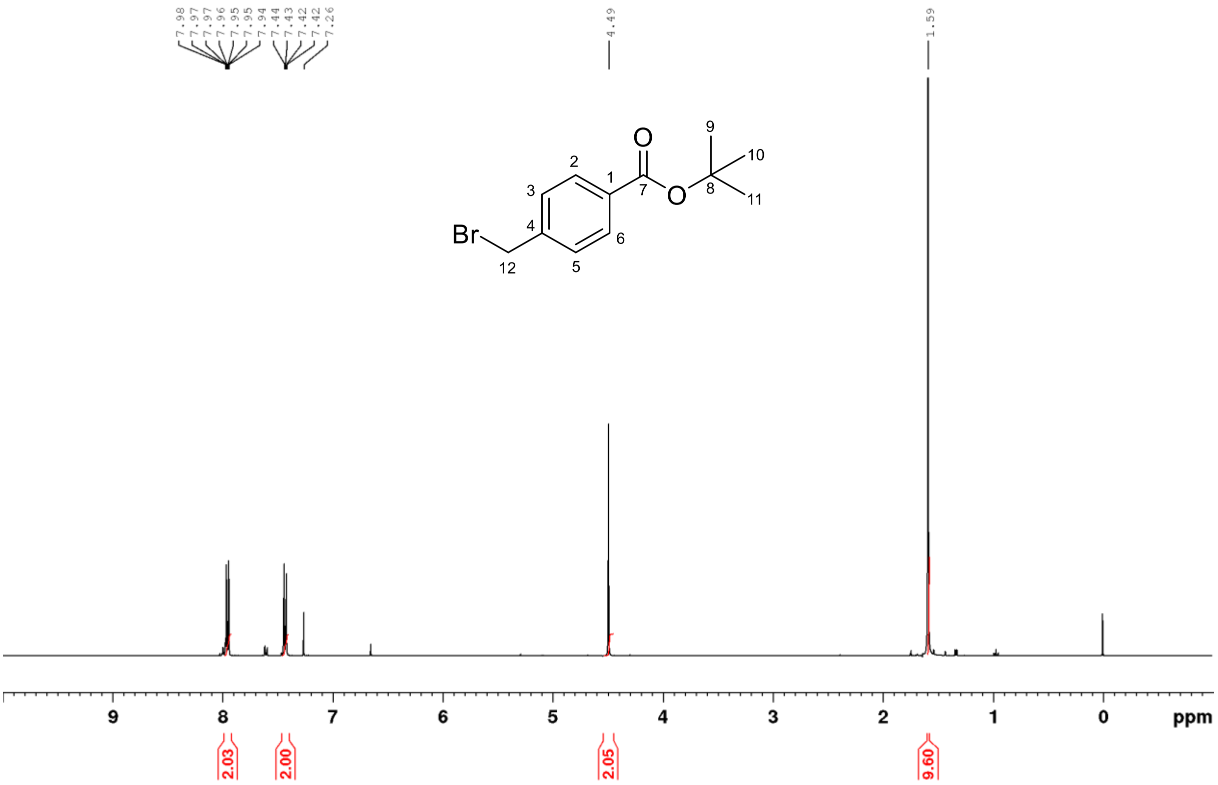

^{13}C NMR – 100 MHz

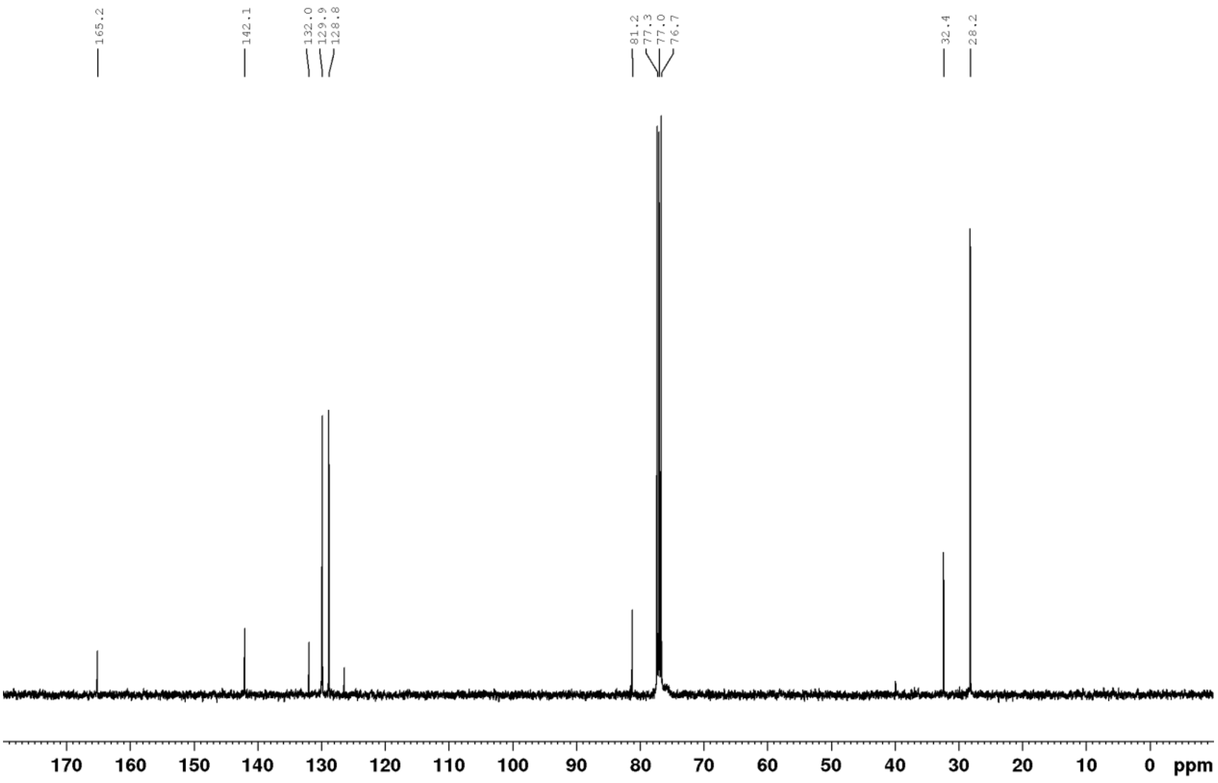

***tert*-Butyl 4-hydroxybenzoate (18)**

^{1}H NMR – 400 MHz

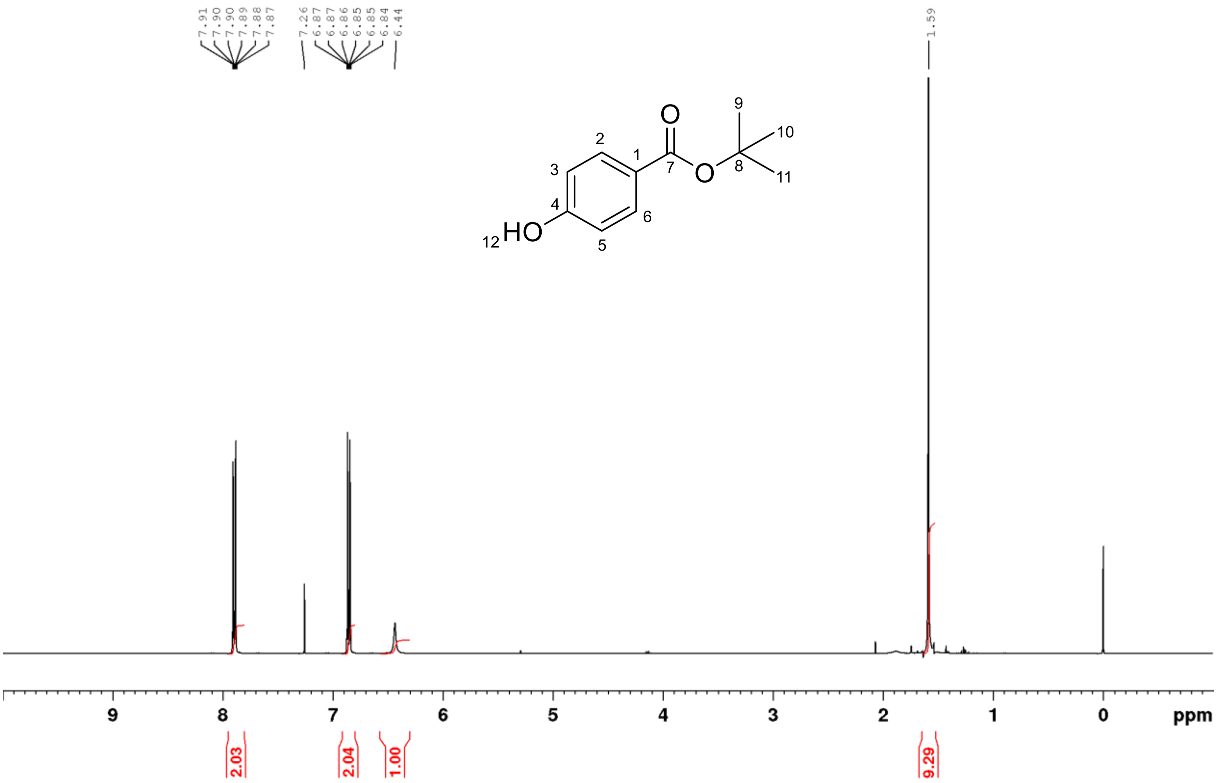

^{13}C NMR – 100 MHz

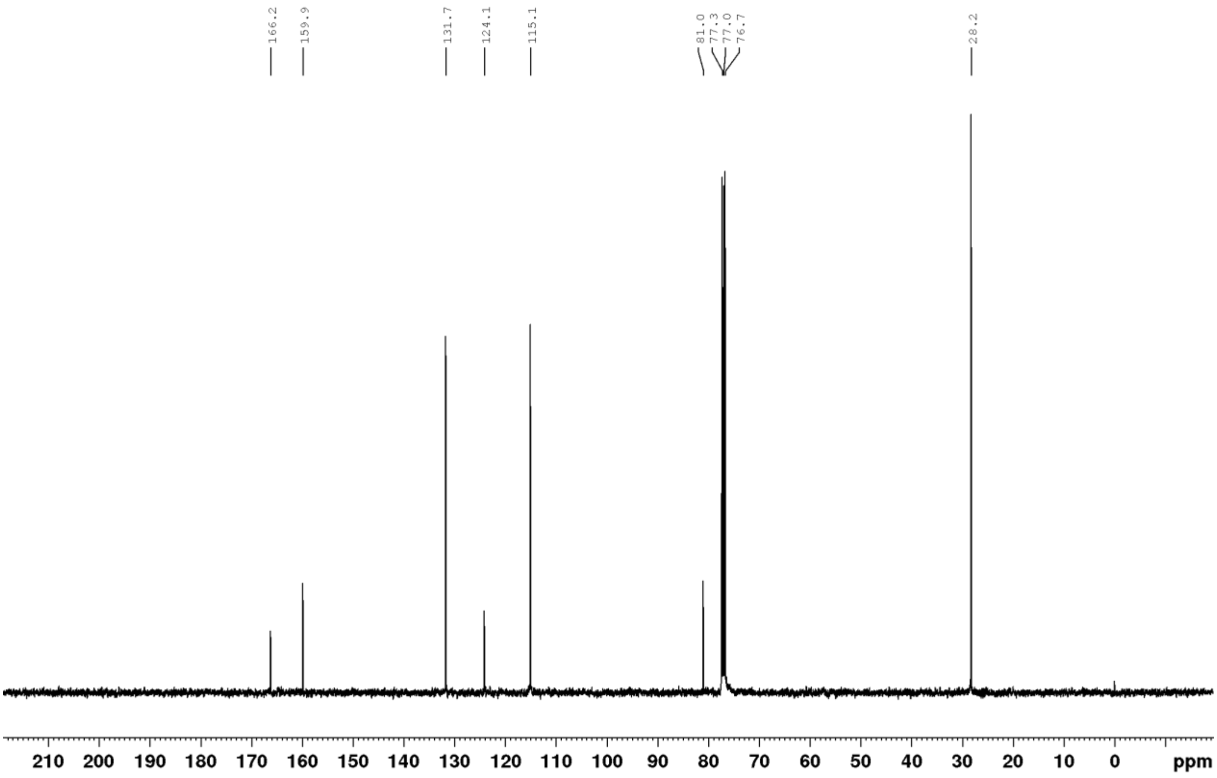

tert-Butyl 3-chloro-4-hydroxybenzoate (19)

^{1}H NMR – 400 MHz

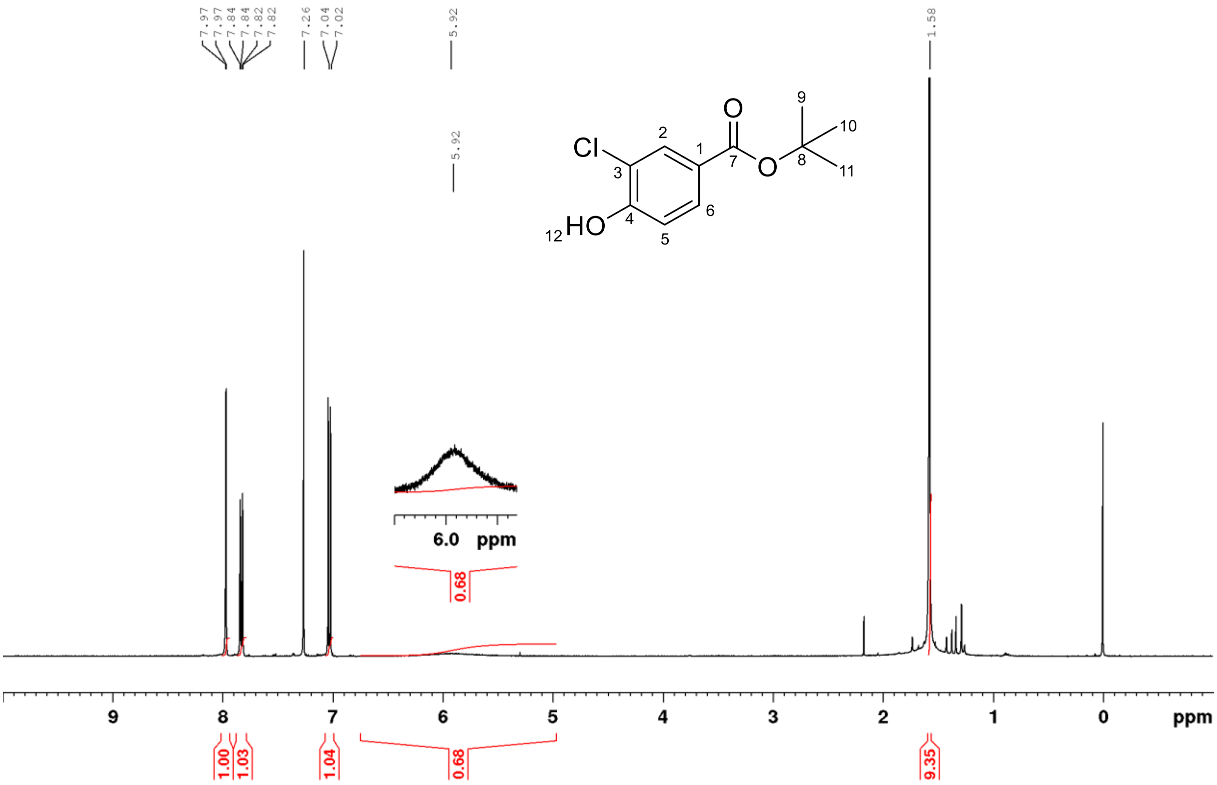

^{13}C NMR – 100 MHz

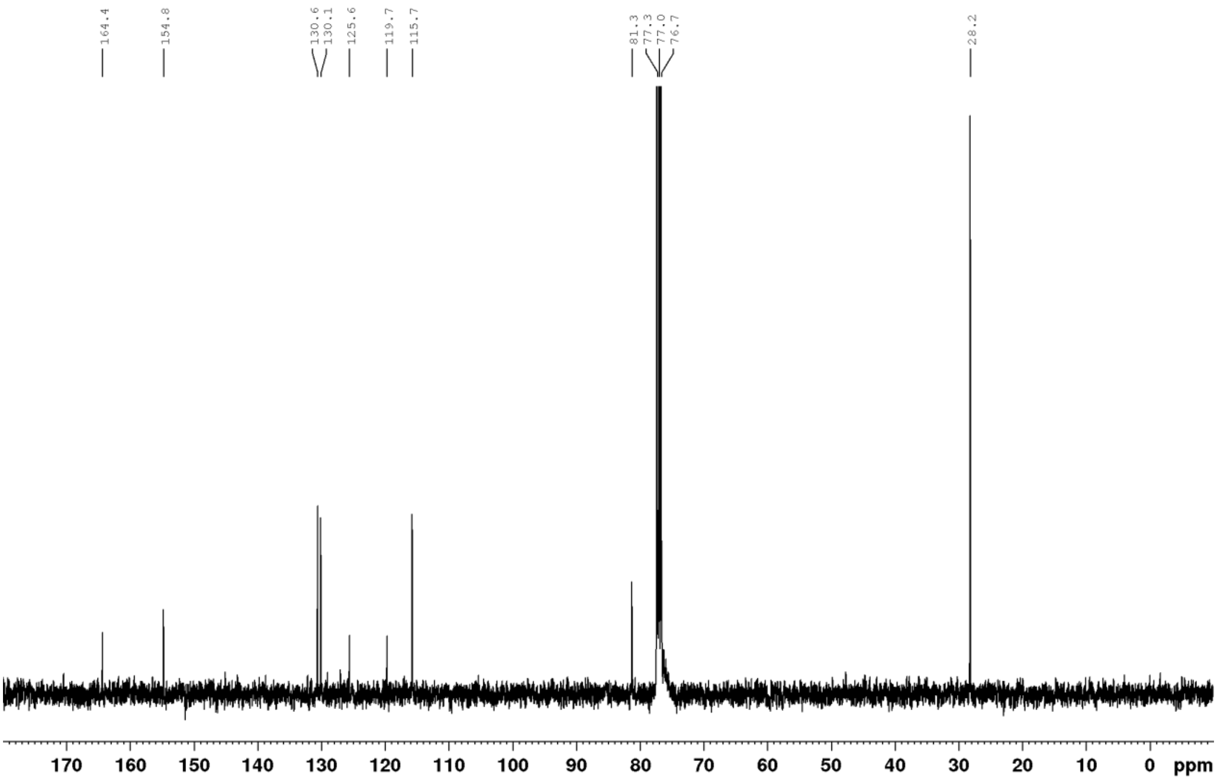

***tert*-Butyl 4-hydroxy-3-nitrobenzoate (20)**

^{1}H NMR – 400 MHz

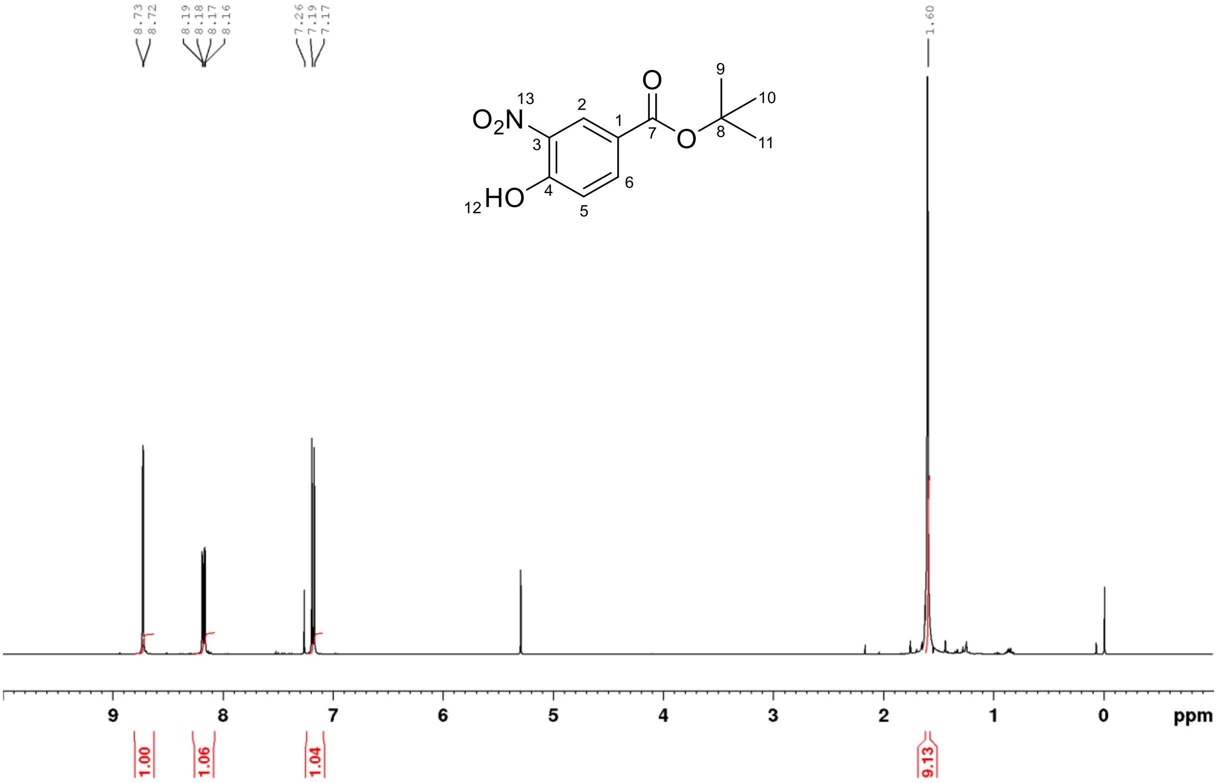

^{13}C NMR – 100 MHz

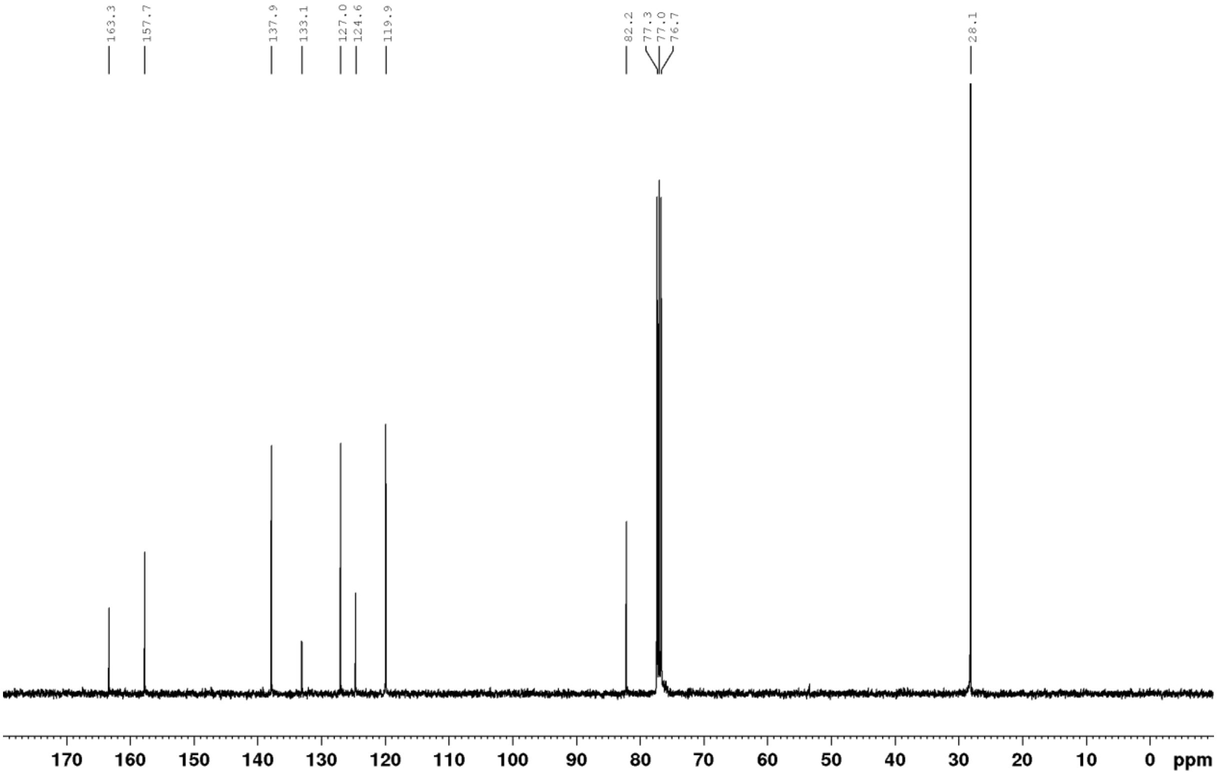

tert-Butyl 4-[(5-bromopentyl)oxy]benzoate (9)

^{1}H NMR – 400 MHz

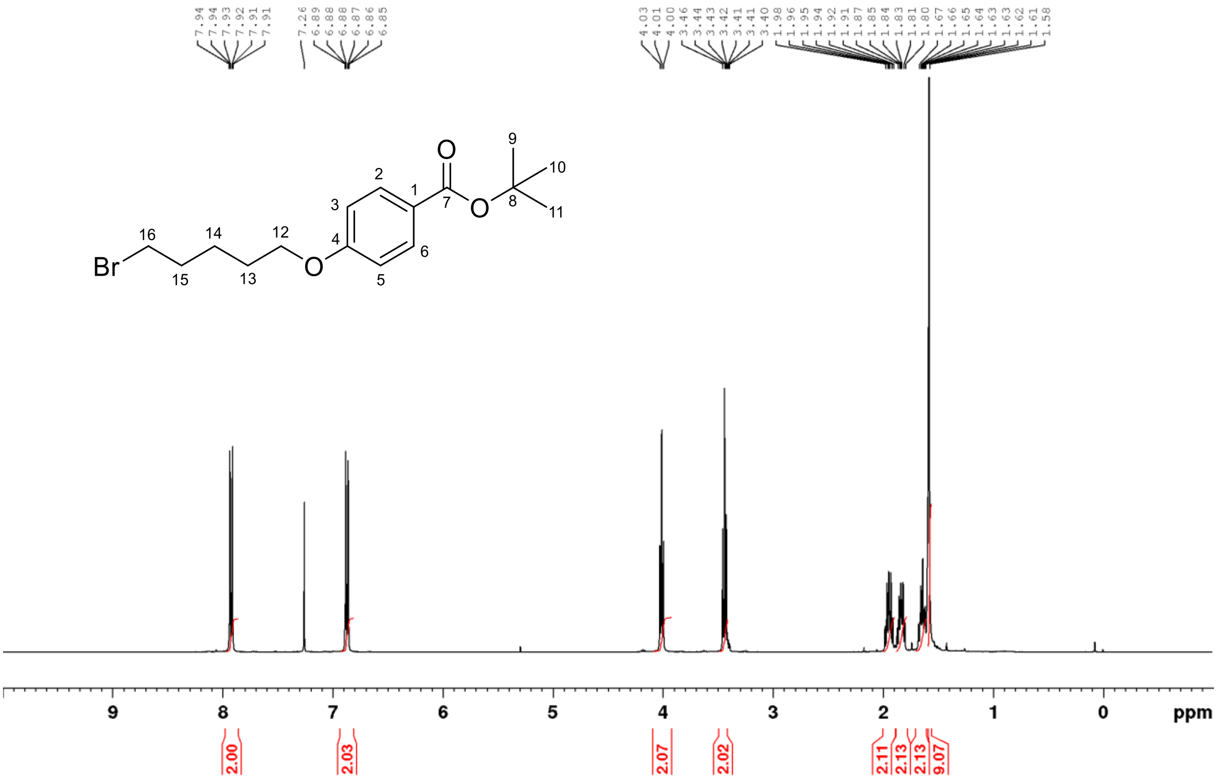

^{13}C NMR – 100 MHz

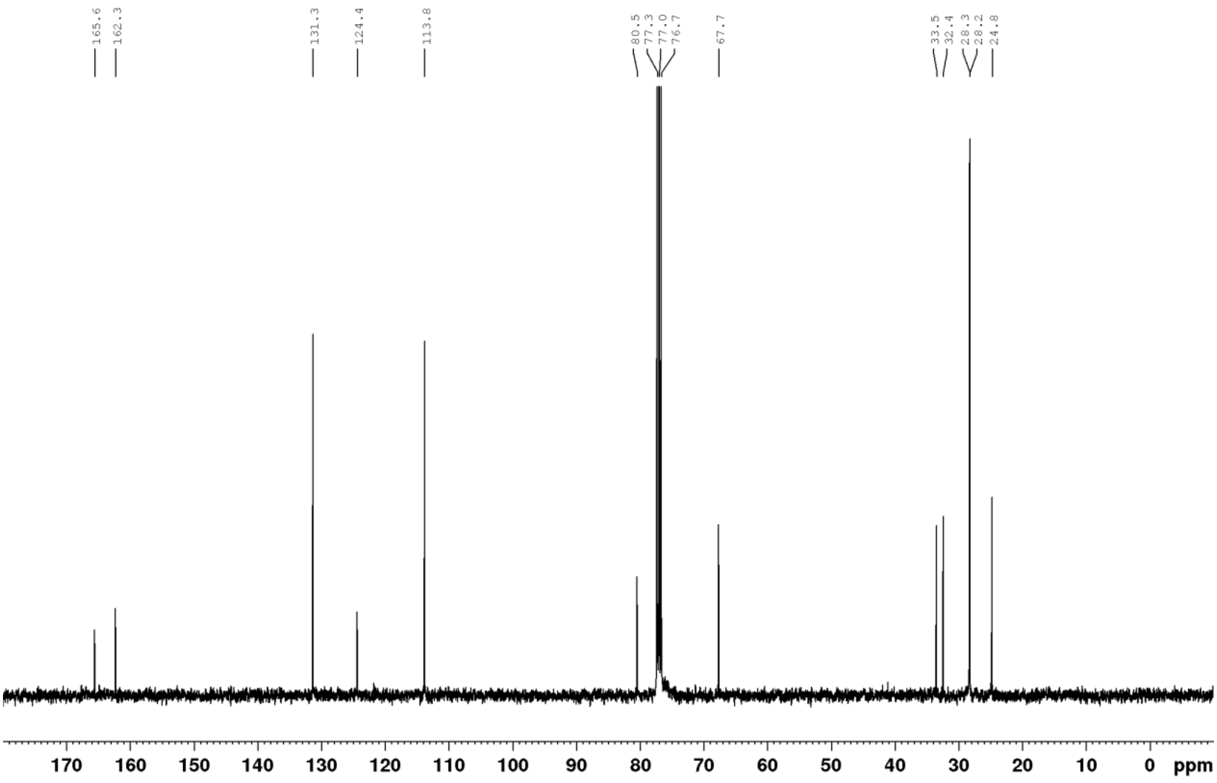

tert-Butyl 4-[(5-bromopentyl)oxy]-3-chlorobenzoate (10)

^{1}H NMR – 600 MHz

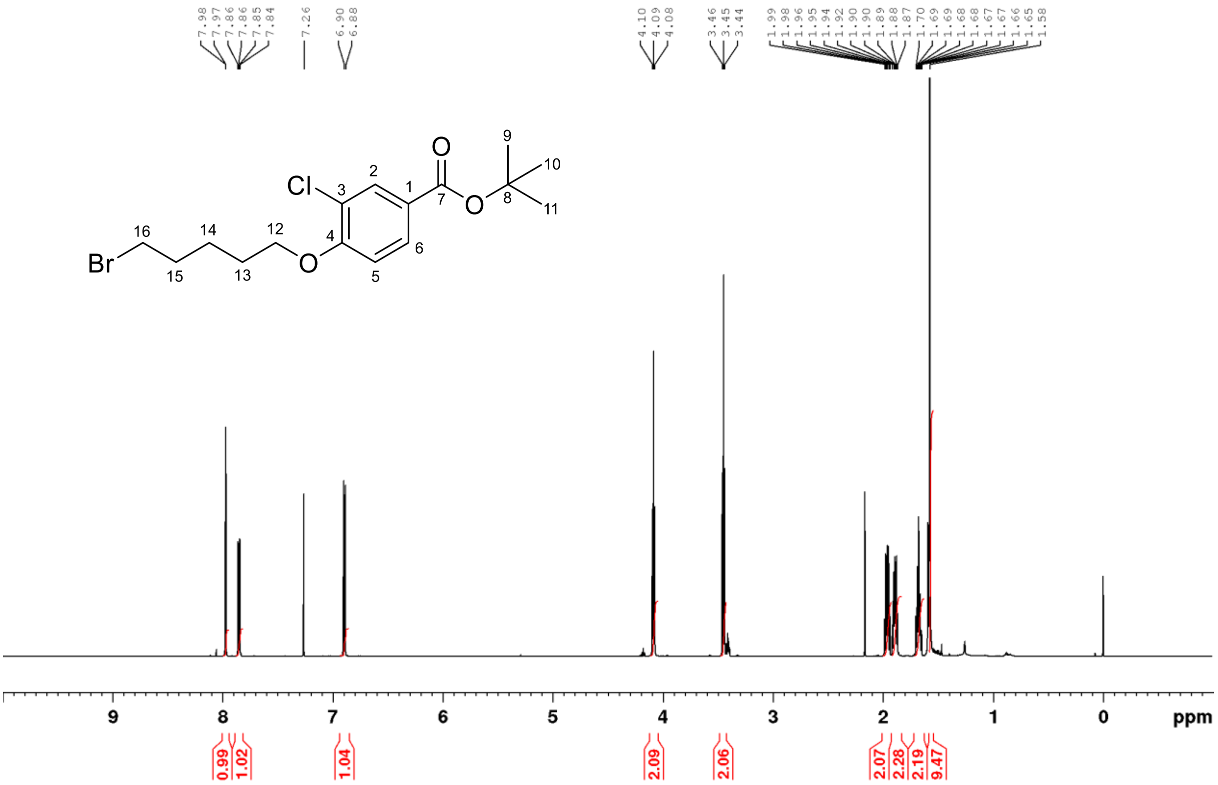

^{13}C NMR – 150 MHz

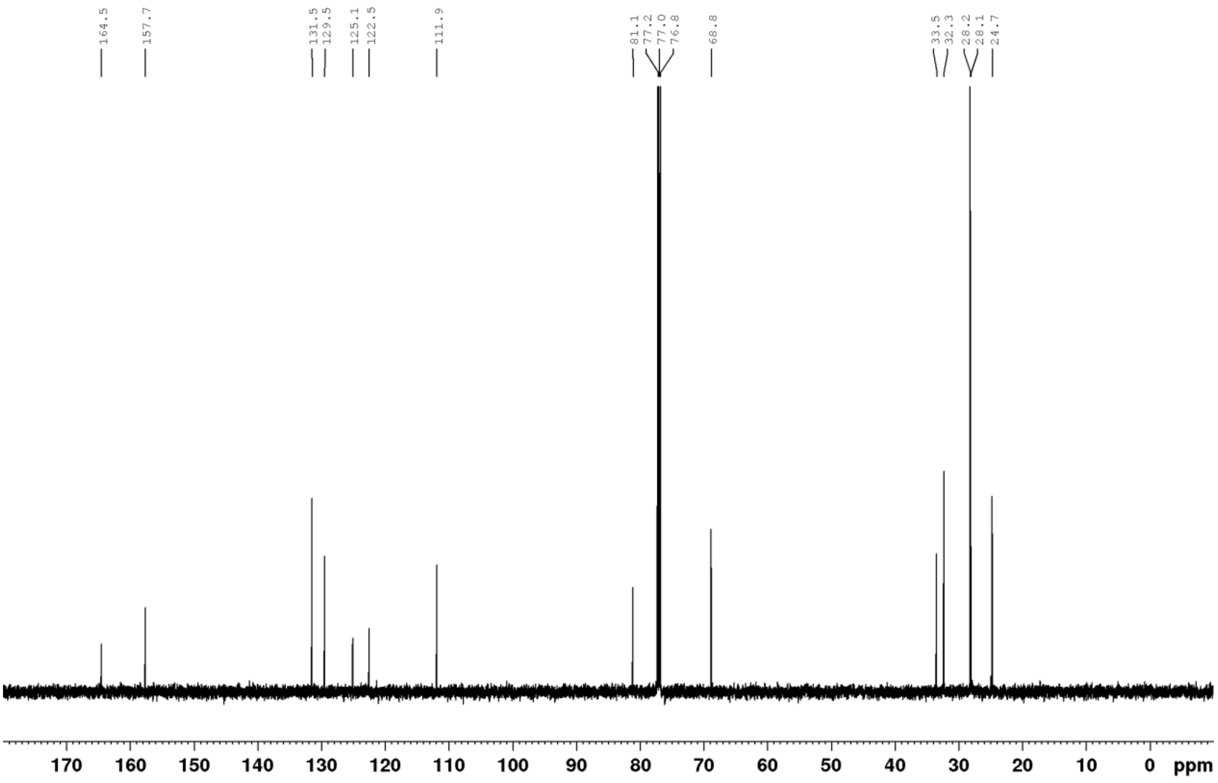

tert-Butyl 4-[(5-bromopentyl)oxy]-3-nitrobenzoate (11)

^{1}H NMR – 400 MHz

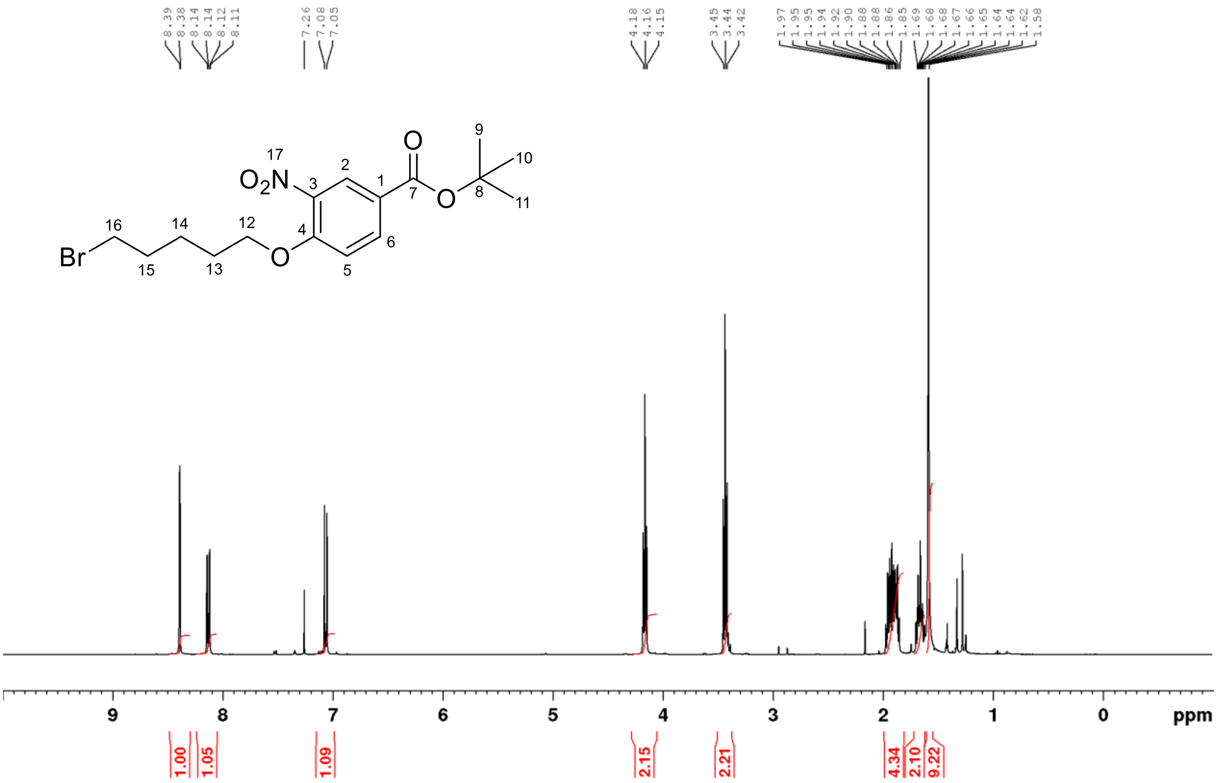

^{13}C NMR – 100 MHz

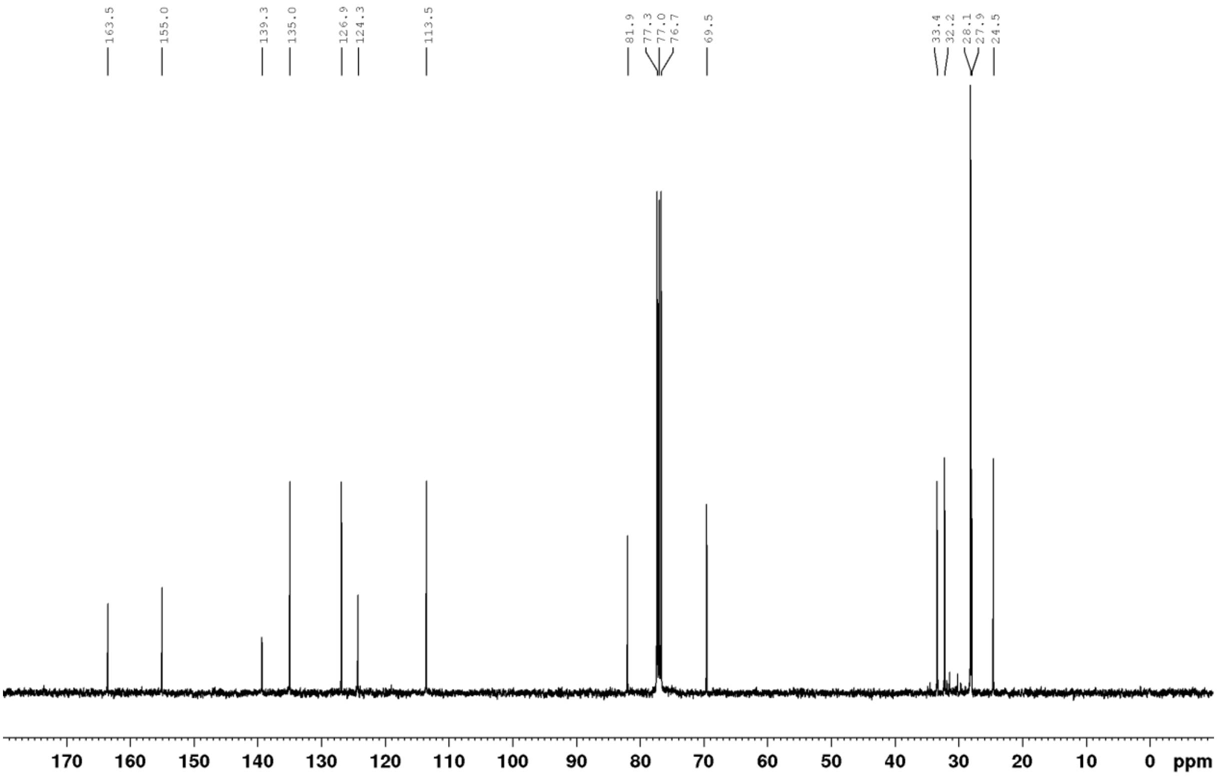

Methyl 4-sulfanylbenzoate (12)

^{1}H NMR – 400 MHz

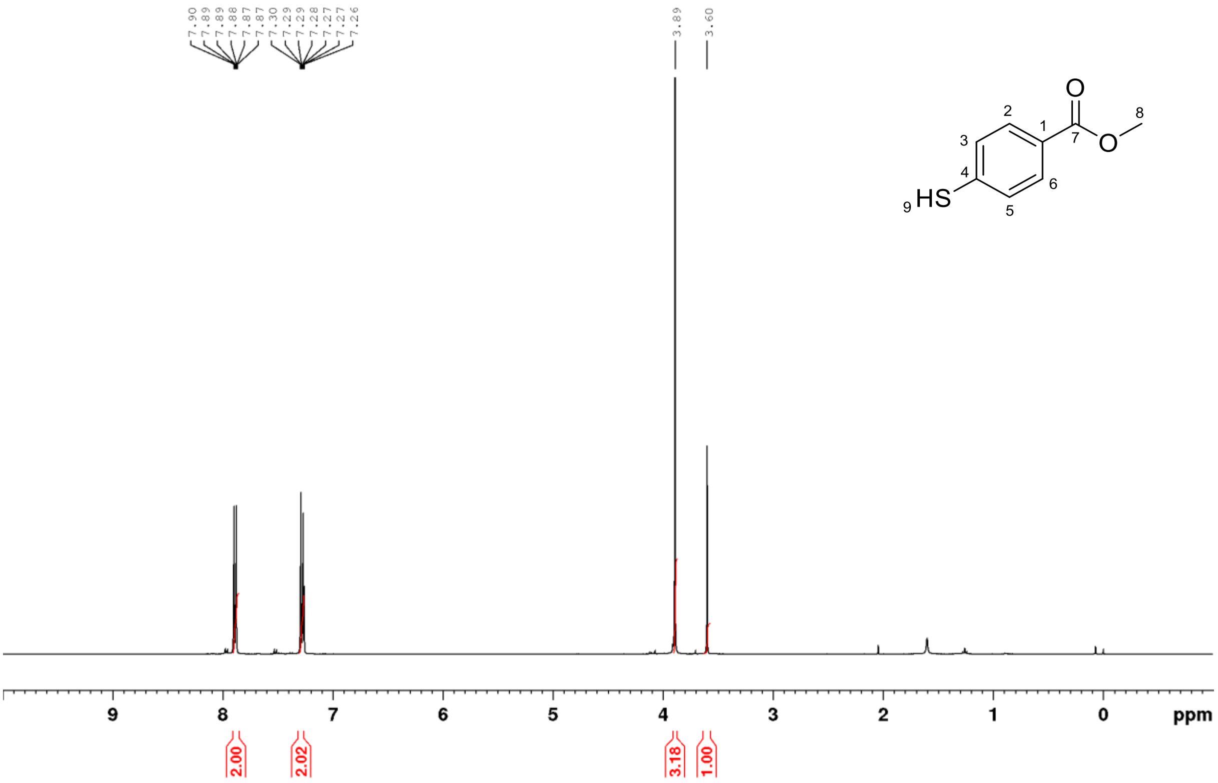

^{13}C NMR – 100 MHz

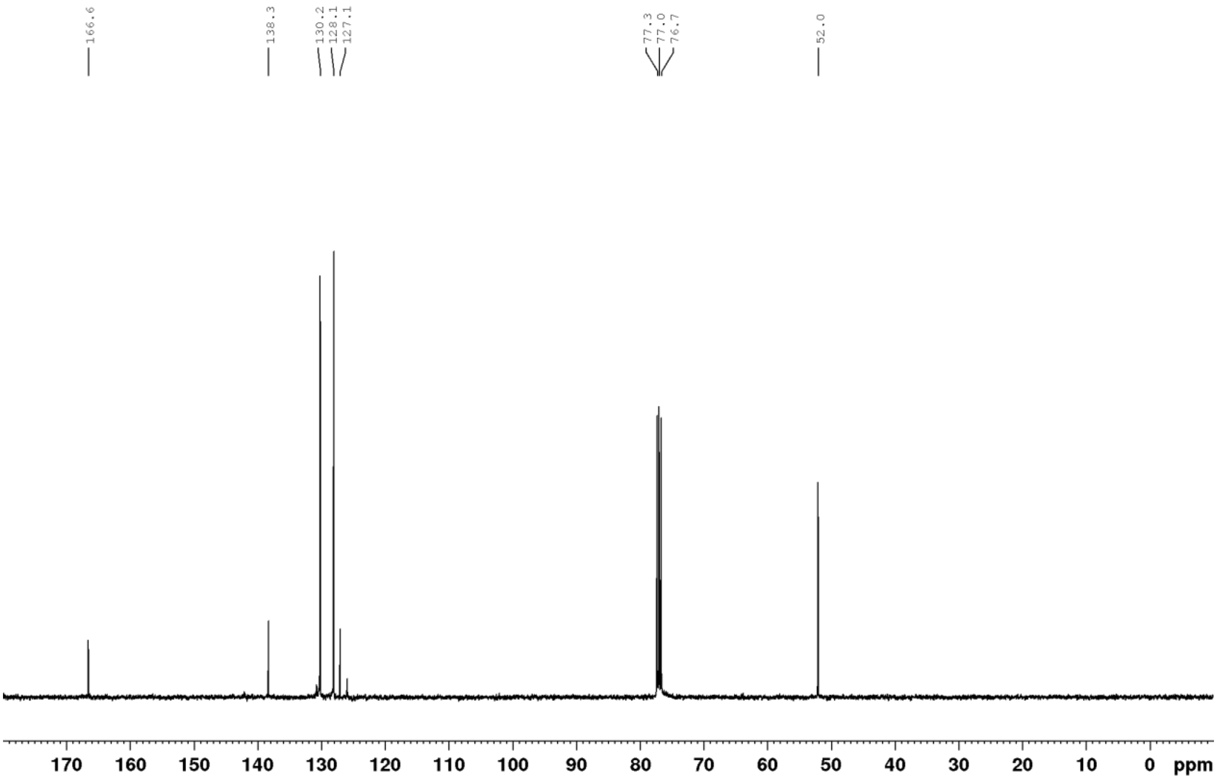

tert-Butyl [3-[(6-chloropyridin-3-yl)methyl]-2-(nitroimino)imidazolidin-1-yl]acetate (7a)

^{1}H NMR – 600 MHz

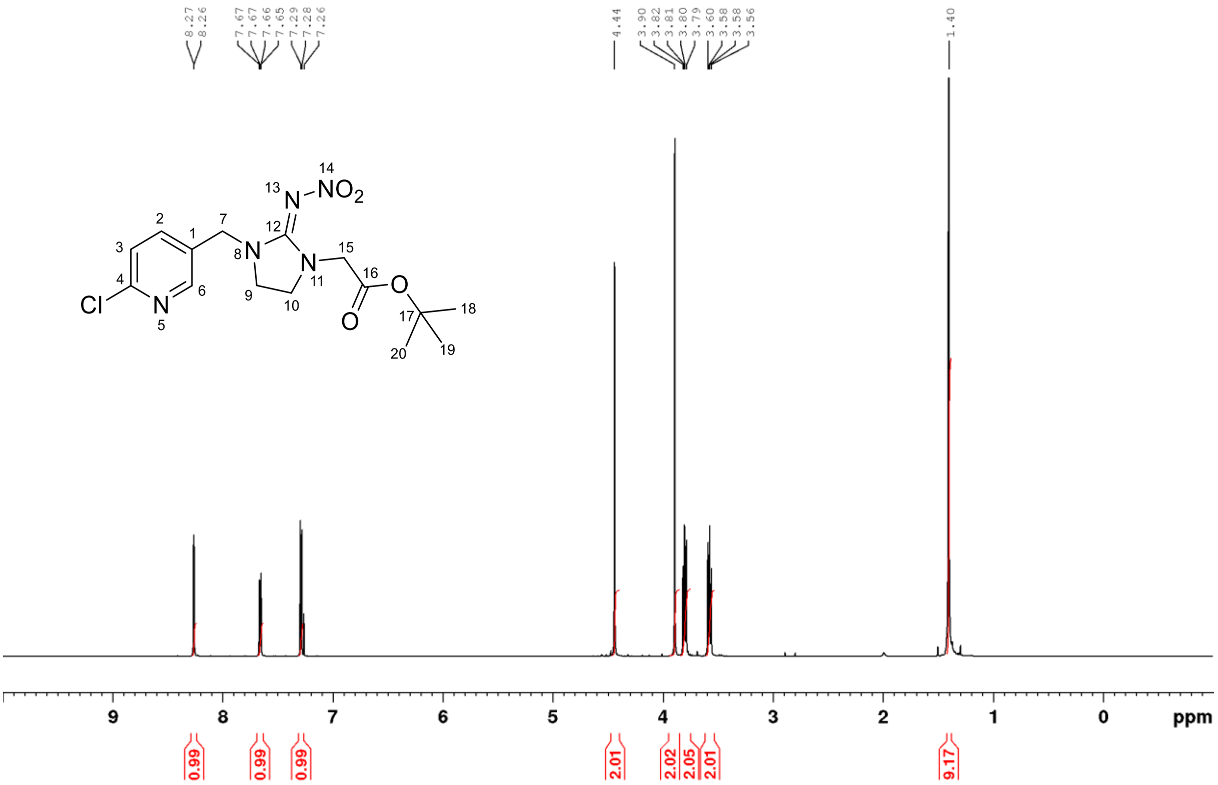

^{13}C NMR – 150 MHz

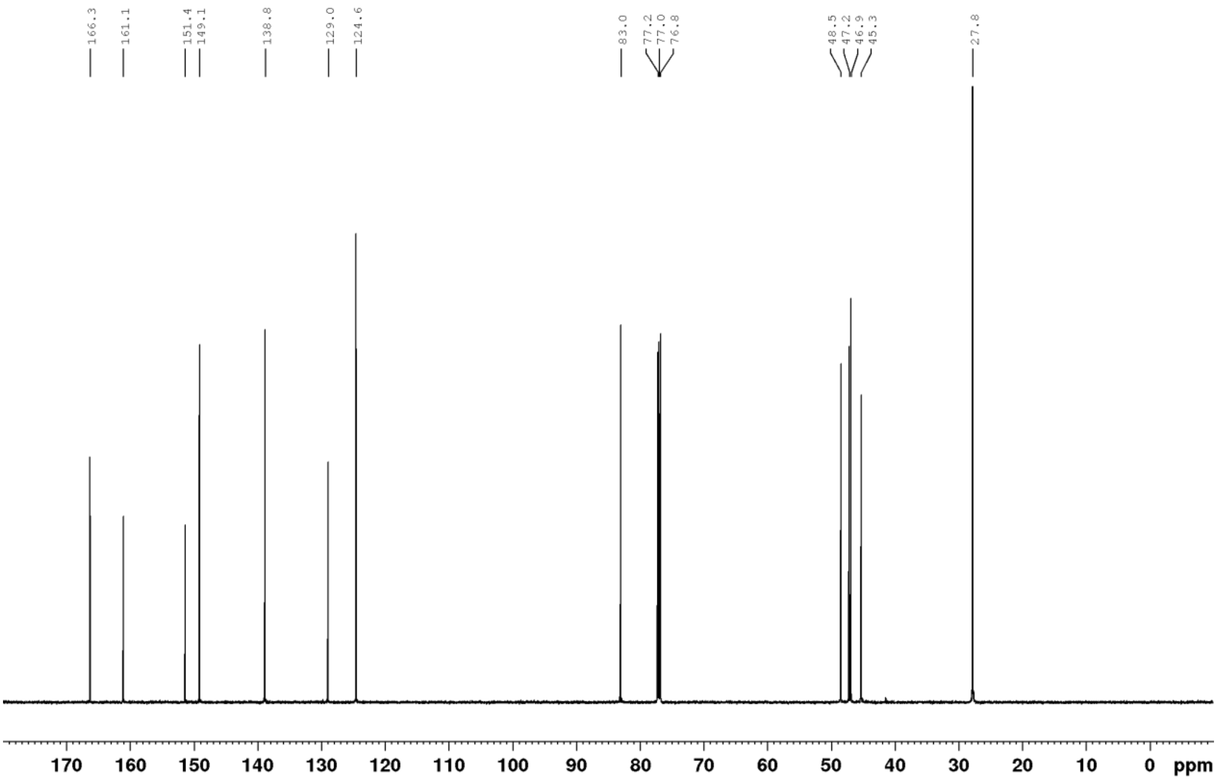

^{15}N NMR – 61 MHz

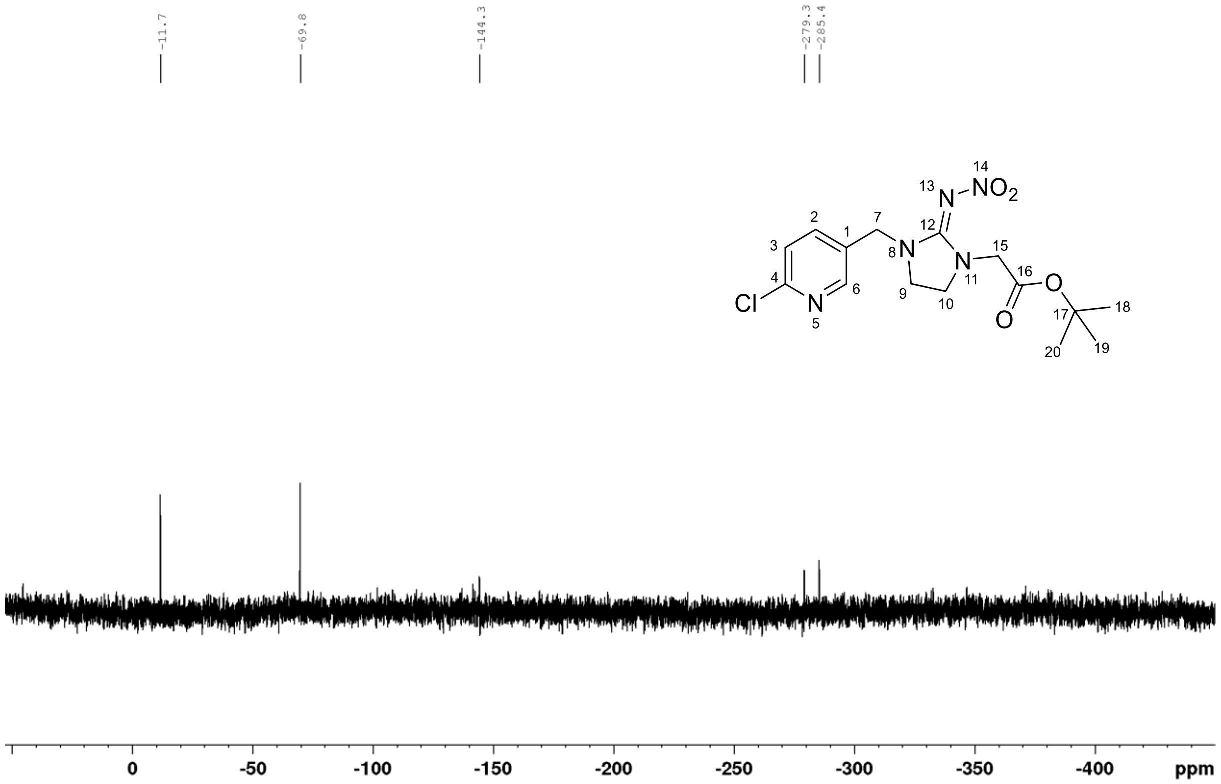

***tert*-Butyl 4-{[3-[(6-chloropyridin-3-yl)methyl]-2-(nitroimino)imidazolidin-1-yl]methyl}benzoate (8a)**

^{1}H NMR – 600 MHz

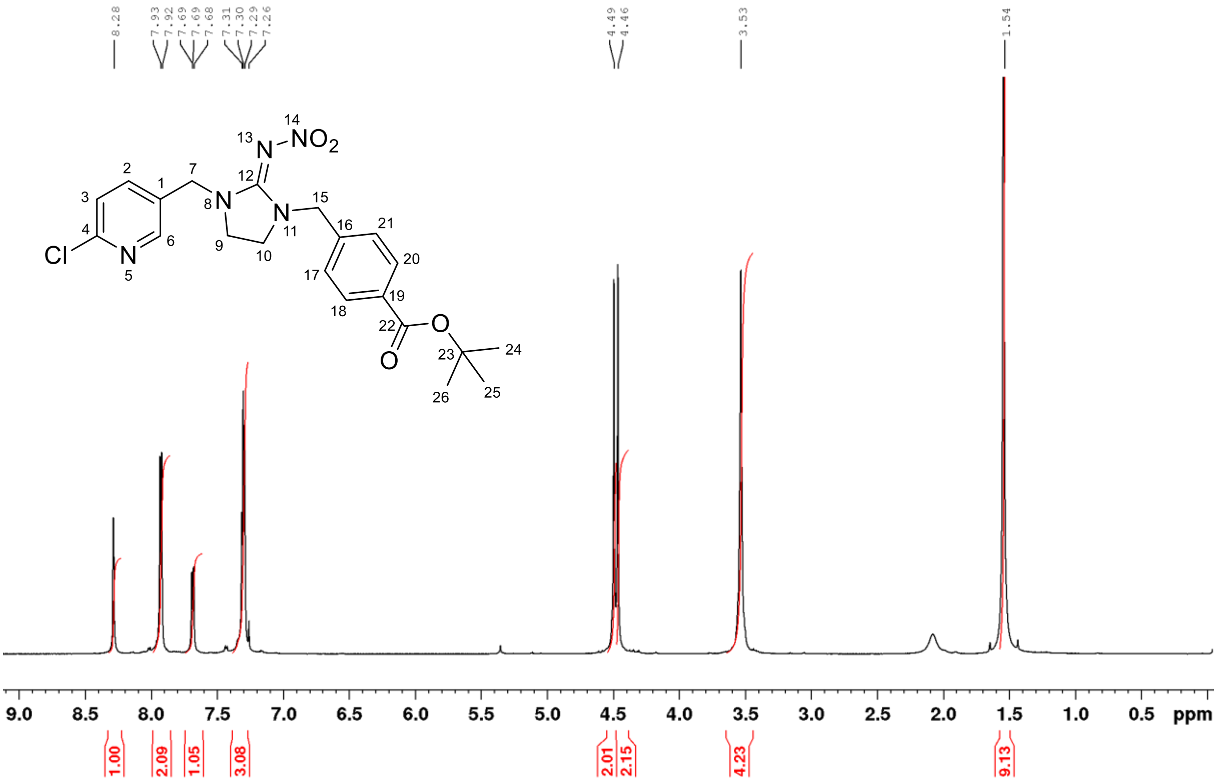

^{13}C NMR – 150 MHz

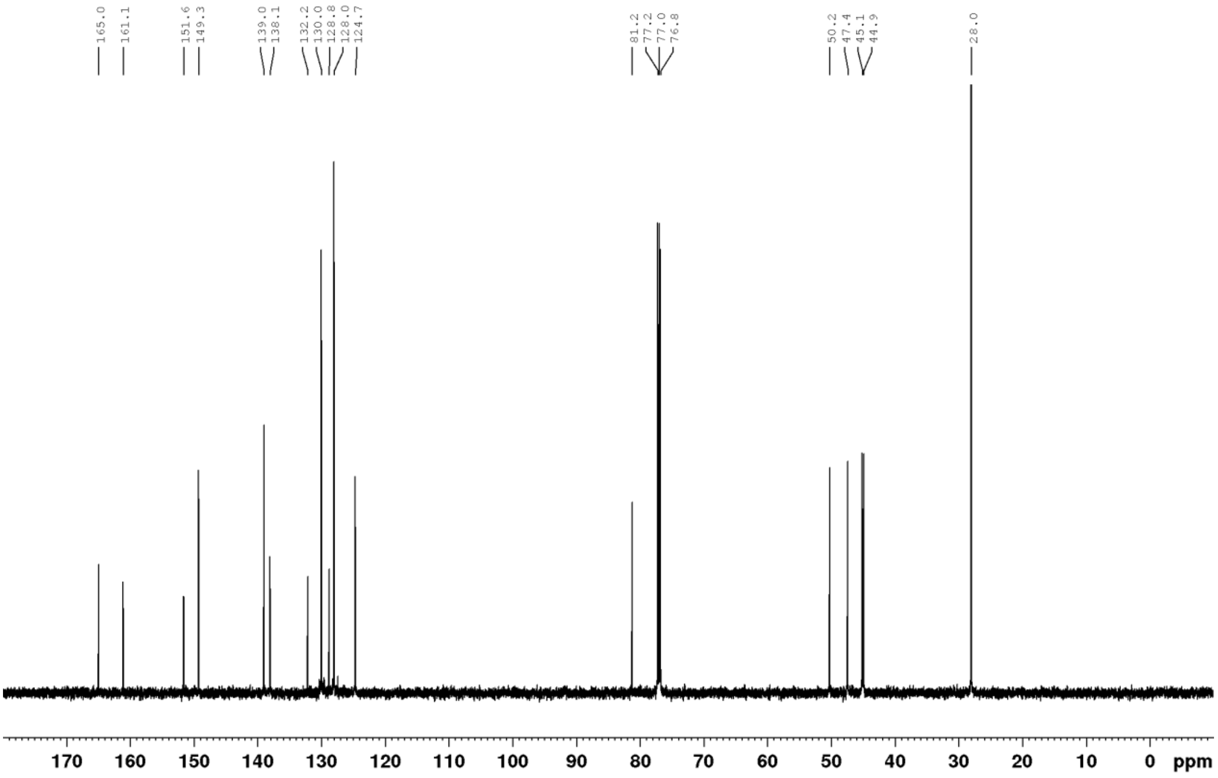

^{15}N NMR – 61 MHz

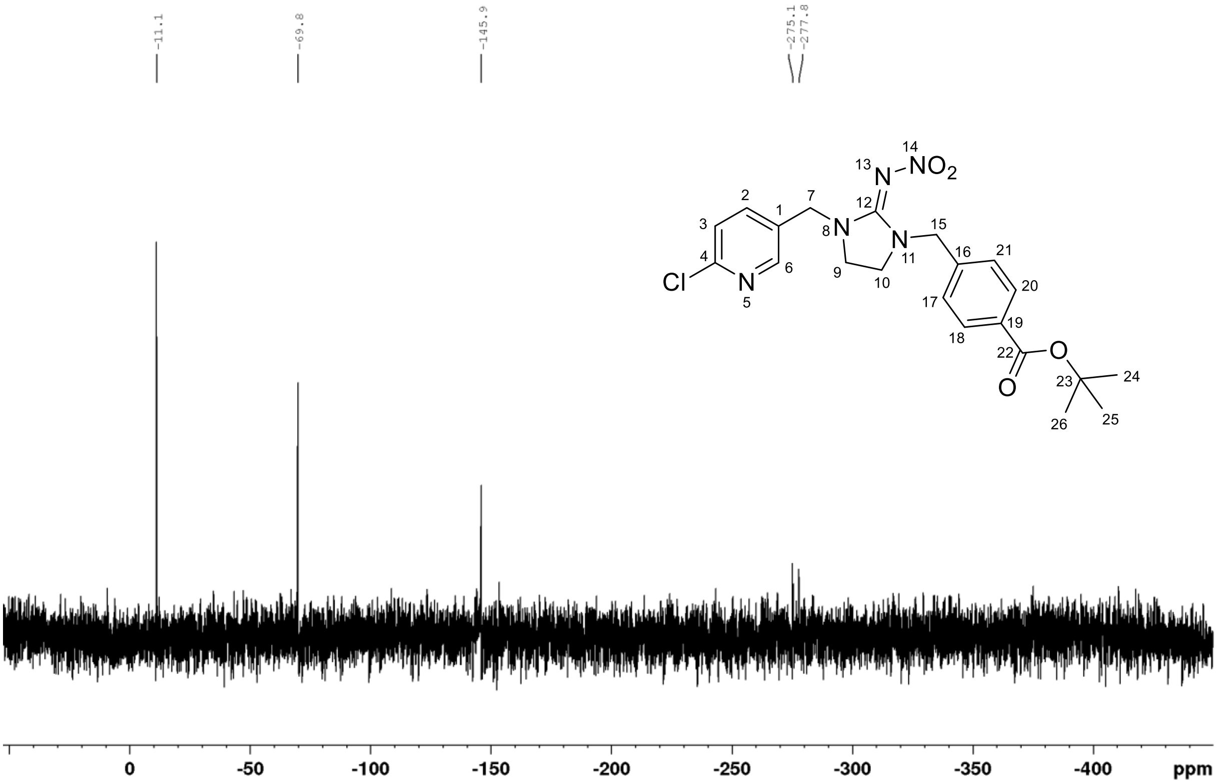

***tert*-Butyl 4-({5-[3-[(6-chloropyridin-3-yl)methyl]-2-(nitroimino)imidazolidin-1-yl]pentyl}oxy)benzoate (9a)**

^{1}H NMR – 600 MHz

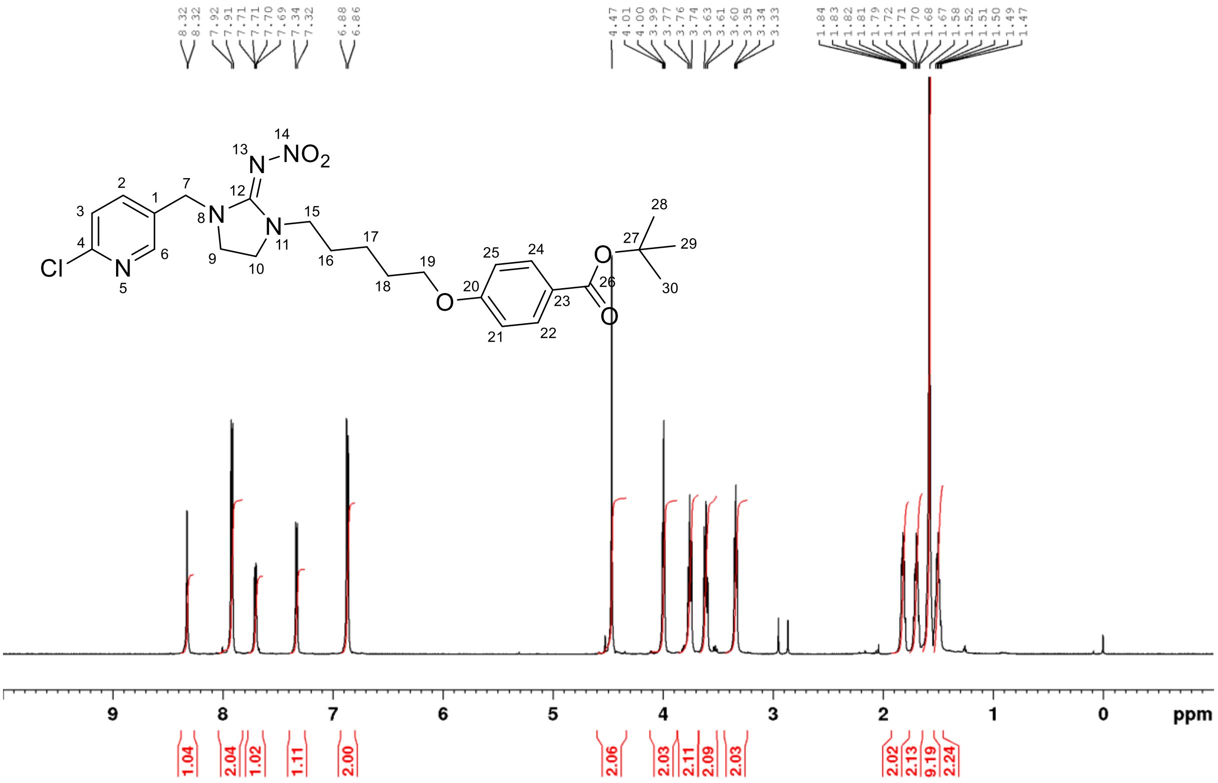

^{13}C NMR – 150 MHz

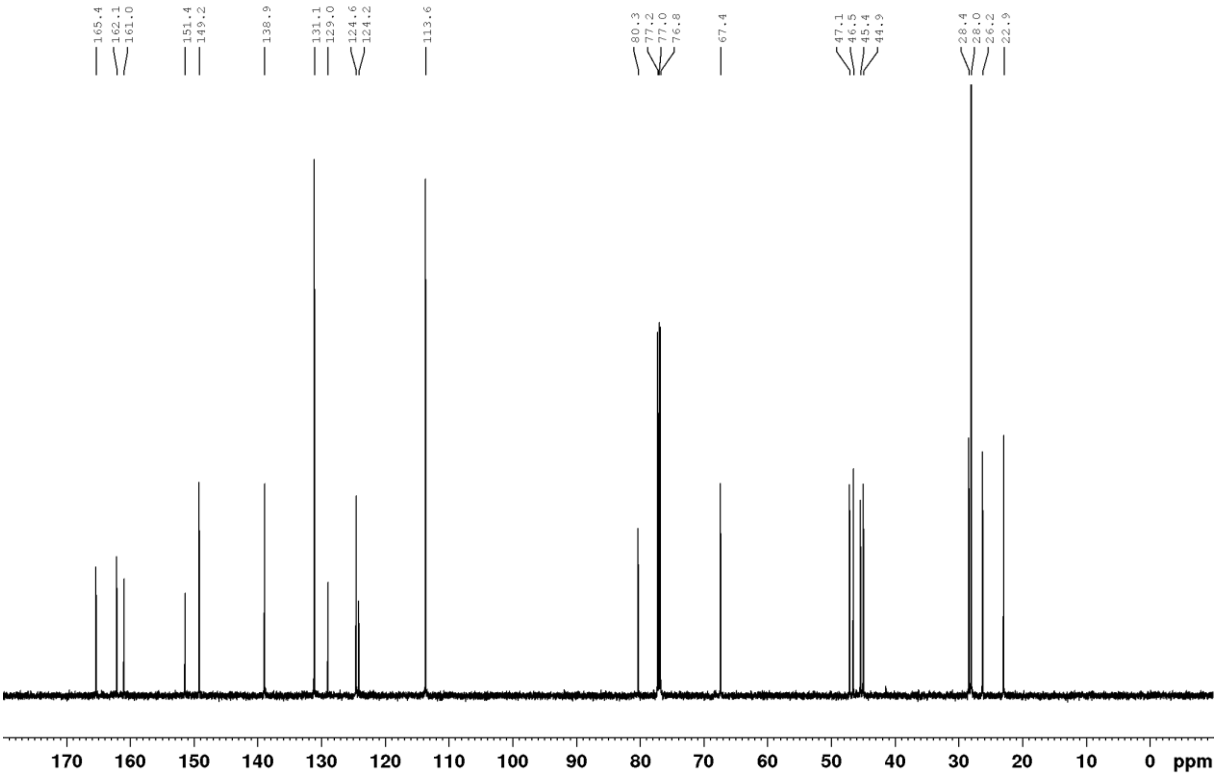

^{15}N NMR – 61 MHz

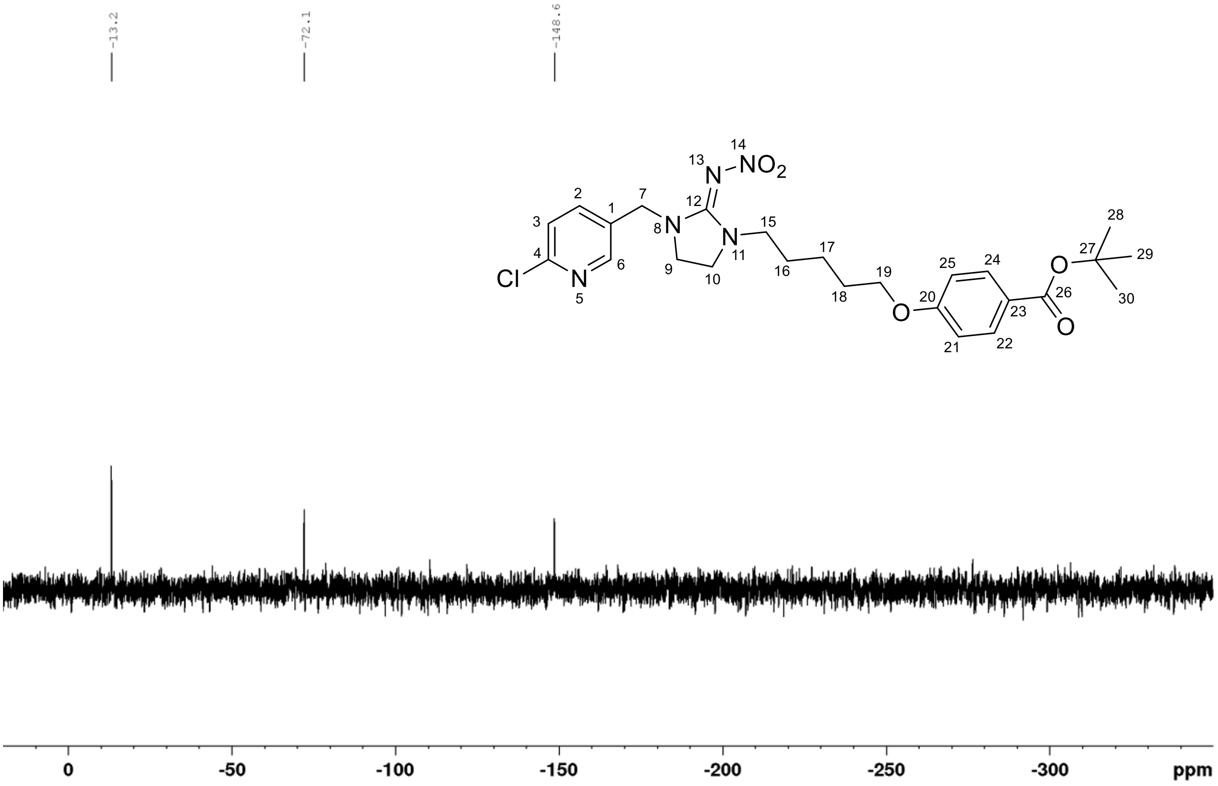

N-8 and N-11 were only detected via $^{1}H,^{15}N$ HMBC:

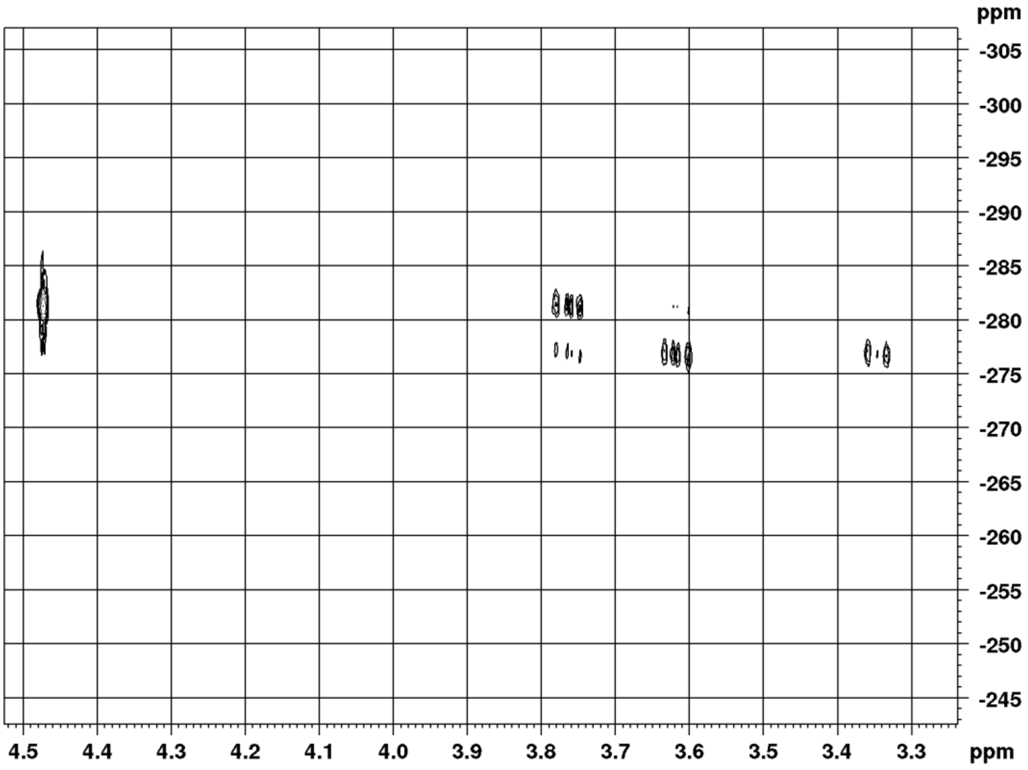

tert-Butyl 3-chloro-4-({5-[3-[(6-chloropyridin-3-yl)methyl]-2-(nitroimino)imidazolidin-1-yl]pentyl}oxy)benzoate (10a)

^{1}H NMR – 600 MHz

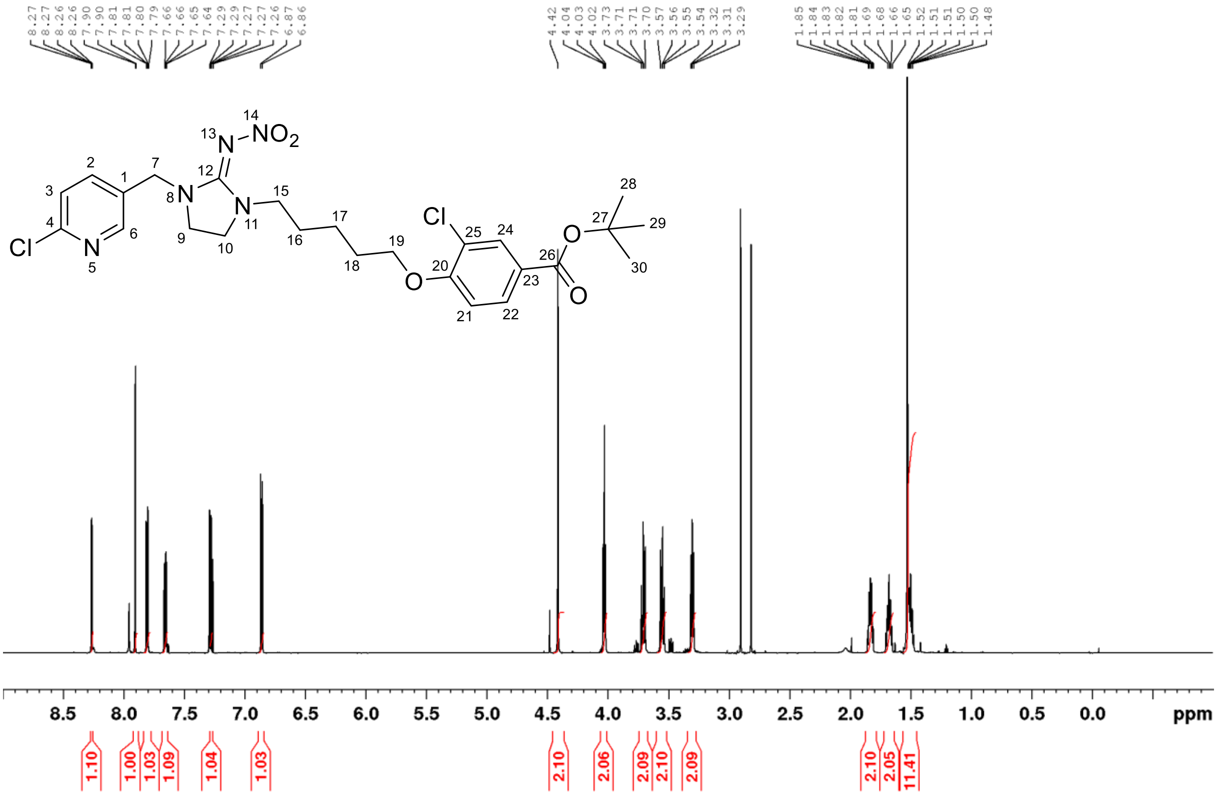

^{13}C NMR – 150 MHz

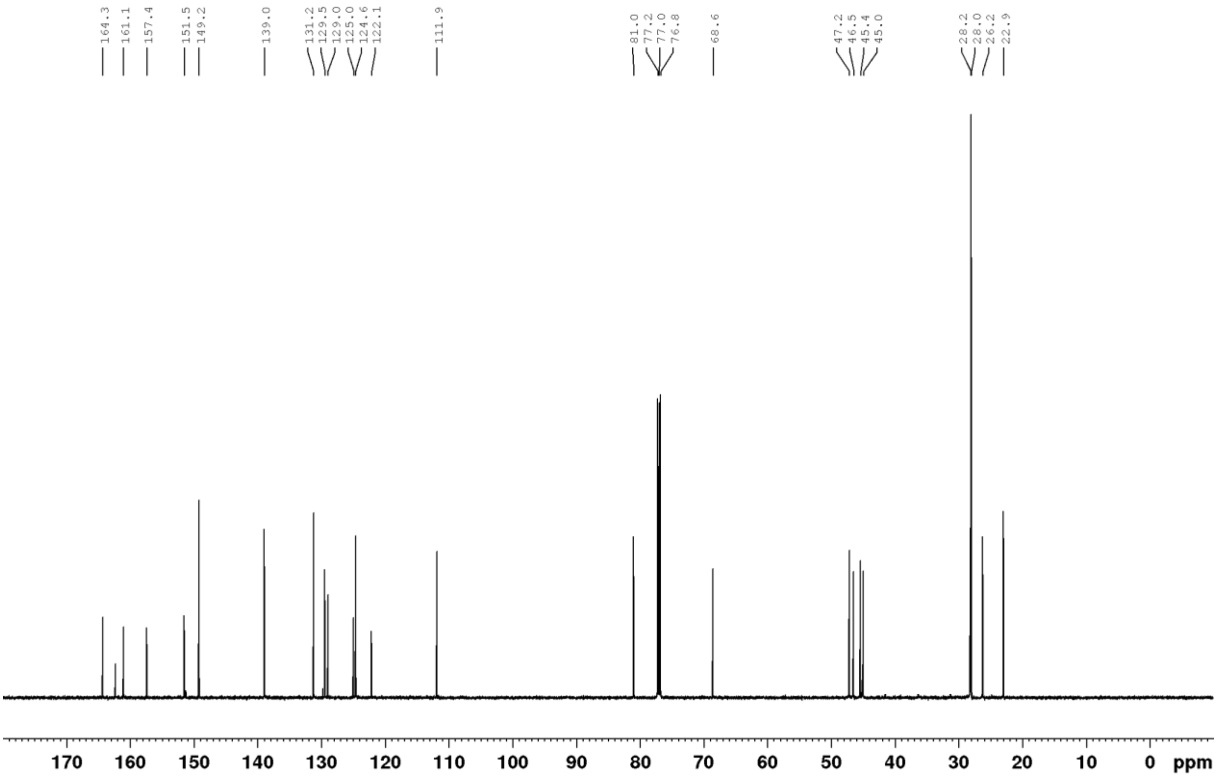

^{15}N NMR – 61 MHz

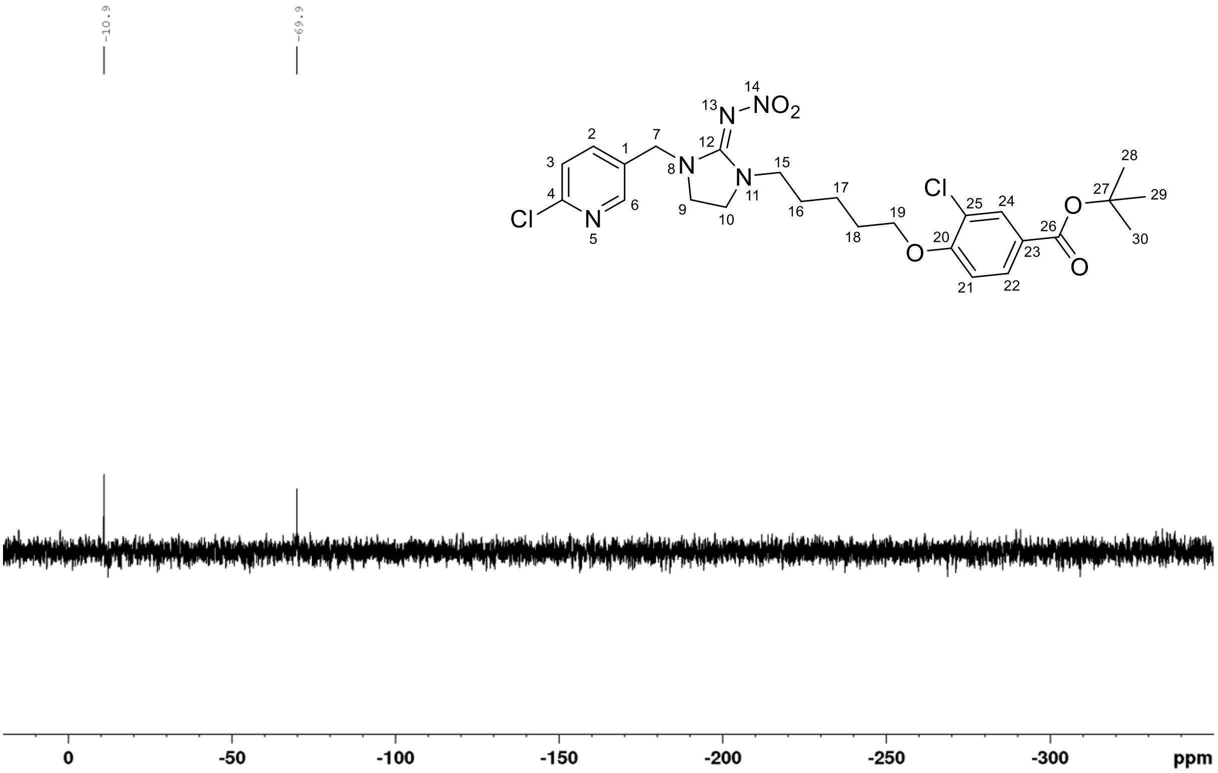

N-8, N-11 and N-13 were only detected via ^{1}H,^{15}N HMBC:

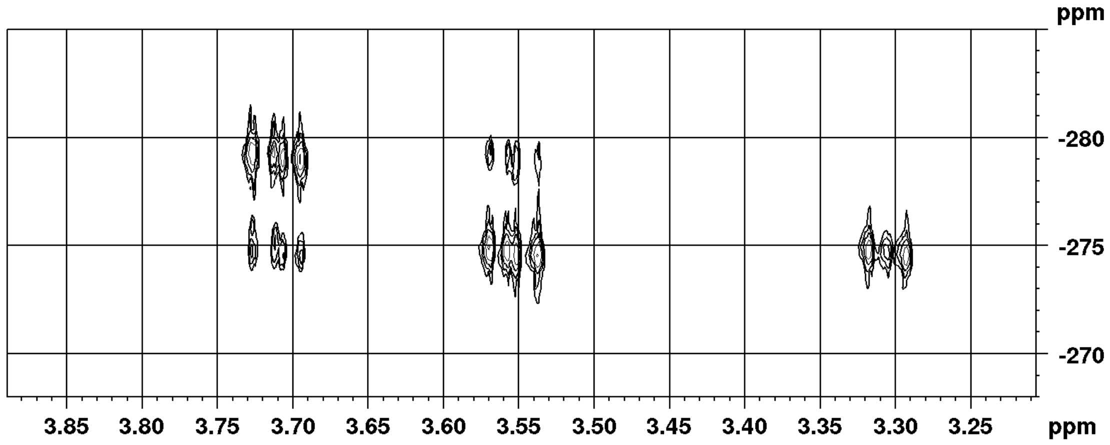

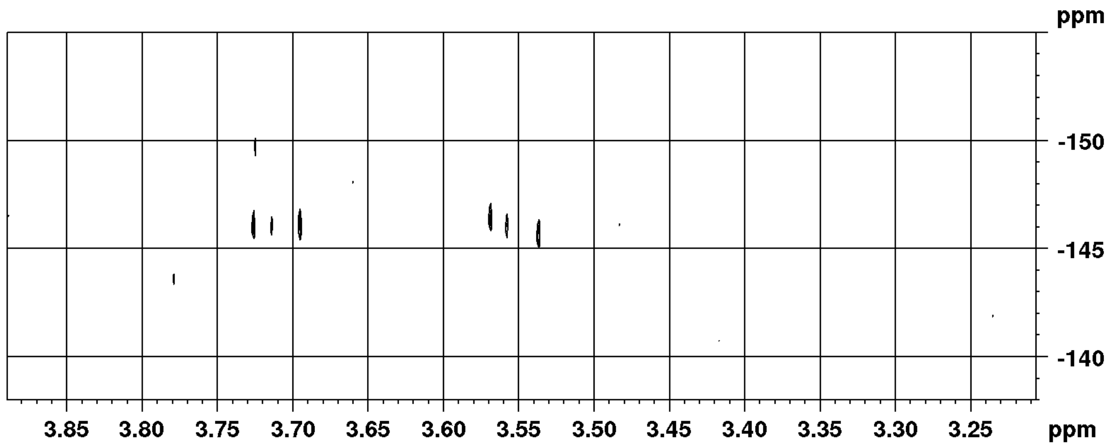

***tert*-Butyl 4-({5-[3-[(6-chloropyridin-3-yl)methyl]-2-(nitroimino)imidazolidin-1-yl]pentyl}oxy)-3-nitrobenzoate (11a)**

^{1}H NMR – 600 MHz

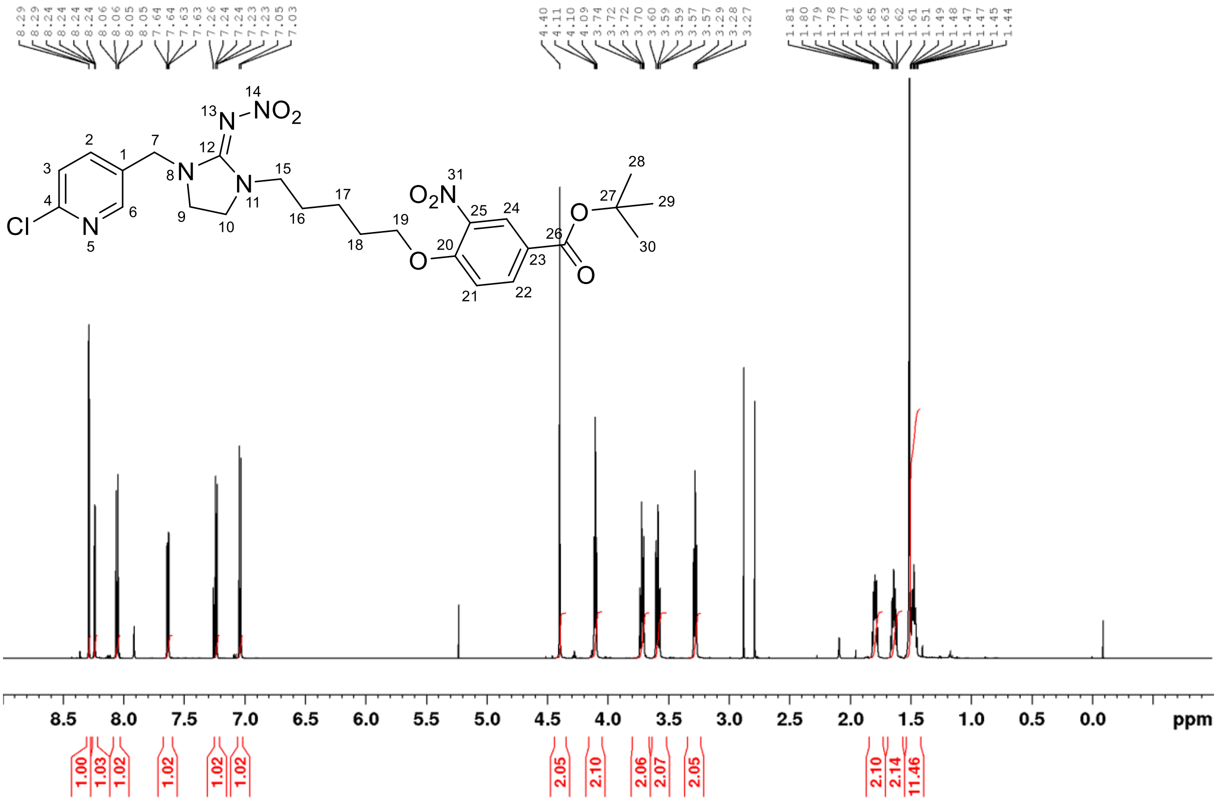

^{13}C NMR – 150 MHz

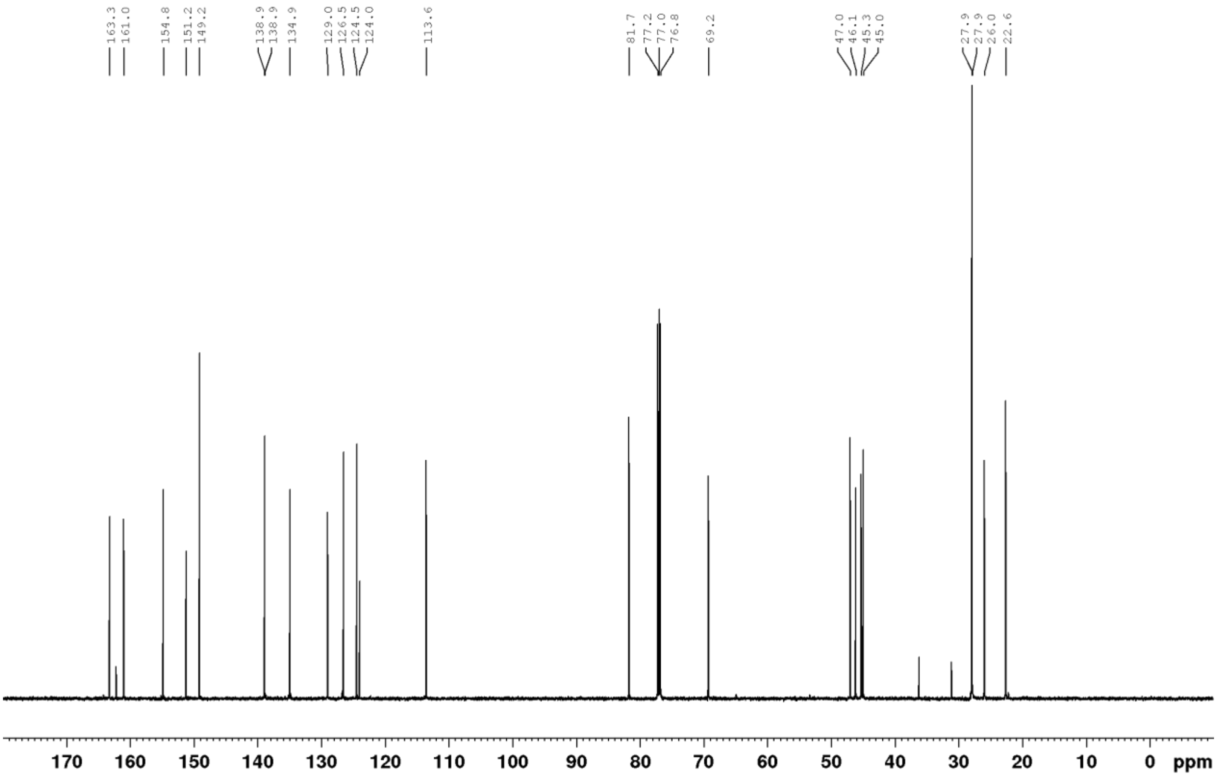

^{15}N NMR – 61 MHz

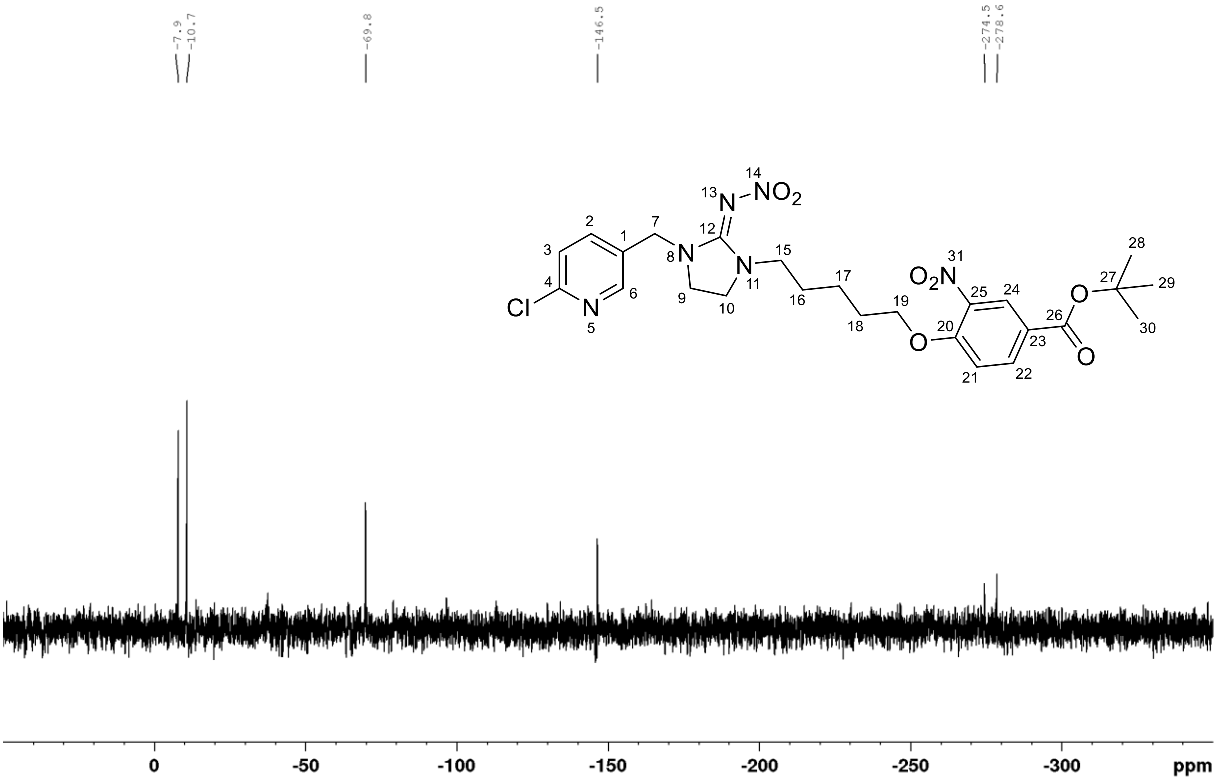

Methyl 4-{[1,1-dichloro-3-{1-[(6-chloropyridin-3-yl)methyl]imidazolidin-2-ylidene}-3-nitroprop-1-en-2-yl]sulfanyl}benzoate (12a)

^{1}H NMR – 600 MHz

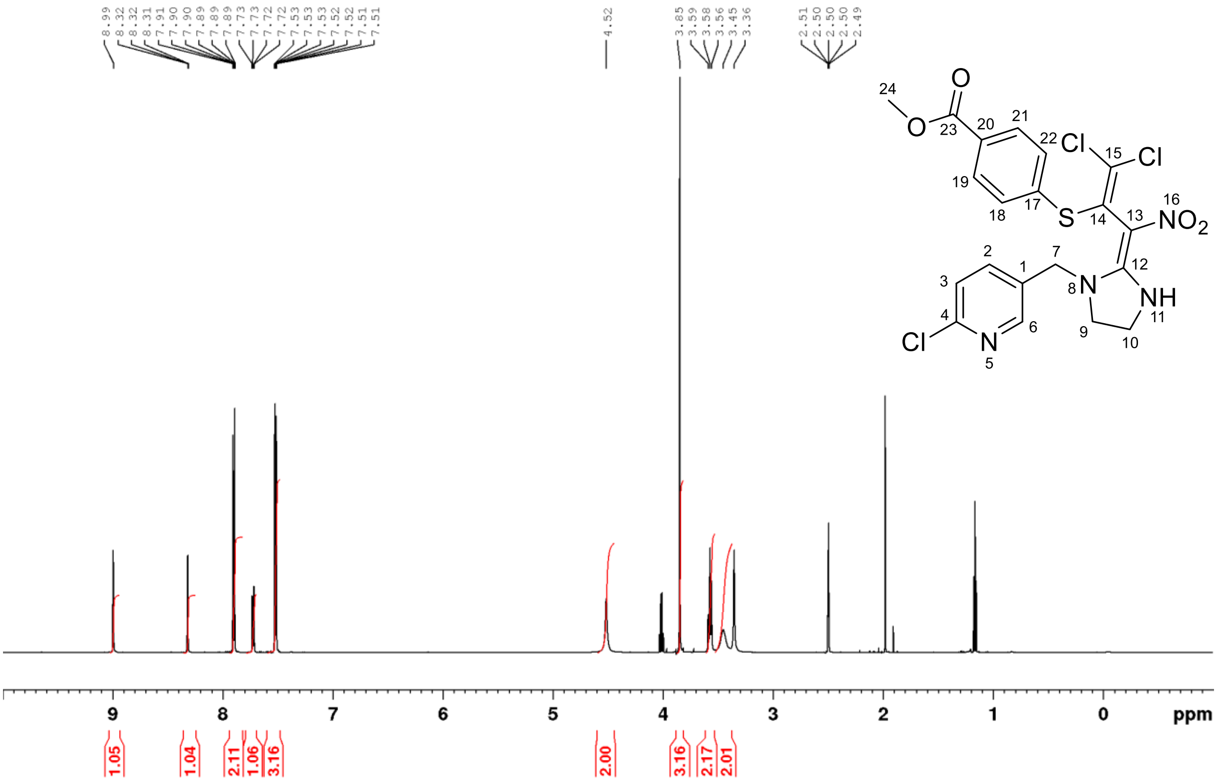

^{13}C NMR – 150 MHz

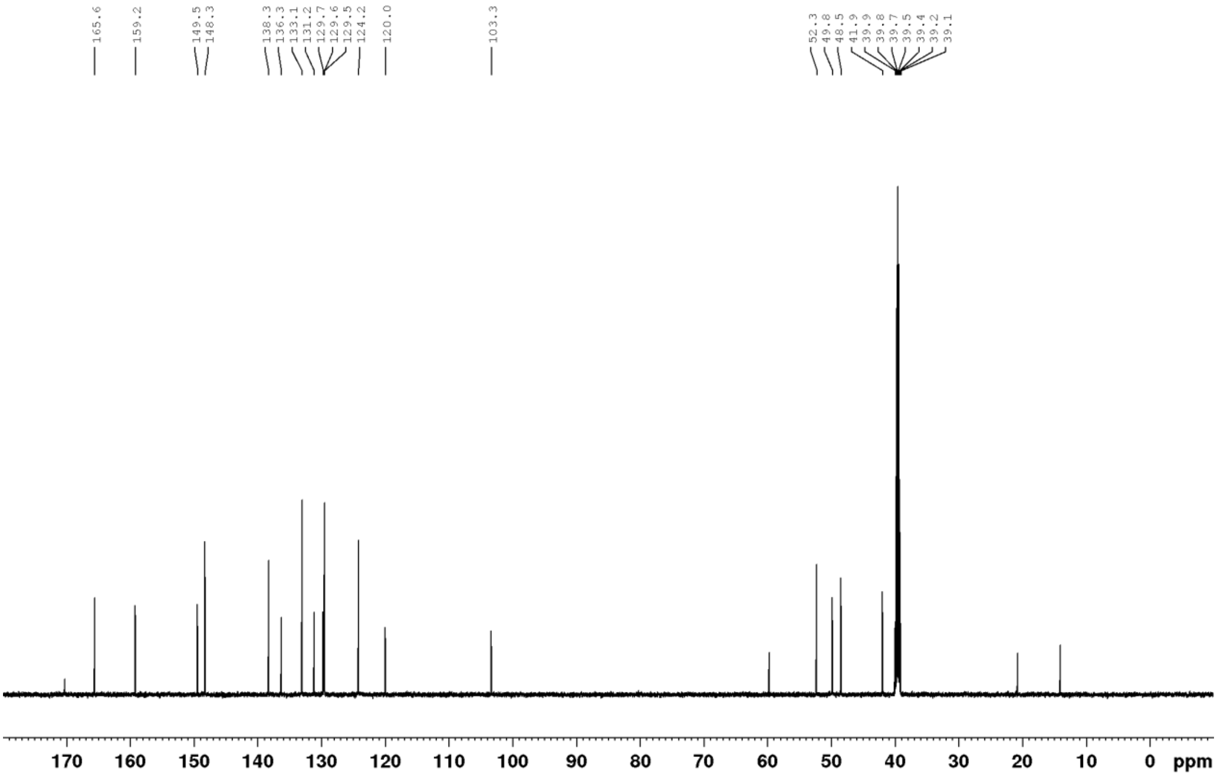

^{15}N NMR – 61 MHz

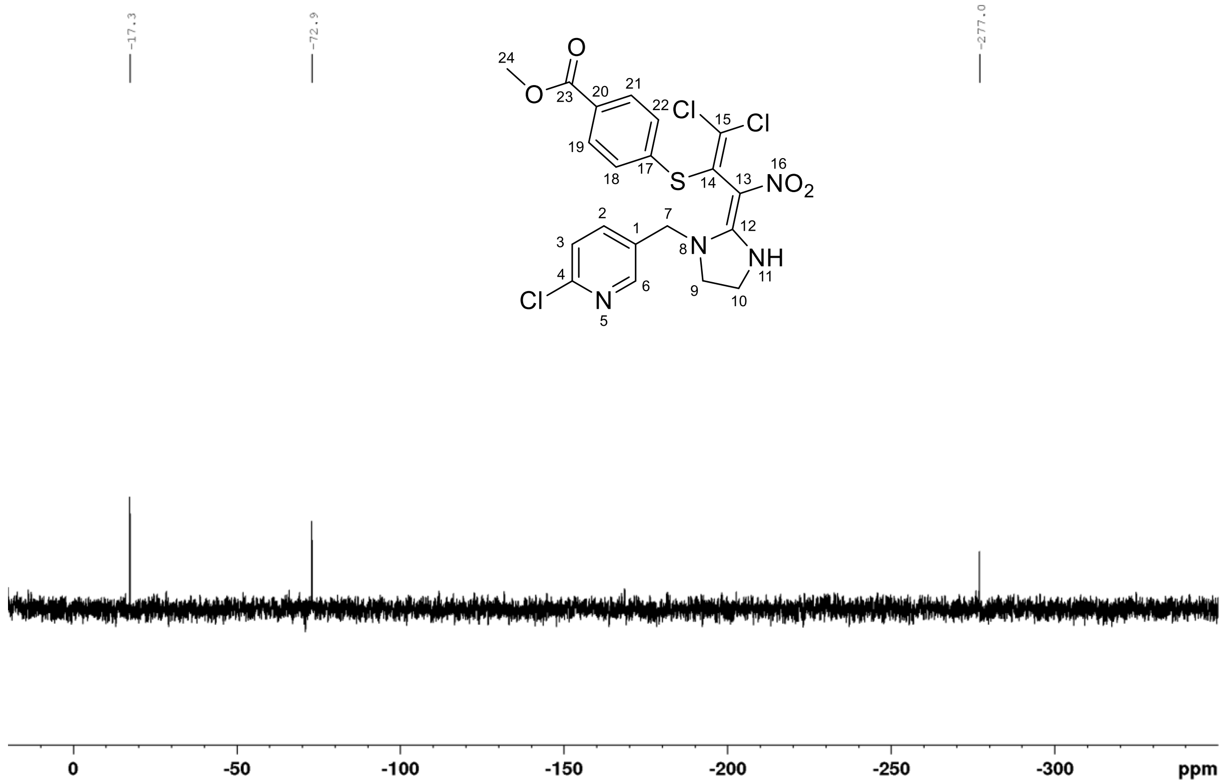

N-8 was only detected via $^{1}H,^{15}N$ HMBC:

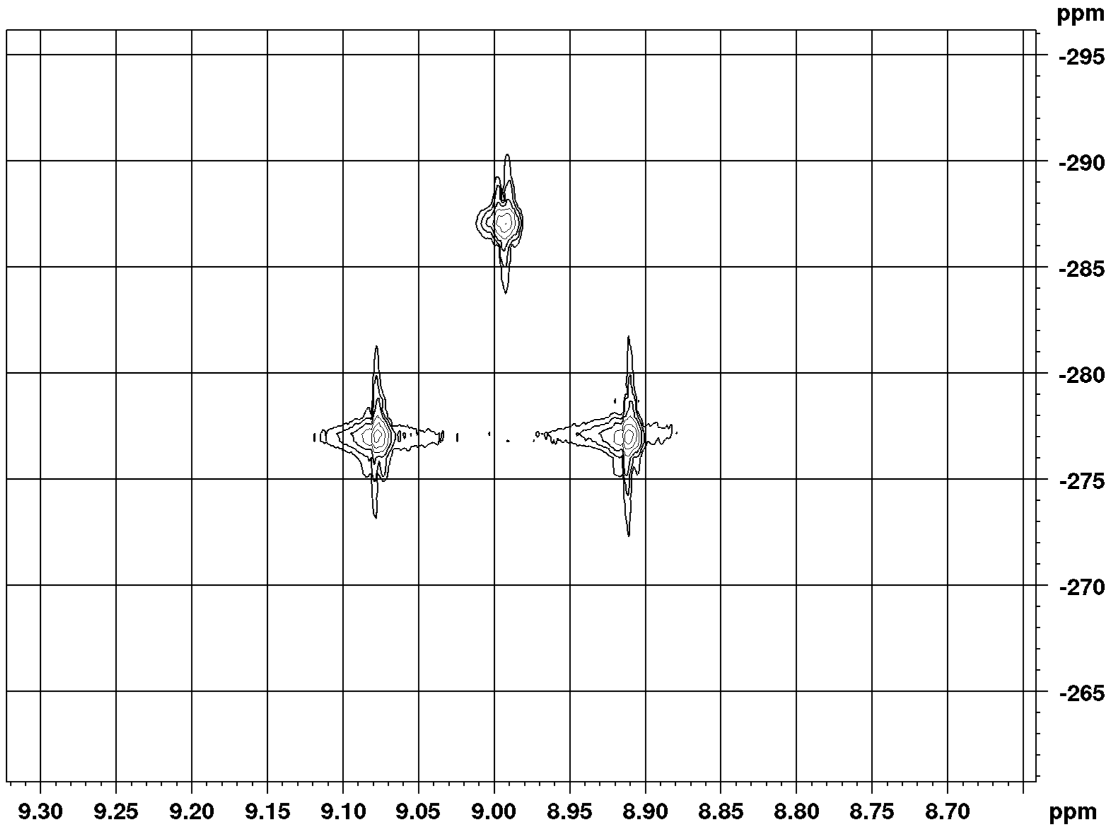

[3-[(6-Chloropyridin-3-yl)methyl]-2-(nitroimino)imidazolidin-1-yl]acetic acid (7b)

^{1}H NMR – 600 MHz

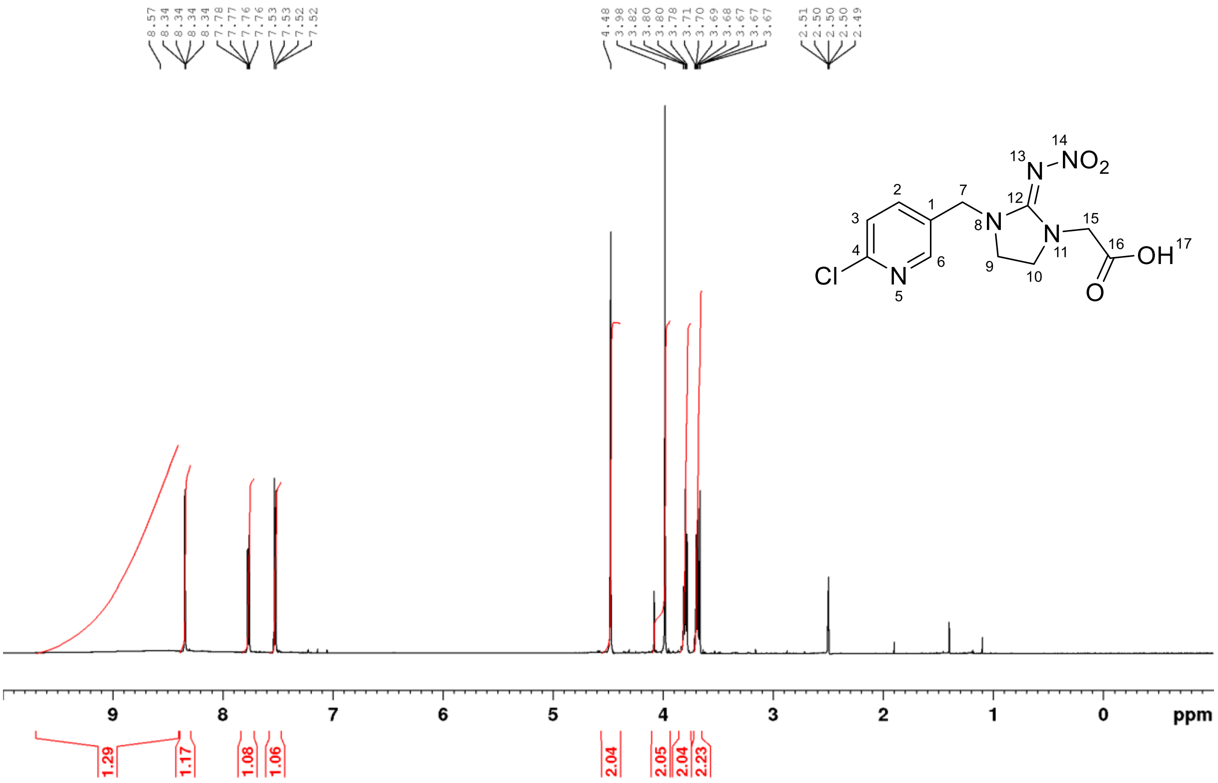

^{13}C NMR – 150 MHz

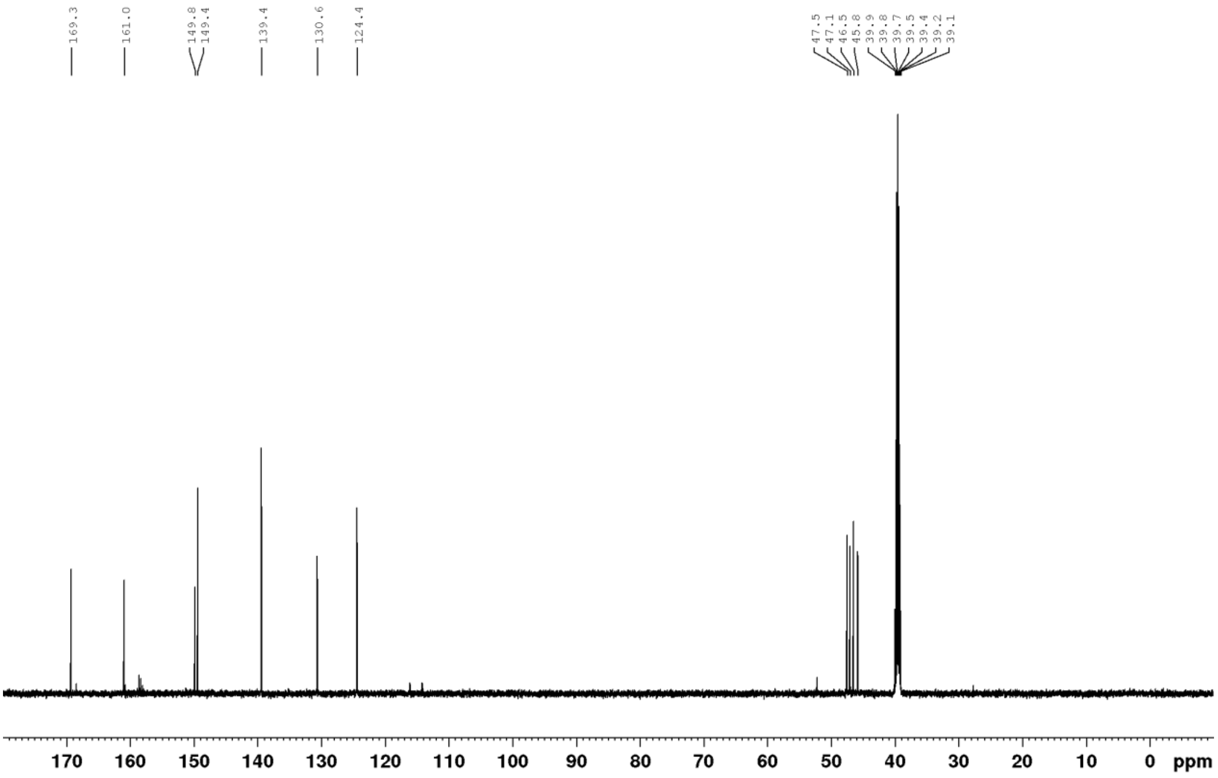

^{15}N NMR – 61 MHz

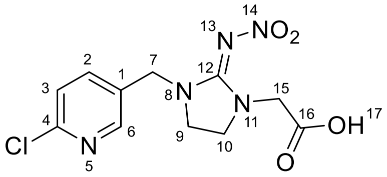

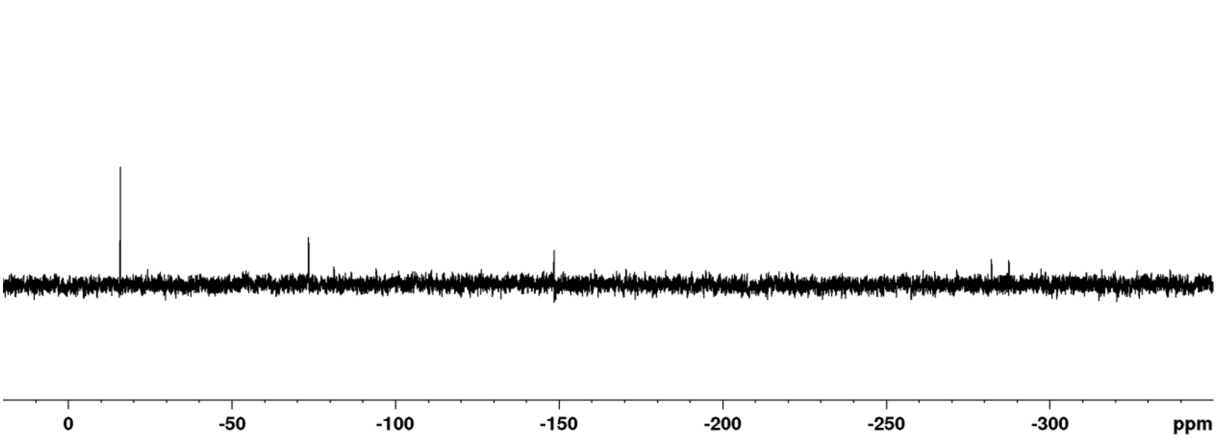

4-{[3-[(6-Chloropyridin-3-yl)methyl]-2-(nitroimino)imidazolidin-1-yl]methyl}benzoic acid (8b)

^{1}H NMR – 600 MHz

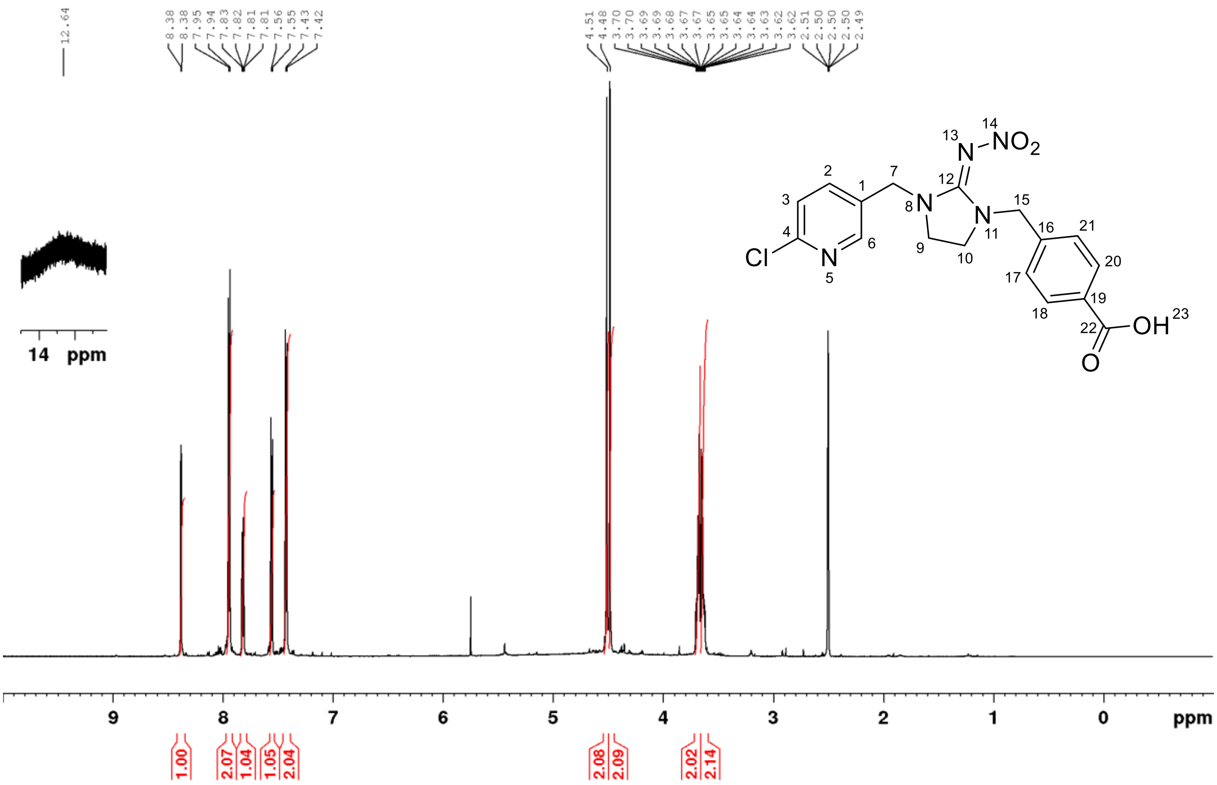

^{13}C NMR – 150 MHz

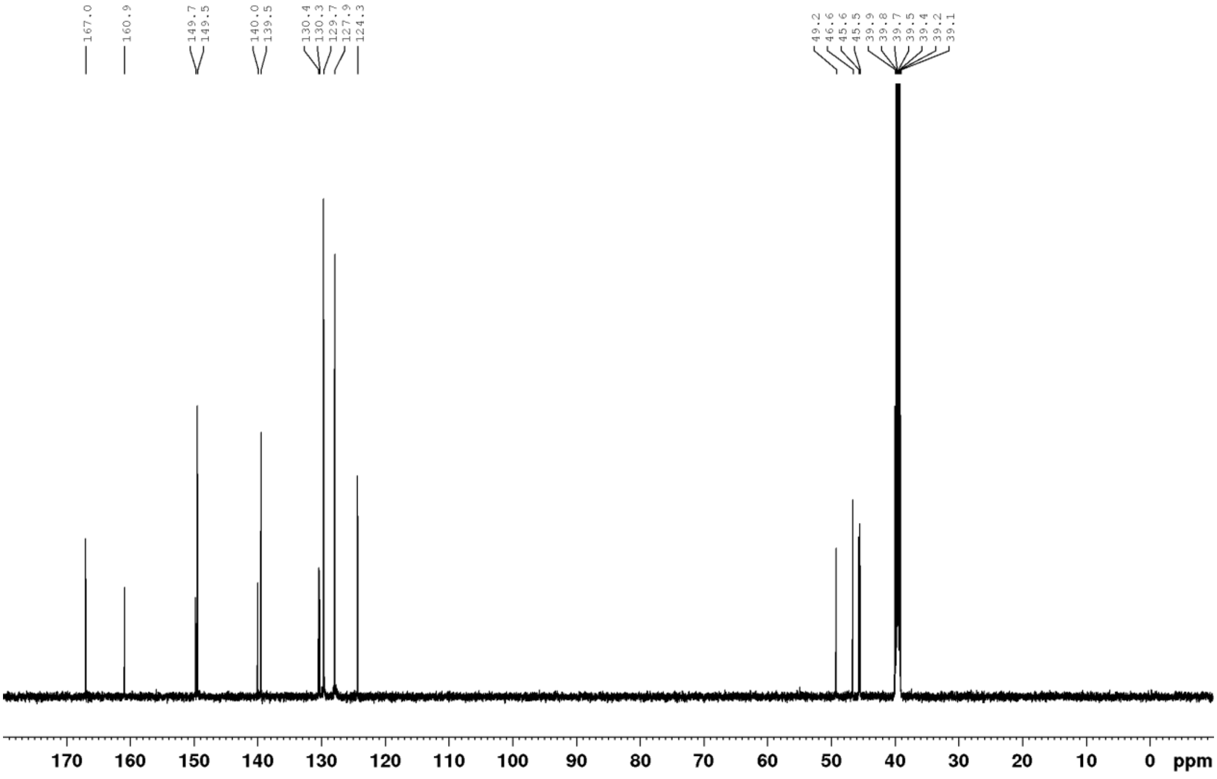

4-({5-[3-[(6-Chloropyridin-3-yl)methyl]-2-(nitroimino)imidazolidin-1-yl]pentyl}oxy)benzoic acid (9b)

^{1}H NMR – 600 MHz

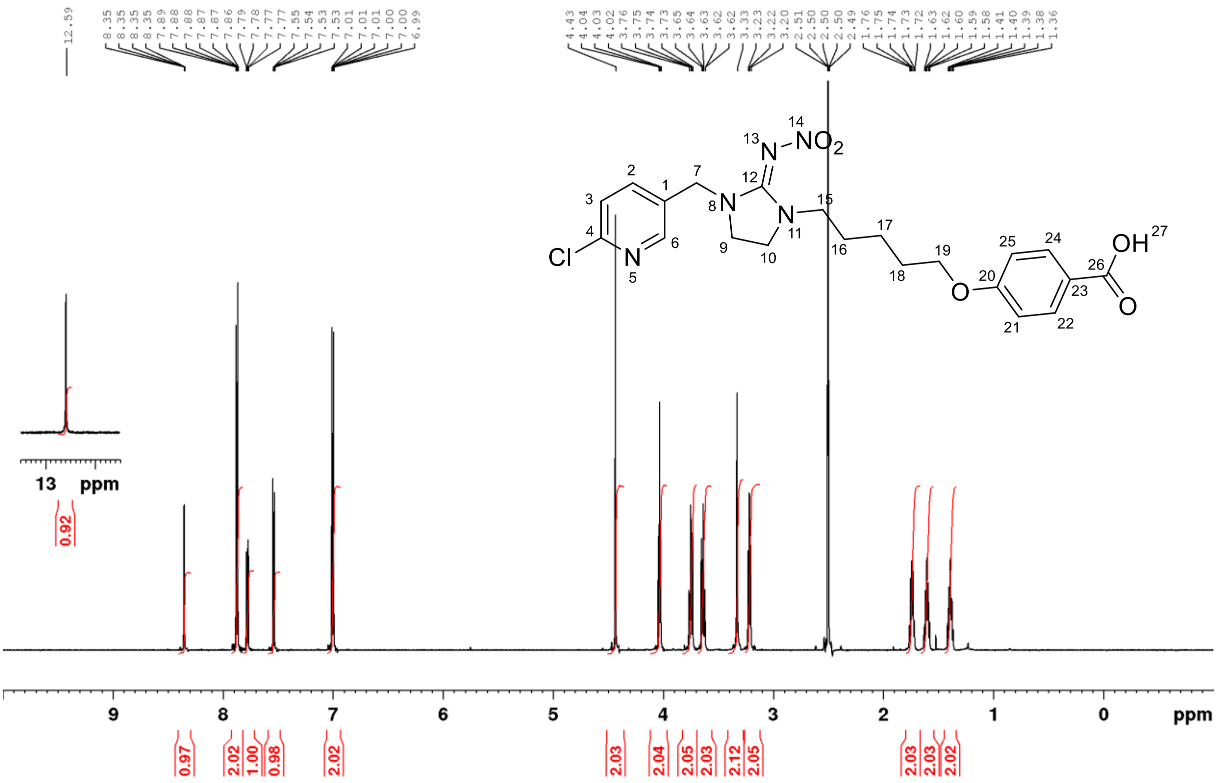

^{13}C NMR – 150 MHz

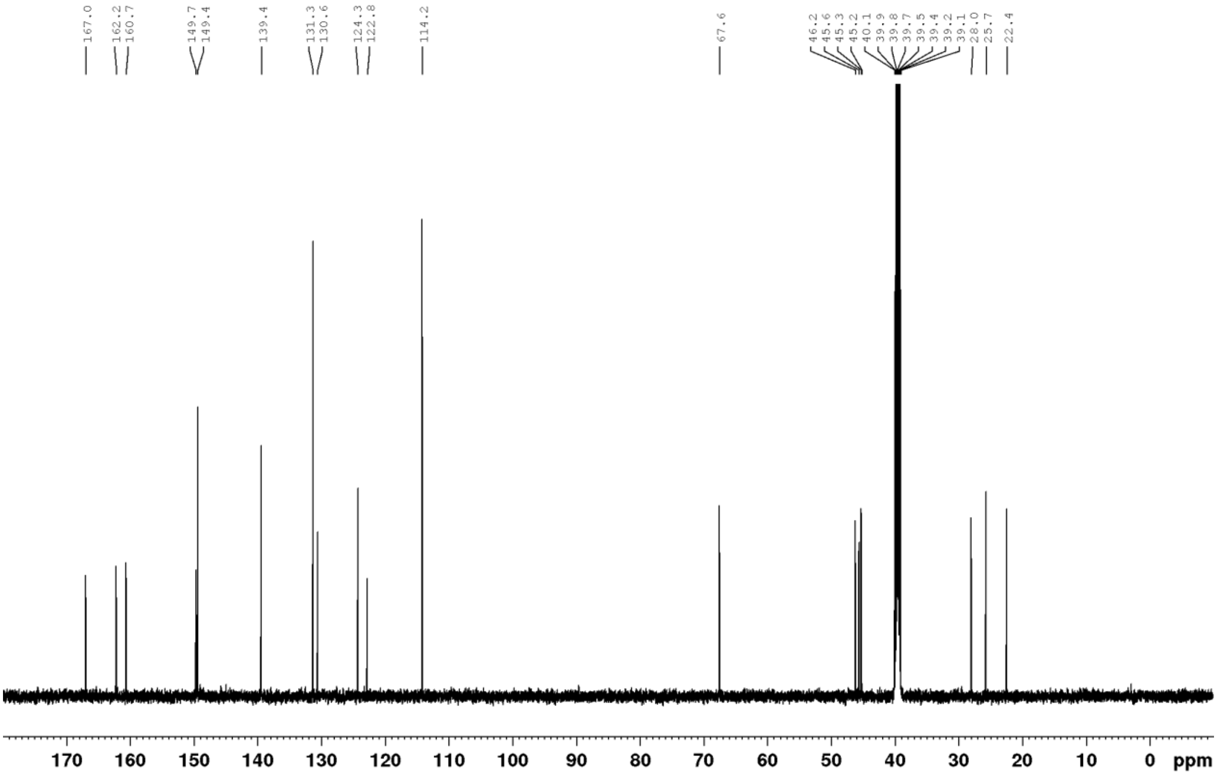

3-Chloro-4-({5-[3-[(6-chloropyridin-3-yl)methyl]-2-(nitroimino)imidazolidin-1-yl]pentyl}oxy)benzoic acid (10b)

^{1}H NMR – 600 MHz

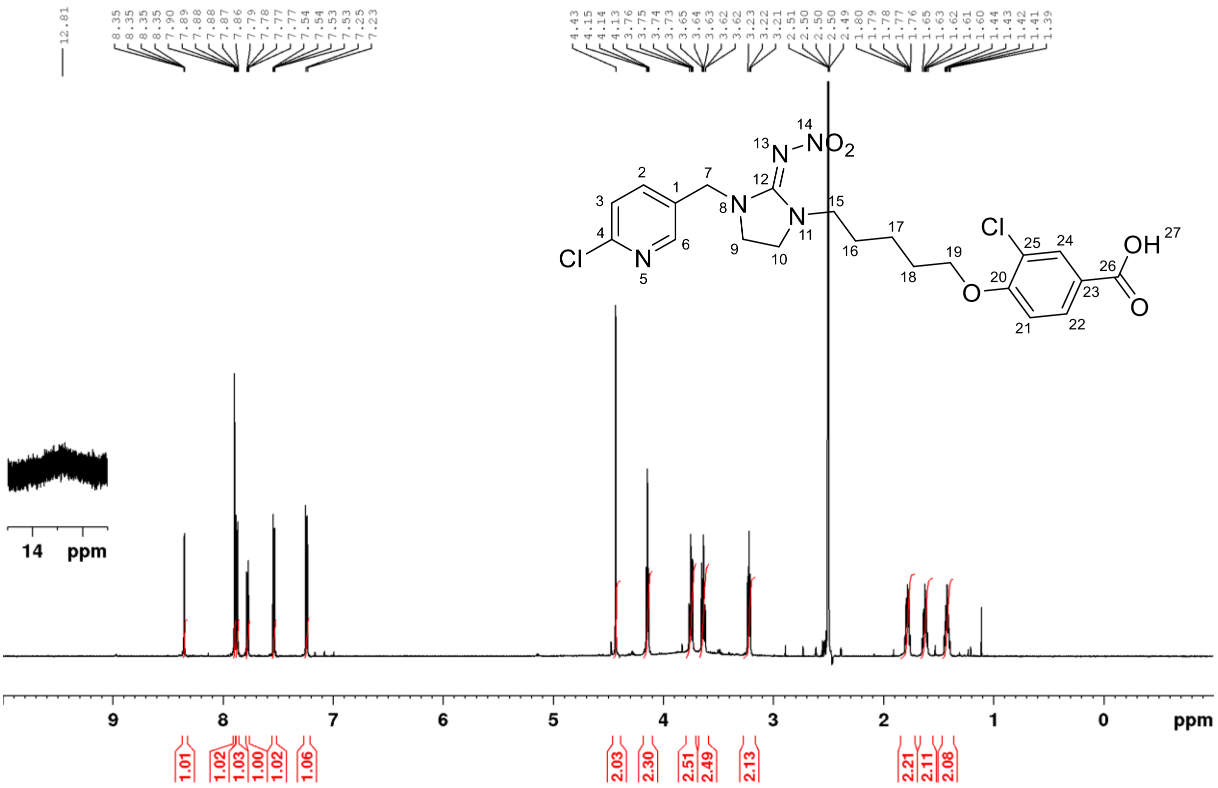

^{13}C NMR – 150 MHz

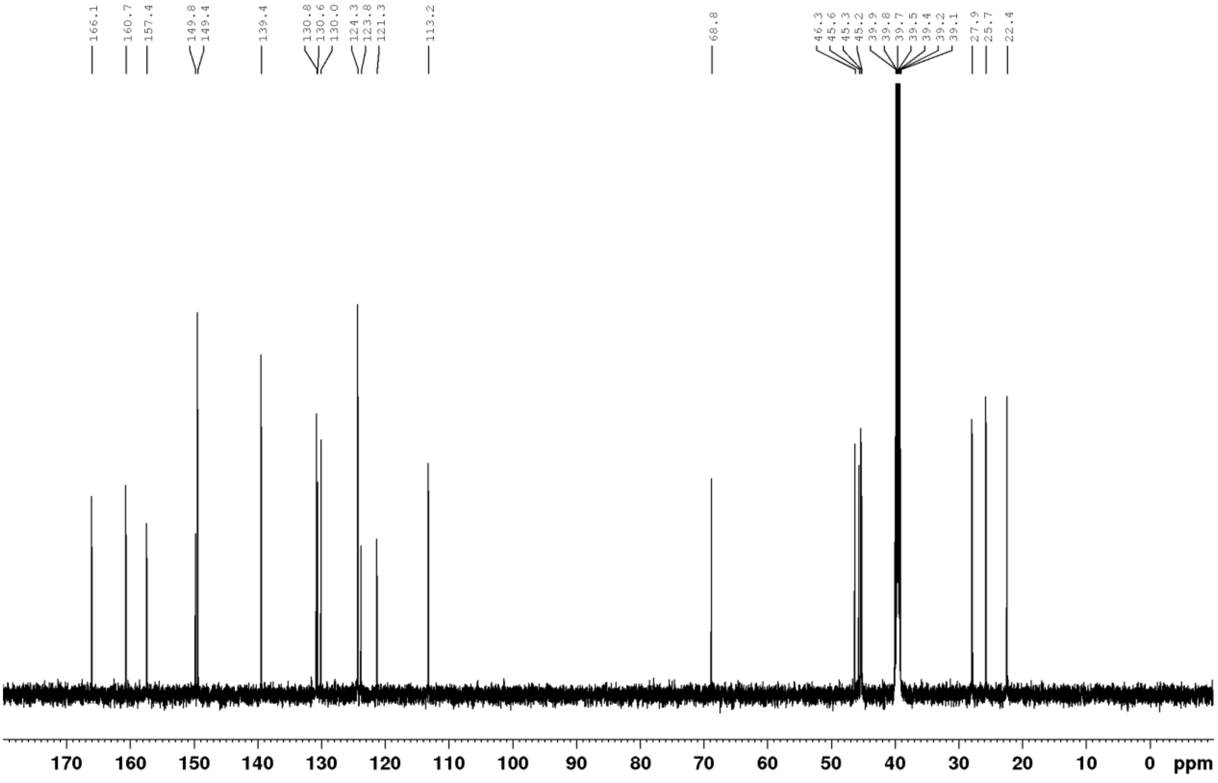

4-({5-[3-[(6-Chloropyridin-3-yl)methyl]-2-(nitroimino)imidazolidin-1-yl]pentyl}-oxy)-3-nitrobenzoic acid (11b)

^{1}H NMR – 600 MHz

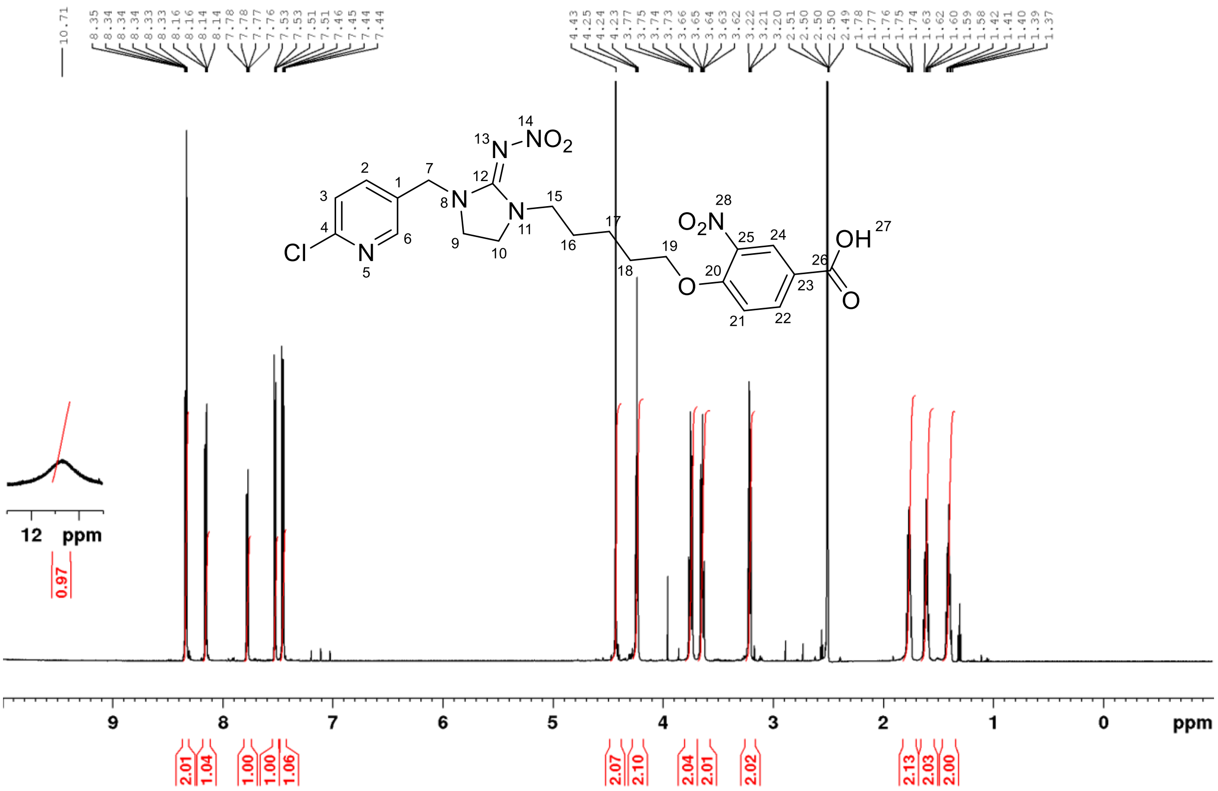

^{13}C NMR – 150 MHz

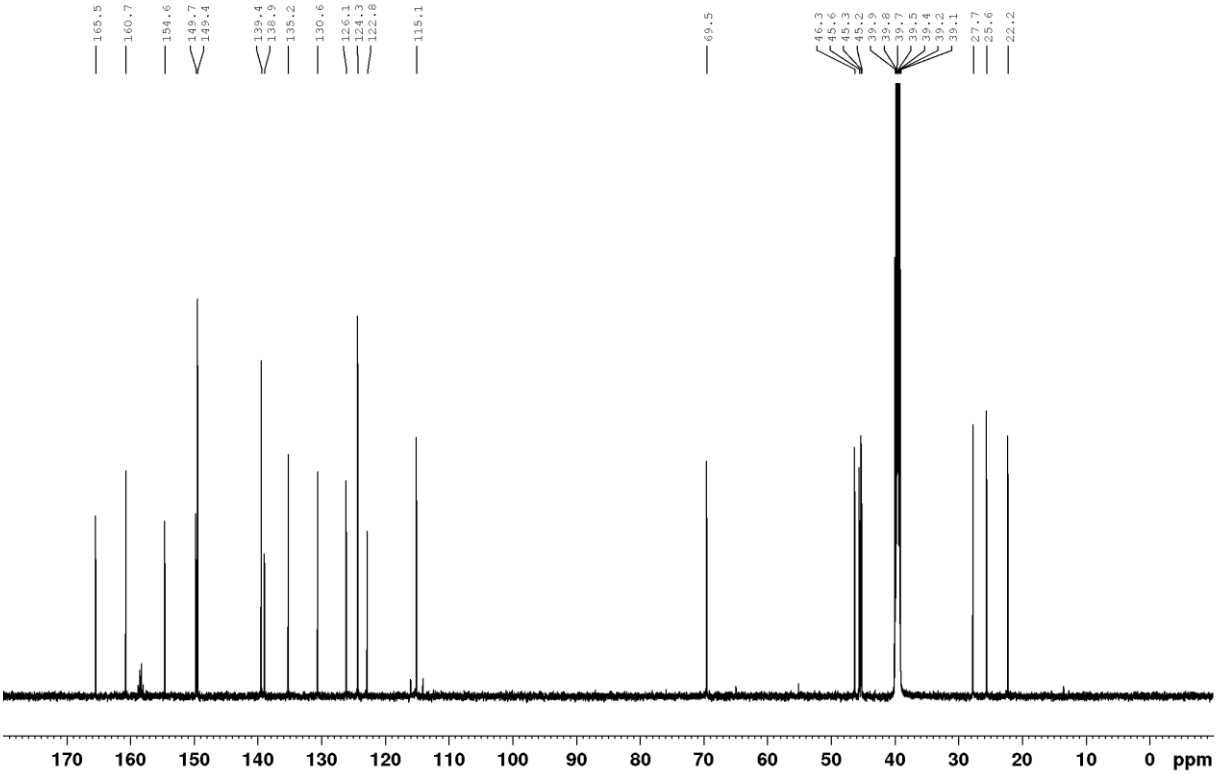

4-{[1,1-Dichloro-3-{1-[(6-chloropyridin-3-yl)methyl]imidazolidin-2-ylidene}-3-nitroprop-1-en-2-yl]sulfanyl}benzoic acid (12b)

^{1}H NMR – 600 MHz

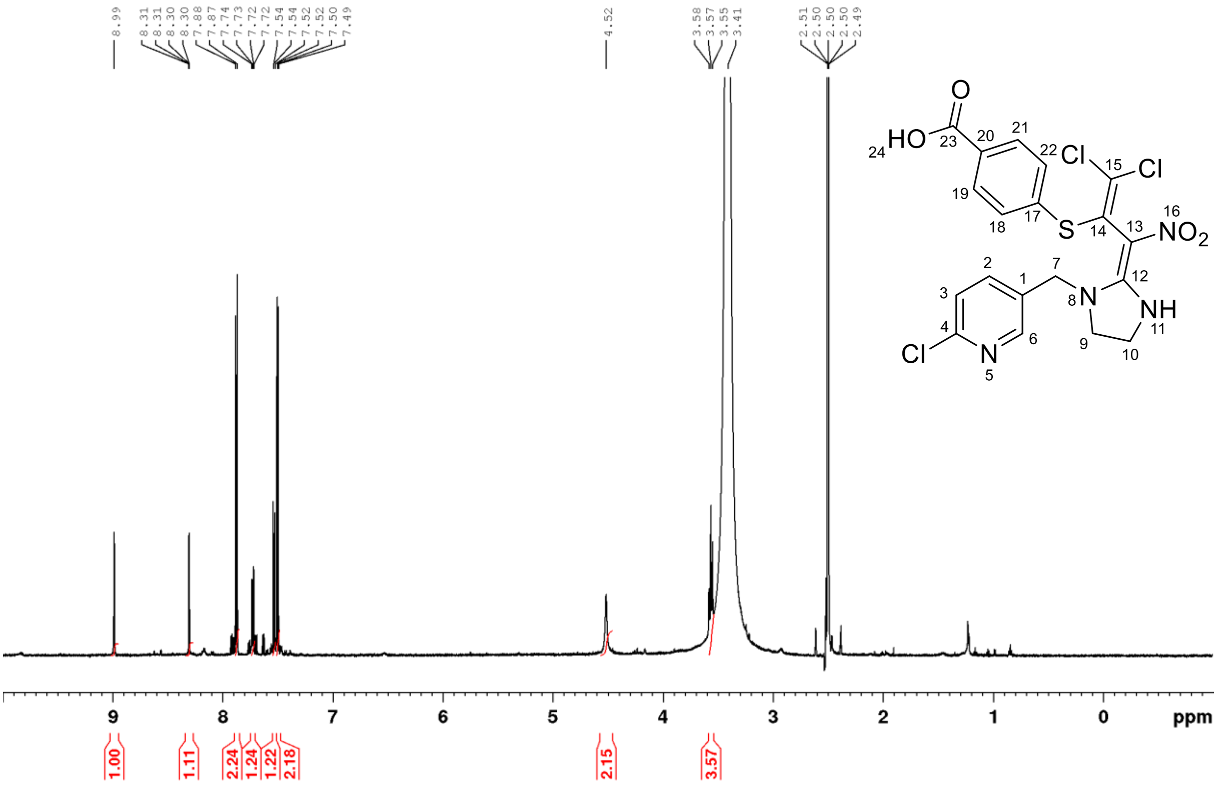

^{13}C NMR – 150 MHz

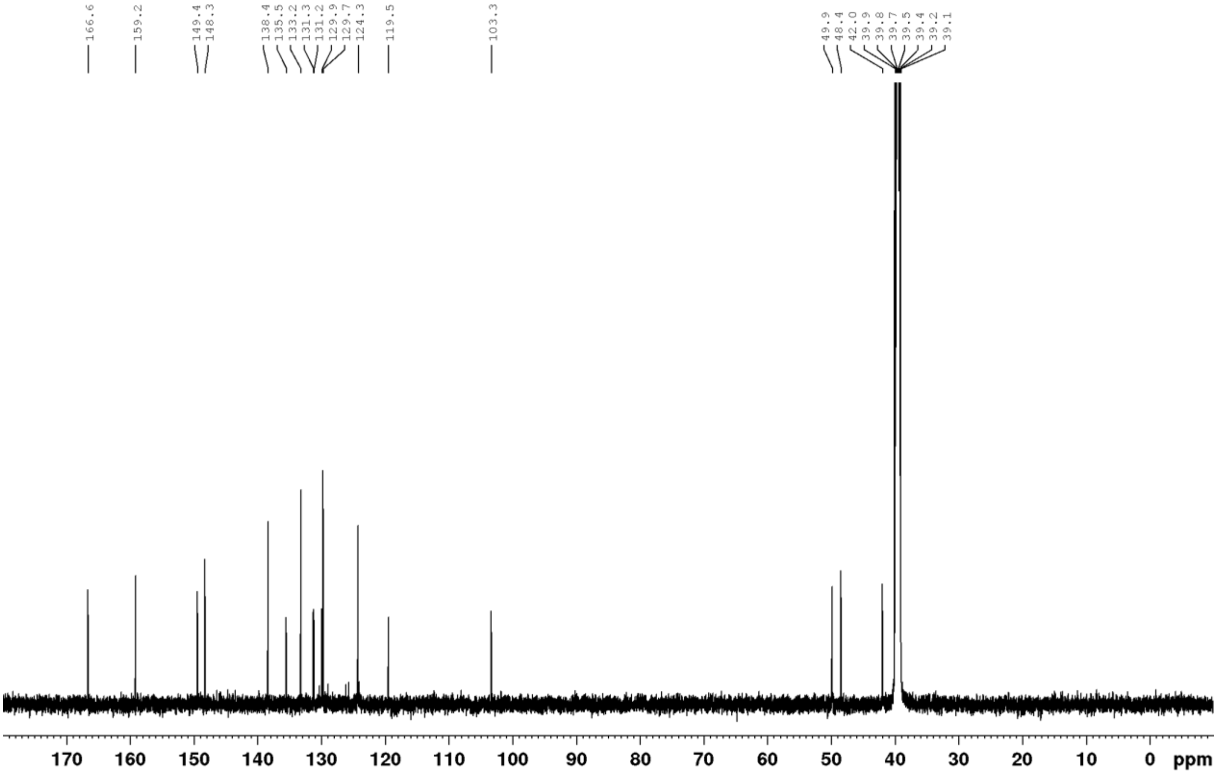

***N*-{1-[2-(1*H*-Benzotriazol-1-yl)-2-oxoethyl]-3-[(6-chloropyridin-3-yl)methyl]imidazolidin-2-ylidene}nitramide (7c)**

^{1}H NMR – 600 MHz

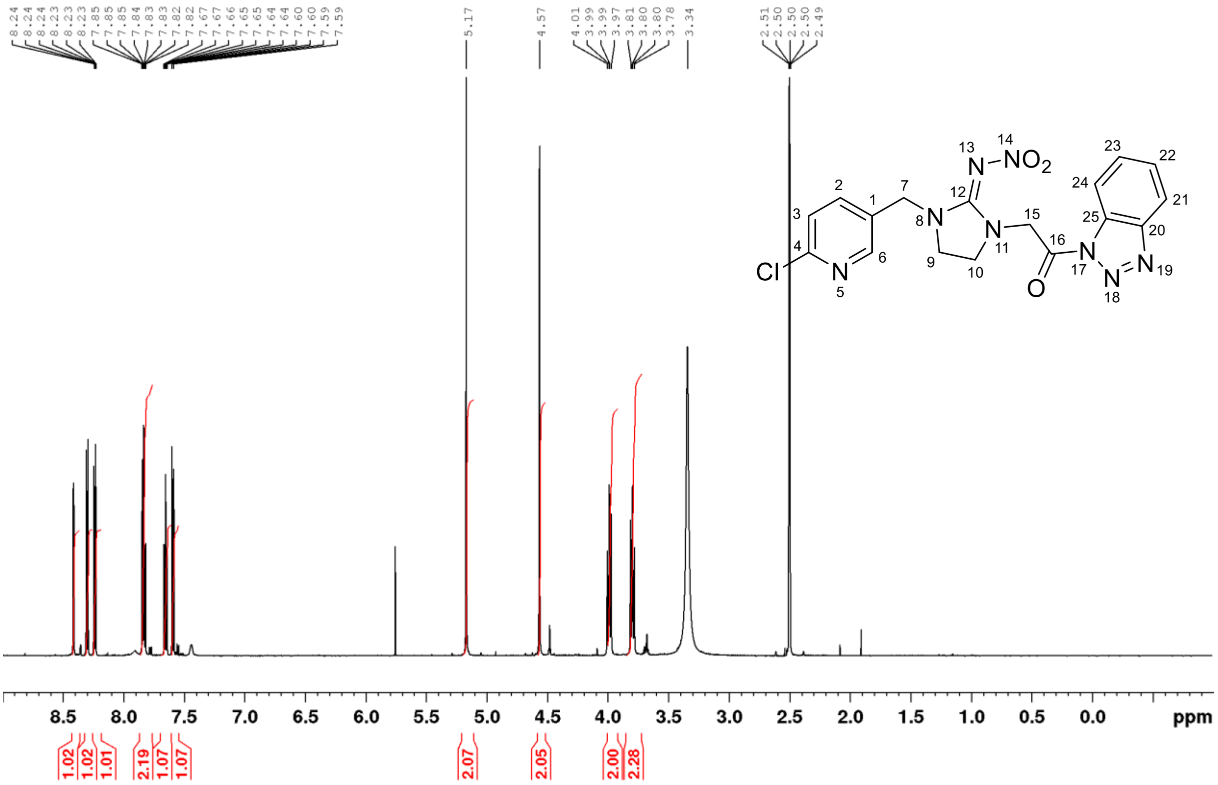

^{13}C NMR – 150 MHz

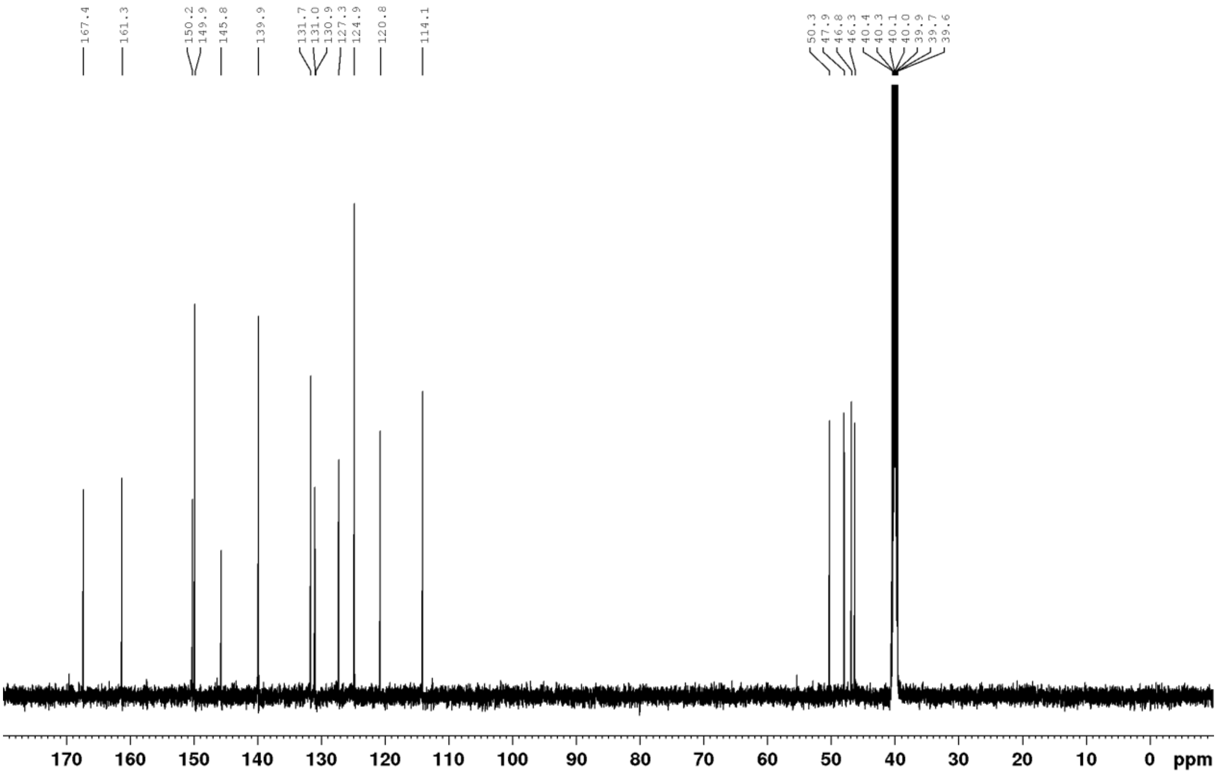

^{15}N NMR – 61 MHz (^{1}H,^{15}N HMBC)

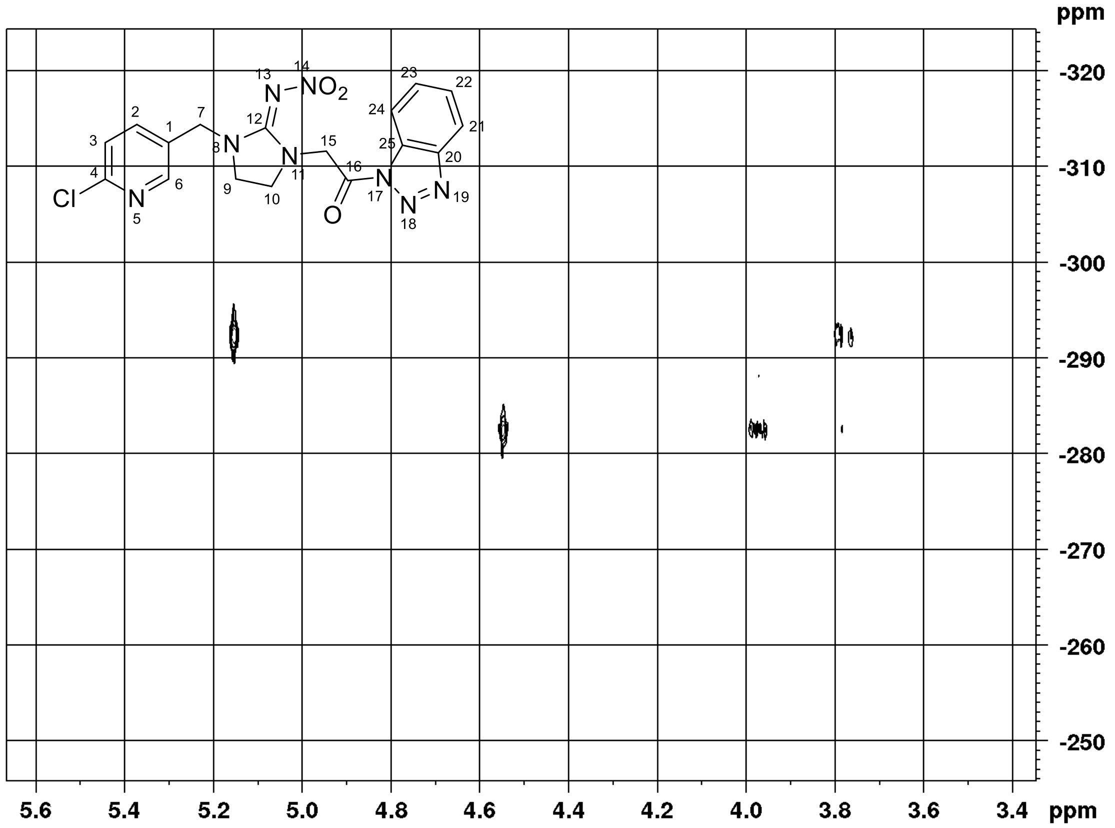

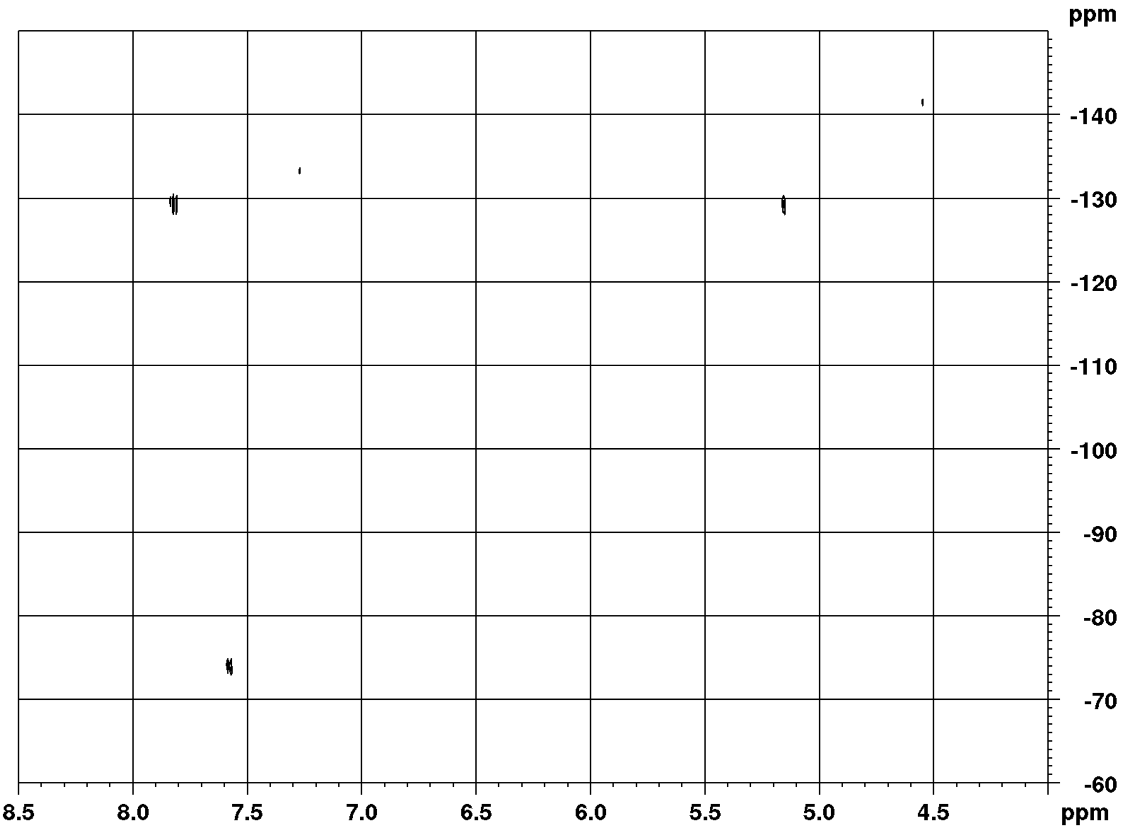

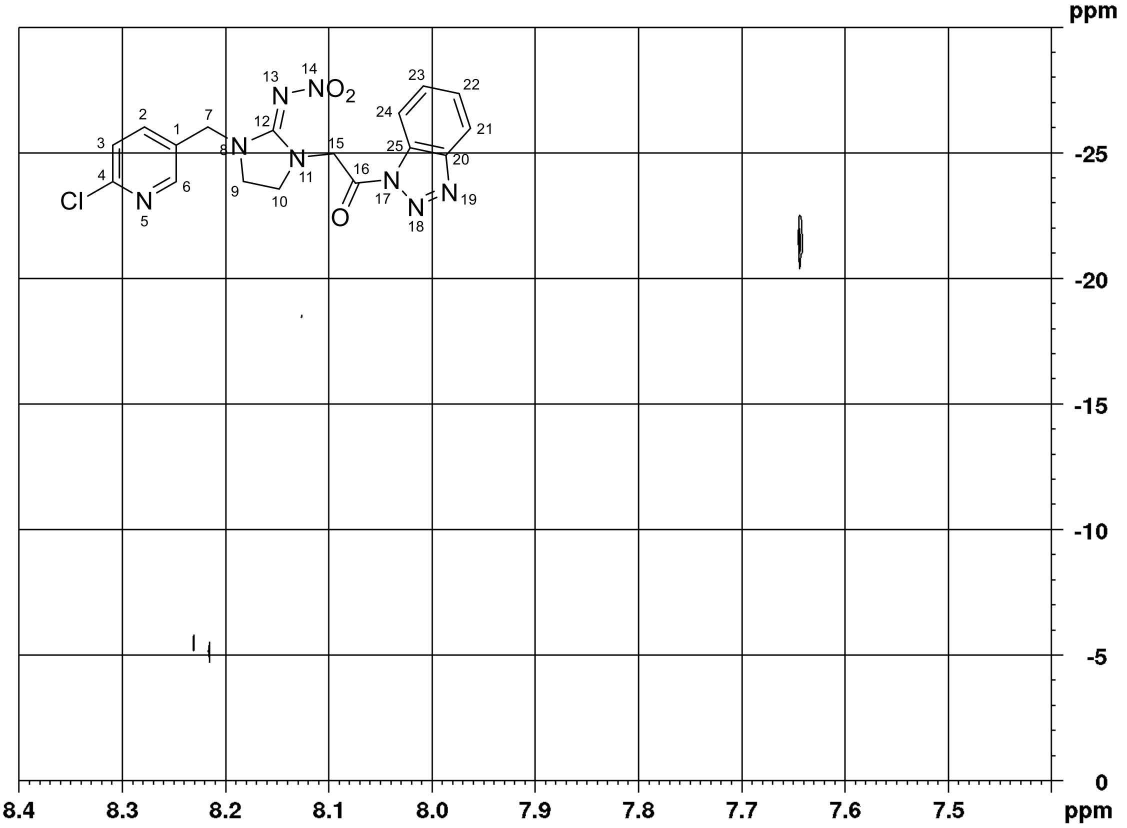

N-14 could not be detected due to insufficient solubility of the substance.

***N*-(1-{[4-(1*H*-Benzotriazole-1-carbonyl)phenyl]methyl}-3-[(6-chloropyridin-3-yl)methyl]imidazolidin-2-ylidene)nitramide (8c)**

^{1}H NMR – 600 MHz

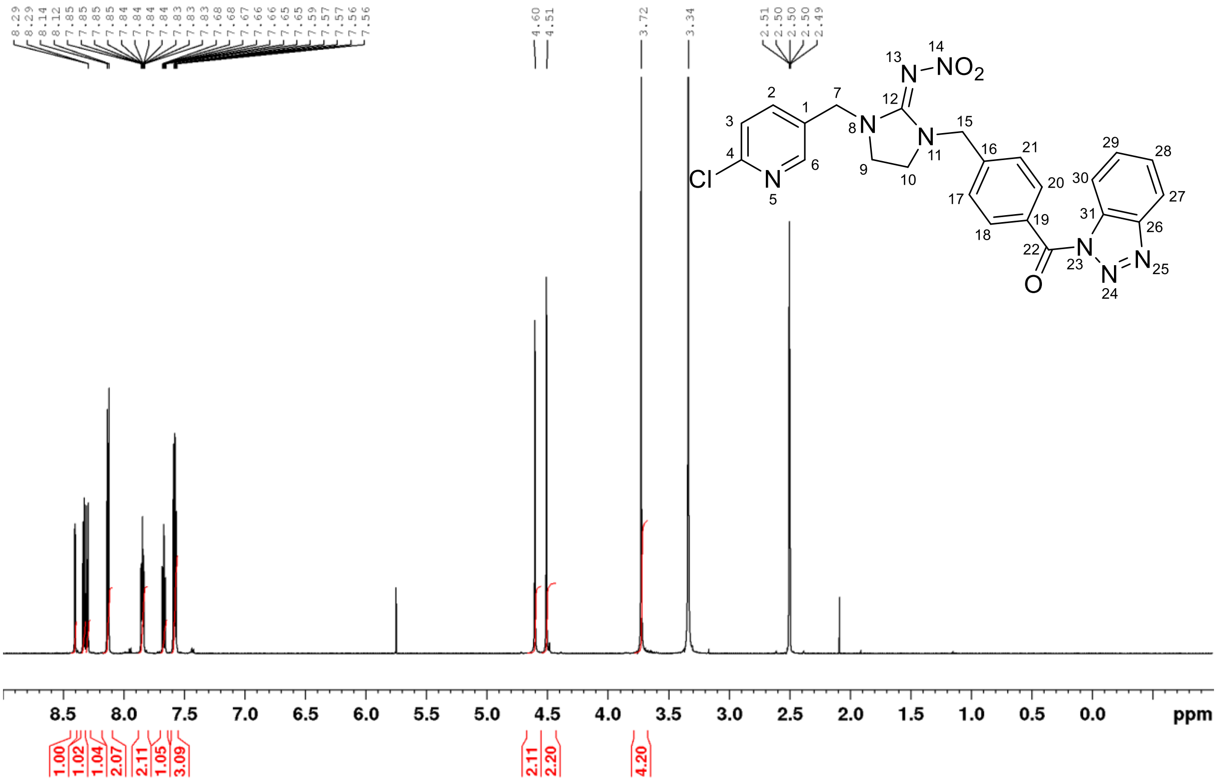

^{13}C NMR – 150 MHz

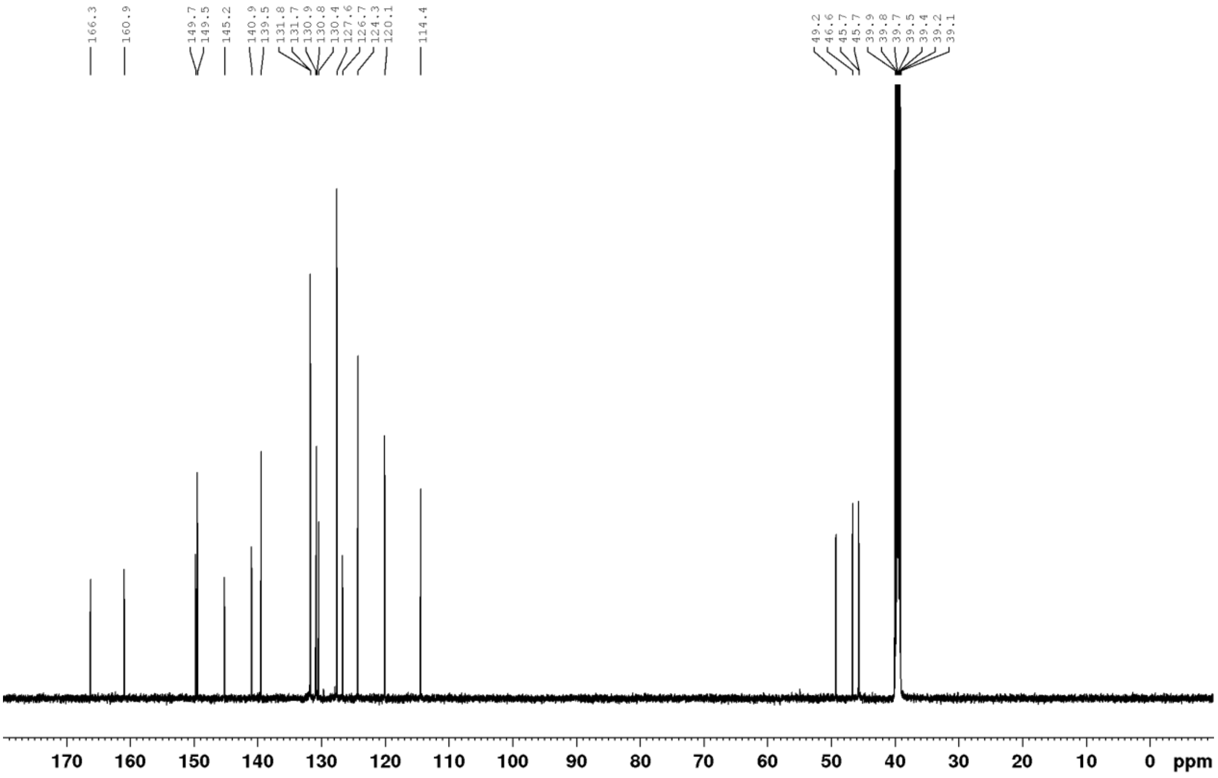

^{15}N NMR – 61 MHz (^{1}H,^{15}N HMBC)

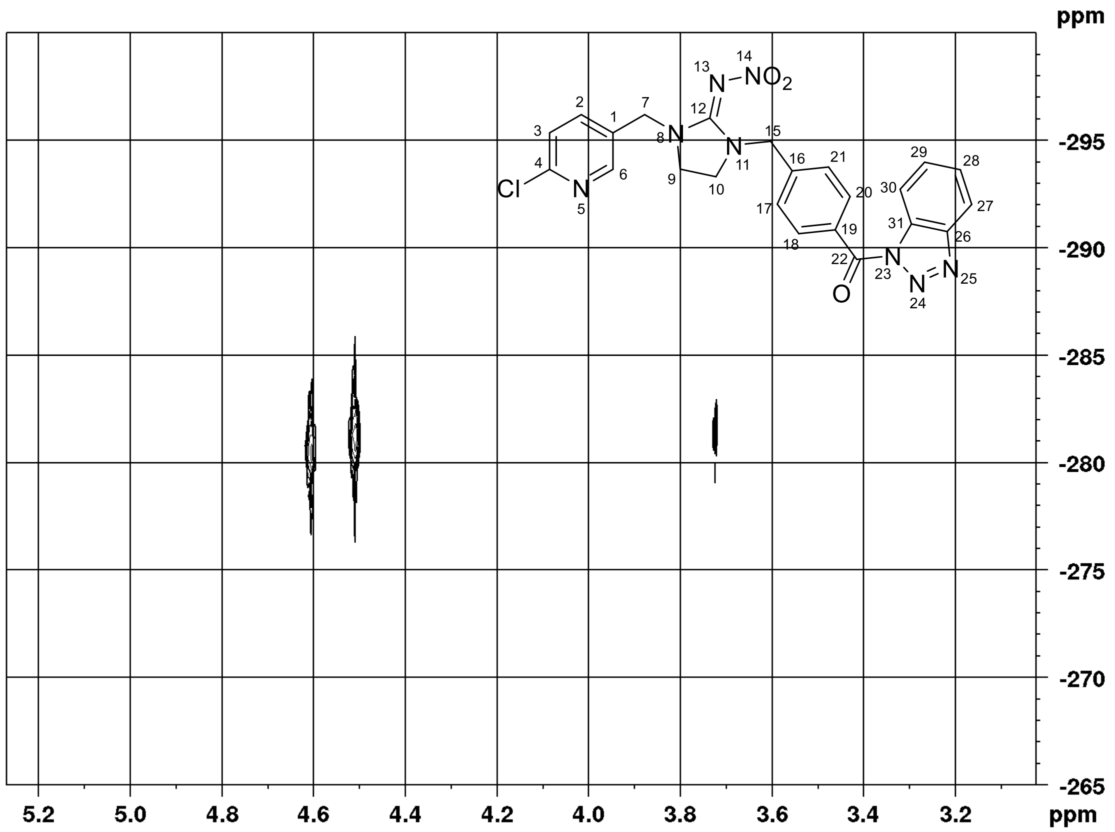

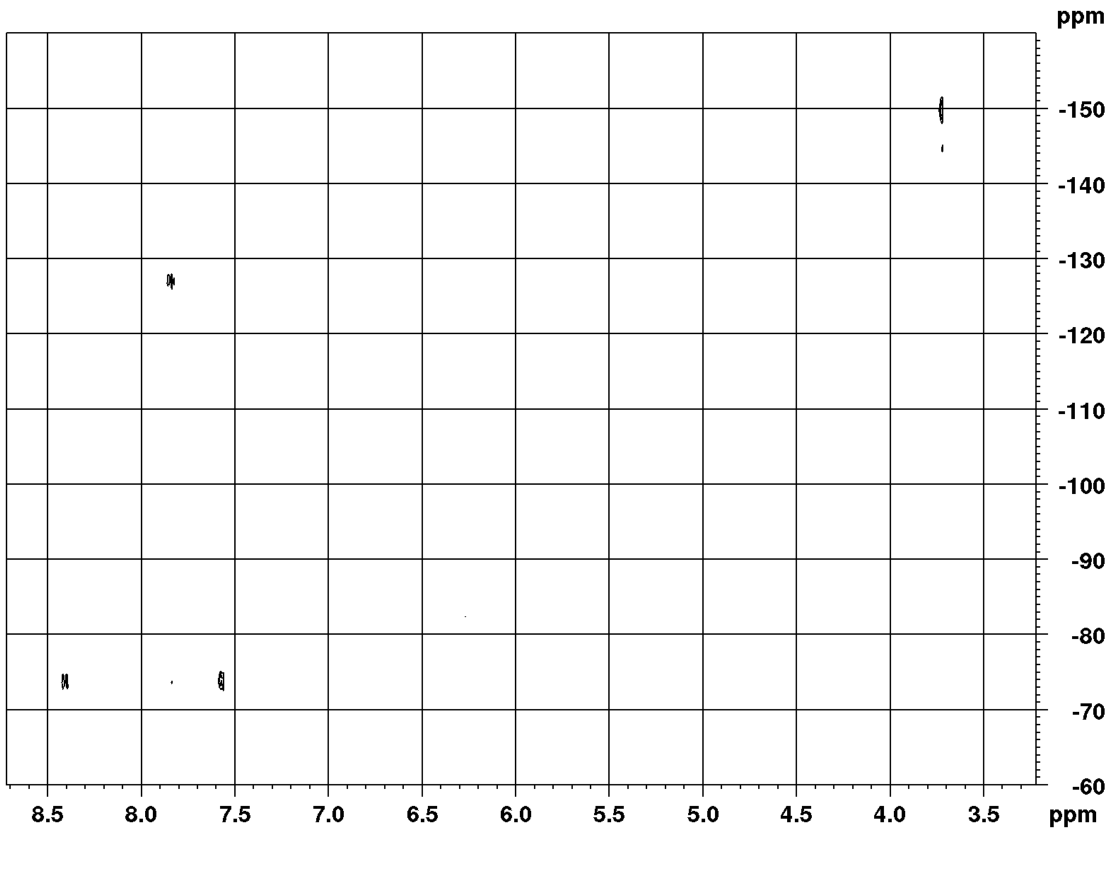

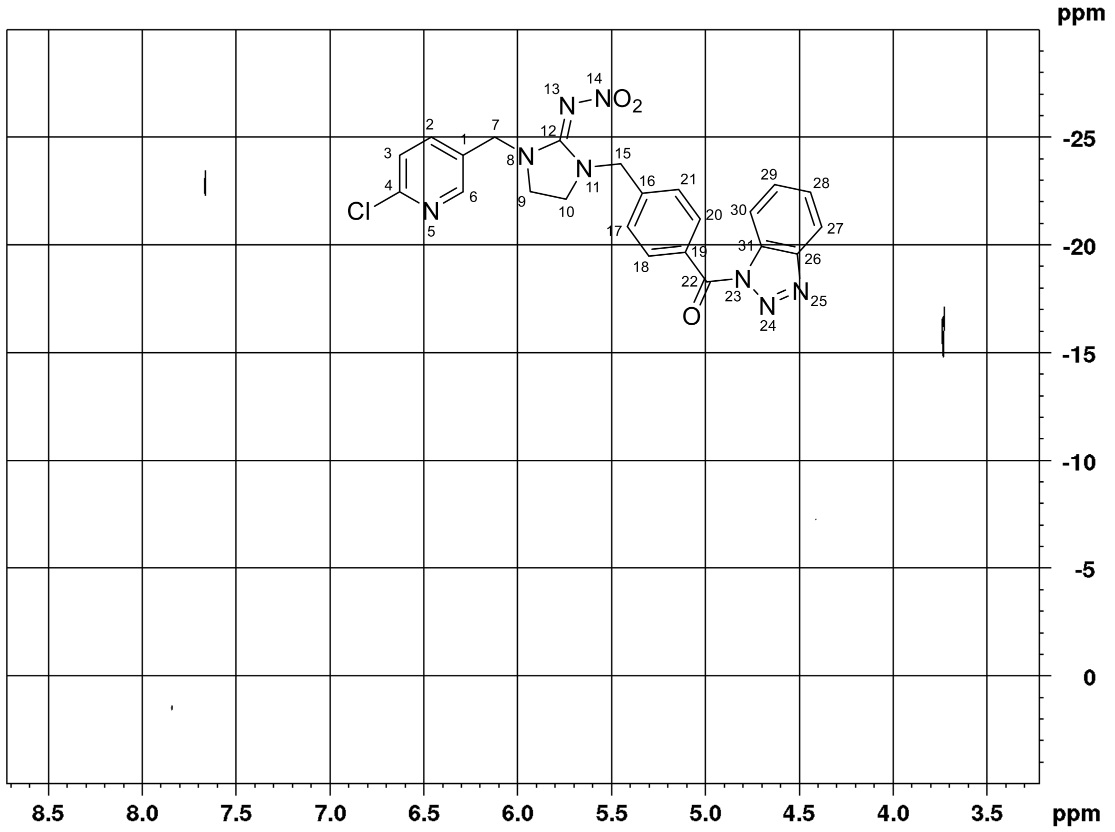
ppm
-25
-20
-15
-10
-5
0
8.5
8.0
7.5
7.0
6.5
6.0
5.5
5.0
4.5
4.0
3.5
ppm

_N_-(1-{5-[4-(1_H_-Benzotriazole-1-carbonyl)phenoxy]pentyl}-3-[(6-chloropyridin-3-yl)methyl]imidazolidin-2-ylidene)nitramide (9c)

^{1}H NMR – 600 MHz

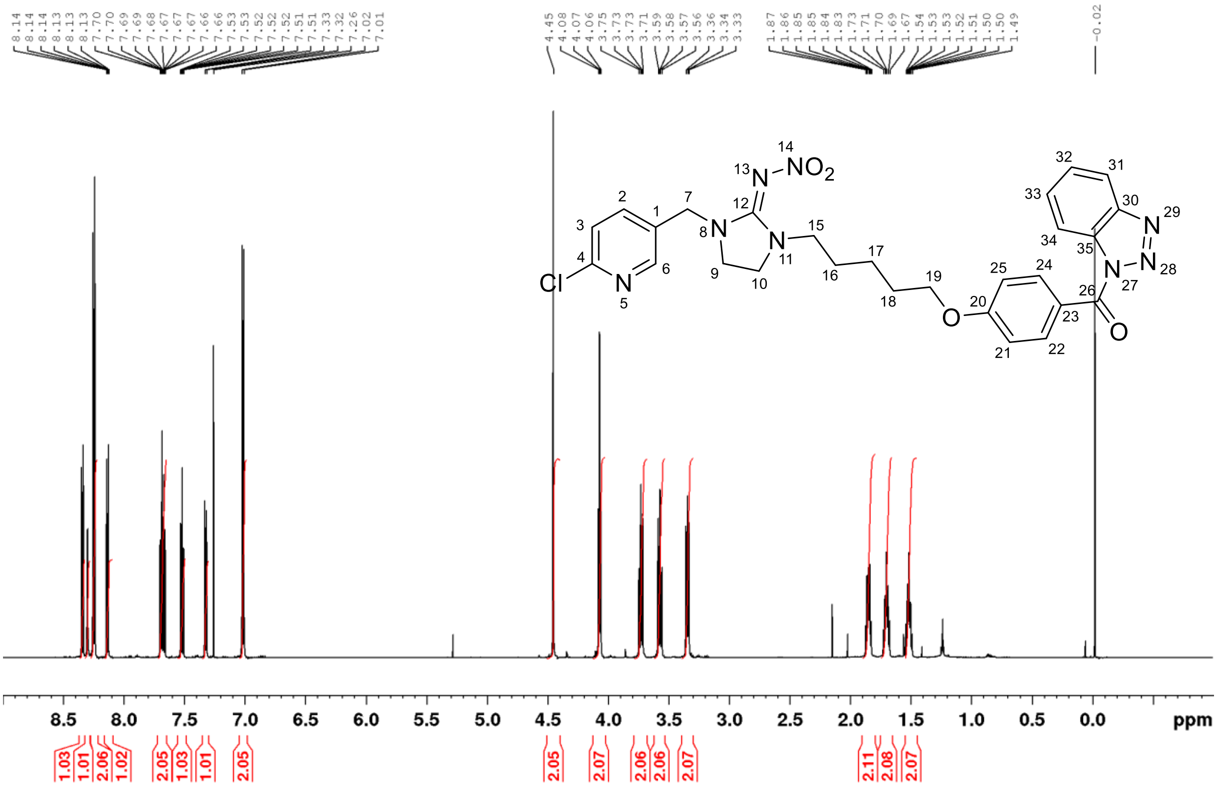

^{13}C NMR – 150 MHz

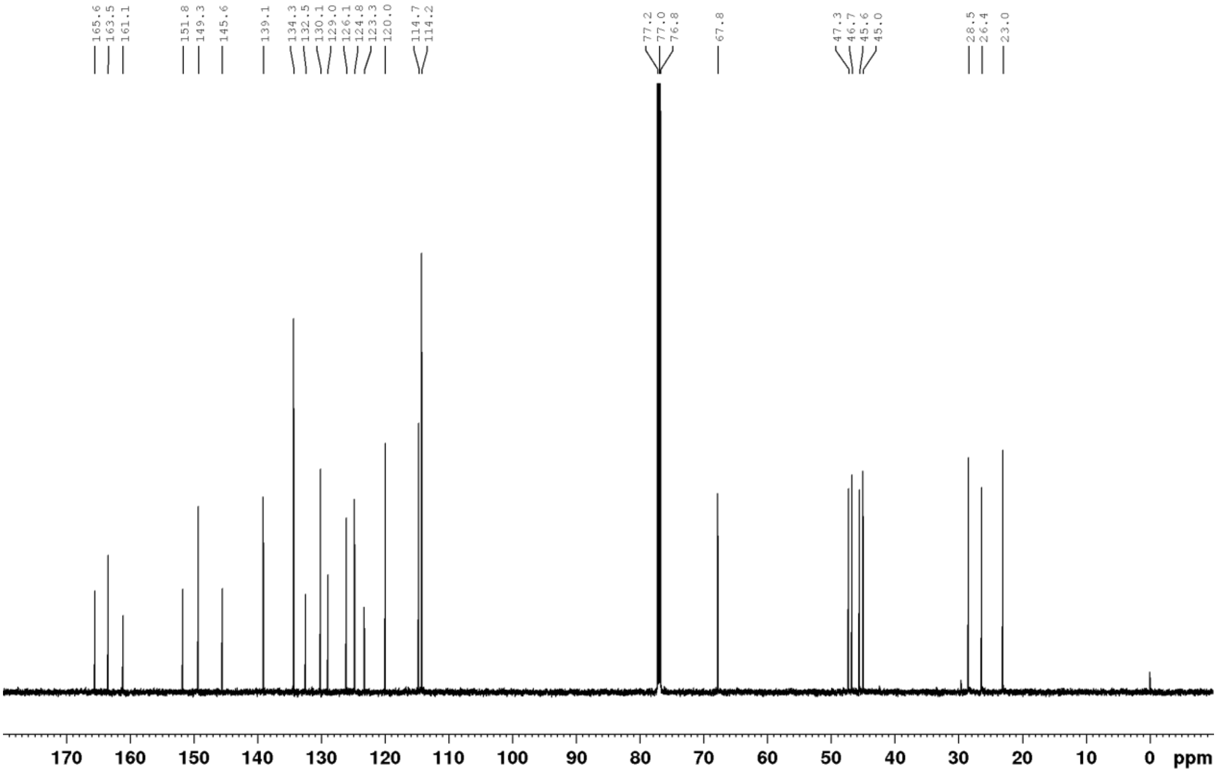

^{15}N NMR – 61 MHz

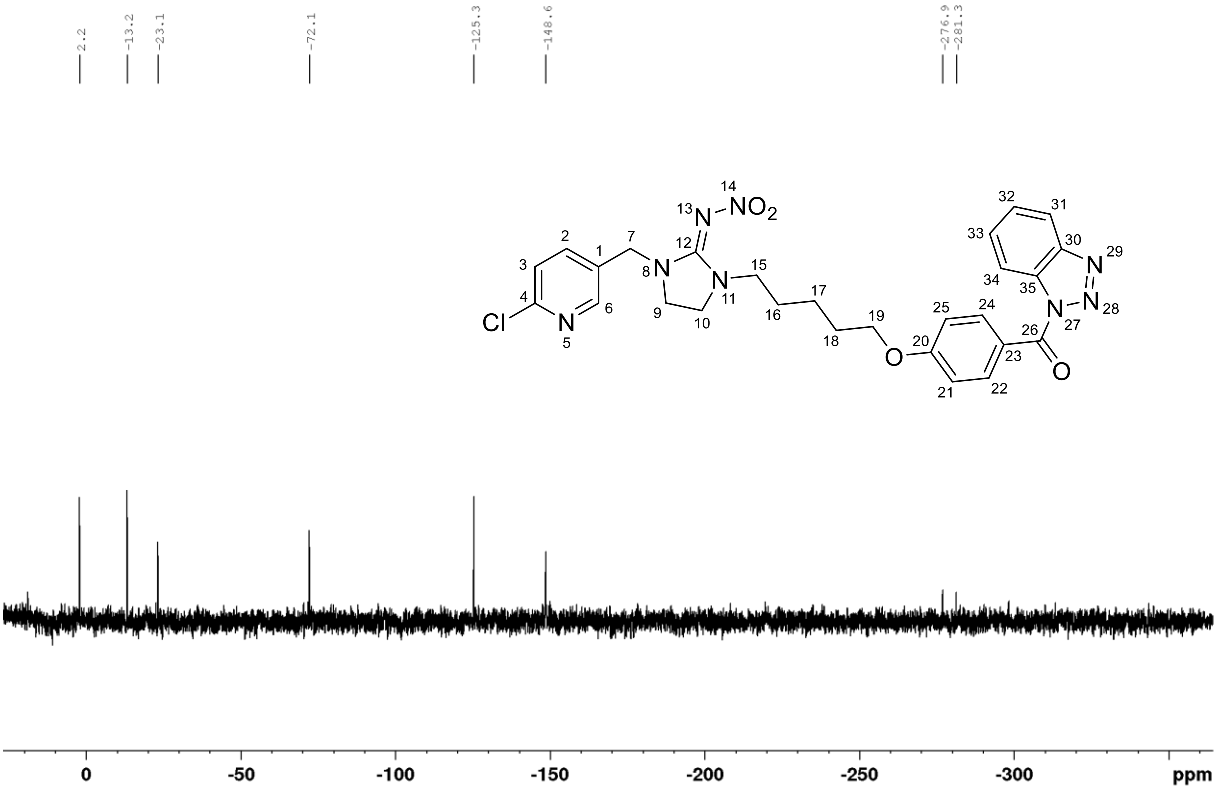

_N_-(1-{5-[4-(1_H_-Benzotriazole-1-carbonyl)-2-chlorophenoxy]pentyl}-3-[(6-chloropyridin-3-yl)methyl]imidazolidin-2-ylidene)nitramide (10c)

^{1}H NMR – 600 MHz

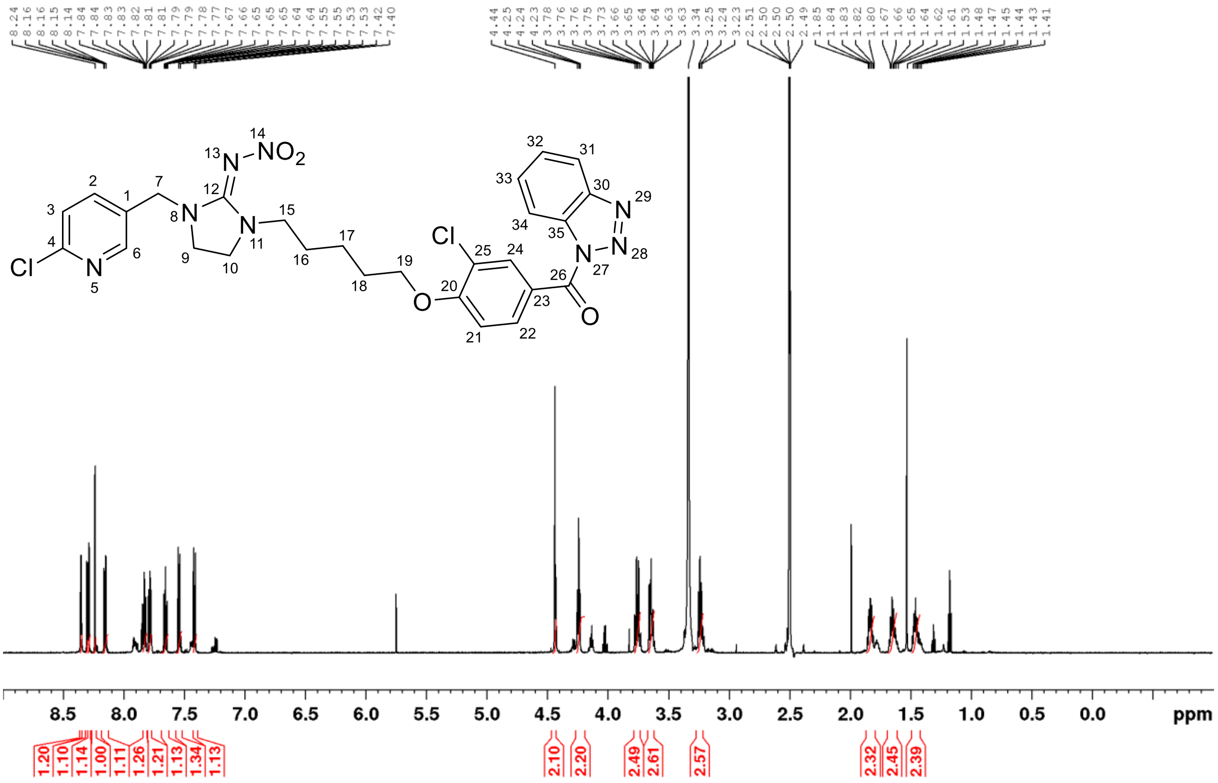

^{13}C NMR – 150 MHz

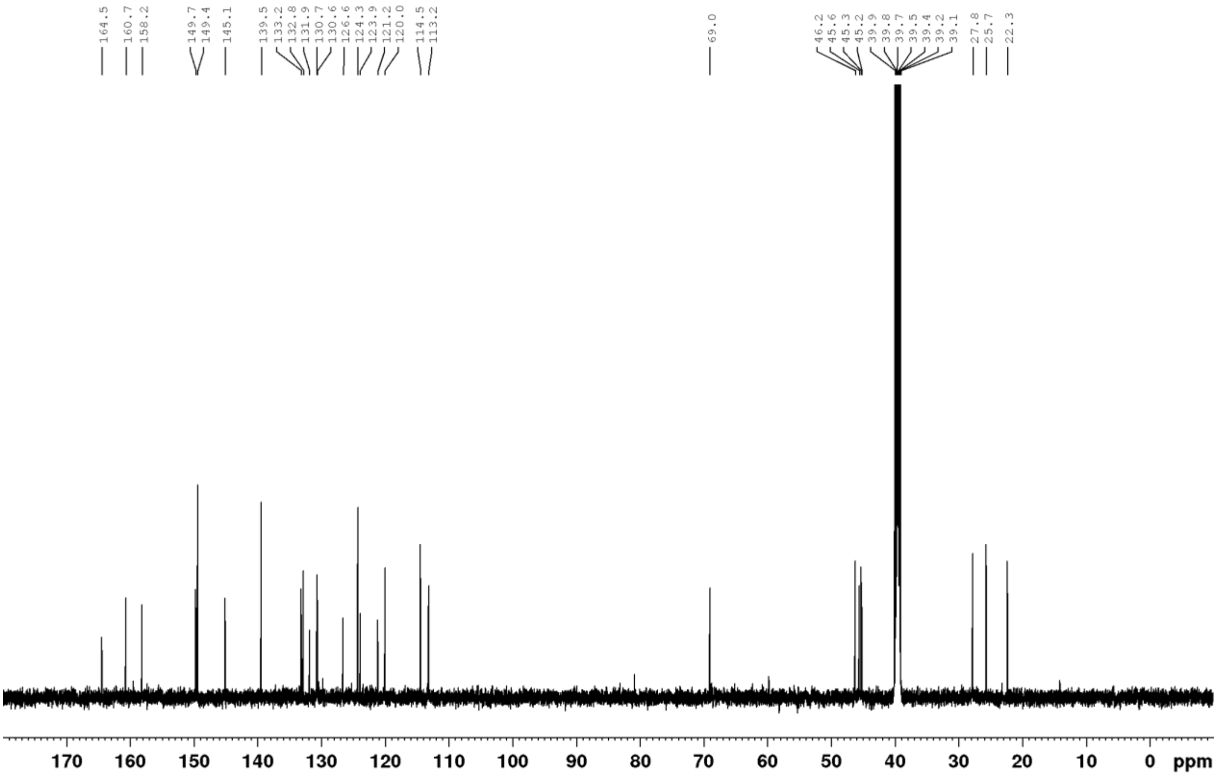

_N_-(1-{5-[4-(1_H_-Benzotriazole-1-carbonyl)-2-nitrophenoxy]pentyl}-3-[(6-chloropyridin-3-yl)methyl]imidazolidin-2-ylidene)nitramide (11c)

^{1}H NMR – 600 MHz

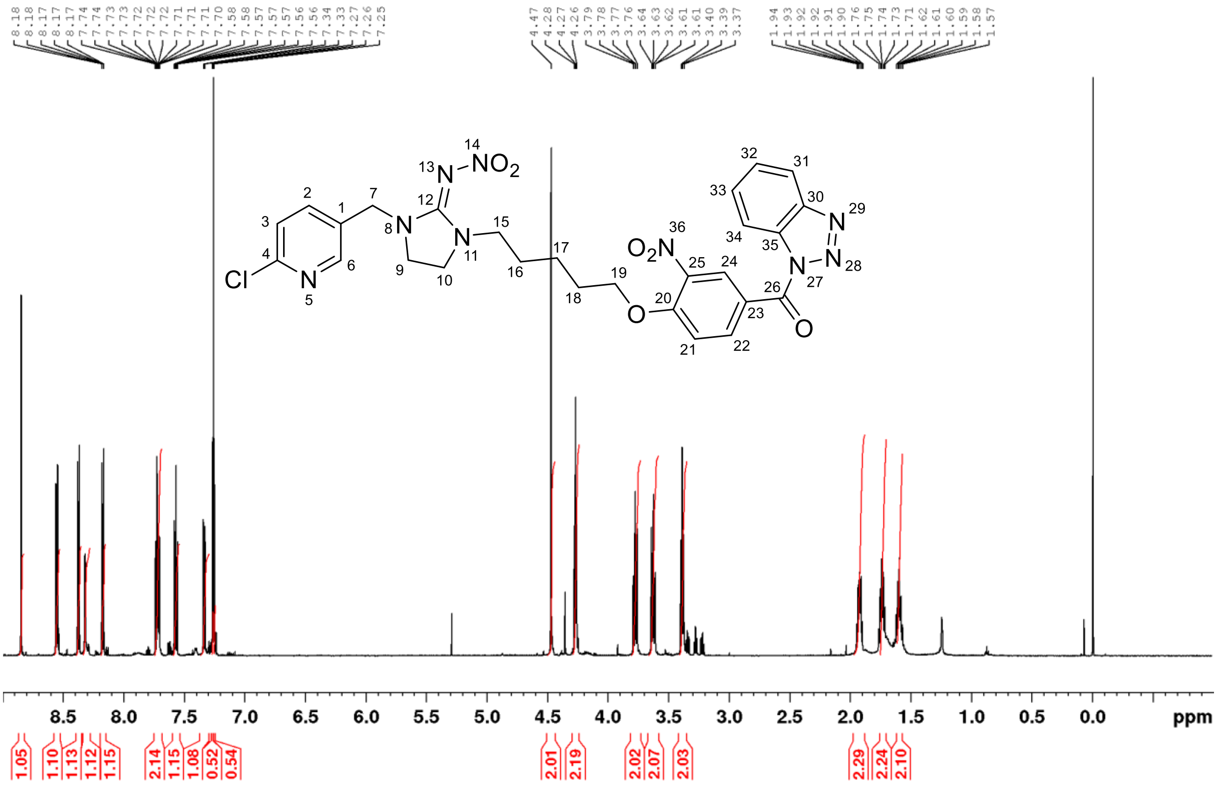

^{13}C NMR – 150 MHz

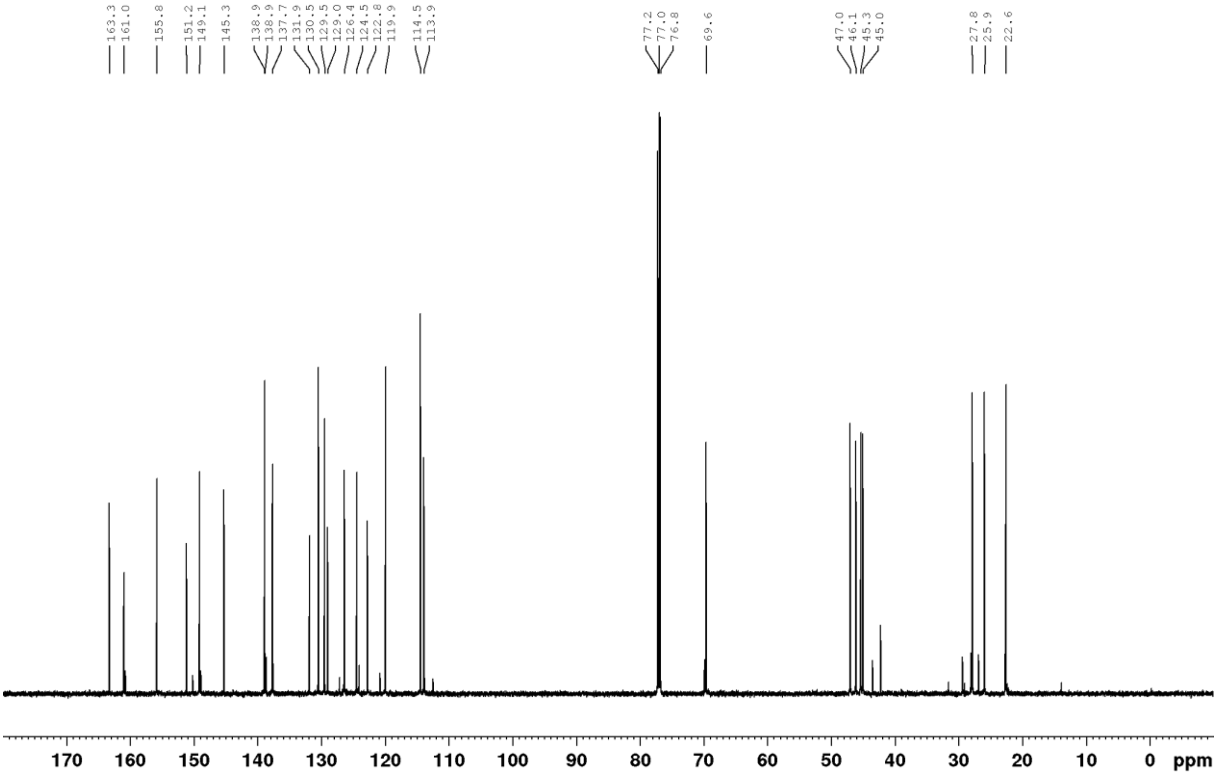

^{15}N NMR – 61 MHz

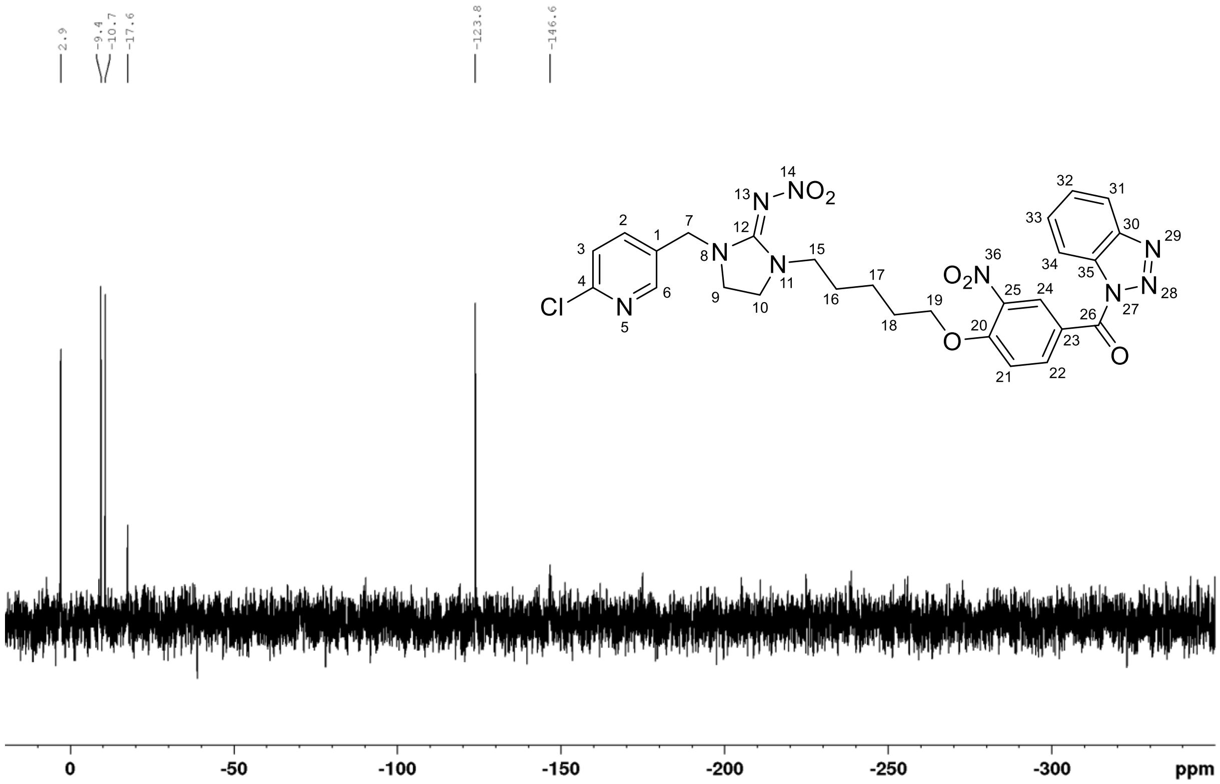

N-5, N-8 and N-11 were only detected via ^{1}H,^{15}N HMBC:

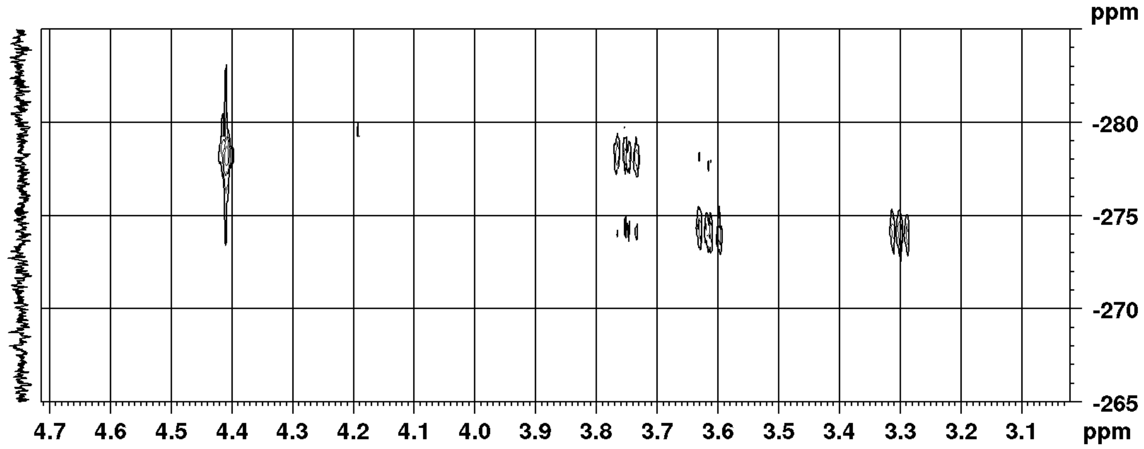

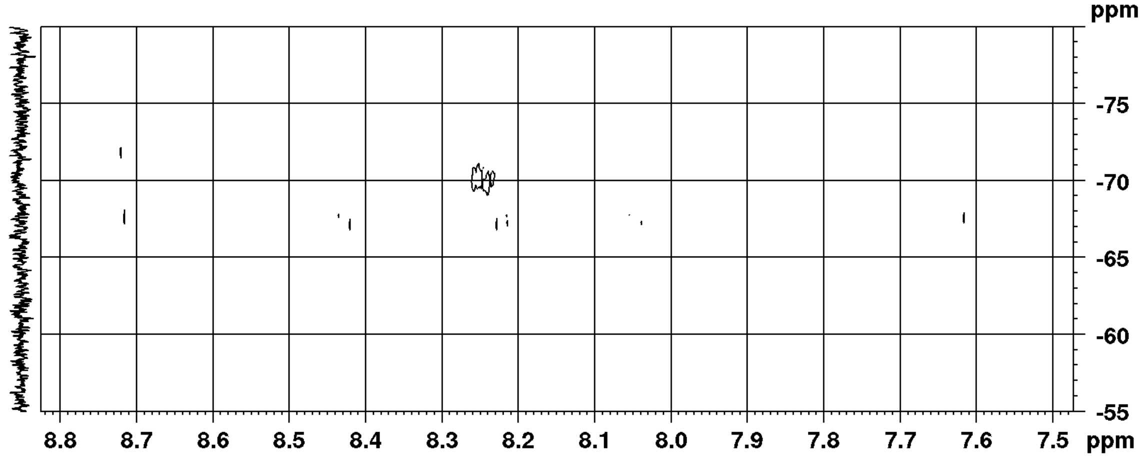

(1*H*-Benzotriazol-1-yl)(4-{[1,1-dichloro-3-{1-[(6-chloropyridin-3-yl)methyl]imidazolidin-2-ylidene}-3-nitroprop-1-en-2-yl]sulfanyl}phenyl)methanone (12c)

^{1}H NMR – 600 MHz

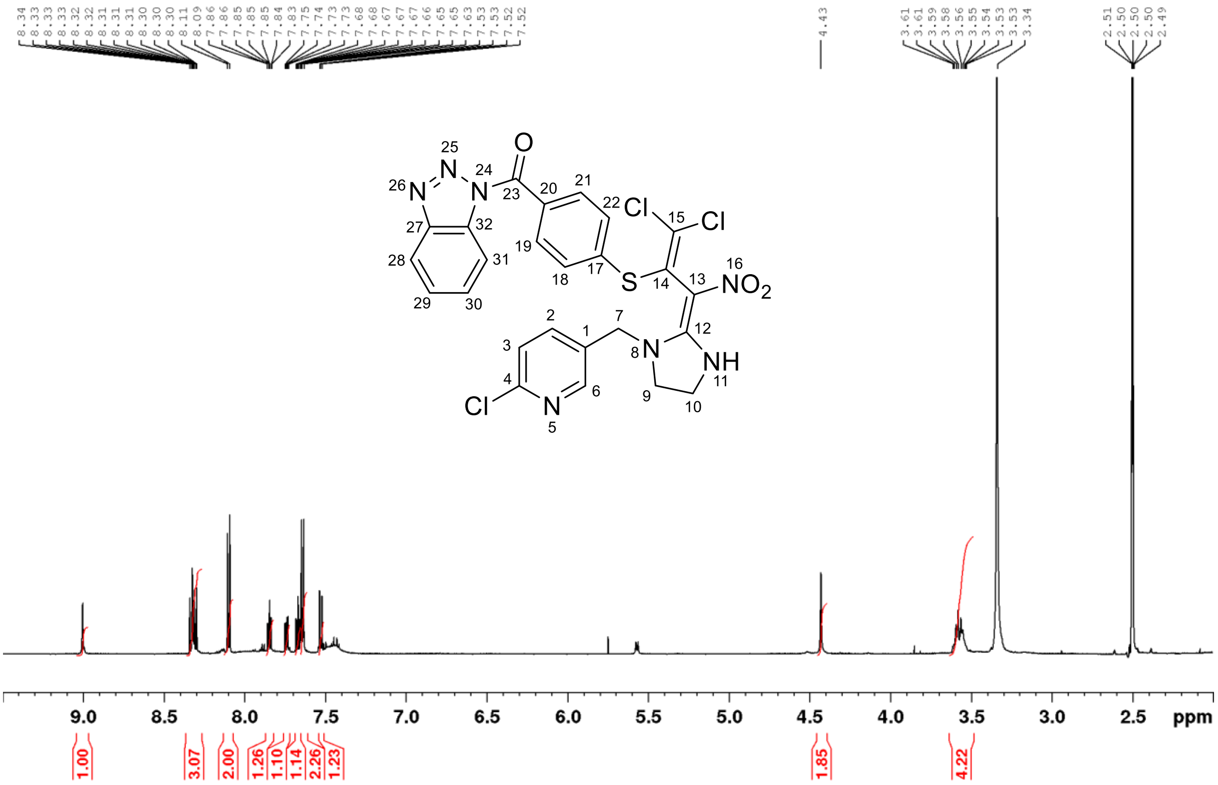

^{13}C NMR – 150 MHz

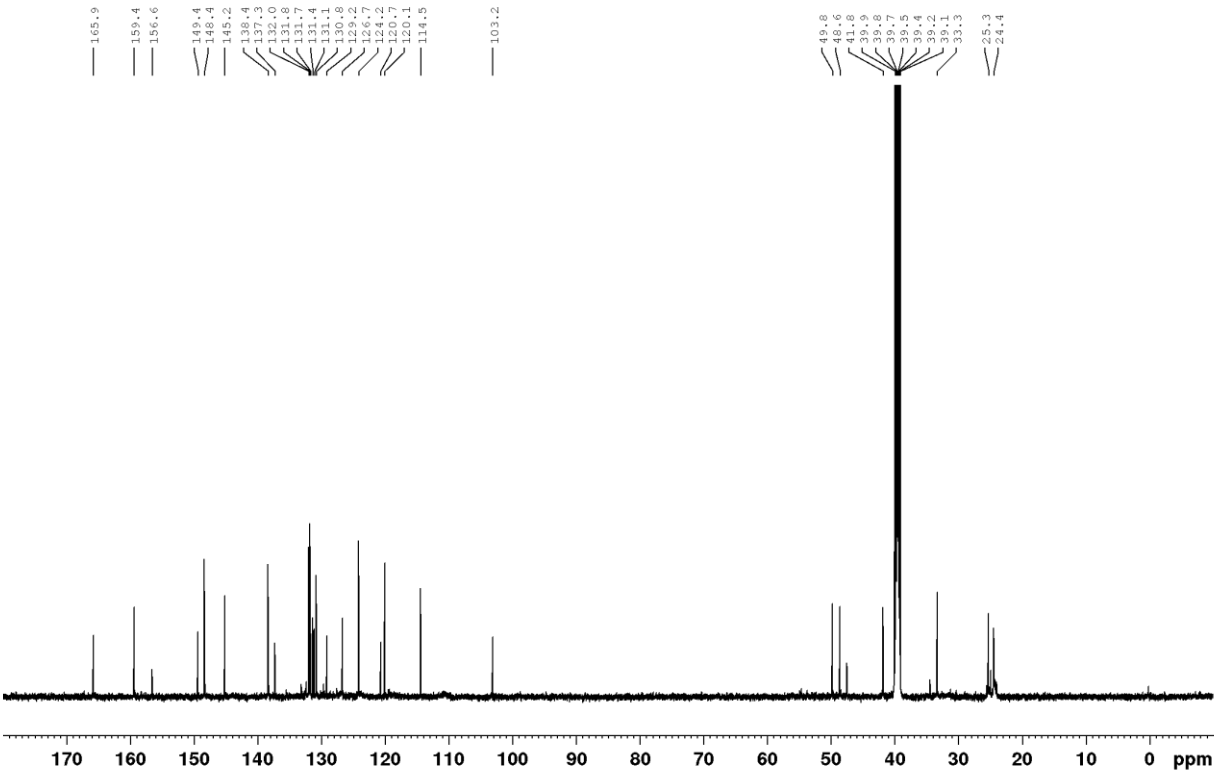

6. Literature

[1] DIN EN 46-1:2016-11, Wood preservatives - Determination of the preventive action against recently hatched larvae of Hylotrupes bajulus (Linnaeus) - Part 1: Application by surface treatment (laboratory method), German version.

[2] S. Kagabu, K. Yokoyama, K. Iwaya, M. Tanaka, *Biosci. Biotechnol. Biochem.*, 1998, **62**, 1216.

[3] A. Manicardi, A. Accetta, T. Tedeschi, S. Sforza, R. Marchelli, R. Corradini, *Artificial DNA: PNA & XNA*, 2012, **3**, 53.

[4] A. Manicardi, L. Guidi, A. Ghidini, R. Corradini, *Beilstein J. Org. Chem.*, 2014, **10**, 1495.

[5] J. C. Namyslo, D. E. Kaufmann, *Holzforschung*, 2009, **63**, 627.

[6] M. H. H. Drafz, S. Dahle, W. Maus-Friedrichs, J. C. Namyslo, D. E. Kaufmann, *Holzforschung*, 2012, **66**, 727.

[7] J. C. Namyslo, D. E. Kaufmann, C. Mai, H. Militz, *Holzforschung*, 2015, **69**, 595.

[8] C. Kaldun, S. Dahle, W. Maus-Friedrichs, J. C. Namyslo, D. E. Kaufmann, *Holzforschung*, 2016, **70**, 411.

[9] A. F. McKay, G. F. Wright, *J. Am. Chem. Soc.*, 1948, **70**, 430.

[10] B. C. Raju, V. J. Rao, *Indian J. Chem.*, 2002, **41B**, 2180.

[11] B. Latli, C. Than, H. Morimoto, P. G. Williams, J. E. Casida, *J. Label. Compd. Rad.*, 1996, **38**, 971.

[12] B.-K. Chen, US20040210054A1, 2004.

[13] V. Zapol’skii, J. Namyslo, M. Gjikaj, D. Kaufmann, *Synlett*, 2007, 1507.

[14] V. A. Zapol'skii, R. Fischer, J. C. Namyslo, D. E. Kaufmann, *Bioorg. Med. Chem.*, 2009, **17**, 4206.

[15] V. A. Zapol'skii, J. C. Namyslo, A. de Meijere, D. E. Kaufmann, *Beilstein J. Org. Chem.*, 2012, **8**, 621.

[16] R. Fischer, P. Jeschke, C. Erdelen, P. Lösel, U. Reckmann, D. E. Kaufmann, V. Zapol'skii, WO2003040129A1, 2003.

[17] A. K. Sharma, N. Kim, C. S. Cameron, M. Lyndon, C. B. Gorman, *Inorg. Chem.*, 2010, **49**, 5072.

[18] S. H. Pagire, H. S. Pagire, G. B. Lee, S.-J. Han, H. J. Kwak, J. Y. Kim, K. Y. Kim, S. D. Rhee, J. Im Ryu, J. S. Song, M. A. Bae, M.-J. Park, D. Kim, D. H. Lee, J. H. Ahn, *Eur. J. Med. Chem.*, 2015, **101**, 716.

[19] R. Kluger, V. D. Stefano, *J. Org. Chem.*, 2000, **65**, 214.

[20] A. M. S. Connolly, WO2000034254A1, 2000.

[21] P. Vianello, T. Bandiera, M. Varasi, US20030069236A1, 2003.

[22] N. B. Heine, A. Studer, *Org. Lett.*, 2017, **19**, 4150.

[23] A. Novoa, T. Eierhoff, J. Topin, A. Varrot, S. Barluenga, A. Imberty, W. Römer, N. Winssinger, *Angew. Chem. Int. Ed. Engl.*, 2014, **53**, 8885.

[24] J. F. Brady, D. P. Simmons, T. E. Wilson, WO2001042787A2, 2001.

[25] M. C. Sheikh, S. Takagi, M. Sakai, T. Mori, N. Hayashi, T. Fujie, S. Ono, T. Yoshimura, H. Morita, *Org. Biomol. Chem.*, 2011, **9**, 1244.
